# Shuffling of *cis*-regulatory elements is a pervasive feature of the vertebrate lineage

**DOI:** 10.1186/gb-2006-7-7-r56

**Published:** 2006-07-19

**Authors:** Remo Sanges, Eva Kalmar, Pamela Claudiani, Maria D'Amato, Ferenc Muller, Elia Stupka

**Affiliations:** 1 Telethon Institute of Genetics and Medicine, Via P. Castellino, 80131 Napoli, Italy; 2 Institute of Toxicology and Genetics, Forschungzenbrum, Karlsruhe, Postfach 3640, D-76021 Karlsruhe, Germany

## Abstract

**Background:**

All vertebrates share a remarkable degree of similarity in their development as well as in the basic functions of their cells. Despite this, attempts at unearthing genome-wide regulatory elements conserved throughout the vertebrate lineage using BLAST-like approaches have thus far detected noncoding conservation in only a few hundred genes, mostly associated with regulation of transcription and development.

**Results:**

We used a unique combination of tools to obtain regional global-local alignments of orthologous loci. This approach takes into account shuffling of regulatory regions that are likely to occur over evolutionary distances greater than those separating mammalian genomes. This approach revealed one order of magnitude more vertebrate conserved elements than was previously reported in over 2,000 genes, including a high number of genes found in the membrane and extracellular regions. Our analysis revealed that 72% of the elements identified have undergone shuffling. We tested the ability of the elements identified to enhance transcription in zebrafish embryos and compared their activity with a set of control fragments. We found that more than 80% of the elements tested were able to enhance transcription significantly, prevalently in a tissue-restricted manner corresponding to the expression domain of the neighboring gene.

**Conclusion:**

Our work elucidates the importance of shuffling in the detection of *cis*-regulatory elements. It also elucidates how similarities across the vertebrate lineage, which go well beyond development, can be explained not only within the realm of coding genes but also in that of the sequences that ultimately govern their expression.

## Abstract 2

Alignment of orthologous vertebrate loci reveals that a significant proportion of conserved *cis*-regulatory elements have undergone shuffling during evolution.

## Background

Enhancers are *cis*-acting sequences that increase the utilization and/or specificity of eukaryotic promoters, can function in either orientation, and often act in a distance and position independent manner [1]. The regulatory logic of enhancers is often conserved throughout vertebrates, and their activity relies on sequence modules containing binding sites that are crucial for transcriptional activation. However, recent studies on the *cis*-regulatory logic of Otx in ascidians pointed out that there can be great plasticity in the arrangement of binding sites within individual functional modules. This degeneracy, combined with the involvement of a few crucial binding sites, is sufficient to explain how the regulatory logic of an enhancer can be retained in the absence of detectable sequence conservation [2]. These observations together with the fact that we are still far from understanding fully the grammar of transcription factor binding sites and their conservation [3] make it difficult to assess the extent of conservation in vertebrate *cis*-regulatory elements.

Very little is known about the evolutionary mobility of enhancer and promoter elements within the genome as well as within a specific locus. Sporadic studies of selected gene families have addressed questions related to the mobility of regulatory sequences involving promoter shuffling [4] and enhancer shuffling [5]; these describe the gain or loss of individual regulatory elements exchanged between specific genes in a cassette manner [6]. These studies suggested that a wide variety of different regulatory motifs and mutational mechanisms have operated upon noncoding regions over time. These studies, however, were conducted before the advent of large-scale genome sequencing, and thus they were performed on a scale that would not allow the authors to derive more general conclusions on the mobility and shuffling of regulatory elements.

The basic tenet of comparative genomics is that constraint on functional genomic elements has kept their sequence conserved throughout evolution. The completion of the draft sequence of several mammalian genomes has been an important milestone in the search for conserved sequence elements in noncoding DNA. It has been estimated that the proportion of small segments in the mammalian genome that is under purifying selection within intergenic regions is about 5% and that this proportion is much greater than can be explained by protein-coding sequences alone, implying that the genome contains many additional features (such as untranslated regions, regulatory elements, non-protein-coding genes, and structural elements) that are under selection for biological functions [7-11]. In order to address this issue, sequence comparisons across longer evolutionary distances and, in particular, with the compact *Fugu rubripes *genome have been shown to be useful in dissecting the regulatory grammar of genes long before the advent of genome sequencing [12]. More recently, the completion of the draft sequence of several fish genomes has allowed larger scale approaches for the detection of several regulatory conserved noncoding features.

Several studies have addressed the issue of conserved noncoding sequences on a larger scale. A first study on chromosome 21 [13] revealed conserved nongenic sequences (CNGs); these were identified using local sequence alignments between the human and mouse genome of high similarity, which were shown to be untranscribed. A separate study focusing on sequences with 100% identity [14] revealed the presence of ultraconserved elements (UCEs) on a genome-wide scale, and finally conserved noncoding elements (CNEs) [15] were found by performing local sequence comparisons between the human and fugu genomes showing enhancer activity in zebrafish co-injection assays. Although the CNG study yielded a very large number of elements dispersed across the genome, and bearing no clear relationship to the genes surrounding them, the latter studies (UCEs and CNEs) were almost exclusively associated with genes that have been termed 'trans-dev' (that is, they are involved in developmental processes and/or regulation of transcription).

One of the major drawbacks of current genome-wide studies is that they rely on methods for local alignment, such as BLAST (basic local alignment search tool) [16] and FASTA [17], which were developed when the bulk of available sequences to be aligned were coding. It has been shown that such algorithms are not as efficient in aligning noncoding sequences [18]. To tackle this issue new algorithms and strategies have been developed in order to search for conserved and/or over-represented motifs from sequence alignments, such as the motif conservation score [19], the threaded blockset aligner program [20] and the regulatory potential score [21], as well as phastCons elements and scores [22]. However, all of these rely on a BLAST-like algorithm to produce the initial sequence alignment and are thus subject to some of the sensitivity limitations of this algorithm and do not constitute a major shift in alignment strategy that would model more closely the evolution of regulatory sequences.

Two approaches were recently reported which provide novel alignment strategies: the promoter-wise algorithm coupled with 'evolutionary selex' [23] and the CHAOS (CHAins Of Scores) alignment program [24]. Whereas the former has been used to validate a set of short motifs, which have been shown to be of functional importance, the latter has not been coupled to experimental verification to estimate its potential for the discovery of conserved regulatory sequences. Unlike other fast algorithms for genomic alignment, CHAOS does not depend on long exact matches, it does not require extensive ungapped homology, and it does allow for mismatches within alignment seeds, all of which are important when comparing noncoding regions across distantly related organisms. Thus, CHAOS could be a suitable method for the identification of short conserved regions that have remained functional despite their location having changed during vertebrate evolution. The only method available that attempts to tackle the question of shuffled elements and that makes use of CHAOS is Shuffle-Lagan [25]; however, it has not been used on a genome-wide scale and its ability to detect enhancers has not been verified experimentally.

Until recently our ability to verify the function of sequence elements on a large scale within an *in vivo *context was strongly limited. This task was eased significantly using co-injection experiments in zebrafish embryos [26], which allows significant scale-up in the quantity of regulatory elements tested; this is fundamental when one is trying to elucidate general principles regarding regulatory elements, the grammar of which still eludes us. The co-injection technique used to test shuffled conserved regions (SCEs) for enhancer activity was previously shown to be a simple way to test *cis*-acting regulatory elements [15,27,28] and was shown to be an efficient way to test many elements in a relatively short period of time [15].

The analysis described herein attempts to tackle the issue of the extent, mobility, and function of conserved noncoding elements across vertebrate orthologous loci using a unique combination of tools aimed at identifying global-local regionally conserved elements. We first used orthologous loci from four mammalian genomes to extract 'regionally conserved elements' (rCNEs) using MLAGAN [29], and then used CHAOS to verity the extent of conservation of those rCNEs within their orthologous loci within fish genomes. The analysis was conducted annotating the extent of shuffling undergone by the elements identified. Finally, we investigated the activity of rearranged and shuffled elements as enhancer elements *in vivo*. We found that the inclusion of additional genomes, the use of a combined global-local strategy, and the deployment of a sensitive alignment algorithm such as CHAOS yields an increase of one order of magnitude in the number of potentially functional noncoding elements detected as being conserved across vertebrates. We also found that the majority of these have undergone shuffling and are likely to act as enhancers *in vivo*, based on the more than 80% rate of functional and tissue-restricted enhancers detected in our zebrafish co-injection study.

## Results

The dataset described in this analysis is available on the internet [30] for full download, as well as the searchable to identify SCEs belonging to individual genes.

### Identification of mammalian regionally conserved elements

**Figure 1:**
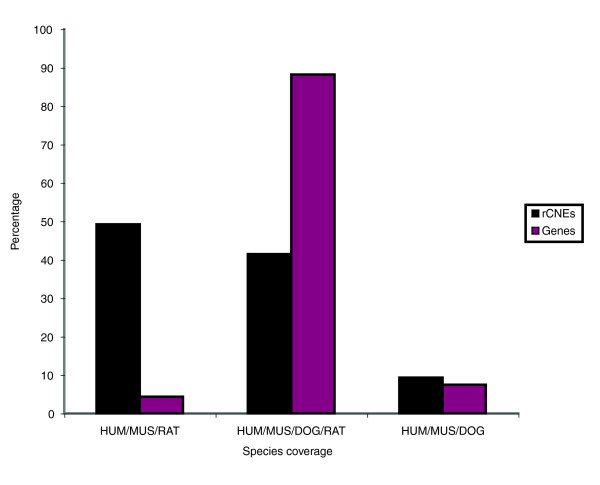
Number of conserved gene loci versus number of rCNEs identified in the mouse, rat, human, and dog genomes. Graph showing the number of rCNEs found conserved in the dog, rat, mouse and human genomes versus the number of genes found conserved across the same genomes. Although almost 90% of the genes can be found in all four genomes, most rCNEs can be found only in three out of four genomes. rCNE, regionally conserved element.

For each group of orthologous genes global multiple alignments among the human, mouse, rat, and dog loci were performed using MLAGAN [25]. We took into consideration all genes for which there were predicted othologs within Ensembl [31] in the mouse genome, human genome, and any third mammalian species, which led us to analyze 9,749 groups of orthologous genes (36% of the annotated mouse genes). Most genes (about 88%) were found to be conserved in all four species considered, with only about 12% found in three out of four species (about 6% in each triplet; Figure 1). For each locus we took into account the whole genomic repeat-masked sequence containing the transcriptional unit as well as the complete flanking sequences up to the preceding and following gene. This lead us to analyze 37% of the murine genome sequence overall. The alignments were parsed using VISTA (visualizing global DNA sequence alignments of arbitrary length) [32] searching for segments of minimum 100 base pairs (bp) length and 70% identity. We further selected these regions by only taking into account those regions that were found at least in mouse, human, and a third mammalian species and which overlapped by at least 50 bp, which resulted in a set of 364,358 rCNEs (Table 1). These were then filtered stringently to distinguish 'genic' from 'nongenic' (see Materials and methods, below). This analysis classified 22.7% of the resulting rCNEs as 'genic', while 281,644 nongenic elements account for about 46 megabases, or 1.77%, of the murine genome.

We further annotated mammalian rCNEs based on their position in the mouse genome with respect to the gene locus in order to define whether they were located before the annotated transcription start site (TSS; 'pre-gene'), within the intronic portion of the gene, or posterior to the transcriptional unit ('post-gene'). Approximately 54% of rCNEs were found to fall within intergenic regions, of which 37% were post-gene and 63% pre-gene (Table 1).

### Shuffling of conserved elements is a widespread phenomenon

**Figure 2:**
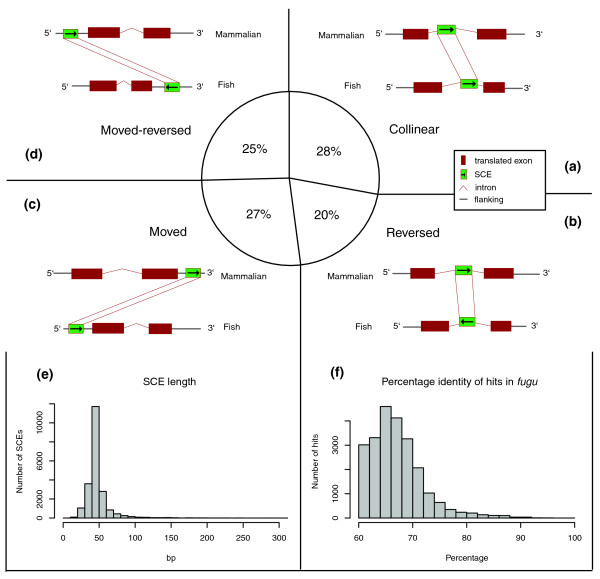
Distribution of length, percentage identity and shuffling categories of SCEs. SCEs were categorized based on their change in location and orientation in *Fugu rubripes *with respect to their location and orientation in the mouse locus. The entire locus, comprising the entire flanking sequence up to the next upstream and downstream gene was taken into consideration. Definitions of specific classes: **(a) **collinear SCEs (elements that have not undergone any change in location or orientation within the entire gene locus); **(b) **reversed SCEs (elements that have changed their orientation in the fish locus with respect to the mouse locus, but have remained in the same portion of the locus); **(c) **moved SCEs (elements that have moved between the pre-gene, post-gene and intronic portions of the locus); **(d) **Moved-reversed (elements that have undergone both of the above changes). **(e) **Frequency distribution of SCE length in base pairs. **(f) **Frequency distribution of percentage identity of SCE hits in fugu. SCE, shuffled conserved region.

We searched for conservation of rCNEs in teleost genomes using CHAOS [24], selecting regions that presented at least 60% identity over a minimum length of 40 bp as compared with the mouse sequence of the rCNEs. This method allowed us to identify regions that are reversed or moved in the fish locus with respect to the corresponding mammalian locus. For each locus in every species analyzed we took into account the whole genomic repeat-masked sequence containing the transcriptional unit as well as the complete flanking sequences up to the preceding and following gene. We defined as SCEs those regions of the mouse genome that were conserved at least in the fugu orthologous locus and filtered out any sequence shorter than 20 bp as a result of the overlap analysis with zebrafish and tetraodon (see Materials and methods, below, for details). Our analysis identified 21,427 nonredundant nongenic SCEs, which were found in about 30% of the genes analyzed (2,911; Table 2). The distribution of their length and percentage identity is shown in Figure 2e,f. The median length and percentage identity (45 bp and 67%, respectively) reflect closely the cut offs provided to CHAOS in the alignment (40 bp and 60% identity), although there is a significant number of outliers whose length is equal to or greater than 200 bp (223 elements whose maximum length is 669 bp) and whose median percentage identity is 74%. No elements were identified that were completely identical to their mouse counterpart (the maximum percentage identity found was 97%).

We decided to investigate further the extent to which the elements identified, which are still retained within the locus analyzed, have shuffled in terms of relative position and orientation relative to the transcriptional unit, and would thus be missed by a simple regional global alignment (such as MLAGAN). The results of this revealed that only 28% of elements identified have retained the same orientation and the same position with respect to the transcriptional unit taken into account (that is to say, have remained pre-gene, intronic, or post-gene. Labeled as 'collinear'; Figure 2a), whereas others have shifted in terms of orientation ('reversed'; Figure 2b), position ('moved'; Figure 2c), or both ('moved-reversed'; Figure 2d). Thus, almost two-thirds of the SCEs identified would have been missed by a global, albeit regional, alignment approach.

A possible explanation for the large number of noncollinear elements is that they could appear shuffled owing to assembly artifacts. In order to assess whether the large number of elements identified as noncollinear were merely due to assembly artifacts, we analyzed the number of SCEs containing a single hit in fugu and not classified as collinear that also had a match in tetraodon. If the shuffling were merely due to assembly artifacts, then we would expect approximately half of the noncollinear hits in fugu also to be noncollinear in tetraodon. The results, however, were significantly different, because more than 80% of the elements were not collinear in both species (*P *< 2.2 × e^-16 ^obtained by performing a χ^2 ^comparison between the proportion obtained and the expected 0.5/0.5 proportion). These findings emphasize that shuffling is a mechanism of particular relevance when searching for short, well conserved elements across long evolutionary distances and that its true extent can only be detected by using a sensitive global-local alignment approach, as opposed to a fast genome-wide approach [25].

**Figure 3:**
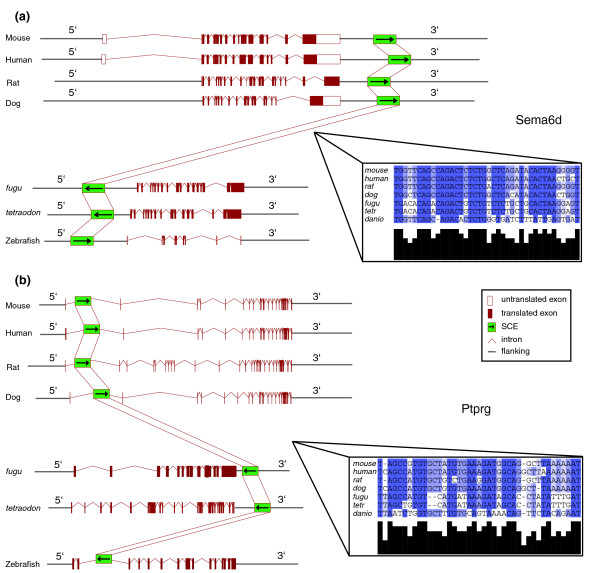
Examples of loci containing shuffled conserved elements. **(a) **The *Sema6d *(sema domain, transmembrane domain, and cytoplasmic domain, semaphorin 6D; MGI:2387661) locus contains a post-genic moved-reversed conserved element. The SCE is found downstream from the gene in mammalian loci and upstream of the gene in fish genomes, and in reverse orientation only in the genomes of fugu and tetraodon. **(b) **the *Ptprg *(protein tyrosine phosphatase, receptor type G; MGI:97814) locus contains an intronic moved-reversed conserved element. The SCE is found in the first intron of the *Ptprg *gene in mammalian genomes, downstream of the gene in reverse orientation in fugu and tetraodon, and in the second intron in reverse orientation in zebrafish. Boxes represent the multiple alignments of the SCEs identified. SCE, shuffled conserved region.

Two examples of SCEs that were identified in our study are shown in Figure 3. Example A shows the locus of *Sema6d*, a semaphorin gene that is located in the plasma membrane and is involved in cardiac morphogenesis. This locus represents a conserved element that is found after the transcriptional unit at the 3' end of the gene in all mammals analyzed, whereas it is located upstream in fish genomes and reversed in orientation in the fugu and tetraodon genomes. Example B shows the locus of the tyrosine phosphatase receptor type G protein, a candidate tumor suppressor gene, which has a conserved element in the first intron of all mammalian loci analyzed, which is found in reversed orientation in all fish genomes, downstream of the gene in the fugu and tetraodon genomes, and in the second intron in the zebrafish genome.

### Shuffled conserved regions cast a wider net of nongenic conservation across the genome

**Figure 4:**
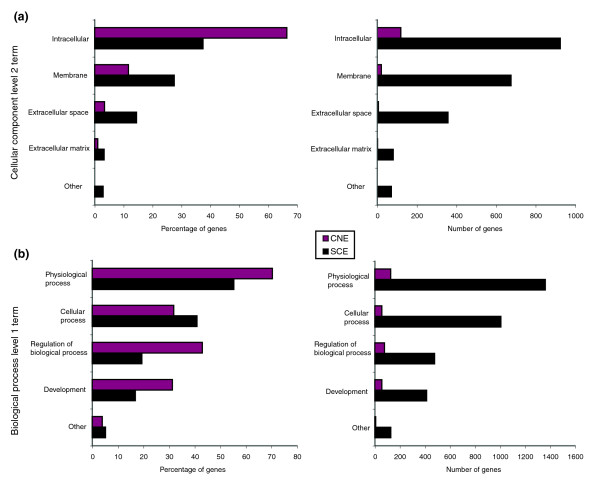
GO Classification of genes harboring CNEs versus genes harboring SCEs. All genes containing CNEs and/or SCEs were analyzed for GO term classification. Genes containing CNEs are shown in red and genes containing SCEs are shown in gray. Plots show differences in absolute numbers as well as relative percentages. Classification is shown for **(a) **cellular component and **(b) **biological process categories. CNE, conserved noncoding element; GO, Gene Ontology; SCE, shuffled conserved region.

We analyzed the type of genes that are associated with SCEs by assessing the distribution of Gene Ontology (GO) terms [33] using GOstat [34] (see Materials and methods, below). Although the results indicate significant over-representation of gene classes typical of genes harboring noncoding conservation ('trans-dev' enrichment) as reported previously (Additional data file 1), the number of genes within our analysis containing nongenic SCEs (2,911) is approximately an order of magnitude greater than that of the number of genes containing CNEs (330). The overlap between the two datasets is 291 genes, and so almost all (>88%) genes containing SCEs also contain CNEs. A GO analysis comparing genes containing CNEs and those containing SCEs (Figure 4) revealed that there are several GO categories that are significantly under-represented in the CNE dataset as compared with ours. These categories were not seen in the previous analysis (Additional data file 1) because they are not over-represented in our dataset as compared with the entire genome.

The most striking difference is found in the analysis by cellular components; there is an approximate 54-fold enrichment in genes belonging to the extracellular regions that contain SCEs as compared with genes in the same class that contain CNEs. In fact SCEs are present in more than 50% of the genes we were able to classify as belonging to the extracellular matrix and in 35% of those belonging to the extracellular space, whereas CNEs are only found in six and two such genes, respectively. These gene sets differ significantly in both extracellular regions and membrane GO cellular component categories (*P *< 0.001; Additional data file 1). Enrichments in the order of 10-fold to 13-fold are seen when comparing genes involved in physiological and cellular processes, respectively. For both of these categories our analysis was able to identify SCEs in more than 30% of the genes belonging to this class. The differences, although substantial (about sevenfold) are not as extreme when comparing 'trans-dev' genes (genes categorized as belonging to the 'regulation of biological process' and 'development' using GO) because the CNE dataset has a stronger bias for those genes (*P *< 0.001; Additional data file 1). Finally, although we identified SCEs in 40% of genes assigned to the 'behavior' class, none of the genes in this class has CNEs. The data thus suggest that there are both quantitative and qualitative differences between the two datasets.

### The proximal promoter region is a shuffling 'oasis'

Because a large proportion of our dataset undergoes shuffling, we decided to investigate whether shuffling is a property that is dependent on proximity to the transcriptional unit. To address this question we divided our dataset of nongenic SCEs between collinear (as discussed above) and noncollinear (all other categories discussed above taken together) elements, and analyzed the distribution of their distances from the TSS (pre-gene set), the intron start (intron start), the intron end (intron-end set) and the 3' end of the transcript (post-gene). This analysis demonstrated that collinear elements were distributed significantly closer to the start and the end of the transcriptional unit compared with noncollinear elements, whereas no differences were observed in terms of proximity to the intron start and intron end (Additional data file 2).

**Figure 5:**
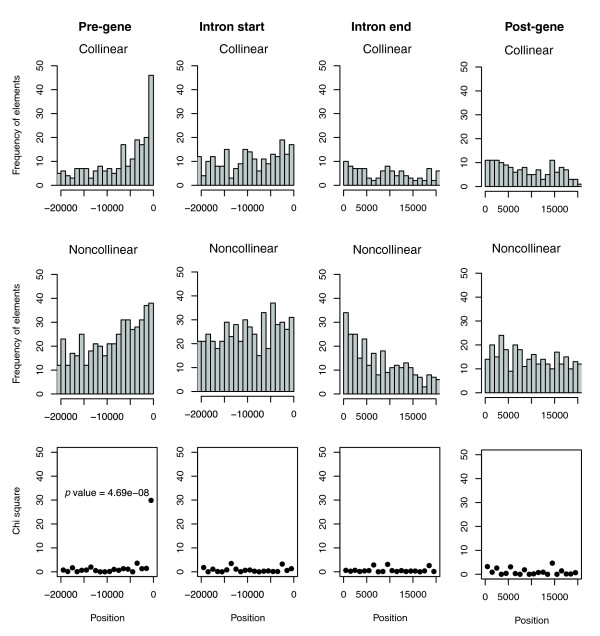
Analysis of SCE shuffling in 1000 bp windows. Each column in the figure shows the analysis of a locus portion (pre-gene, intron-start, intron-end and post-gene) divided into 1000 bp windows. In each column the first graph indicates the number of collinear SCEs identified, the second graph the number of noncollinear SCEs identified, and the third graph the χ^2 ^test used to identify windows that show a significant deviation from the expected proportion of collinear to noncollinear SCEs. The *P *value is shown for the only window (1000 bp upstream of the transcription start site) that exhibits significant deviation from the expected proportion. bp, base pairs; SCE, shuffled conserved region.

In order to investigate this phenomenon at higher resolution, we subdivided all loci analyzed in our dataset into 1,000 bp windows within the areas, and verified whether the proportion of collinear versus noncollinear elements deviated significantly from the expected proportions in any of these windows (see Materials and methods, below, for details). The results of the analysis are shown in Figure 5. The only window that exhibited a high χ^2 ^result with significantly less shuffled elements than collinear ones (*P *= e^-08^), was the 1,000 bp window immediately upstream of the TSS. No similar results were found in any other 1,000 bp windows across the gene loci analyzed. Similar results were obtained when deploying other window sizes (data not shown). To ascertain whether the result observed was due to annotation problems, we inspected the GO classification of the genes that presented nongenic collinear elements in the 1,000 bp window discussed above and observed significant enrichment (*P *< 0.001) for 'trans-dev' genes, whereas the same test conducted on genic collinear elements in the same window revealed no significant GO enrichment (Additional data file 3).

### Shuffled conserved regions are able to predict vertebrate enhancers

**Figure 6:**
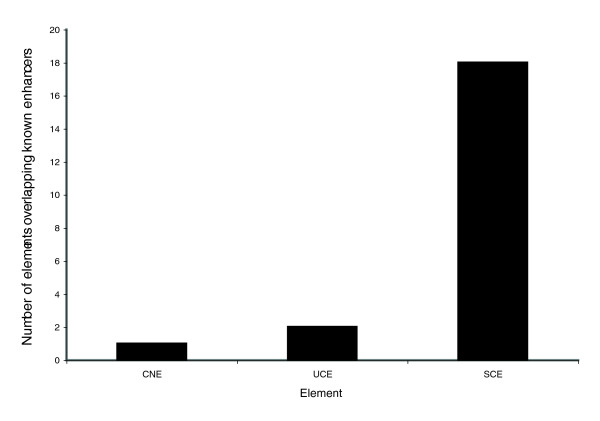
Overlap of known mouse enhancers with conserved elements. All mouse enhancers deposited in GenBank (94) were mapped to the genome and compared with previously published conserved elements (UCEs and CNEs) as well as our own dataset of SCEs to verify their overlap. Only one known mouse enhancer is overlapped by a CNE and two by a UCE, whereas our dataset of SCEs identifies 18 known mouse enhancers as being conserved within fish genomes. CNE, conserved noncoding element; SCE, shuffled conserved region; UCE, ultraconserved element.

In order to verify the ability of SCEs to predict functional enhancer elements, we conducted an overlap analysis (see Materials and methods, below) of SCEs with 98 mouse enhancer elements deposited in Genbank. We compared the overlap of SCEs with that of two other datasets that present conservation in fish genomes, namely CNEs and UCEs. The results presented in Figure 6 show that although CNEs and UCEs are able to detect only one and two known enhancers from our dataset, respectively, SCEs detect 18 of them successfully.

### Shuffled conserved regions act as enhancers *in vivo*

**Figure 7:**
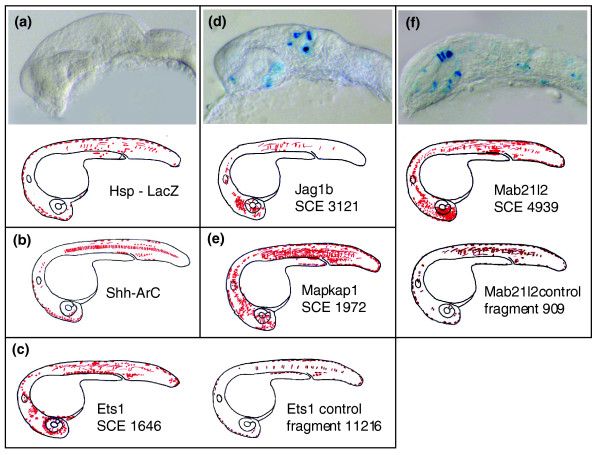
Expression profiles of X-Gal stained embryos. **(a-f) **Expression profiles of 1-day-old X-Gal stained zebrafish embryos. Each expression map represents a composite overview of the *LacZ*-positive cells of 65-175 embryos. Gene names and fragment/SCE id are shown. Detailed distribution of X-Gal stained cells in different tissues as well as data for all other fragments are shown in Table 3. Side view of head region of *LacZ*-stained embryos are shown with anterior to the left. (panel a) *HSP*-*lacZ *injected embryo. **(d) **Embryo co-injected with SCE 3121 associated with *Jag1b *gene. **(f) **Embryo co-injected with SCE 4939 associated with Mab21l2 gene. SCE, shuffled conserved region.

In order to validate the *cis*-regulatory activity of SCEs we chose a subset of SCEs to be tested for *in vivo *enhancer activity by amplifying them from the fugu genome and co-injecting them in zebrafish embryos with a minimal promoter-reporter construct yielding transient transgenic zebrafish embryos. Twenty-seven SCEs were tested, of which four overlapped known mouse enhancers for which activity had not previously been reported in fish, and the remaining 23 (from 12 genes, of which four were not trans-dev genes, for a total of eight fragments not associated with trans-dev genes) did not overlap any known feature. Detailed information on each SCE tested, including diagrams of their localization in mammalian and fish genomes as well as multiple alignments, is shown in Additional data file 4. As a control set 12 noncoding, nonrepeated, and nonconserved fragments were also chosen for co-injection assays, of which nine were from the same genes from which SCEs had been picked and three were from random genes (see Materials and methods, below, for details). Owing to the mosaic expression patterns that are obtained with this technique, results were recorded in two ways: by counting the number of cells stained for X-Gal and recording, where possible, the tissue in which the LacZ-positive cells were found; and by plotting LacZ-positive cells on expression maps that represent a composite overview of the LacZ-positive cells of all the embryos tested. Results of the cell counts are shown in Table 3 (For greater details, see Additional data file 3) and the expression maps are shown in Figure 7. The cell counts were used to define statistically which fragments exhibited tissue-restricted enhancer activity or generalized enhancer activity (see Materials and methods, below).

As a positive control a published regulatory element from the *shh *locus, *ar-C *[27], was coinjected with the *HSP:lacZ *fragment. From a total of 27 SCEs, 22 (about 81%) were able to enhance significantly the activity of the *HSP:lacZ *construct in comparison with the embryos injected with *HSP:lacZ *only (see Materials and methods, below, for details). Of these, three out of the four tested known mouse enhancers that were found to be conserved in fish were confirmed to act as enhancers in fish. A similar percentage of positive results (82.6%) was obtained excluding these enhancers in the count. The enhancer effect in 20 out of the 22 positive SCEs was not generalized but observed in a tissue-restricted manner.

The expression patterns obtained in our experiments were compared with expression data retrieved from the Zebrafish Information Network [35,36]. Multiple SCEs found within a single gene locus gave similar tissue-restricted enhancer activity. For example, all four SCEs tested from the *ets-1 *locus gave expression that was highly specific to the blood precursors (SCE 1646 in Figure 7c). This result is in accordance with reported data, which showed *ets-1 *expression in the arterial system and venous system. Moreover, both elements tested from the *zfpm2 *(also described as *fog2 *[37]) gene gave central nervous system (CNS) specific enhancer activity, which is in accordance with a recent report showing that the expression of both *fog2 *paralogs is restricted to the brain [37]. Similarly, elements tested from the *mab-21*-like genes gave CNS and eye specific enhancer activity (SCE 4939; Figure 7f). This pattern of expression corresponds with the patterns reported in the brain, neurons, and eye [38,39]. The SCEs that were found in the *pax6a *and *hmx3 *genes were shown to give CNS specific enhancement, which is in accordance with the reported expression of these genes in the CNS [35]. Finally, SCE 3121 from the gene *jag1b *gave specific expression in the CNS and in the eye (Figure 7d), which is in partial agreement with reported expression of this gene (expressed in the rostral end of the pronephric duct, nephron primordia, and the region extending from the otic vesicle to the eye [40]).

Novel enhancer functions were also detected for SCEs neighboring *lmx1b1*, which showed CNS specific activity, and SCEs neighboring four genes not belonging to the trans-dev category, such as *mapkap1 *(Figure 7e), *tmeff2 *and *3110004L20Rik *(producing proteins integral to the membrane), and *elmo1 *(associated with the cytoskeleton), which exhibited strong generalized and/or tissue specific activity. No endogenous expression data are available for these genes for comparison. In contrast to the results with SCE elements, only two out of 12 (about 17%) of the genomic control fragment set derived from the same loci of the SCEs exhibited significant enhancement of LacZ activity (Table 3).

Taken together, these data demonstrate that SCEs act as *bona fide *enhancers that can drive tissue-restricted as well as generalized expression during embryo development.

## Discussion

### Widespread shuffling of *cis*-regulatory elements in vertebrates

In this study we demonstrate, using a unique combination of tools aimed at obtaining regional, global-local sensitive alignments applied at the genome level, that the number of conserved non-coding sequences shared between mammalian and fish genomes is at least an order of magnitude higher than was previously proposed and is spread across thousands of genes. In fact, approximately 30% of the genes analyzed presented at least one SCE. Our GO analysis results indicate a 'trans-dev' bias similar to those described in previous studies addressing genes exhibiting noncoding conservation [14,15]. On the other hand, the significant increase in the sheer number of elements identified and in the number of genes exhibiting SCEs enabled us to detect conserved nongenic elements in a third of the genes studied, indicating that conservation of *cis*-regulatory modules is a widespread phenomenon in vertebrates, and is not limited to a few hundred genes, as suggested by previous studies. The GO analysis also revealed that certain classes of genes, such as those located in the extracellular space and extracellular matrix, exhibit conserved non-coding sequences, which were not identified with previous approaches and indicate that noncoding elements conserved across vertebrates are present in a larger and more diverse set of genes than was previously thought. Although we also observed a larger number of genes involved in cellular and physiological processes, many of them are also assigned to 'trans-dev' categories, and so their involvement in development and regulation of transcription cannot be excluded. Indeed, it is important to note that eight out of the 23 randomly selected fragments were not associated with trans-dev genes by GO classification, and that six of these fragments exhibited significant enhancer activity in our co-injection assays (Table 3). This confirms that conservation is not an exclusive characteristic of regulatory regions associated with trans-dev genes.

That shuffling plays an important role in the identification of conserved non-coding sequences is illustrated by the fact that 72% of our dataset was observed to be either inverted or moved, or both, in the fish locus with respect to the mouse locus. Assembly artifacts are unlikely to be an important factor in the elements identified as shuffled because they would also affect gene structures and therefore correct gene prediction and ortholog detection, which is at the basis of our dataset. We were reassured about this by our tetraodon-fugu comparison, which indicated that most elements found to be shuffled in one species were also shuffled in the other. A notable exception to the general shuffling bias in the elements found was a 1,000 bp window immediately upstream of the TSS. Taking into account that the proximal promoter region is considered to be approximately -250 bp to +100 bp from the TSS [41], and assuming that TSS annotations in the mouse genes analyzed are precise, this finding suggests that there is a class of enhancer elements that are more constrained in both position and orientation, perhaps working in tight connection to the promoter complex. The fact that the genes containing nongenic collinear elements in this window show the 'trans-dev' bias associated with our overall SCE dataset, as well as with previous analyses of noncoding conservation, reassures us that this result is not a mere product of bad annotation of the first exon in these genes. It is particularly reassuring that performing the same analysis on SCEs found in the same window but classified as 'genic' (and thus more likely to be real evidence of annotation problems) did not exhibit this bias.

Lack of conservation can also be due to the fact that the evolution of regulatory motifs involves constant *de novo *creation and destruction of them over time because of their short sequences and plastic nature [42] (for review [43]). The dissection of *cis*-regulatory elements from different species, however, indicates clearly that there are cases in which although the same transcription factors are involved in the regulation of a gene, all sequences that are not responsible directly for the binding of transcription factors are not preserved and so overall sequence conservation is very poor [2]. Thus, the quest to identify regulatory conservation must be complemented by a more thorough understanding of the inherent grammar of regulatory sequences, which would lead to improved alignment models specifically tailored to regulatory sequences [23].

### Conservation versus function

During the past few years several strategies have been deployed to perform genome-wide sequence comparisons, which in turn identified several novel functional elements in vertebrate genomes. However, they have not yet defined how far conservation of noncoding elements can be pushed to identify functional elements efficiently. The approach used to build our dataset is significantly different from previous approaches, because on the one hand it is stringent by focusing on fish-mammal comparisons and on the other hand it is more sensitive than previous approaches because of its CHAOS-based alignments and lower length cut offs. The requirement for conservation in fish genomes in the SCE dataset would thus lead to the loss of mammalian-specific enhancers, but on the other hand it is likely to act as a stringent filter for slowly evolving DNA that may be free from any functional constraints. The differences between the SCE dataset and previously reported datasets became evident by performing an overlap analysis among them (see Materials and methods, below, for details; also see Additional data file 5). The partial overlap between the analyzed datasets once again emphasizes that the approach used to determine conserved nongenic elements has a notable impact on the elements identified. Approximately 50% of SCEs do not overlap any known feature, suggesting that the use of nonexact seeds for the initial local alignments has a significant impact on the analysis of noncoding DNA harboring short, well conserved elements, and that our dataset is substantially different from previous datasets both quantitatively, and qualitatively.

UCEs were detected using a whole-genome local alignment strategy between human and mouse (although they are often conserved in fish genomes as well) and selected for being 100% identical over at least 200 bp [14]. They were shown to be often located in clusters in the proximity of 'trans-dev' genes. Poulin and coworkers [44] showed that the ultraconserved Dc2 element is necessary and sufficient for brain tissue enhancer activity, and an ongoing systematic study using transgenic mice has shown enhancer activity for more than 60% of the elements tested so far (Pennachio and coworkers, unpublished data). Our dataset overlaps only 45% of the UCE elements because of its 'regional approach', which will miss any elements that are conserved across nonorthologous loci or that are found beyond the region we took into consideration (namely, beyond the previous or next gene). Nonetheless, the results of our study indicate that the enhancer function that has so far been associated with them does not explain fully their level of conservation, because our dataset, although rich in enhancers, has much lower levels of sequence identity and length as compared with UCEs. Only one of the fragments that we tested (SCE 1973 from the *mapkap1 *gene) overlaps with a UCE element. The overlap is only 33 bp, and there is no further identity with the UCE in fugu, but the element nonetheless acted as a tissue-restricted enhancer *in vivo*. A region adjacent to the UCE in mouse (SCE 1973), although not ultraconserved, is also conserved in fish and acted as a generic enhancer in our assays, highlighting the complexity of these regions and adding to the ongoing debate regarding their function and evolution [45].

A large set of sequences, defined as CNGs, was constructed by using pair-wise local sequence comparison between the human and mouse genome on chromosome 21 (identity = 70%, length = 100 bp), and it was shown that two-thirds of them lacked transcriptional evidence *in vivo *[13]. The conservation of these regions in other mammalian genomes was later also confirmed [8]; however, thus far they have not been shown to represent functional regulatory elements to a satisfactory scale, and so the specificity of this method in the identification of enhancers is not known. A recent genome-wide study of functional noncoding elements conserved in fish genomes used pair-wise local sequence comparison between the human and fugu genomes to define 1,400 highly conserved noncoding elements (length = 100) and found that these were principally associated with developmental genes [15]. The overlap analysis highlights that although CNGs are three orders of magnitude larger than UCEs and CNEs and they contain the former fully and 96% of the latter, they only overlap approximately half of the SCE dataset. This suggests that there are qualitative differences between CNGs and our dataset. Interestingly, it has been shown that megabase deletions of two-gene deserts containing thousands of CNGs in mice had no phenotypic effects [46]. The authors stated that none of the CNGs contained are conserved in fish, and when we inspected these regions we discovered only a single SCE, very close to the boundary of the deletion.

Our dataset overlaps only 51% of the CNEs within the loci analyzed, probably because of the regional approach taken, which disregards elements conserved across nonorthologous loci. On the other hand more than 88% of the genes that contain CNEs also present SCEs, thus identifying regulatory elements in the majority of those genes nonetheless. A group of CNEs were shown to act as enhancers when tested *in vivo *in zebrafish by co-injecting them with promoter/reporter constructs. Our data, compared with the CNE dataset, is a radical extension (of an order of magnitude) of similar conserved elements, indicating a significant quantitative difference. There is also a qualitative difference, however, because we identified elements in a very broad range of genes, including genes from the extracellular regions and membrane and many genes participating in physiological and cellular processes, which are not transcription factors. The quantitative and qualitative differences in our dataset constitute a major departure from previously published datasets, which show conservation across vertebrates and clear evidence of involvement in enhancing gene expression, namely CNEs and UCEs.

Thus, the lack of overlap between the datasets taken into consideration is probably a compounded effect of methodological differences (for example, CNEs versus SCEs), real biological differences (CNGs versus others) and a compound effect of the two differences (UCEs versus CNEs and SCEs). Our results suggest that a large portion of the noncoding genome is composed of enhancers. Although it is certain that conserved noncoding regions play other roles that we were unable to verify, either they constitute a minority or they are able to perform several functions besides that of enhancers.

Comparative genomics has been applied successfully to the study of regulatory elements in the past, using approaches based on motif libraries. Xie and coworkers [19] aligned the promoter and 3'-untranslated region sequences from four mammalian genomes by using BlastZ with a regional approach and were able to identify motifs that were over-represented in conserved regions around genes. They showed that these motifs are nonrandomly distributed with respect to gene expression data but they did not identify specific instances of the motif as active copies in the genome. Thus, this study, apart from using a different methodology, focused on mammalian genomes only (as compared with our vertebrate-wide approach) and focused on proximal 5'- and 3'-untranslated region sequences, discarding introns as a negative control set based on the assumption that they contain few regulatory elements. Our study was based on sequence alignment, focused on a broader dataset comprising several vertebrate genomes and made use of the full intergenic and intronic sequence for each locus taken into consideration.

Ettwiller and coworkers [23] proposed a novel computational method that also makes use of comparative genomics. First, they developed a novel alignment routine, called promoterwise, that models promoter evolution more closely. Then, they used an efficient method to allow direct enumeration of all possible motifs up to 12-mers, including motifs with wildcards. Finally, active instances of the motif set thus generated were confirmed by searching them in regions that were found to be conserved in the alignment routine. This work was aimed at comparing distantly related genomes, by searching for over-representation in related orthologs across mammalian and fish genomes to identify specific instances of these motifs. Moreover, they proved using experiments in Medaka that these active motifs are necessary to drive expression *in vivo*. This study resembles our strategy more closely because it involves a vertebrate-wide comparison, although it focused only on 5 kb promoter sequences.

Motif library based approaches are complementary to our alignment focused approach. One important difference between these approaches is that the computational requirements of motif-based approaches are very high, and so it is not feasible to execute a motif library approach over a third of the genome sequence, as was done in this work. On the other hand motif library approaches are able to pinpoint specific motifs that are at the core of the regulatory grammar, whereas our approach uncovers a dataset that is likely to contain a redundant set of regulatory motifs. It would be a natural extension of our work to compare these datasets in order to elucidate shuffling and determine the extent to which enhancers can be represented as clusters of simpler motifs as well as to investigate shuffling of enhancers in relation to the shuffling of single motifs.

### Toward improved detection of *cis*-regulatory elements

The fact that, despite an increase of an order of magnitude in our dataset, a similar ratio of elements was found to act as enhancers as compared with the CNE dataset suggests that the extent of sequence conservation of regulatory elements is a moving target that reflects the technique used to identify them. There is a clear need for novel methodologies to detect thus far hidden conserved elements. The algorithm Shuffle-LAGAN is an alignment program that resembles our approach, although it only aligns shuffled elements within pair-wise alignments and therefore it would have not helped to bypass the initial step of selecting rCNEs found conserved in at least three mammalian genomes. A desirable extension of Shuffle-Lagan would be to add the ability to process orthologous loci from several genomes at once. More knowledge about the evolution of noncoding DNA will be needed in order to obtain better scoring schemes and thus yield not only sensitive alignments but more reliable predictions of enhancers and other regulators of gene expression [25].

An important aspect that differentiates our approach from previous BLAST-based approaches is the use of CHAOS for the alignment of mammalian loci to fish loci. In order to verify the extent to which CHAOS differs from BLAST in this particular type of search, we performed the search for SCEs from our set of rCNEs in the fugu genome, comparing NCBI BLAST and CHAOS at different word sizes and identical length and identity cut offs. The results indicate that although CHAOS scales exponentially as word size decreases, the number of hits obtained with BLAST is almost unaltered by the difference in word size. Moreover, there is a qualitative difference in the hits obtained because the increase in number of elements identified at small word sizes using CHAOS is due in large part to shuffled elements that BLAST is unable to identify (Additional data file 6). This qualitative difference is most notable using word size 10, for which only about 4% of BLAST results are shuffled elements as compared with 72% of the elements identified by CHAOS.

This significant difference reiterates quite clearly that looking for sequence similarity across long stretches of identical words is not a valid approach to identifying conserved regulatory elements. At the same time, if we were to decrease word sizes to what would be biologically sensible (that is to say, word size 5-8, similar to the size of transcription factor binding sites) it would be difficult to assess whether the elements identified as conserved were the result of convergent transcription factor binding site architecture generated *de novo*, rather than truly conserved across vertebrate evolution. Thus, novel methodologies need to be developed that would make use of small word sizes but include other constraints and scoring systems that would help to distinguish biological features preserved through evolution from neutrally evolving short fragments in the genome. To this extent, a well curated resource collecting known enhancers (deposited in GenBank, for example) as well as a large set of systematically validated enhancers (such as Enhancer Browser [47]; Pennacchio LA, unpublished data) would help in building valid scoring systems and improve current methods.

### *In vivo *transient assays

Our *in vivo *assays by co-injection revealed interestingly that most enhancers identified using this method were restricted in their activity to one or two tissues. Reassuringly, the expression profile of 24-hour-old embryos co-injected with the *ArC *positive control exhibited clear notochord enhancement (Figure 7b), as described previously [27]. The relative evolutionary closeness of fugu and zebrafish implies that expression and regulation of expression of developmentally regulated genes is probably well conserved [15,48]. Very little is known about *Fugu *gene expression patterns, but the availability of gene expression pattern information for many zebrafish genes provides a reliable assessment for the tissue specificity of the *Fugu *SCEs tested in our transient transgenic embryo assays. The functional analysis of SCEs by enhancer essays carried out in the transient transgenic zebrafish identified several new tissue restricted enhancer functions for genes where the endogenous expression pattern is not known. Future work will be required to analyze the role of these enhancers in relation to the detailed analysis of expression patterns of the genes they are associated with. In several cases the SCEs found within a locus provided tissue specificity reminiscent of the gene expression pattern of the flanking gene, arguing strongly for a direct role of these SCEs in regulating the expression of the flanking gene. It will, however, only be possible to prove unequivocally that there is a need for these enhancers to drive the expression of the candidate gene by site-specific mutation of the SCEs in the genomic context. Two of the control fragments that do not contain detectable conservation were also shown to have significant enhancer effect, and in particular one of the two exhibited activity that was greater than that of most SCEs tested.

### Mechanisms for genome-wide shuffling

Genomic rearrangements have already been reported on a large scale in a study examining gene order in regions of synteny between human and *Takifugu rubripes *[49]. Similar rearrangements should be seen when analyzing smaller regulatory regions that could harbor enhancers, which have strong evolutionary constraints on their sequence but frequently not on their specific localization with respect to the gene they act upon. We found that shuffling and rearrangements are not only applicable to nongenic sequences but also are a widespread phenomenon that involves 30% of the genes we analyzed.

Recently, there has been discussion on the role of *cis*-regulatory elements in the spatial organization of the genome and their possible role in restricting chromosomal rearrangements (see Liu and Garrard [50] and the review by Pederson [51]). The most well known examples of this are the hox clusters, although they do exhibit wider plasticity in fish genomes than in other genomes. Our work shows clearly that shuffling of *cis*-regulatory elements is a widespread phenomenon within orthologous loci. It would be interesting to investigate further the extent to which shuffling occurs on a genome-wide scale. Further analysis is required to determine the real extent of this phenomenon outside orthologous loci. This is the first genome-wide study to show that regulatory elements are mobile across species; this finding should be taken into consideration when using comparative evolutionary methods to locate potential regulatory elements.

It would be useful to assess the extent of shuffling on a genome-wide basis to develop a thresholding statistic. We investigated this by searching for SCEs in fugu nonorthologous loci. Although this results in a significantly lower number of hits (23,100 hits in orthologous analysis, 9,884 in nonorthologous analysis; *P *< 2.2 × e^-16^), the result shows that shuffling does occur outside of the orthologous locus. It is difficult to interpret this result without taking into account other data (for example, expression data and sequence similarity for genes considered nonorthologous or, indeed, *in vivo *assays on hits in nonorthologous loci) that would allow us to establish the extent to which hits in nonorthologous loci are noise and to which they represent regulatory elements in genes with similar expression patterns. Finally, we must emphasize that the fact that our mammalian rCNE dataset is built using a global alignment approach will limit the search space and will not allow us to investigate the extent of regulatory element shuffling within mammals. This data reduction step has been used in the past [52], and it was used in the present analysis based on the assumption that shuffling of regulatory elements is more likely to occur over longer evolutionary distances. Widespread shuffling of elements could act as a potential mechanism for providing new expression sites to genes that are placed in the vicinity of a translocated enhancer. These issues can only be tackled appropriately by performing further analysis of the extent to which conserved elements shuffle beyond their locus of origin on both small and large evolutionary distances.

## Conclusion

Our work shows that shuffling of *cis*-regulatory regions is a widespread phenomenon across the vertebrate lineage that affects approximately 70% of the conserved noncoding elements identified. The approach used allowed us to demonstrate that there is an order of magnitude more conserved elements in the vertebrate lineage than has previously been shown. Moreover, conservation of regulatory elements occurs over thousands, rather than hundreds, of genes. By casting a wider net over vertebrate noncoding conservation, we were able to demonstrate that there are hundreds of genes that do not belong to the 'trans-dev' category, such as genes found in the membrane and extracellular regions, which also contain conserved noncoding elements. Finally, our *in vivo *assays prove that although we cast a wider net, the catch was just as rich; more than 80% of the elements tested acted as enhancers, and the majority of them showed tissue-restricted patterns of expression in line with the neighboring gene.

## Materials and methods

### Selection of genes and sequences

Groups of homologous genes from the genomes of *Mus musculus*, *Homo sapiens*, *Canis familiaris*, *Rattus norvegicus*, *Takifugu rubripes*, *Tetraodon nigroviridis*, and *Danio rerio *were selected from the Ensembl-compara database [13] and their sequences were obtained from Ensembl database release 32 [14]. Genes were considered homologous if they were classified as best reciprocal hits in Ensembl-compara. We analyzed all of the genes that were conserved in at least four species, of which three had to be human, mouse and fugu, and one could be either dog or rat. This selection led to 9,749 groups of homologous genes. For each gene we analyzed the whole genomic repeat-masked sequence containing the transcriptional unit as well as the complete flanking sequences up to the next gene upstream and the next gene downstream. The region was extracted from Ensembl and the 5'-3' sequence of the locus was stored in a custom database (all mouse genes were stored as being in forward strand on the sequences stored). In cases in which the Ensembl gene contained multiple transcripts, the longest transcript was taken into consideration for the pre-gene, post-gene, and intron assignments of SCEs, but all exons (including those of other transcripts) were used to mask the sequence from coding regions. Similarly, if there were nested genes present in the locus, they were not taken into consideration to determine the extent of sequence to analyze, but they were taken into consideration to mask coding sequences in the region.

### Identification of mammalian regionally conserved elements

Global multiple alignments among human, mouse, rat, and dog were performed on each group of homologous genes using MLAGAN [25] with default parameters. The multiple alignments thus obtained were parsed using VISTA [32] with a window of 50 bases searching for conserved segments of at least 100 bp having a percentage identity of at least 70%. From these regions we selected as rCNEs only those regions that were shared and overlapped in at least mouse, human, and a third mammalian genome (either dog or rat) with a minimum length of 50 bp. In cases in which the upstream region of an analyzed gene coincided with the downstream region of another analyzed gene, rCNEs were counted only once.

### Identification of shuffled conserved regions

Mouse rCNEs were used as query sequences against the respective fugu, zebrafish, and tetraodon homologous sequences using CHAOS [24] on both strands with the following parameters: word length 10, score cut off 10, rescoring cut off 1,000, and BLAST-like extension on. Other parameters were left as set by default including the degeneracy tolerance of 1 (allowing a single mismatch in the seed of the alignment). The hits thus obtained were filtered to retain only those with at least 60% identity and 40 bp length. Although three genomes were queried, a hit in *Fugu *was required to consider the result an SCE. All other hits (if any) were used to select the region of overlap as the final SCE, but only SCEs greater than 20 bp after the overlap analysis were taken into consideration.

### Gene Ontology analysis

Ensembl gene IDs were converted into the corresponding RefSeq IDs before the analysis. The GOstat program [34] was used to find statistically over-represented GO IDs in the groups of genes, using the 'goa_mouse' GO gene association database as a reference. The false discovery rate and the *P *value cut off of 0.001 options were used. Raw output was converted in supplementary tables using a custom Perl script. The simple association of genes to GO classes presented in Figure 4 were produced using DAVID version 2 [53].

### Mapping of conserved elements

rCNEs and SCEs were classified as 'genic' if they overlapped any Ensembl genes, Ensembl expressed sequence tag (EST) genes [31], ESTs [54], EMBL proteins [55], or Genscan predictions [56] from the Ensembl *Mus musculus *genome build release 32. Furthermore, each rCNE and SCE was classified with respect to the gene structure as 'pre-gene', 'intronic', and 'post-gene' based on its location within these three portions of the locus. According to this 'gene-centric' classification, as well as the strand of the fugu CHAOS hits (because all genes were stored in forward strand), SCEs were classified as 'collinear' (that is to say not changed in orientation and not shifted between gene portions), 'moved' (shifted between gene portions), 'reversed' (changed in orientation, but retained in the same gene portion), and 'moved-reversed' (changed in orientation as well as shifted in gene portion).

### BLAST versus CHAOS comparison

A subset of about 50% of the mammalian rCNEs were used as query sequences against the corresponding fugu homologous sequences using CHAOS [24] and BLAST2 [16], using a gap penalty of 2 as was used in the CNE analysis and e-value set at infinity to ensure that no hits would be filtered because of their statistical significance, analyzing both strands. The analysis was conducted three times varying only the word length used between 20, 15, and 10. The hits thus obtained were filtered in order to take only those sharing an identity of at least 60% and a length of at least 40 bp.

### Overlap analysis

Overlaps among different classes of conserved noncoding regions were defined using their genomic coordinates after having mapped all elements on the mouse loci used in this analysis. Because there is no downloadable dataset for CNGs, they were obtained by querying the GALA database [57] for conserved regions shared between human an mouse of at least 100 bp and 70% identity. CNEs [15], UCEs [14], and known enhancers were downloaded from Genbank. Enhancers were downloaded by searching for enhancer features in mouse Genbank records and then checking them manually to eliminate mis-annotated entries. All the sequences thus downloaded were then mapped on the mouse loci used in our analysis by using Megablast [58] with default parameters for CNGs, UCEs and known enhancers, and with a gap penalty of 2 for mapping CNEs, in accordance with the parameters used by Woolfe and coworkers [15] in their analysis. Elements were considered mapped with 75% coverage and 75% percentage identity. Only elements that did not map to exons were taken into consideration.

### Identification of control fragments

A set of control fragments to be tested *in vivo *was built from the same gene loci in which the tested SCEs were found, by selecting regions that were not conserved and did not present repeats, of the same length and number as the elements tested.

### Zebrafish embryo injections

The enhancer activity was assayed in conjunction with the minimal promoter *mHSP68*, which was previously shown to have low activity in zebrafish embryos and which has allowed the detection of enhancer function from several heterologous gene elements [28,59]. HSP68lacZ-pBS DNA plasmids containing the mouse *HSP68 *promoter [59] and *lacZ *were prepared using the Promega PureYield Plasmid Midiprep System plasmid preparation kit, digested by Promega *BamH*I enzyme, and DNA fragments were gel purified using the Promega Wizard SV Gel and PCR Clean-Up System kit (Promega, Madison, WI, USA). *HSP*:*lacZ *DNA fragments were resuspended in 1% phenol red containing nuclease-free water at a concentration of 25 ng/μl, as described previously [60], and were injected into the cytoplasm of zebrafish embryos at one cell stage. Wild-type embryos (Tubingen AB) were collected after fertilization and dechorionated by pronase, as described previously [61]. Fugu DNA was used for production of SCE fragments. Fragments were amplified by polymerase chain reaction, then isolated and purified using the Qiagen Qiex DNA purification kit (Qiagen, Valencia, CA, USA), and finally eluted in sterile water. For injection, phenol red was added to yield a final concentration of 50 ng/μl. Coinjection of polymerase chain reaction fragments at a concentration of 50 ng/μl reaching a range of 5 to 1 molar ratio with the *HSP:lacZ *fragment. Embryos were maintained at 28°C and collected at prim 6 stage [62], fixed and *lacZ *stained as described previously [27].

### Analysis of transgene expression

*LacZ *stained embryos were analyzed by plotting the mosaic expression activity on expression maps, as described previously [63,64]. The co-injection experiments were repeated three times. Data from approximately 100-120 embryos were collected on a single expression map providing an expression profile. For each embryo expressing *lacZ *the number of expressing cells was counted and classified in muscle, notochord, CNS, eye, ear, and vessels. These tissues were selected because they are well defined at the time of inspection [27]. Other tissues that were either difficult to determine or might have represented abnormalities (ectopic tissue growth, apoptotic mismigrating cells) were counted as 'other'. Twenty-three SCEs, four SCEs overlapping known mouse enhancers, 12 control fragments, one negative control consisting only of the *HSP:lacZ *fragment, and the positive control *ArC *[65] were analyzed.

We verified the significance of the enhancement of expression over the general low level improvement of expression of co-injected fragments probably caused by carrier DNA effect (see, for example, [65]) in two ways. First, we aimed to detect tissue-restricted enhancers; second, we aimed to identify generic enhancers. To identify tissue-restricted enhancers we compared, for each fragment co-injected and for each tissue, the number of expressing cells with respect to the number of expressing cells from the embryos injected with the negative control in the respective tissues, only when the average of cells expressing *lacZ *in injected embryos was higher than in the control. Fisher exact tests were then used on the comparisons and a *P *value cut off of 0.01 was used to classify a fragment as a tissue-restricted enhancer. The identification of generic enhancers was performed by establishing the average and standard deviation of the number of expressing cells per expressing embryo in the control fragments and then classifying as enhancers fragments in which the number of expressing cells per embryo was higher than the average plus twice the standard deviation of the control fragments. In the calculation of the average and standard deviation we excluded the UBL7 control fragment because it was a clear outlier that exhibited activity that was higher than any of the enhancers tested, including the positive control. All fragments classified as enhancers by either of the two tests were considered positive.

## Additional data files

The following additional data are included with the online version of this article: A table showing GO analysis of genes associated with CNEs and genes associated with SCEs (Additional data file 1); a figure showing boxplots comparing the distribution of the distance of collinear versus noncollinear nongenic SCEs from the transcriptional unit (Additional data file 2); a table providing further information on all fragments tested (Additional data file 3); a document providing supplementary information about tested fragments containing SCEs (Additional data file 4); a figure showing a Venn diagram that illustrates the overlap analysis of four datasets (CNGs, UCEs, CNEs and SCEs; Additional data file 5); a figure showing the number and type of conserved elements identified by CHAOS and BLAST2 in our dataset as a function of the word size used; and a table providing GO analysis results for genes associated with collinear nongenic SCEs located 1,000 bp upstream of the TSS (Additional data file 7).

## Supplementary Material

Additional data file 1 A table showing GO analysis of genes associated with CNEs and genes associated with SCEs Click here for file

Additional data file 2 A figure showing boxplots comparing the distribution of the distance of collinear versus noncollinear nongenic SCEs from the transcriptional unit Click here for file

Additional data file 3 A table providing further information on all fragments tested Click here for file

Additional data file 4 A document providing supplementary information about tested fragments containing SCEs Click here for file

Additional data file 5 A figure showing a Venn diagram that illustrates the overlap analysis of four datasets (CNGs, UCEs, CNEs and SCEs) Click here for file

Additional data file 6 A figure showing the number and type of conserved elements identified by CHAOS and BLAST2 in our dataset as a function of the word size used Click here for file

Additional data file 7 A  table providing GO analysis results for genes associated with collinear nongenic SCEs located 1,000 bp upstream of the TSS Click here for file

## Figures and Tables

**Table 1:** Transcription potential, localization, and number of mammalian rCNEs

rCNE type^a^	Total^b^	Coding^c^	Noncoding^d^
Total^e^	364,358	82,714	281,644
Pre-gene^f^	120,001	23,832	96,169
Intronic^g^	158,722	29,002	129,720
Post-gene^h^	85,521	29,766	55,755

^a^Type of conserved non-coding sequence (rCNE). ^b^Total number of rCNEs, including genic and nongenic. ^c^Number of genic rCNEs: overlapping EMBL proteins, ESTs, GenScan predictions, and Ensembl genes. ^d^Number of nongenic rCNEs: not overlapping EMBL proteins, ESTs, GenScan, and Ensembl genes. ^e^Total number of rCNEs, including pre-gene, intronic and post-gene. ^f^Number of pre-gene rCNEs: rCNEs localized before the translation start of the reference gene. ^g^Number of intronic rCNEs: rCNEs localized within the introns of the reference gene. ^h^Number of post-gene rCNEs: rCNEs localized after the translation end of the reference gene. EST, expressed sequence tag; rCNE, regionally conserved non-coding element.

**Table 2:** Transcription potential, localization, and number of vertebrate

SCE type^a^	Total^b^	Coding^c^	Noncoding^d^
Total^e^	27,196	5,769	21,427
Pre-gene^f^	8,387	1,363	7,024
Intron^g^	11,657	1,838	9,819
Post-gene^h^	7,152	2,568	4,584

^a^Type of SCE. ^b^Total number of SCEs, including genic and nongenic. ^c^Number of genic SCEs: overlapping EMBL proteins, ESTs, GenScan predictions, and Ensembl genes. ^d^Number of nongenic SCEs: not overlapping EMBL proteins, ESTs, GenScan, and Ensembl genes. ^e^Total number of SCEs, including pre-gene, intronic, and post-gene. ^f^Number of pre-gene SCEs: SCEs localized before the translation start of the reference gene. ^g^Number of intronic SCEs: SCEs localized within the introns of the reference gene. ^h^Number of post-gene SCEs: SCEs localized after the translation end of the reference gene. EST, expressed sequence tag; SCE, shuffled conserved element.

**Table 3:** Analysis of X-Gal staining in zebrafish embryos co-injected with the *HSP *promoter and SCEs or control fragments

Gene	Trans dev	Name	SCE bp	SCE Class	ENH	Embryo	Cell	ce/emb	*P *value						
									
									Muscle	Notochord	CNS	Eye	Ear	Vessels	Other
*No*	NA	lacZ			Neg control	161	40	0.25							
*Shh*	Y	ArC			Pos control	96	242	**2.52**		**8.48E-07**					
*Shh*	Y	12058	45	Rev	Y	139	69	0.5	**6.86E-09**						
*Otx2*	Y	13988	51	Mov	Y	111	93	0.84	0.6444		**0.006269**	0.5536	0.3155		
*Gata3*	Y	15402	40	Mre	Y	107	103	**0.96**			0.398	0.5764	0.1906		1
*Ets*	Y	8744	40	Mov	Y	105	180	**1.57**			**0.002593**			**4.78E-09**	
*Ets*	Y	8745	46	Mov	Y	133	210	**1.58**			0.1558	0.6015	0.3619	**2.15E-06**	
*Ets*	Y	8726	41	Mre	Y	159	345	**2.17**			0.05534	0.6136	0.1485	**2.08E-06**	
*Ets*	Y	8728	48	Mre	Y	149	176	**1.18**			0.0444	0.129	0.07924	**1.31E-05**	
*Pax2b*	Y	31027	39	Col	Y	149	105	0.7			**0.002374**	0.06327	0.1902		
*Pax6a*	Y	15696	33	Mov	Y	133	122	**0.92**			**8.21E-06**	0.3343	0.01268		
*Pax3*	Y	24781	42	Mov	N	124	67	0.54	0.02982		0.5287	1			
*Zfpm2*	Y	23818	48	Col	Y	140	119	0.85			**1.49E-06**	0.01296	1		
*Zfpm2*	Y	23838	48	Mre	Y	131	148	**0.98**			**0.0003576**	0.04369	0.1231		
*Tmeff2*	N	26014	48	Mov	N	164	125	0.76			0.7654	0.02301	0.3371		0.2801
*Tmeff2*	N	26015	38	Mov	Y	120	159	**1.33**	**0.001035**		0.303	0.2088			
*Tmeff2*	N	26016	51	Mre	Y	109	148	**1.36**			**0.0006309**	0.0149	0.5862		
*Jag1b*	Y	16407	37	Col	N	136	98	0.72	1		0.1849	1	1		
*Jag1b*	Y	16408	55	Col	Y	142	109	0.86			**5.45E-08**	**0.006524**	0.3245		
*Jag1b*	Y	16409	44	Rev	N	106	54	0.51	1		0.5088	1	0.5058		
*Mapkap1*	N	17058	37	Mov	Y	143	295	**2.06**	0.6825		0.05292	0.3788	0.6065		1
*Mapkap1*	N	17059	39	Mov	Y	136	171	**1.26**	0.6686		**0.004037**	0.5973	0.077	0.5197	
*Mab21l2*	Y	23001	42	Col	Y	142	317	**2.23**			**1.24E-07**	**0.004985**	0.2339		
*Mab21l2*	Y	23002	37	Mre	Y	155	122	0.79			**7.85E-08**	**0.004138**			
*Hmx3*	Y	11669	150	Col	Y	165	136	0.82			**0.001029**	0.07062	0.01423		
*Lmx1b*	Y	17027	300	Col	Y	116	105	0.91			**0.00762**	0.1876	1		
*3110004L20Rik*	N	5803	45	Mre	N	65	16	0.25	0.2929						1
*3110004L20Rik*	N	5802	39	Mov	Y	122	320	**2.62**	0.1874	0.01209					
*Elmo1*	N	6026	45	Rev	Y	103	76	0.74	**0.007132**	0.6848					
*Ets*	Y	11216	NA	Ctrl	N	104	74	0.71	1						0.6954
*Gata3*	Y	3255	NA	Ctrl	N	174	110	0.63	0.04481		0.281	0.5739	0.02163		
*1300007F04Rik*	N	2797	NA	Ctrl	N	157	115	0.73							
*Tmeff2*	N	198	NA	Ctrl	N	145	23	0.16	0.7448		0.6597		0.3651		
*Mab21l2*	Y	909	NA	Ctrl	N	165	92	0.56	0.06359		1	1	1		
*3110004L20Rik*	N	410	NA	Ctrl	N	107	23	0.21							0.01984
*Elmo1*	N	10157	NA	Ctrl	N	146	38	0.26	0.287	0.8126					
*Shh*	Y	11271	NA	Ctrl	Y	165	83	0.5	**3.34E-07**		1	1	1		
*Impact*	Y	5990	NA	Ctrl	N	150	101	0.67	0.6496		0.2754		0.0622		
*Ubl7*	N	268	NA	Ctrl	Y	117	644	**5.5**	**0.0003325**		**7.15E-11**	0.02555	0.6197		
*Lmx1b*	Y	11767	NA	Ctrl	N	116	15	0.13	0.2743				0.0707		1
*Irx3*	Y	5945	NA	Ctrl	N	93	15	0.16	0.03938						

For each DNA fragment tested the following information is given, from left to right: the gene locus in which the DNA fragment is found; indication about the GO classification of the gene in the 'trans-dev' class (Y = yes, N = no); the identifier given to the SCE or control fragment; the size of the SCE; the class (rev = reversed, mov = moved, mre = moved and reversed, col = collinear, Ctrl = control); summary about the potentially enhancer function of the element (Y = yes, N = no); the number of embryos injected; the total number of cells X-gal-stained; the ratio of stained cells divided by the number of embryos observed (with bold highlighting those with significant generalized enhancer activity); the *P *values for the significance of the number of cells observed in the fragment tested versus the *lacZ:HSP *control for each tissue (bold for *P *values < 0.01; see Materials and methods). See Additional data file 3 for further info on the fragments tested. CNS, central nervous system; SCE, shuffled conserved element.
