# Supplementary material for: Shuffling of cis-regulatory elements is a pervasive feature of the vertebrate lineage
Source: Genome Biol. 2006 Jul 19;7(7):R56. doi: 10.1186/gb-2006-7-7-r56 (PMC1779573; doi:10.1186/gb-2006-7-7-r56)
Supplement: Additional data file 2 — A figure showing boxplots comparing the distribution of the distance of collinear versus noncollinear nongenic SCEs from the transcriptional unit [file gb-2006-7-7-r56-S2.pdf]

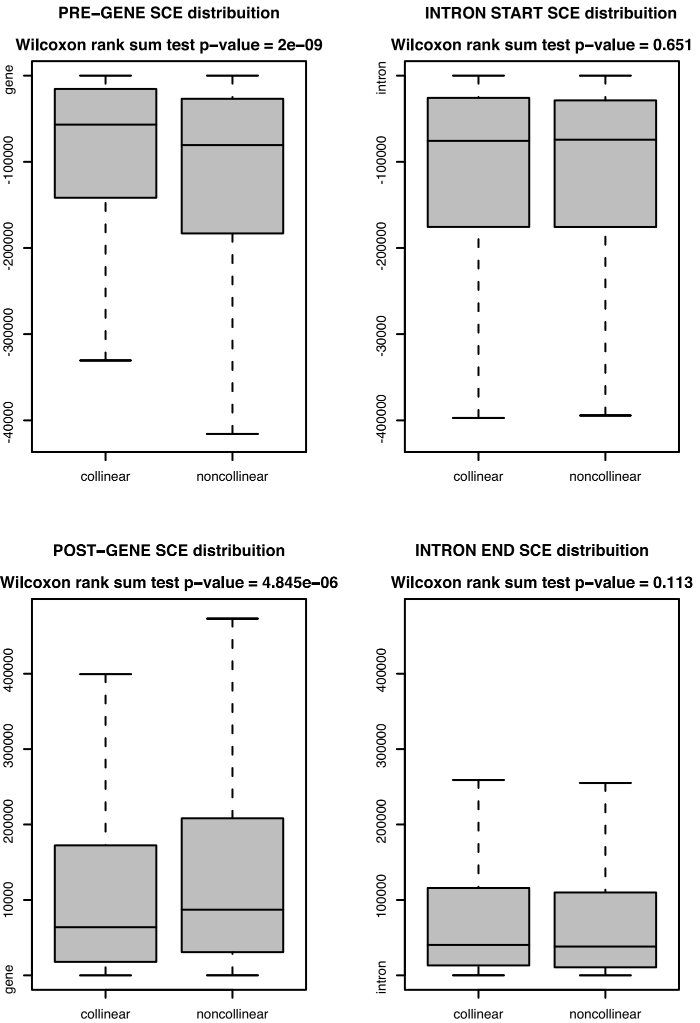
PRE−GENE SCE distribuition
Wilcoxon rank sum test p−value = 2e−09
gene
-100000
-200000
-300000
-40000
collinear
noncollinear
INTRON START SCE distribuition
Wilcoxon rank sum test p−value = 0.651
intron
-100000
-200000
-30000
-400000
collinear
noncollinear
POST−GENE SCE distribuition
Wilcoxon rank sum test p−value = 4.845e−06
400000
300000
200000
10000
gene
collinear
noncollinear
INTRON END SCE distribuition
Wilcoxon rank sum test p−value = 0.113
400000
300000
200000
100000
intron
collinear
noncollinear
