# Supplementary material for: Shuffling of cis-regulatory elements is a pervasive feature of the vertebrate lineage
Source: Genome Biol. 2006 Jul 19;7(7):R56. doi: 10.1186/gb-2006-7-7-r56 (PMC1779573; doi:10.1186/gb-2006-7-7-r56)
Supplement: Additional data file 4 — A document providing supplementary information about tested fragments containing SCEs [file gb-2006-7-7-r56-S4.pdf]

Tested Fragments

Regions within gene structure context, details of conserved regions,

sequences of conserved regions, and multiple alignments of conserved element.

Fragment Pax2_31027

Representation of conserved regions within their genic context

Within the mouse context is shown the rCNE from which the tested SCE is derived (red diamond)

Within the fugu context all CHAOS hits obtained from the mouse rCNE are shown (green diamonds, in red the one tested)

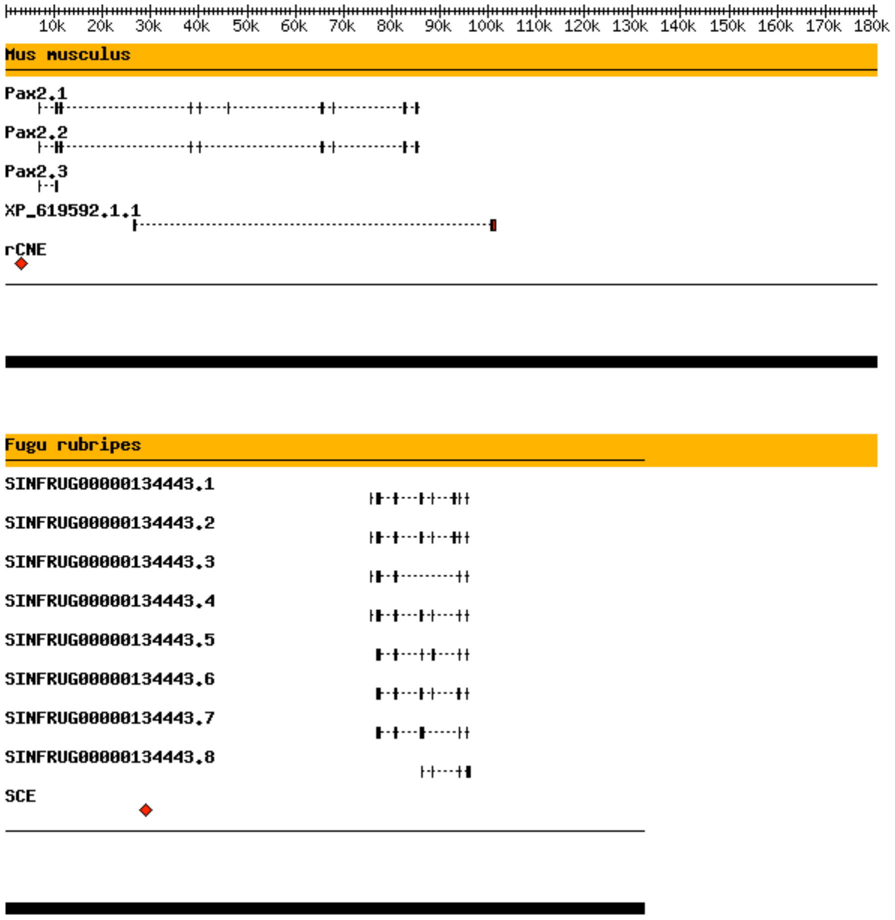

Conserved regions shown within 2000bp context

The mouse context contains:

-rCNEs green boxes, in red the one considered for this test

-SCEs yellow boxes, in red the one considered for this test

-CHAOS hits blue boxes, in red the one considered for this test

The fugu context contains:

-the fragment amplified in this test

-CHAOS hits blue boxes, in red the one considered for this test

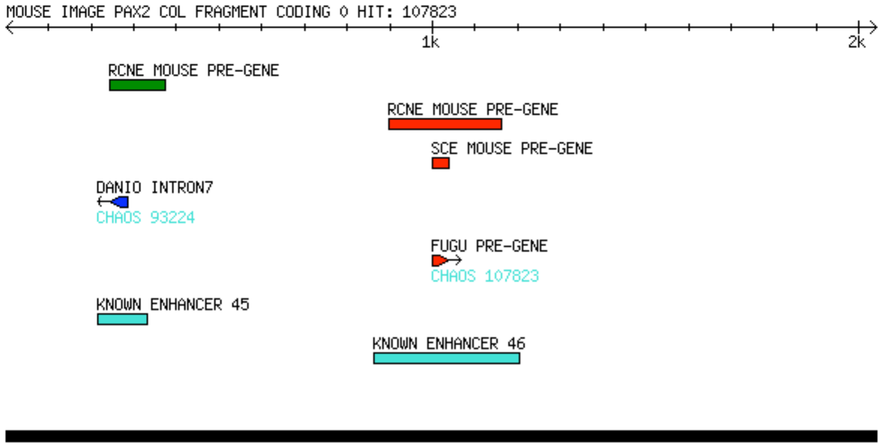

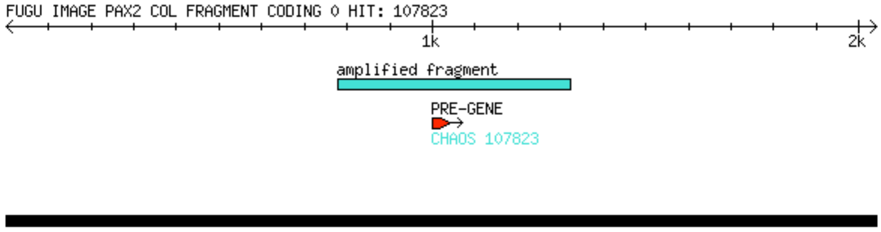

SEQUENCES

>fugu_fragment_for_primer_design_31027
AGTACGGCGACACTTCTGGAAACATATTGGGCCTTGTTATATTGAATGAAATGCTAATTC
TCACAGACTGTCTGGATTATGGCTCTTTGTGTGCACGCGTGGCCTCGGGGCCGTGCGTGC
ACGTAGTGCTTATGTCAAAAAACGATCTAAAGAATGTGAAAAAACAAATTTTTTATAAAT
GCAGCTCTACAATATCGGCTCTAGCTGTGTAGGAACATTAAAGGACATAAATGTAGCTTT
GGCTCAGGAGGAAAGGTTTTAAGAGAAACTGTTGCTCCTTGGACAGCCTGCAGATGTGGG
AGTCACTGAAAGCATCAATAAGTGTTTGAAGGGATGTTAAGCAGCAGGCCGGCCAGCATC
TGTTTTAACCCCCCCACCAACCCGACCCTTTCTGAACTTACCTTTCTTTGTGTGGGGACT
TGGACAAAAGCCATAGTTCCTTCTGGTCTGTCAAAGCTATTCTATAATCAACCAACCATG
GCCCTCCCTTCAAAGAATAGGAAGACATTATTCAGCTTCCTCCTTCTGATCTTGGGGCCA
CAACAAACCATGTAAGCCATGGCTGACTGGTTAAAGCAGGGGGTGGGGAGGGAAGAGGGG
CACAATGTCCTCCTGTCAGCAGCTGCAGATACAGAGGCCGACGAGCACCACGTGACCCTT
CGACCACTCCTTCAACCATGGTGGCCACACTGCTGAGCACTGCCCCGCAGATAGAAGCCT
TGAACTTACACACTGGCGGGGGTCAGAGGTCACAGCGGACGGACTGTAGTTAACTAACCA
ACCACGAGTGAAACTCGGACAAAACAGTTTGCCTCATGAAACAGTTCCACGTTGTGAGCG
TGCACTGCAAAATCCTGGAAAGGTGAAATGAGTCGGTAACAAGATGAAGATATCGAGGCC
GCCGCAACACGAAGCGCTGCACTTAAGCTCATTTCCACGACTGACTGAAATGTAGAGACT
TAAAACACTCACCCAGAACATTTACTGGCTGGACGGTTCCAGTTTCCCAAAAGTGCACAC
GCAGATTTAAATTTAAGGAGGAGCAGAAGTCTTCGCATTATTCATTAAATAAAGATGAAG
CCGGCGGAGGAGTGTTTGGTCATAGGAGATAACCGGACGGGGAAAACGTGGGCACGAAAC
GCGCACGCGTGTCGATCAGCAAAGGCACTTTTCATCCAGAAAATGAAAAGACAAAATGAT

```
CATTTGTCATTTTCTAAGCAAGAACAGTTTTAATGGGGCCACTTTAGAATCGGTTATTAC
TCCACTGAGTAACGTTGATTCTATAAGAGTAATGCCAATGATCACAGAGAGCCAGGCTGA
ATGTTAAATAGGTAAAGAGCCCTGTGGCAGAGGCATTGGCCCCCTGTTACCACTTAGCCC
TATTTGGCTGGCCTCACTCGCGCTCATAATTAAGACCCCCTACTGTGAATATAAATGGCC
CCTCAATCAATAGTGATAGAGAGAAATAAAGAGACGGAGACGTGGAGACCACGGGCATAT
CTCAGGGGCAAACATGATAGCATTATGAAAGGAGCAGCTTGAAAGCAGCTTTTACTCTGA
CAGGGGAGCAAACGTTGTAATTGTCCCTCGGGGGAGCAGAAGCGCTTGTTATTAGAAAAC
AATTTCTCCCTCTAGGCCACTTCTCCTCCAGCCTGAGGGCGTCACTGATCCTGCGTGTCA
CTTCTGCGCCCTTATCCACCACTTCCAAACACTTTTCATGTGTGACAGGAAGCCGCGGTG
CCTGTGGTTTCGCGTCACTCCAGATTAACCAGCGATTCCATGAATATTACTCGCAGTCAC
ATCAGCAAACTTTGTGCAACGCCAGAGACGTGGCGGCCATGTGCCACGCATACATCATTT
CAAATATTTAGCCAGTTCCCTGGCTAATTTCAAAGGTTTATTTCCTGAAATGAATCCCCT
TTGTCAAATCCATTGATCCCTGCAAACCTAAAGCAACATTACTGTTTGTGTAATAAATTG
ACCTGTAATGTAGATTAAGCTGGGTAGTGGTGGTGGTGTTGGGAGGGGTTTTGTACNNNN
```

```
>amplified_fugu_segment
AACCACGAGTGAAACTCGGACAAAACAGTTTGCCTCATGAAACAGTTCCACGTTGTGAGC
GTGCACTGCAAAATCCTGGAAAGGTGAAATGAGTCGGTAACAAGATGAAGATATCGAGGC
CGCCGCAACACGAAGCGCTGCACTTAAGCTCATTTCCACGACTGACTGAAATGTAGAGAC
TTAAAACACTCACCCAGAACATTTACTGGCTGGACGGTTCCAGTTTCCCAAAAGTGCACA
CGCAGATTTAAATTTAAGGAGGAGCAGAAGTCTTCGCATTATTCATTAAATAAAGATGAA
GCCGGCGGAGGAGTGTTTGGTCATAGGAGATAACCGGACGGGGAAAACGTGGGCACGAAA
CGCGCACGCGTGTCGATCAGCAAAGGCACTTTTCATCCAGAAAATGAAAAGACAAAATGA
TCATTTGTCATTTTCTAAGCAAGAACAGTTTTAATGGGGCCACTTTAGAATCGGTTATTA
CTCCACTGAGTAACGTTGATTCTATAAGAGTAATGCCAATGATCACAGAGAGCCAGGCTG
AATGT
```

```
>rCNE_mouse_sequence_130793
AGCAGAGAAGGCGAAGGAACTGAGATCCACAAGGAAGATTTATTGGGCAGATCAGATGCA
CAGAGGCGACTAATGAAGCAAATCCCGAGATGGGTATCAGAGCAACTCCCCAAAAGTTTA
TTTGCCTTTAAATTTCCGCAGGGAGGCGGGCTCCTTGTTTGAAGTGTAAATGCCCCTAGG
TTGTGGGGGGGTGTTGAAGGGCTGCTTTGAAAACACTAGAGAGAAAAGGTTCATTTAGAG
TCAGATGAGGGAAAAGCAACCAA
```

```
>SCE_mouse_sequence_130927
CAACTCCCCAAAAGTTTATTTGCCTTTAAATTTCCGCAGG
```

FUGU HITS IN THE CONTEST OF MAMMALIAN rCNE

CLUSTAL W (1.83) multiple sequence alignment

```
ENSCAFG00000009709      AGCGGAGACGGCGAAGGAACGGAGATCCACAAGGAAGATTTATTGGGCAGATCAGATGCA
ENSG00000075891         AGCCGAGAAGGCAAAGGAGCCGAGATCCACAAGGAAGATTTATTGGGCAGATCAGATGCA
ENSMUSG00000004231      AGCAGAGAAGGCGAAGGAACTGAGATCCACAAGGAAGATTTATTGGGCAGATCAGATGCA
ENSRNOG00000014253      AGCAGAGAAGGCGAAGGGACTGAGATCCACGAGGAAGATTTATTGGGCAGATCAGATGCA
SINFRUG00000134443      ------------------------------------------------------------


ENSCAFG00000009709      CAGAGGCGGCTAATGAAGCAAATCCCGAGATGGGTATCAGAGCAACTCCCCAAAAGTTTA
ENSG00000075891         CAGAGGCGGCTAATGAAGCAAATCCCGAGATGGGTATCAGAGCAACTCCCCAAAAGTTTA
ENSMUSG00000004231      CAGAGGCGACTAATGAAGCAAATCCCGAGATGGGTATCAGAGCAACTCCCCAAAAGTTTA
ENSRNOG00000014253      CAGAGGCGACTAATGGAGCAAATCCCGAGATGGGTATCAGAGCAACTCCCCAAAAGTTTA
SINFRUG00000134443      ------------------------------------------CAGTTTCCCAAAAGTGCA
                                                                  **  * *********  *

ENSCAFG00000009709      TTTGCC--TTTAAATTTCCGCAGGGAGGCGGGCGCCTTGTTTGAAGTGTAAATGCCCCTA
ENSG00000075891         TTTGCC--TTTAAATTTCCGCAGGGAGGCGGGCTCCTTGTTTGAAGTGTAAATGCCCCTA
ENSMUSG00000004231      TTTGCC--TTTAAATTTCCGCAGGGAGGCGGGCTCCTTGTTTGAAGTGTAAATGCCCCTA
ENSRNOG00000014253      TTTGCC--TTTAAATTTCCGCAGGGAGGCGGGCGCCTTGTTTGAAGTGTAAATGCCCCTA
SINFRUG00000134443      CACGCAGATTTAAATTTAAGGAGG------------------------------------
                           **   *********  * ***

ENSCAFG00000009709      GGTTG------GGGGGTG--GAAGGGCCGCTTTGAAAACACCAGAGAGAAAAGGTTCATT
ENSG00000075891         GGTTG------GGGGGTG--GAAGGGCCGCTTTGAAAACACCAGAGAGAAAAGGTTCATT
ENSMUSG00000004231      GGTTGTG---GGGGGGTGTTGAAGGGCTGCTTTGAAAACACTAGAGAGAAAAGGTTCATT
```

```
ENSRNOG00000014253      GGTTGGGANNNNNNNNTGTTGAAGGGCTGCTTTGAAAACACTAGAGAGAAAAGGTTCATT
SINFRUG00000134443      ------------------------------------------------------------


ENSCAFG00000009709      TAGAGGCAGACG--GGAAAAGCAACCAA
ENSG00000075891         TAGAGGCGGACG--GGAAAAGCAACCAA
ENSMUSG00000004231      TAGAGTCAGATGAGGGAAAAGCAACCAA
ENSRNOG00000014253      TAGAGTCAGATGAGGGAAAAGCAACCAA
SINFRUG00000134443      ----------------------------
```

Fragment Zfpm2_23818

Representation of conserved regions within their genic context

Within the mouse context is shown the rCNE from which the tested SCE is derived (red diamond)

Within the fugu context all CHAOS hits obtained from the mouse rCNE are shown (green diamonds, in red the one tested)

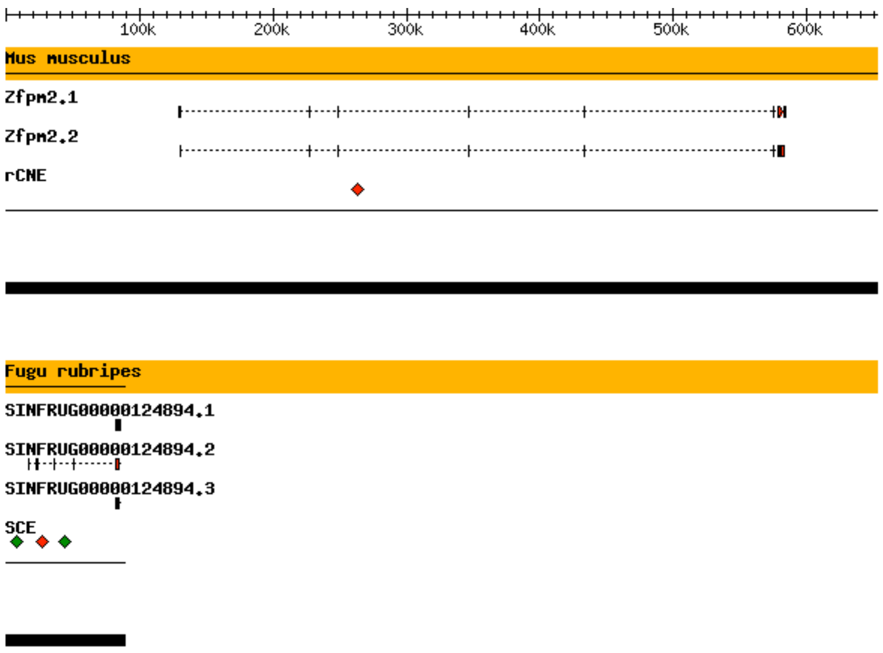

Conserved regions shown within 2000bp context

The mouse context contains:

-rCNEs green boxes, in red the one considered for this test

-SCEs yellow boxes, in red the one considered for this test

-CHAOS hits blue boxes, in red the one considered for this test

The fugu context contains:

-the fragment amplified in this test

-CHAOS hits blue boxes, in red the one considered for this test

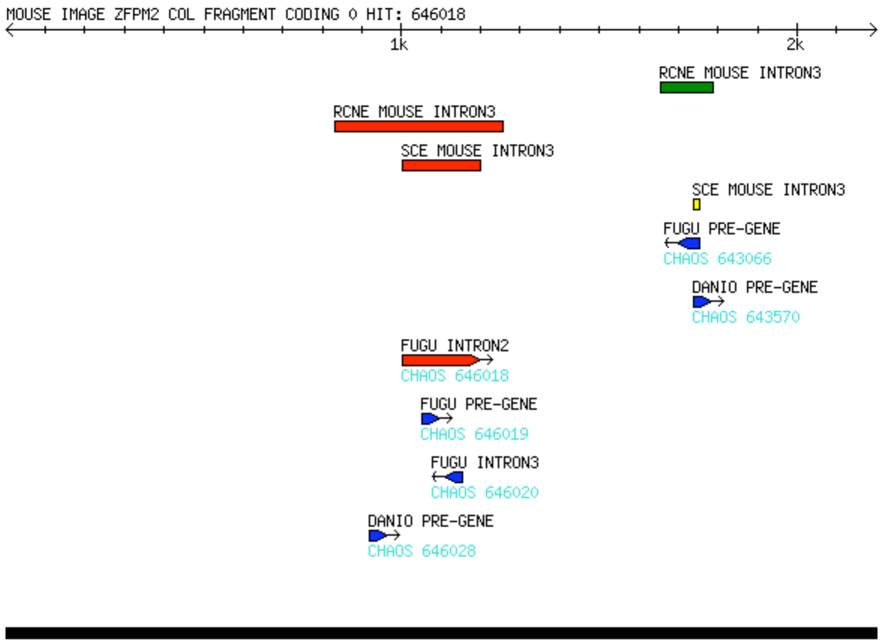

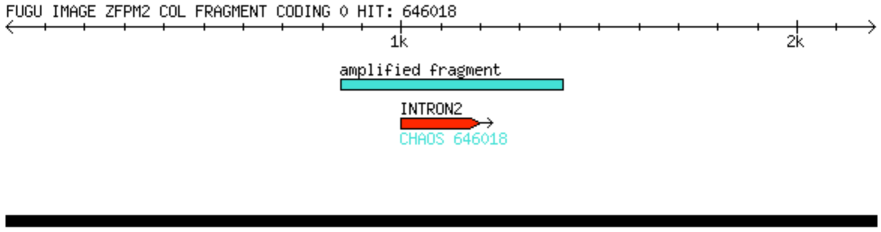

SEQUENCES

>fugu_fragment_for_primer_design_23818
GTTAAGTCACAAAGCTGTGACCGTATTAATGTTATAGCAAATGCCACACTACTTATTCGA
TGGTCCTGTTTTAGCGTGACGACCACGTGGCGGCTGCATGGTCGTCTCCCCCTCCTCGGC
CAGCCTGTGGCGCCGTGGGTGCCAGCTGGGCGTCGGCCAGAGAGAGGCGGGGGTCATCTG
TAGGAGCGGGCACTGAGAATCCCCCTAATGGCCAGACCCAGACAAACTAAGGGCAAAGGT
GCACCCCCCCACCCGCCCTCTCACCCGCGCCTGCCTTGAGAACAACATCCATTTTCATGT
ACCTCCTTCCTTCCTTCCGCTTTAAAAACATCAAACGTGCACATGCTGCTAACGCAGCTT
CGCTTAGATGCTNNNNNNNNNNNGCTGCAAGTGTGCGACCGATCTTACCGTCTCCAGAAA
AAGACACAATAGCCATTTCAGGTTTACCGCACATATGCCATGCTAGGCGATCTGGAAGAG
CAGATGGCCACGCTGGTGTGACAGGTGCCTCGACAAATGTTTGCGGTCGCCCGTGCTGCT
ATTTATAACACGGTGCAACACAGACATGCAAATATTTGCGAAAAAAGGACAGGGGGGCCC
TTTTTGTTCCGAGTCAAAGTGGTCACCGTCAGAAACAGATGGGATTAAACATTCTGGGCA
TCGTTTCATCAGACCTGAAGAACTTTATTGTTCTGTAGGAGAAACAGGAAGTGCTGGAAG
TGGTGAGGTGTAGCCACTCTTGTCCGTAAACAAGACTCTCCTCTTTGCATCTTTGGAATT
AATGTTCGCTCTTTCCGCATTGAGGCGGCGGAAGCATTTTTAGCCCTCAGCTGCAGCTTA
ACTGAGCATACAGTCGCCCAAACTCTGCTGCGCATACAGCAAAAAAAGTCCACTGGAGCA
GAGTTCAGTCAGGTCCTCCTCGGTAAACAAGGCATTTTCCAACTCAGCACCCGAGTCCAC
GATAAAGTCGGGGCGTCGTCAAGGAAGGAGGGGCGGGTGGAAATGAGCGCAAGAGACAAA
CGCAGAGCCCAGATGCAGATTAGGCTGCAGAAGATGAGGGTGTAAAAGGGATTTAATGGT
AGGATGGATTAGAGAAATCCTTGTAACAATGTTAAATAATTATGGTTCCAGCCGAGATCA
AAGCGCCACTGATTGAACCCCTCAAGCCTTCAGAACACGGAGTCTTGGAAAGCAAAGATT
GGTAAACTAGTGCTCAGACCACTCCGGCTGCTCCAGTTAAATTGGATTGGAAATATTAGG
ATACAGATGAATCCGTCAGGAGGCTTTCACAAAGCTTTTGTCACCGGAAATGTAGCTTGG
GAACGGTATGAAAGGTTTGATAGCGCACTGTCTCTGCAGAATCGTAAGACGAGGGTGTCA
CAGCTGTCTCCGCTGATACCTAAGCGTTTTAAGTAGACTTATAGACACACTTGACTGTTA

```
TATCAGCGGACCCAAAACTTTCAGCGCCGGCATTTATTCGAGGTTTAATTTTTTTCTTCT
TCTCTTCTCGTCTCCCAATCAAGAAATTTGGACGTGCATCACGGCGGTTCTCGCTGGGAA
AAAGGAAGGTTTTATCTTGCTCCTCCAGGGGTTTATTCCGAGTCCTCCGACGCGTCCCAC
AAAACATGAATGACACGGAGATGGCCGATACTGATGATGAAAGTCCGTCATCAGCTCCTC
GCCACGTTCCAGGCCCCCGAGTGATGTAGAATTAAATCGCAATCAAAGTGTTTTGTGTAA
ACACCGCGCGCCTGTTTTCGCCAAATGTATTTGTTCCTGAGGAGATTGCTGATTAAGGCC
TTTACGCCATCACCTGGATGCGCGAGCGTCCCCTGCCCGCCNNNNNNNNNNNNNNNNNNN
NNNNNNNNNNNNNNNNNNNNNNCCTTTTGCATTTCACAATGTGTGAATATAGTGATGAAA
TATGCAACAAGAGATTAGACAAATCACTGTACTGCTGCATCGCCTTGATGCAAATTTAGT
CACTGATCCGTTATACTAAGAGGCTTTATGCAGGAACTTCATGGTTGTTAGTTATATAAT
CTAAAATAGGGCAAAATGCAATCAGCGTTCTTATCATCTGAAGAAGATGTCATTACATTC
AGTTCTAGTTTTTTAGGGGGACGTAGCACATTGAAAACATTTCGAATAAGATAATTCATT
TCCAGCCATTTTATCCACATTTCAGCTGAATTTGTGT
```

```
>amplified_fugu_segment
GCATACAGTCGCCCAAACTCTGCTGCGCATACAGCAAAAAAAGTCCACTGGAGCAGAGTT
CAGTCAGGTCCTCCTCGGTAAACAAGGCATTTTCCAACTCAGCACCCGAGTCCACGATAA
AGTCGGGGCGTCGTCAAGGAAGGAGGGGCGGGTGGAAATGAGCGCAAGAGACAAACGCAG
AGCCCAGATGCAGATTAGGCTGCAGAAGATGAGGGTGTAAAAGGGATTTAATGGTAGGAT
GGATTAGAGAAATCCTTGTAACAATGTTAAATAATTATGGTTCCAGCCGAGATCAAAGCG
CCACTGATTGAACCCCTCAAGCCTTCAGAACACGGAGTCTTGGAAAGCAAAGATTGGTAA
ACTAGTGCTCAGACCACTCCGGCTGCTCCAGTTAAATTGGATTGGAAATATTAGGATACA
GATGAATCCGTCAGGAGGCTTTCACAAAGCTTTTGTCACCGGAAATGTAGCTTGGGAACG
GTATGAAAGGTTTGATAGCGCACTGTCTCTGCAGAATCGTAAGACGAGGGTGTCACAGCT
GTCTCCGCTGATACCTAAGCGTT
```

```
>rCNE_mouse_sequence_610907
GAATAGTGGATGATACTGGTGTAAGAGCTCAATAGAGCCCTCAAAGGTACAGCCAATGCA
TACAGCATTTTTGCAGATTTCAATTTGTAAGAAAATAATTTCATTAGAGTGGAAGAGAGA
AGAGAGAGAAACCACCAGTGAAGCCTTGGGAGAATGCAGGCTCCTGATGAGACCAGCAGA
GAAGACAGACTCAGCGTTCACATGCAGATTAGTCCTCAGAAGATGAGGGTGTAAAAAGGA
TTTAATGGTGGTGTGGATTATAGAAATCCATGTAACAATGTTAAATAATTATAGTTCCAG
CAGAGATCAAAGCAGCGCTGATTGAAGGCCCTCAAGCTGTTAACATGAAGTATCTTGAAG
GGGGAAAAACCCCCGCCTCAGAGATTGGTCAACCTGGGCAGGCATTGTCTATGATTTGTC
TCTGT
```

```
>SCE_mouse_sequence_611046
GAGACCAGCAGAGAAGACAGACTCAGCGTTCACATGCAGATTAGTCCTCAGAAGATGAGG
GTGTAAAAAGGATTTAATGGTGGTGTGGATTATAGAAATCCATGTAACAATGTTAAATAA
TTATAGTTCCAGCAGAGATCAAAGCAGCGCTGATTGAAGGCCCTCAAGCTGTTAACATGA
AGTATCTTGAAGGGGGAAAA
```

FUGU HITS IN THE CONTEST OF MAMMALIAN rCNE

```
CLUSTAL W (1.83) multiple sequence alignment


ENSRNOG00000004109      GAATAGTAGATGATACTGGTGTAAGAGCTCAATAG--AGCTCTCAGAAGTAGAGCAAATG
ENSMUSG00000022306      GAATAGTGGATGATACTGGTGTAAGAGCTCAATAG--AGCCCTCAAAGGTACAGCCAATG
ENSG00000169946         GAATGGTGGATGATACTGCTAT------TCAATAGGGAGCTCTTAAAAATAGAGCAAATG
ENSCAFG00000000678      GAATGGTGGATGATACTGGTAT------TCAATAGGGAGCTCTTAAAAATAGACCAAATG
SINFRUG00000124894      ------------------------------------------------------------

ENSRNOG00000004109      CACACCACATTTTG-GCAGATTTCAATTTGTAAGAAAATGATTTCATTAGAGTACAAGAG
ENSMUSG00000022306      CATACAGCATTTTT-GCAGATTTCAATTTGTAAGAAAATAATTTCATTAGAGTGGAAGAG
ENSG00000169946         CATAAGACATTTTTTACAGTTTTCAATTTGTAAGAAAATGATTTCATTAGAGGGGAAAAA
ENSCAFG00000000678      CAGAAGACATTTTT-GCCGTTTTATATTTGTAAGAAAATGATTTCATTAGAGGGGAAAAG
SINFRUG00000124894      ------------------------------------------------------------

ENSRNOG00000004109      AGAAGAGA----AACCACCAGTGAAGACTTGGAAGAATGCAGGCTCCTGATGAGACCAGC
ENSMUSG00000022306      AGAAGAGAGAGAAACCACCAGTGAAGCCTTGGGAGAATGCAGGCTCCTGATGAGACCAGC
ENSG00000169946         AGAGAGAGAGAAAACCATCAGTCAGGATGTGGGAGAATGCAGGCTCCTGATGAGACCAGC
ENSCAFG00000000678      AGAGAAA---AAAACCTTCATTGGGAATGTTGGAGAATGCAGGCTCCTAAGGAGACCAGC
SINFRUG00000124894      ---------------------------------------------------GAAATGAGC
```

```
                                                                                   ** *  ***

ENSRNOG00000004109      AGAGAAGACGGACTCAGTGTTCACATGCAGATTAGGCCTCAGAAGATGAGGGTGTAAAAA
ENSMUSG00000022306      AGAGAAGACAGACTCAGCGTTCACATGCAGATTAGTCCTCAGAAGATGAGGGTGTAAAAA
ENSG00000169946         GGAGAAGACAAACTAAGTGTATGGATGCAGATTAGGCCTCAGAAGATGAGGGTGTAAAAG
ENSCAFG00000000678      GGAGAAGACAAACTAAGTCCACAGATGCAGATTAGGCCTCAGAAGATGAGGGCGTAAAAG
SINFRUG00000124894      GCAAGAGACAAACGCAGAGCCCAGATGCAGATTAGGCTGCAGAAGATGAGGGTGTAAAAG
                          *  ****  **  **       *********** *  ************* ******

ENSRNOG00000004109      GGATTTAATGGTGGGGTGGATTATAGAAATCCATGTAACAATGTTAAATAATTATAGTTC
ENSMUSG00000022306      GGATTTAATGGTGGTGTGGATTATAGAAATCCATGTAACAATGTTAAATAATTATAGTTC
ENSG00000169946         GGATTTAATGGTGGGGTGGATTATAGAAATCCATGTAACAATGTTAAATAATTATAGTTC
ENSCAFG00000000678      GGATTTAATGGTGGGGTGGATTATAGAAATCCATGTAACAATGTTAAATAATTATAGTTC
SINFRUG00000124894      GGATTTAATGGTAGGATGGATTAGAGAAATCCTTGTAACAATGTTAAATAATTATGGTTC
                        ************ *  ******* ******** ********************** ****

ENSRNOG00000004109      CAGCAGAGATCAAAGCAGTGCTGATTGAAGGCCCTCAAGCTGTTAACATGAAGTATCTTG
ENSMUSG00000022306      CAGCAGAGATCAAAGCAGCGCTGATTGAAGGCCCTCAAGCTGTTAACATGAAGTATCTTG
ENSG00000169946         CAGCCGAGATCAAAGCAGTGCTGATTGAACGCCCTCAAGCTGTTAACATGAAGTATCTTG
ENSCAFG00000000678      CAGCCGAGATCAAAGCAGTGCTGATTGAACGCCCTCAAGCTGTTAGCACGAAGTATCTTG
SINFRUG00000124894      CAGCCGAGATCAAAGCGCCACTGATTGAAC-CCCTCAAGCCTTCAGAACACGGAGTCTTG
                        **** ***********    *********  *********  * *  *    *  *****

ENSRNOG00000004109      GAGGGG---AAAAAACCC--GCCTCAGAGGTTGGTCAACCTGAGCAGACAACGACTGTGA
ENSMUSG00000022306      AAGGGG---GAAAAACCCCCGCCTCAGAGATTGGTCAACCTGGGCAGGCATTGTCTATGA
ENSG00000169946         AAGGG-----AAAAAAACCTGCCTCAAAGATTGGTCAGTCTGGGCAAACAATGTCTGTGA
ENSCAFG00000000678      AAGGGGGGNNNNNNNNNCCTGCCTCAGAGATTGGTCATCCTGAGTAAACTGTATCTGTGA
SINFRUG00000124894      GAAAGC----AAAGA---------------------------------------------
                         *  *

ENSRNOG00000004109      TCTGTCCAAGT
ENSMUSG00000022306      TTTGTCTCTGT
ENSG00000169946         TCTGTAGAAGT
ENSCAFG00000000678      TCTATAGAAGT
SINFRUG00000124894      -----------
```

Fragment Zfpm2_23838

Representation of conserved regions within their genic context

Within the mouse context is shown the rCNE from which the tested SCE is derived (red diamond)

Within the fugu context all CHAOS hits obtained from the mouse rCNE are shown (green diamonds, in red the one tested)

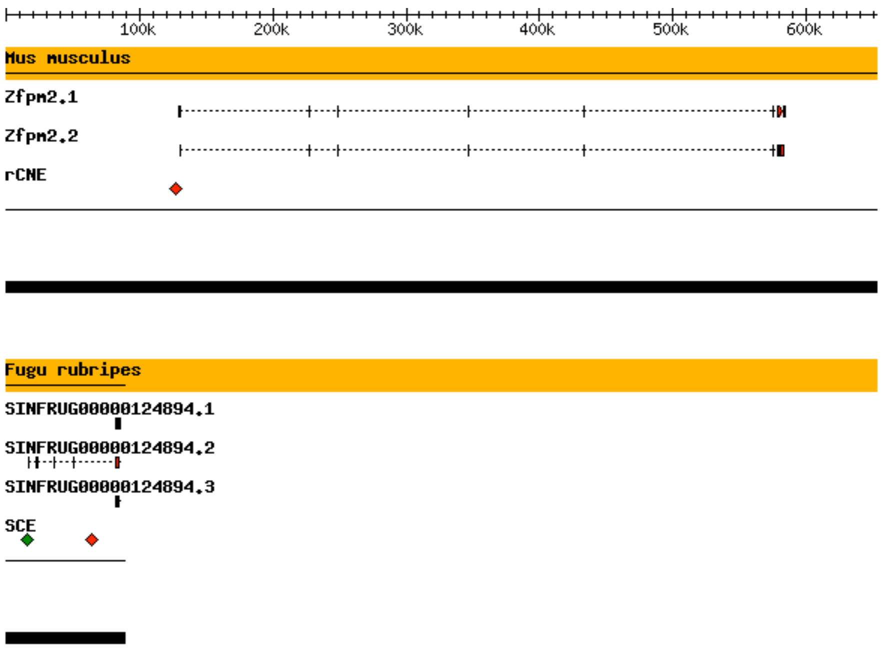

Conserved regions shown within 2000bp context

The mouse context contains:

-rCNEs green boxes, in red the one considered for this test

-SCEs yellow boxes, in red the one considered for this test

-CHAOS hits blue boxes, in red the one considered for this test

The fugu context contains:

-the fragment amplified in this test

-CHAOS hits blue boxes, in red the one considered for this test

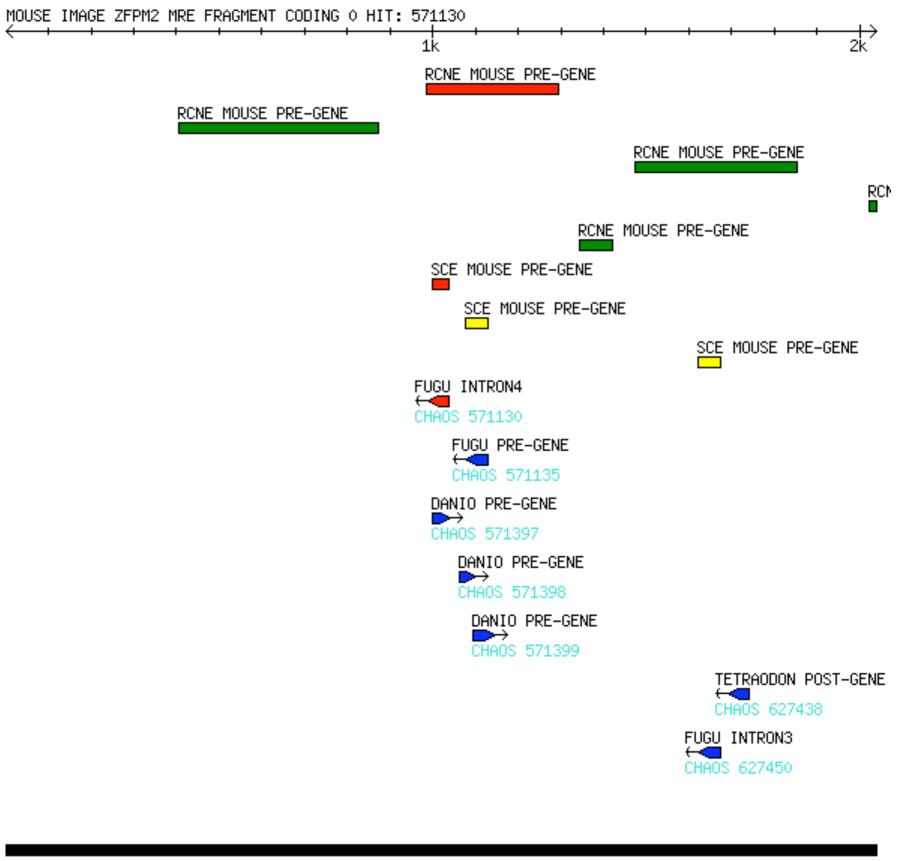

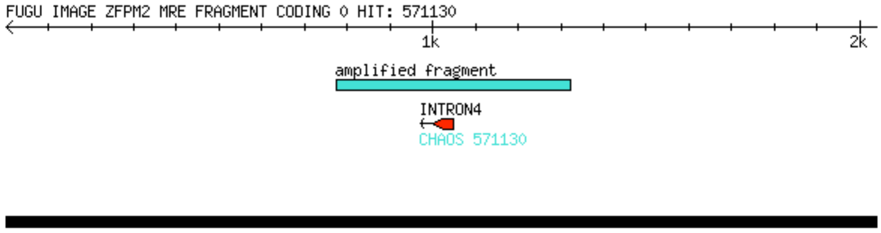

SEQUENCES

```
>fugu_fragment_for_primer_design_23838
CCAGATACTGCACTTGCAGCATGGACCTGGATGAAGCTGAGCCAACGGANNNNNNNNNNN
NNNNNNNNNNNNNNNNNNNNNNTCTTGTAGGAAAACTACAAAGGCGTAGGCGACATCTTG
GAGAACACCCCCGTCTCCTCCTCCTCAACCCCAGCTGGACTGTCACATTACCACGCGTGA
ATAAAGGGCATATTTTGACCTGCGGCGGGGAGCTCCCCCCTTTCGGCCCAGACAGACTGC
ACGGGGGGGTGTTAATCTATAGATCCGGCGGGGCCCCAGAATGATCAAAAATCAGCAAAT
GCCACAAAGGTTAACATCTGCGCCTGAAAGCACACATCCGTCTCCCCTATACAAAGACAA
GAGCGCGCTTCAAGGCGACTCTGCAGCGTTTGTTAGGAGCTAATTGGGTGACGGAGACCT
TGATGTTGGCGTGCAGAACCTCTGACTTTAGGCACGTCGGTAAATAAATAAACTGTGAAG
TTTGTTTTAGTTTCCCGAGGAGCCAAAAACGAAACCTTTTCTTTGTTTTTGCGCTGGGCT
CCTTTTCCAGCAACGCGAGGCCAAAGCCCCCCAAGACTGTTCAGGAAAAGCTCCTAAATG
GCGCCTCTGAAGCTCGGTTTGAGCTTTGTCATCACGCTTGAAGAATTTCCTTTTAATGTA
CTGTAATGTGAAATTCAGTGGGGACAAGTTTAATTGAGTTTTACACAAACCAGTTGGGAG
TAGCGCTGGAAGGTAGGTGATGTTGAGGTTTTTAATCTCTGGCTCCGGAGAGGCTTGTTG
ACGGAGTGGTTCTTTCTCTTTGTGGAGAAAAAAAGAGGAAGCCTAAGAAATGATACATCC
GTGGATAAAACCAATTGGCAACCCTCAAAGGGACGGGGGGTGATAAGGCAAATCCAGCAC
TTGTAGAGAAATAAAAGAATCCAACTGTTCATTAAAACAGGAAACAAAACAGGCGATTGA
GAAGGAAGGCAGACTGCTCAGTGGAGGAGTGAGTGAACCAGACAAACAGAGCCAGAGATA
TCAGAGGAAAGGATTGGAAAATAATCAAGGATTTGTGAGGAGAATTAATAAGGAAGATAG
GCACAAATAAGCATTTTTTCCCACTCTGGGCAAAATTAGGAGGGATGGACGACAAATATC
```

AAACGGGGGGAATGGCATAAGCTACATAGACACCTAATTATAAAGGATAACCCAGATATG
AAGGTCTGTGCCTTTTCATTTTACACACTTTGACTGTGAGGCGGTGGGGTCTTTTGTGGT
TGGTAAACTAGATATTCACTGGATAGGGAAGATTCAGACGCTCTCACAAAGCAGCGTTCT
GTCAGTCTCTGCCAGCAGAAATGATTTTAAACTTCACATTAAAGCCTTTTGGCAAGTGCT
GGAATGGCTCTTGTTAAGGAAGATTTCAGATAAAATGTGAGATTCAAGGAGCAAAATTGG
TTTGTGCTAAATCTCACTGAAAAATCGGAGTCTTTTTCTGAAGATGACCGGGAGTTTGCT
TTCGCCGGACTGTTTTAGAGGATANNNNNNNNNNNNNNNNNNNNNNNNNNNNNNNNNNNTTG
ACCCTGTCACGTGATGCAGCTCGGGGTAATGAGGACTGGATGTGGAGACTTGTGGTAATC
AAAGACTGGAGAGGAGAGGTTGTAGGAAAGATGGGGGTGAAATAAAGCAGCAATGGAATC
ATCAGCACGTGGACGATCGGCCACTGAGACCTTCCATAGGTGTTTTGTGTGAAAGGGAGC
CATTCATCAAGACATCAATAAAGCAGCATGGAGCAGATTATTGTTGTGTTTGCGTACCGC
CGGCCCGCCATCATAAATAATGAGTTTGCTGCTGCTGTATTAATTATAATTAGAATACTG
ATAATGTGTATGCGCTTTTATTACTGGGGCTGAATCAATTTTTACAGTCTTATTGAGGCT
CACAAGGTCACGCAAAGTTGGCAGAGTGAGAGATGGCGTTTTGTTGAGCATCTCTCTGCA
GTCGGGGCTGTGGGGGGTGGGGGTGGTCACAGAACTGGCCGCTTGTTCTCTGCGGATGTA
TCTCTATAA

>amplified_fugu_segment
GCTTGTTGACGGAGTGGTTCTTTCTCTTTGTGGAGAAAAAAAGAGGAAGCCTAAGAAATG
ATACATCCGTGGATAAAACCAATTGGCAACCCTCAAAGGGACGGGGGGTGATAAGGCAAA
TCCAGCACTTGTAGAGAAATAAAAGAATCCAACTGTTCATTAAAACAGGAAACAAAACAG
GCGATTGAGAAGGAAGGCAGACTGCTCAGTGGAGGAGTGAGTGAACCAGACAAACAGAGC
CAGAGATATCAGAGGAAAGGATTGGAAAATAATCAAGGATTTGTGAGGAGAATTAATAAG
GAAGATAGGCACAAATAAGCATTTTTTCCCACTCTGGGCAAAATTAGGAGGGATGGACGA
CAAATATCAAACGGGGGGAATGGCATAAGCTACATAGACACCTAATTATAAAGGATAACC
CAGATATGAAGGTCTGTGCCTTTTCATTTTACACACTTTGACTGTGAGGCGGTGGGGTCT
TTTGTGGTTGGTAAACTAGATATTCACTGGATAGGGAAGATTCAGACGCTCTCACAAAGC
AGCGTTCTGT

>rCNE_mouse_sequence_610690
TACTCTGGTTATTTTATCAACCTTTCCTTAAATATTCTGACTTTTATTTCTTTCAGCTTT
TAAACTGAATATTGAGGTCTGATTTATTGGTATCTCTTTTGCTTTGCATATTTAACCATT
TCAGATGCCAAAAGAAACACTAAGTGCTGTATTATTTTAATTCCAGGGGACATTCCTTTT
GGGTGATGTAAGTCAGCCAATTCTATGAAAGATCTATGAAAACAGTAACGTACAATAAAT
CCAATTCTAAGATTCTGTGCATGCCAATTCAACTATAATGATTTGAGAATGAGGGGGCTA
AAAAGCT

>SCE_mouse_sequence_611063
TATCAACCTTTCCTTAAATATTCTGACTTTTATTTCTTT

FUGU HITS IN THE CONTEST OF MAMMALIAN rCNE

CLUSTAL W (1.83) multiple sequence alignment

```
ENSG00000169946         TAATGCATTCTGGTTATCAATCTTGCCTTGAATTCTCTGACTTCTGCTCCTTTTAGCTTT
ENSCAFG00000000678      CAGTCTGGTTATTTTATCAATCTTGC-TTGAATTCCCTGACTTTTGCTCCTTTCAGCTTT
ENSMUSG00000022306      TACTCTGGTTATTTTATCAACCTTTCCTTAAATATTCTGACTTTTATTTCTTTCAGCTTT
SINFRUG00000124894      --CCTTGATTATTTTCCAATCCTTTCCTCTGATATCTCTGGCTCTGTTTGTCT-------
                                *    **   *  *** * *   **         * *  *  * *

ENSG00000169946         TAAACTAAACATTTAGCTGAGATTTATTTGTTTTTCTTTTGCATTGCATATTTAACCACT
ENSCAFG00000000678      TAAACTGAATATTTAGCTGAGATTTATTTGTTTCTCTTTTGCTTTGCATATTTAACCATT
ENSMUSG00000022306      TAAACTGAATATTGAGGTCTGATTTATTGGTATCTCTTTTGCTTTGCATATTTAACCATT
SINFRUG00000124894      ------------------------------------------------------------

ENSG00000169946         TCAAACTCCAAAAGAAACA------GTTACGATATTTTAATTCCAATGGGCATTCCTTTT
ENSCAFG00000000678      TCAAATGCCAAAAGAAACACTAACTGTTATGATATTTTAATTCCAATGGGCATTCCTTTT
ENSMUSG00000022306      TCAGATGCCAAAAGAAACACTAAGTGCTGTATTATTTTAATTCCAGGGGACATTCCTTTT
SINFRUG00000124894      ------------------------------------------------------------

ENSG00000169946         GGGTGACATAAAAATTCAGCCAGTTCTGTAAAAAGATCTTTAAAAACCATAATGTACAAT
ENSCAFG00000000678      GGCTGATATAAAAAGTCAGCCAGTTCTGTAAAAAGAGCTTTAAAAACCGTAATGTATAAT
ENSMUSG00000022306      GGGTGATGTA---AGTCAGCCAATTCTATGAAA-GATCTATGAAAACAGTAACGTACAAT
```

```
SINFRUG00000124894      ------------------------------------------------------------

ENSG00000169946         ACATCTGATTCTAAG--------CACACCAATTTAACTGTAATGAATTGAAAATGAAGGA
ENSCAFG00000000678      ACATCCAATTCTGAG--------CACACCAATTTAATTGTAATGAATTGAAAATTAAGGA
ENSMUSG00000022306      AAATCCAATTCTAAGATTCTGTGCATGCCAATTCAACTATAATGATTTGAGAATGAGGGG
SINFRUG00000124894      ------------------------------------------------------------

ENSG00000169946         GGTAAAAATCT
ENSCAFG00000000678      GGCAAAAATCT
ENSMUSG00000022306      GCTAAAAAGCT
SINFRUG00000124894      -----------
```

Fragment Tmeff2_26014

Representation of conserved regions within their genic context

Within the mouse context is shown the rCNE from which the tested SCE is derived (red diamond)

Within the fugu context all CHAOS hits obtained from the mouse rCNE are shown (green diamonds, in red the one tested)

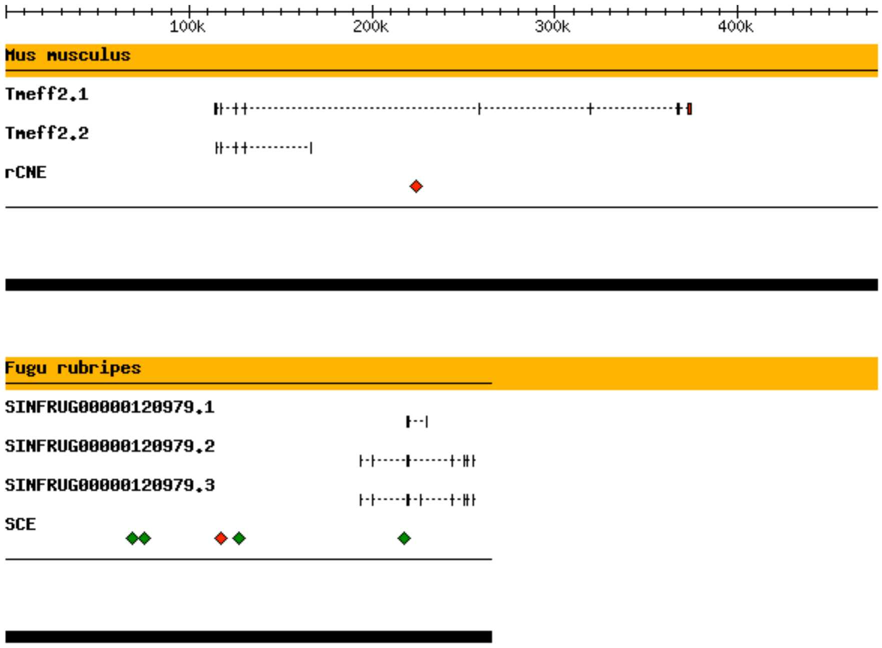

Conserved regions shown within 2000bp context

The mouse context contains:

-rCNEs green boxes, in red the one considered for this test

-SCEs yellow boxes, in red the one considered for this test

-CHAOS hits blue boxes, in red the one considered for this test

The fugu context contains:

-the fragment amplified in this test

-CHAOS hits blue boxes, in red the one considered for this test

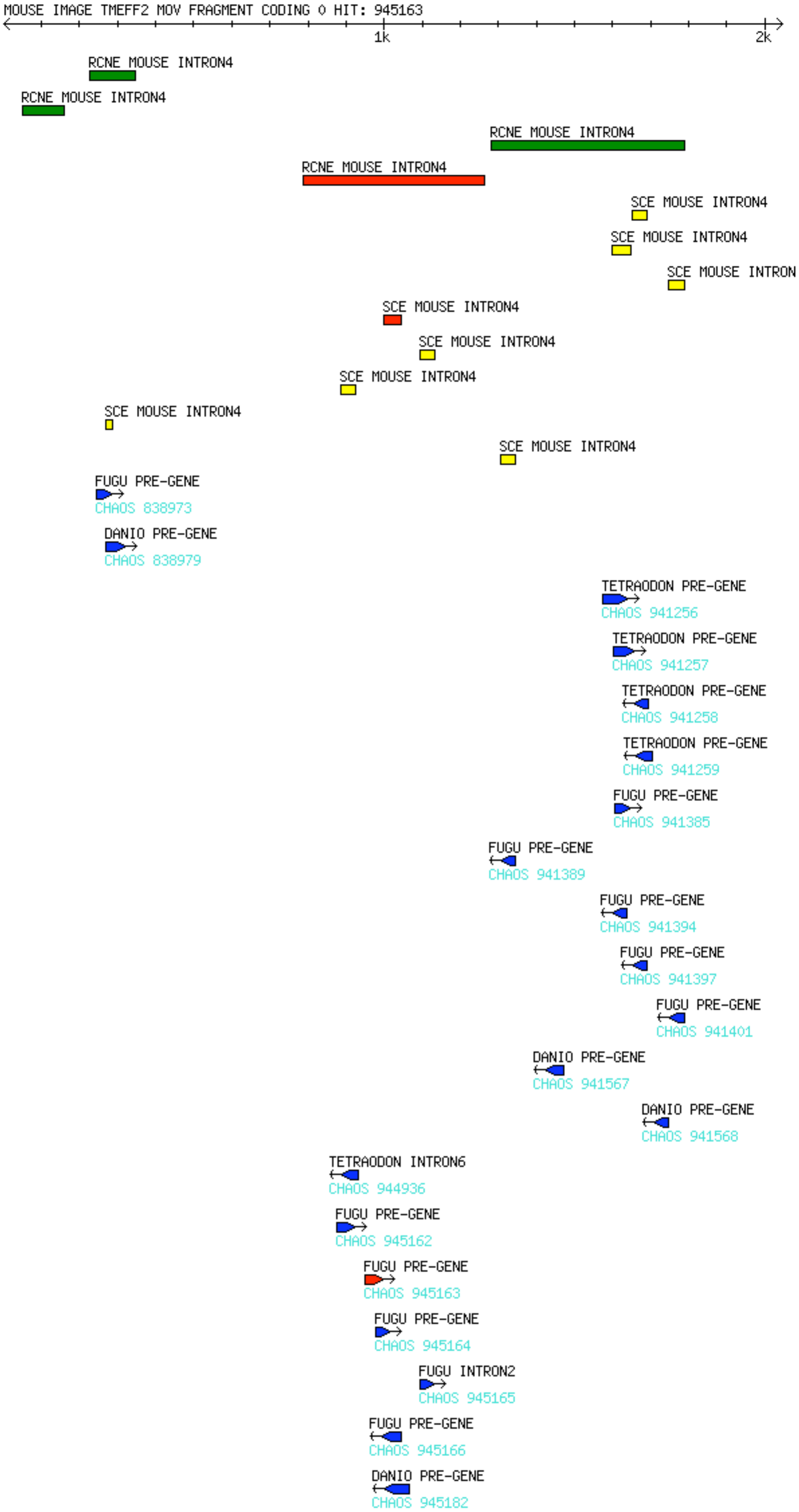
MOUSE IMAGE TMEFF2 MOV FRAGMENT CODING 0 HIT: 945163
1k
2k
RCNE MOUSE INTRON4
RCNE MOUSE INTRON4
RCNE MOUSE INTRON4
RCNE MOUSE INTRON4
SCE MOUSE INTRON4
SCE MOUSE INTRON4
SCE MOUSE INTRON
SCE MOUSE INTRON4
SCE MOUSE INTRON4
SCE MOUSE INTRON4
SCE MOUSE INTRON4
SCE MOUSE INTRON4
FUGU PRE-GENE
CHAOS 838973
DANIO PRE-GENE
CHAOS 838979
TETRAODON PRE-GENE
CHAOS 941256
TETRAODON PRE-GENE
CHAOS 941257
TETRAODON PRE-GENE
CHAOS 941258
TETRAODON PRE-GENE
CHAOS 941259
FUGU PRE-GENE
CHAOS 941385
FUGU PRE-GENE
CHAOS 941389
FUGU PRE-GENE
CHAOS 941394
FUGU PRE-GENE
CHAOS 941397
FUGU PRE-GENE
CHAOS 941401
DANIO PRE-GENE
CHAOS 941567
DANIO PRE-GENE
CHAOS 941568
TETRAODON INTRON6
CHAOS 944936
FUGU PRE-GENE
CHAOS 945162
FUGU PRE-GENE
CHAOS 945163
FUGU PRE-GENE
CHAOS 945164
FUGU INTRON2
CHAOS 945165
FUGU PRE-GENE
CHAOS 945166
DANIO PRE-GENE
CHAOS 945182

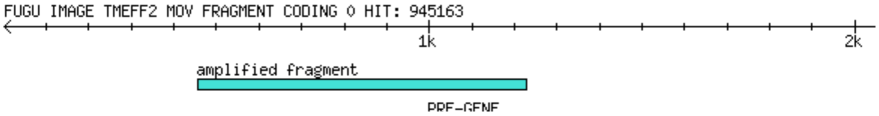
FUGU IMAGE TMEFF2 MOV FRAGMENT CODING 0 HIT: 945163
1k
2k
amplified fragment

SEQUENCES

```
>fugu_fragment_for_primer_design_26014
GACAATAAACTTGAAGCTGGGCCTTTTATATTAGCATTTATCTTTAGTTAACAAATATGG
AAACTATGAGTCACCGCGTAACCTCTGTAAAAGTTTTACAACTTTTACTATTAATAAAAC
ATGGAGCGTACACAGGACTCAGACTTCCTCTCATTTGAAATATGCAGCAATTATACTAAT
TAGGACAAGGCGTGAGGAAATTGCTTCTGGTTCTGGTTCTAAACATCAGACTTGGAGAAA
AATGTCGTCTACGGCACGATGCAAATATTCATCAATTATCTTTCAAGCTTACGACGCGTA
AAAAGCGTTTGATTTTGAGTTTCTGTGTGTTTGACTACAGAGATGAGCAGCTCAACGGGA
CGTCCAAGAGTTTTCATATAGACGGGACGGATAGAGAAGTCTATGCTGCCAAATCCTCCA
CCACTGAAAACAAATCAATGAATAAATCATAAAATAGCCTGATGGCCATTAACAATTCAT
TTGATCATTCATTCTGTGTGGAATTTTGGCCACCGCCATTTGTGTTCCAACACGGTTTCA
CTGATAATGGCCAGATCATATANNNNNNNNNNNNNNNNNNNNNNNNNNNNNNNNNNACTG
GAGAGGCAGAAGGGGCCGTAAAAATTAATATAAAACACAGGTTCTGCTTGGCGAGGCACC
CAAGCTTCAACTCTCAAAAGACAACAAGTTAACAGATGTTGTGCATCATGGGAGAACCTC
CCCTCCCCAACCGCCCACCCCCCTGCGCCAGTGATGGTAGGCTCCCCCTGGCATTATTTT
TTATTGTCATCTCTGAAACACCACAAACACTGTCCGTGCAACTCATTTCTTCCCCAAAAC
TCATTCCTGCCATCTGTTTTTTTCCGAGCAGGTCGTGCACTCTGCGAGGGAGAAAATATC
GAGAAAAGGAGGTNNNNNNNNNNNACAAAGTCATGCTTGACAGGCTCGGTGCTGCACTCC
CAGGCCTCGGGATGCGTTGAGAAGGAGCTGGCATTTCATGGTAGTTGACAACAACAGAGG
AACATCCCAGCAGCTTTAAGTGACAGCTTGCCGGTGCCAGTTTTCATTTGCTAACAAGCA
CATCAGCTCCCCATTAAGCTCCTTGTTGGAGACGGTGTGAGGTCTGCAGTGAGGGAATTC
AAGGCAGACTCTCACCAAAATGACATCAGATAAGGTGATGCGGCCAGCCGAACCGTGACC
ACATTGGGAGCGAATGTAAGTTTCCATTAATAGCTAGAGACAGTCAACTATCTCCTCAGA
CGCTACTCAGTATTCAGGAGCGCAACCACTCGGCAATGCATTAAAAAATGTTCTGCACAG
ATAAACAAACACTGTGTCAGGGACTTTTTTTAAAGACGACAGCTGCAAATACTCCAATTT
AAACCCATCAATAAAGATGAATACCGTTTTAAATTATTTCTCTATCTATGTGCGCTGCAG
ATGGTCTCATAGTTTATGTATGAAGCTGAAAACACCTTCAGATTTAAAGGCTTTTAGATC
TTCCCCAGCCAGAACAACAAAGACACGTTTGTGACTGTAGGGTTGCTGTGAAATACCCCA
GCAGGGCAGAGCATCCAAACTCTTTGGCGCCACTGTTTTCCTCTCTTCATATATCTGCTC
TGTCTTAGCTCTTCTGACTGTGATTGCCGCCTTTGCCCGTATTTTTCCCTCTTCTTTCAG
GCCGGCGCTCCACTTTTTATTCCAAATCTTGCGCGCCTTCCCAAAGGTCTTATCTGTTCC
CCACCAGCACTGCCAAACACCCTCTCAATCATGTATGCCTCAGCCTCTGCCTCCCAAGCC
CCCACCAGTGTCATTCATTTCGGTTTCAGGCATCAGTCTTTCCAGCTCTGCCGTTCTAGC
CAGCTAGCAGCTATAGCTGTCAGGACCCCGGAGCCGTCTCCGCCTGATTACGCTATCATA
TTGCCCCGCCAATGCAGTTATCTCCCGTTTGGACGGAGAGGAATCCAGTTTCATATTGGC
CATTAATGTTTAACGAGACAGAGGTTTAGAGNNNNNNNNNNNNNNNNNNNNNNNNNNNGG
GGGGCACTG
```

```
>amplified_fugu_segment
TAGCCTGATGGCCATTAACAATTCATTTGATCATTCATTCTGTGTGGAATTTTGGCCACC
GCCATTTGTGTTCCAACACGGTTTCACTGATAATGGCCAGATCATATANNNNNNNNNNNN
NNNNNNNNNNNNNNNNNNNNNNACTGGAGAGGCAGAAGGGGCCGTAAAAATTAATATAAA
ACACAGGTTCTGCTTGGCGAGGCACCCAAGCTTCAACTCTCAAAAGACAACAAGTTAACA
GATGTTGTGCATCATGGGAGAACCTCCCCTCCCCAACCGCCCACCCCCCTGCGCCAGTGA
TGGTAGGCTCCCCCTGGCATTATTTTTTATTGTCATCTCTGAAACACCACAAACACTGTC
CGTGCAACTCATTTCTTCCCCAAAACTCATTCCTGCCATCTGTTTTTTTCCGAGCAGGTC
GTGCACTCTGCGAGGGAGAAAATATCGAGAAAAGGAGGTNNNNNNNNNNNACAAAGTCAT
GCTTGACAGGCTCGGTGCTGCACTCCCAGGCCTCGGGATGCGTTGAGAAGGAGCTGGCAT
TTCATGGTAGTTGACAACAACAGAGGAACATCCCAGCAGCTTTAAGTGACAGCTTGCCGG
TGCCAGTTTTCATTTGCTAACAAGCACATCAGCTCCCCATTAAGCTCCTTGTTGGAGACG
GTGTGAGGTCTGCAGTGAGGGAATTCAAGGCAGACTCTCACCAAAATGACATCAGATAAG
GTGATGCGGCCAGCCGAACCGTGACCACATTGGGAGCGAATGTAAGTTTCCA
```

```
>rCNE_mouse_sequence_837802
GTCATTCCGGAATAGGTGTTTGATAGAAGCCATTAAACAGCCATCAAGGCCTTAAACAGC
TATACATGAGGGAATCTGCCACAAGTGCTACCATGATTTGGCTGCTGAGAGCCCTTAAGC
CTTGCAGCAGGTGTGATACTACTTAGAGTCAGTCTTGGGAGCAGGGAGTTGACAACAATG
GCCAAAGACTGGAGCAGAAAGAACAGGCAGCTTGAGTGTGAAAGACAGAAGCTGCCTTCC
CCATCTCCCACCTGTAATATTGCCAACCCATTAACAGAGCTTTGGAGTTCACGGTGTGCT
GAGTGACAGGAGCAGAACCATGAAGGTGGTAATGGCTTGCCAGGTTTAAATGTAGATGAA
TAACCTCTTGTCAGGGCAAAGAAGTCGTTTTTATCCTATAAAAATTCTGTGCATAACTAA
TGAGACACCTGGGCCATCATACCTGTTATAGTCAATTTCCAGTTTAGCTGTGTATCTA
```

```
>SCE_mouse_sequence_837950
GAGTGTGAAAGACAGAAGCTGCCTTCCCCATCTCCCACCTGTAATA
```

FUGU HITS IN THE CONTEST OF MAMMALIAN rCNE

CLUSTAL W (1.83) multiple sequence alignment

```
ENSG00000144339           GTCATTCACGAATAGGTGTTTGAGAGGAGCCATTAAACAACCATCAAAGCATTAAACAGA
ENSCAFG00000010115        GTCATTCAGGAATAGGTCTTAGAAAGGGGCCATTAAACAACTACCAAAGCATTAAACAGC
ENSMUSG00000026109        GTCATTCCGGAATAGGTGTTTGATAGAAGCCATTAAACAGCCATCAAGGCCTTAAACAGC
ENSRNOG00000016623        GTCATTCCAGAATAGGTATTTGATCAAAGCCATTAAACAGCCATCAAGTCCTTAAACAGC
SINFRUG00000120979        ------------------------------------------------------------


ENSG00000144339           TACACACGGGGGAAG-TACCACAAGTACTGCCATAAGTTGCCTGGAGAGAATCGCTAAGC
ENSCAFG00000010115        TACACACATGGGAGCCTACCACAAGTACTGCCATGAGCTGGCTGTGGAGGATCCCTAAGC
ENSMUSG00000026109        TATACATGAGGGAATCTGCCACAAGTGCTACCATGATTTGGCTGCTGAGAGCCCTTAAGC
ENSRNOG00000016623        TGTACATGAGGGAGTCCACCATAAGTGCTACCATGACTTGGCTGCTGAGAGCCCTTAAGT
SINFRUG00000120979        ------------------------------------------------------------


ENSG00000144339           CTGGCAGCAGACGTGATACTACTTGGAGTAGGAATCTGGGGAGCTGAGAGTTGACAACAA
ENSCAFG00000010115        CTTGCAGCAGGTGTGATACTACTTAGAGTAGGAGTCTTGGGAGCTGGGTGTTGACAACAT
ENSMUSG00000026109        CTTGCAGCAGGTGTGATACTACTTAGAGT--CAGTCTTGGGAGCAGGGAGTTGACAACAA
ENSRNOG00000016623        CTGGCAGCAGGTGTGACACTACTTAGAGT--CAGTCTTGGGATCAGGCAGTTGACAACAA
SINFRUG00000120979        ---------------------------------------------GGTAGTTGACAACAA
                                                                       *   **********

ENSG00000144339           TGTCCAGAGGACTGGAGGTGAAAGAGCAGGCAGCTTGAGTGTGTGAGACAGATGCTGCTT
ENSCAFG00000010115        TGTCCAAAGGACTGGAGGCAAAAGAGCATGCGGCTTGAGTGTGCGAGACAGATGCTACTT
ENSMUSG00000026109        TGGCCAAAG-ACTGGAGCAGAAAGAACAGGCAGCTTGAGTGTGAAAGACAGAAGCTGCCT
ENSRNOG00000016623        TGTCCAAAAGACCGGAGCAGAAAGAGCAGACAGCTTGAGTGTGAAAGACAGAAGCTGCTT
SINFRUG00000120979        CAGA-GGAACATCCCAGCAGCTTTAAGTGACAGCTTG-----------------------
                                 *  *    **       *     * *****

ENSG00000144339           -----------TCACCATCTCCCACGTGTAATATTTTCAACACATCAGTGGAGCATTAGA
ENSCAFG00000010115        CCTTGCTGCATTCACCATCTCCCACGTGTGATATTC-CAACACATTAACAGAGCATTAGA
ENSMUSG00000026109        -----------TCCCCATCTCCCACCTGTAATATTGCCAACCCATTAACAGAGCTTTGGA
ENSRNOG00000016623        -----------TCCCCATCTCCCACATGTAATATTTCCAACACATTAACAGATCTCTGGA
SINFRUG00000120979        ------------------------------------------------------------


ENSG00000144339           GTTCGTGGCATGCCTAGTGACAGGAATAGAACCATGAAATTG-TAATGTCTTGCCAGTTT
ENSCAFG00000010115        GTTCGTGGCATGCTTAGTGACAGGAATAGAACCATGAAATTGGTAATGTCTTGCCAGTTT
ENSMUSG00000026109        GTTCACGGTGTGCTGAGTGACAGGAGCAGAACCATGAAGGTGGTAATGGCTTGCCAGGTT
ENSRNOG00000016623        GTTCATGGTGTGCTGAGTGACAGGAGCAGAACCATGAAGGTGGTAATGGCTTGCCAGGAT
SINFRUG00000120979        ------------------------------------------------------------


ENSG00000144339           TAAGATGTAGATGAATAACTTCTTGTCAGAGCAATGAAGTCTTTCTTATCCTATAAAAAA
ENSCAFG00000010115        TAAAATGTAGATGAATAACTTCTTGTCAGAGCAATGAAGTCTTTCTCATCCTATAAAAAA
ENSMUSG00000026109        TAA-ATGTAGATGAATAACCTCTTGTCAGGGCAAAGAAGTCGTTTTTATCCTATAAAAAT
ENSRNOG00000016623        TAA-ATGTAGATGAATAACCTCTTGTCAGAGCAAAGAAGTCATTTTTAGCCTATAAAAAT
SINFRUG00000120979        ------------------------------------------------------------


ENSG00000144339           TCCACATATAACTAATGAGACTCTTGGGCCATCATACCTGCTCTGTTCAATGTCTTACAC
ENSCAFG00000010115        TTTGTGTATAACTAATGAGACTCCTGAGCCATCATACCTGTTCAATTCAATTTCTTACAC
ENSMUSG00000026109        TCTGTGCATAACTAATGAGACACCTGGGCCATCATACCTGTTATAGTCAATTTCC-----
ENSRNOG00000016623        TCTGTGCATAACTAATGAGACACCTGGGCCATCATACCTGCTCTAGTCAATTTCC-----
SINFRUG00000120979        ------------------------------------------------------------


ENSG00000144339           TGATTTGGCTGTTCAACTA
ENSCAFG00000010115        TAGTTTGGCTGTTCAACTA
ENSMUSG00000026109        -AGTTTAGCTGTGTATCTA
ENSRNOG00000016623        -AGTTTAGCTGCGTATCTA
SINFRUG00000120979        -------------------
```

Fragment Tmeff2_26015

Representation of conserved regions within their genic context

Within the mouse context is shown the rCNE from which the tested SCE is derived (red diamond)

Within the fugu context all CHAOS hits obtained from the mouse rCNE are shown (green diamonds, in red the one tested)

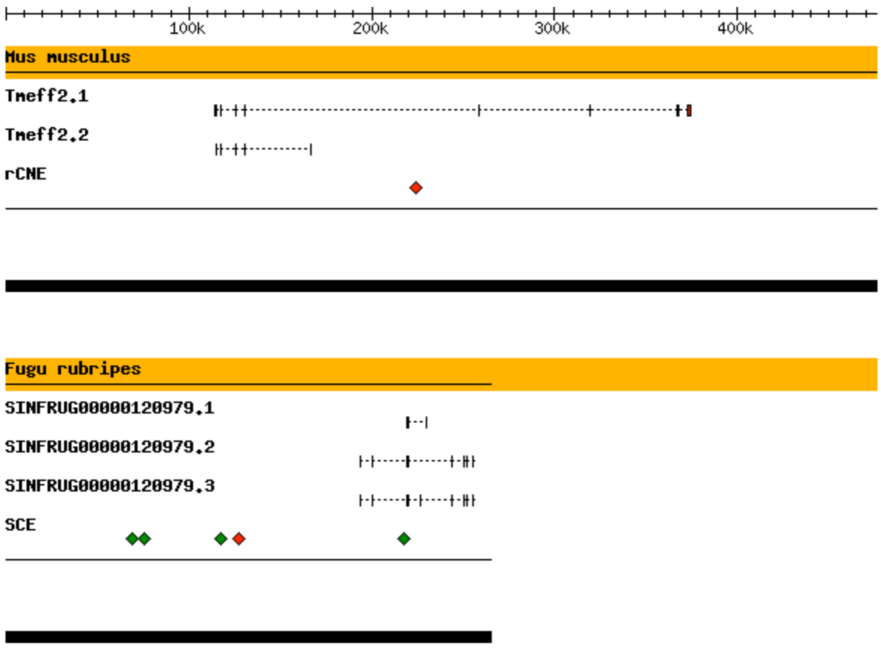

Conserved regions shown within 2000bp context

The mouse context contains:

-rCNEs green boxes, in red the one considered for this test

-SCEs yellow boxes, in red the one considered for this test

-CHAOS hits blue boxes, in red the one considered for this test

The fugu context contains:

-the fragment amplified in this test

-CHAOS hits blue boxes, in red the one considered for this test

MOUSE IMAGE TMEFF2 MOV FRAGMENT CODING 0 HIT: 945164

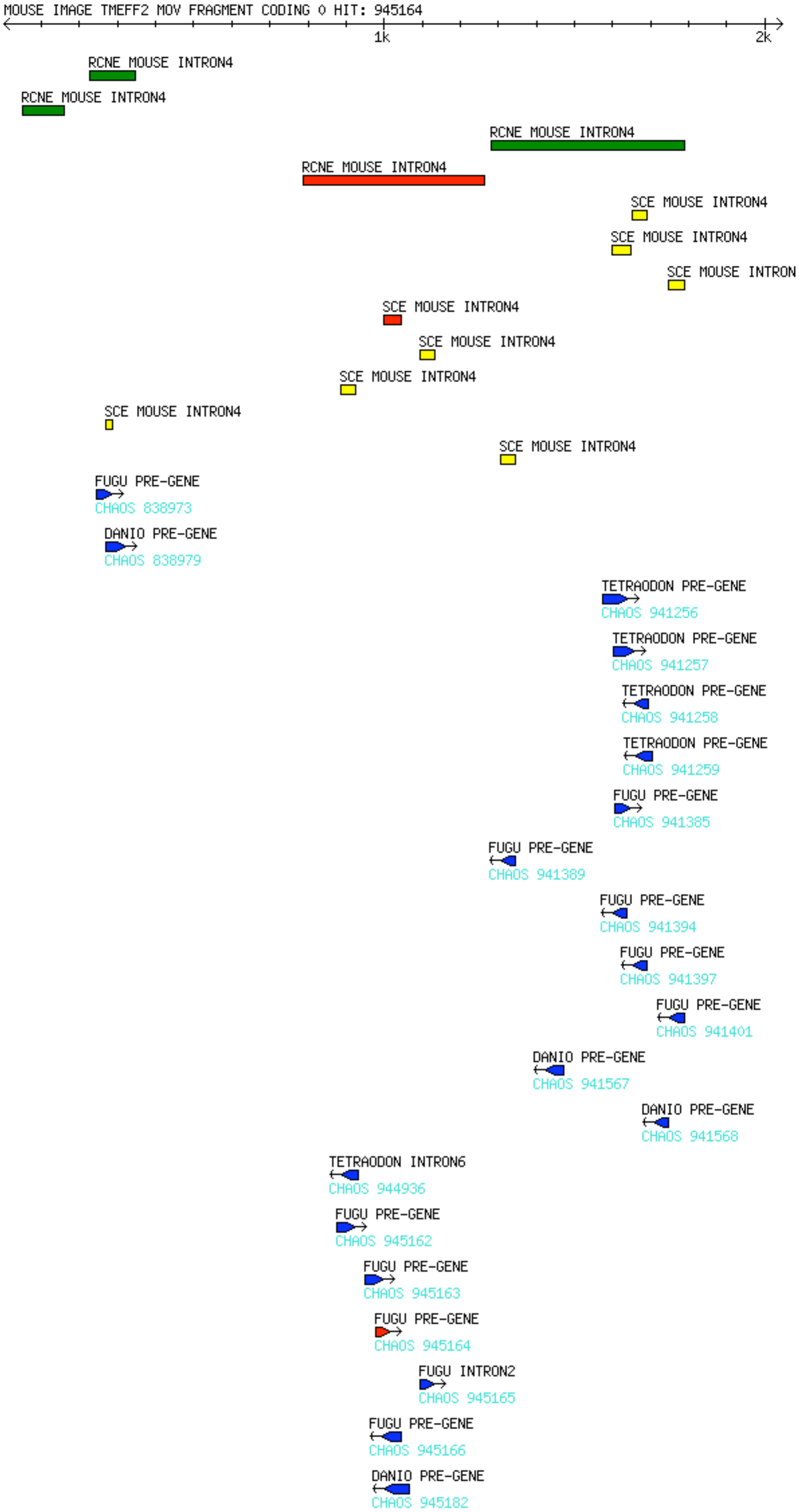

FUGU IMAGE TMEFF2 MOV FRAGMENT CODING 0 HIT: 945164

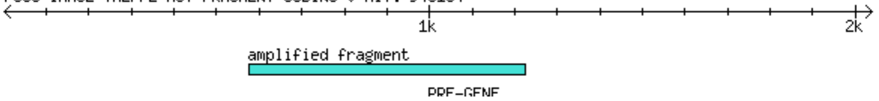

SEQUENCES

```
>fugu_fragment_for_primer_design_26015
GATTTGTCAACACACTCGGCCGGGATAATTGACTGTTCTCTGGTGGGTATAAATAATGAC
TTCTGATTTATTCCACGACGACATAAATCATCCGAAGGCTTATCCGGATGCACAGCCTTC
CGCACACAAGGGAGAGGCTCTTGTATGGAGGAAATCTCATTTGTTGTCTCTTATTTCACG
TATGAGTAAATCTGATTAGTTGTATTATGAAGTTTCTCAGTCAAATTATTTGCTATATAT
TTGTTTGGAAGATGTTTAATTATGTTTGGAATTAAAAAAATAAAAGGCTGCTCGAGATTT
GGATACATCTGAAAAATAGACTGTGTATTTCTCTTATTATTATTTTGATTGACGACTGCA
TAATCAATACGATGTGCTGTTGTGTGTAATAATAGGGGTCTTGTCTTGATGGACGTCAAA
GCAACCCAGCAGTGGTGTATGGATCAGAGACGATGGCGGCGTCAACAAACAAACAGACAA
CATAAAGTTTCAAGGCAGTGCCGGAGGAGGCAGAGCTAAAAGTGCCTATTCAAGAGTGAT
GGAGATGGTCAGGATTAGGGTGGAGGACATCACGGAGATAGCACAGGCCAGATGAATCGG
AGATGGCAGACACAGATGTTGTTGGACATGTGCAAAAGTTGGATAGTGGGAATGCTGCTA
GAAGGACGGTAGCTGTCCACTGGTTTATGTATGTGGTGAATAAGGACACGCTGATGTTCT
CATGCAGATGGGTGCTGATGTCAGTCTTAGGAATGGATGGATGTTAAAACTCCATTTGTC
ACTGGAGCTCGAAGGGTAATTGTTGTGACACTGATGCATCCTTTAGTGTTATTTTTCACA
CCCCAGTCTTCACTGGTACTGGTTTGTGCTTATTTTATTAATGGGGCACTTCCTGTTGCA
AAAAATGGTGCCAATCCTACTTTTATTAACCTGTAACCTAAGCAAGGCCTTAGAAATTAA
GTTAAAATTAAATCATATGAAGTAACTTTTCTGATGCTTTAGGGTATATGGTATATGCAC
AGTGTGTGTGAAAGAAGAAACTGACTGCTAATATAGGTCCTCTTAAAGGCCTGCAGGCAT
GTCTGAAGTCTTCTGACCAAAAACAAACTCTTCATTTCCTACCAGGAATACAAATGTTGC
ACCGTTGATGGAGGAATTCAGTCAATTAGAAATATGGTAATTTGATCAGCATTTTAGATC
ATTGCTCATCTTTTTATCGCTCCTGAATCTACAAATAAGTGCTTGCAATTGTGCTAATGT
CAAGGAGTGTGCGCCCCNNNNNNNNNNNNNNNNNNNNNNNNNNNNNNNNNNNNNNNNNTA
TACATTACATCAGCATAATCATCATTCACACTACCTACAGGCACCAAACACTCTCCCAAC
CATACATGACGTTAATTAAGAGGGGAGTGAAAACCACAGCATCTCGTTTGTGCTGGGAAT
AAAACTGGGAAGCGCGTGTGTGTGGCTGTGCATTTTGATGGAAACATGCTGGCACCGGGA
AAATCTAAATAAATAAATTTACTATGCGGTGATTAGCTGGTTGAATAATTTGAGATATGA
TTACAGTTAATCAAGAGCAAACGAGAGGNNNNNNNNNNNNNNNNNNNNNNNNNNNNNNNN
NAAGTACATGTGTTTTGCGCATCAGCCTCCAGCTGCCCCACTGCTTACAAACTGTCAGCA
GCCATCATGGAGGAGGGCCCTCATTGTGTGAAGGACAAAGGCGCCACGTGTCACCAGTGG
GAGATAAGGGTGATTTATGAGCACCTGTCAGAGCAGTGGCAGGTAAACATGCACNNNNNN
NNNNNNNNNNNNNNNNNNNNNCTGGTTTTGGGGAGTGATCTCACCACCGTTCTCCAAATG
GAGGCACTGGTATCTAATTAACACTATCACTACATCTGGATTGCTGGGATTCTGAATTTG
GTCTGAAAGGTCAAAACCTTTTGGGGCAAGAGCGTGACGTGTGGCTGTGGAGTCAAAAGA
CCAATACAGAGGCCAACATGAGCGGAATCCTCAGGGCAAACTGGGAAAAAGGCAAAGAC
```

```
>amplified_fugu_segment
AGATAGCACAGGCCAGATGAATCGGAGATGGCAGACACAGATGTTGTTGGACATGTGCAA
AAGTTGGATAGTGGGAATGCTGCTAGAAGGACGGTAGCTGTCCACTGGTTTATGTATGTG
GTGAATAAGGACACGCTGATGTTCTCATGCAGATGGGTGCTGATGTCAGTCTTAGGAATG
GATGGATGTTAAAACTCCATTTGTCACTGGAGCTCGAAGGGTAATTGTTGTGACACTGAT
GCATCCTTTAGTGTTATTTTTCACACCCCAGTCTTCACTGGTACTGGTTTGTGCTTATTT
TATTAATGGGGCACTTCCTGTTGCAAAAAATGGTGCCAATCCTACTTTTATTAACCTGTA
ACCTAAGCAAGGCCTTAGAAATTAAGTTAAAATTAAATCATATGAAGTAACTTTTCTGAT
GCTTTAGGGTATATGGTATATGCACAGTGTGTGTGAAAGAAGAAACTGACTGCTAATATA
GGTCCTCTTAAAGGCCTGCAGGCATGTCTGAAGTCTTCTGACCAAAAACAAACTCTTCAT
TTCCTACCAGGAATACAAATGTTGCACCGTTGATGGAGGAATTCAGTCAATTAGAAATAT
GGTAATTTGATCAGCATTTTAGATCATTGCTCATCTTTTTATCGCTCCT
```

```
>rCNE_mouse_sequence_837802
GTCATTCCGGAATAGGTGTTTGATAGAAGCCATTAAACAGCCATCAAGGCCTTAAACAGC
TATACATGAGGGAATCTGCCACAAGTGCTACCATGATTTGGCTGCTGAGAGCCCTTAAGC
CTTGCAGCAGGTGTGATACTACTTAGAGTCAGTCTTGGGAGCAGGGAGTTGACAACAATG
GCCAAAGACTGGAGCAGAAAGAACAGGCAGCTTGAGTGTGAAAGACAGAAGCTGCCTTCC
CCATCTCCCACCTGTAATATTGCCAACCCATTAACAGAGCTTTGGAGTTCACGGTGTGCT
GAGTGACAGGAGCAGAACCATGAAGGTGGTAATGGCTTGCCAGGTTTAAATGTAGATGAA
TAACCTCTTGTCAGGGCAAAGAAGTCGTTTTTATCCTATAAAAATTCTGTGCATAACTAA
TGAGACACCTGGGCCATCATACCTGTTATAGTCAATTTCCAGTTTAGCTGTGTATCTA
```

>SCE_mouse_sequence_837950
GAGTGTGAAAGACAGAAGCTGCCTTCCCCATCTCCCACCTGTAATA

FUGU HITS IN THE CONTEST OF MAMMALIAN rCNE

CLUSTAL W (1.83) multiple sequence alignment

```
ENSG00000144339         GTCATTCACGAATAGGTGTTTGAGAGGAGCCATTAAACAACCATCAAAGCATTAAACAGA
ENSCAFG00000010115      GTCATTCAGGAATAGGTCTTAGAAAGGGGCCATTAAACAACTACCAAAGCATTAAACAGC
ENSRNOG00000016623      GTCATTCCAGAATAGGTATTTGATCAAAGCCATTAAACAGCCATCAAGTCCTTAAACAGC
ENSMUSG00000026109      GTCATTCCGGAATAGGTGTTTGATAGAAGCCATTAAACAGCCATCAAGGCCTTAAACAGC
SINFRUG00000120979      ------------------------------------------------------------


ENSG00000144339         TACACACGGGGGAAG-TACCACAAGTACTGCCATAAGTTGCCTGGAGAGAATCGCTAAGC
ENSCAFG00000010115      TACACACATGGGAGCCTACCACAAGTACTGCCATGAGCTGGCTGTGGAGGATCCCTAAGC
ENSRNOG00000016623      TGTACATGAGGGAGTCCACCATAAGTGCTACCATGACTTGGCTGCTGAGAGCCCTTAAGT
ENSMUSG00000026109      TATACATGAGGGAATCTGCCACAAGTGCTACCATGATTTGGCTGCTGAGAGCCCTTAAGC
SINFRUG00000120979      ------------------------------------------------------------


ENSG00000144339         CTGGCAGCAGACGTGATACTACTTGGAGTAGGAATCTGGGGAGCTGAGAGTTGACAACAA
ENSCAFG00000010115      CTTGCAGCAGGTGTGATACTACTTAGAGTAGGAGTCTTGGGAGCTGGGTGTTGACAACAT
ENSRNOG00000016623      CTGGCAGCAGGTGTGACACTACTTAGAGT--CAGTCTTGGGATCAGGCAGTTGACAACAA
ENSMUSG00000026109      CTTGCAGCAGGTGTGATACTACTTAGAGT--CAGTCTTGGGAGCAGGGAGTTGACAACAA
SINFRUG00000120979      ------------------------------------------------------------


ENSG00000144339         TGTCCAGAGGACTGGAGGTGAAAGAGCAGGCAGCTTGAGTGTGTGAGACAGATGCTGCTT
ENSCAFG00000010115      TGTCCAAAGGACTGGAGGCAAAAGAGCATGCGGCTTGAGTGTGCGAGACAGATGCTACTT
ENSRNOG00000016623      TGTCCAAAAGACCGGAGCAGAAAGAGCAGACAGCTTGAGTGTGAAAGACAGAAGCTGCTT
ENSMUSG00000026109      TGGCCAAA-GACTGGAGCAGAAAGAACAGGCAGCTTGAGTGTGAAAGACAGAAGCTGCCT
SINFRUG00000120979      ------------TAGGGTATATGGTATATGCACAGTGTGTGTGAAAGAAGAAA-------
                                      * *   *  *   *  *    ** *****  ***   *

ENSG00000144339         -----------TCACCATCTCCCACGTGTAATATTTTCAACACATCAGTGGAGCATTAGA
ENSCAFG00000010115      CCTTGCTGCATTCACCATCTCCCACGTGTGATATTC-CAACACATTAACAGAGCATTAGA
ENSRNOG00000016623      -----------TCCCCATCTCCCACATGTAATATTTCCAACACATTAACAGATCTCTGGA
ENSMUSG00000026109      -----------TCCCCATCTCCCACCTGTAATATTGCCAACCCATTAACAGAGCTTTGGA
SINFRUG00000120979      ------------------------------------------------------------


ENSG00000144339         GTTCGTGGCATGCCTAGTGACAGGAATAGAACCATGAAATTG-TAATGTCTTGCCAGTTT
ENSCAFG00000010115      GTTCGTGGCATGCTTAGTGACAGGAATAGAACCATGAAATTGGTAATGTCTTGCCAGTTT
ENSRNOG00000016623      GTTCATGGTGTGCTGAGTGACAGGAGCAGAACCATGAAGGTGGTAATGGCTTGCCAGGAT
ENSMUSG00000026109      GTTCACGGTGTGCTGAGTGACAGGAGCAGAACCATGAAGGTGGTAATGGCTTGCCAGGTT
SINFRUG00000120979      ------------------------------------------------------------


ENSG00000144339         TAAGATGTAGATGAATAACTTCTTGTCAGAGCAATGAAGTCTTTCTTATCCTATAAAAAA
ENSCAFG00000010115      TAAAATGTAGATGAATAACTTCTTGTCAGAGCAATGAAGTCTTTCTCATCCTATAAAAAA
ENSRNOG00000016623      TAA-ATGTAGATGAATAACCTCTTGTCAGAGCAAAGAAGTCATTTTTAGCCTATAAAAAT
ENSMUSG00000026109      TAA-ATGTAGATGAATAACCTCTTGTCAGGGCAAAGAAGTCGTTTTTATCCTATAAAAAT
SINFRUG00000120979      ------------------------------------------------------------


ENSG00000144339         TCCACATATAACTAATGAGACTCTTGGGCCATCATACCTGCTCTGTTCAATGTCTTACAC
ENSCAFG00000010115      TTTGTGTATAACTAATGAGACTCCTGAGCCATCATACCTGTTCAATTCAATTTCTTACAC
ENSRNOG00000016623      TCTGTGCATAACTAATGAGACACCTGGGCCATCATACCTGCTCTAGTCAATTTCC-----
ENSMUSG00000026109      TCTGTGCATAACTAATGAGACACCTGGGCCATCATACCTGTTATAGTCAATTTCC-----
SINFRUG00000120979      ------------------------------------------------------------


ENSG00000144339         TGATTTGGCTGTTCAACTA
ENSCAFG00000010115      TAGTTTGGCTGTTCAACTA
ENSRNOG00000016623      -AGTTTAGCTGCGTATCTA
ENSMUSG00000026109      -AGTTTAGCTGTGTATCTA
SINFRUG00000120979      -------------------
```

Fragment Tmeff2_26016

Representation of conserved regions within their genic context

Within the mouse context is shown the rCNE from which the tested SCE is derived (red diamond)

Within the fugu context all CHAOS hits obtained from the mouse rCNE are shown (green diamonds, in red the one tested)

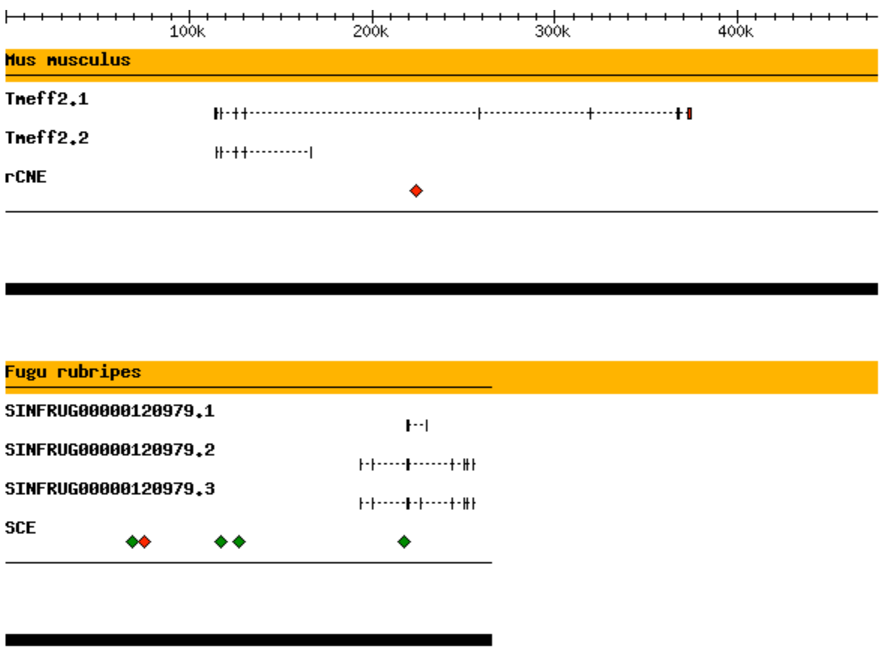

Conserved regions shown within 2000bp context

The mouse context contains:

-rCNEs green boxes, in red the one considered for this test

-SCEs yellow boxes, in red the one considered for this test

-CHAOS hits blue boxes, in red the one considered for this test

The fugu context contains:

-the fragment amplified in this test

-CHAOS hits blue boxes, in red the one considered for this test

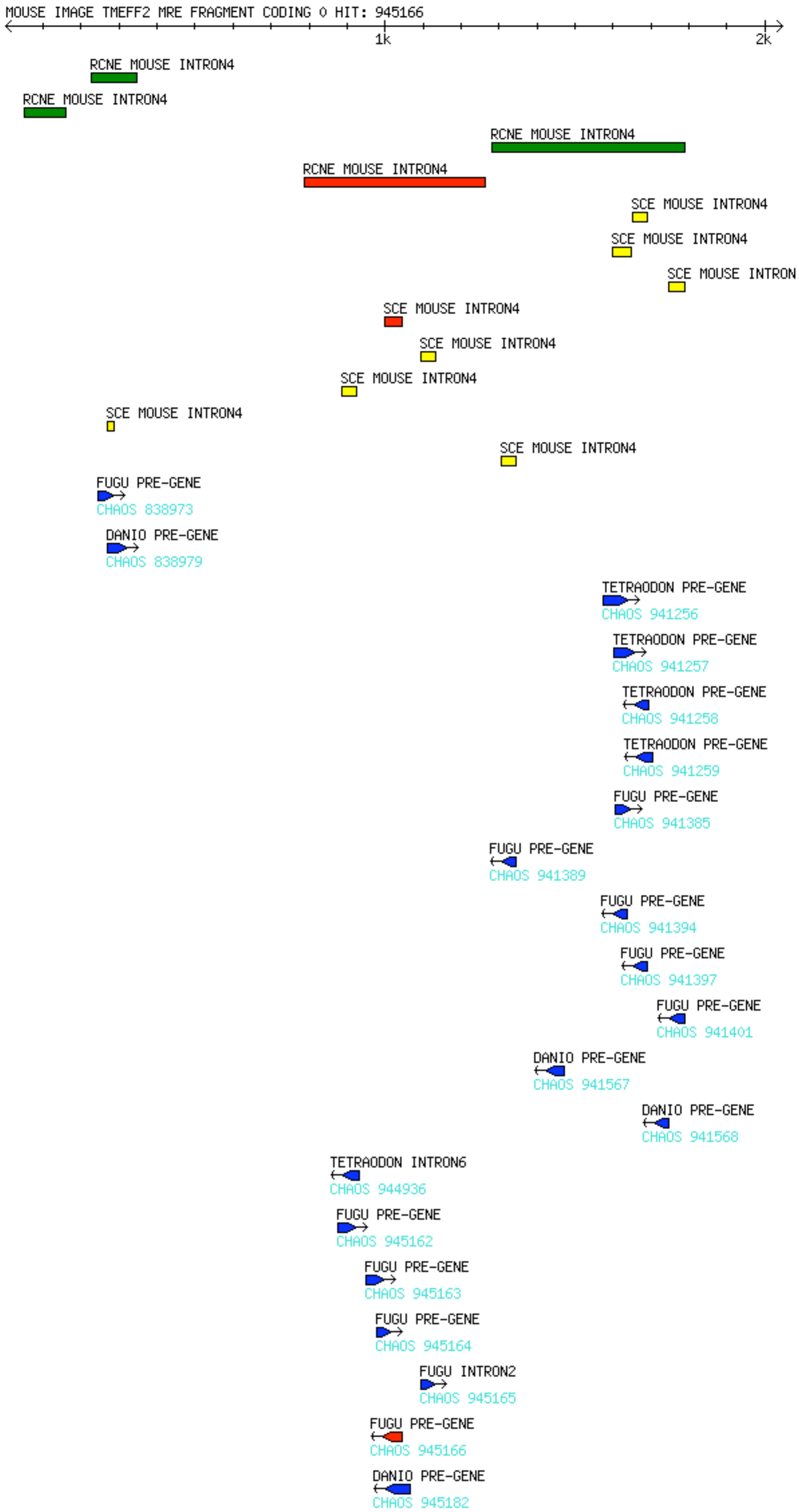
MOUSE IMAGE TMEFF2 MRE FRAGMENT CODING 0 HIT: 945166
1k
2k
RCNE MOUSE INTRON4
RCNE MOUSE INTRON4
RCNE MOUSE INTRON4
RCNE MOUSE INTRON4
SCE MOUSE INTRON4
SCE MOUSE INTRON4
SCE MOUSE INTRON
SCE MOUSE INTRON4
SCE MOUSE INTRON4
SCE MOUSE INTRON4
SCE MOUSE INTRON4
SCE MOUSE INTRON4
FUGU PRE-GENE
CHAOS 838973
DANIO PRE-GENE
CHAOS 838979
TETRAODON PRE-GENE
CHAOS 941256
TETRAODON PRE-GENE
CHAOS 941257
TETRAODON PRE-GENE
CHAOS 941258
TETRAODON PRE-GENE
CHAOS 941259
FUGU PRE-GENE
CHAOS 941385
FUGU PRE-GENE
CHAOS 941389
FUGU PRE-GENE
CHAOS 941394
FUGU PRE-GENE
CHAOS 941397
FUGU PRE-GENE
CHAOS 941401
DANIO PRE-GENE
CHAOS 941567
DANIO PRE-GENE
CHAOS 941568
TETRAODON INTRON6
CHAOS 944936
FUGU PRE-GENE
CHAOS 945162
FUGU PRE-GENE
CHAOS 945163
FUGU PRE-GENE
CHAOS 945164
FUGU INTRON2
CHAOS 945165
FUGU PRE-GENE
CHAOS 945166
DANIO PRE-GENE
CHAOS 945182

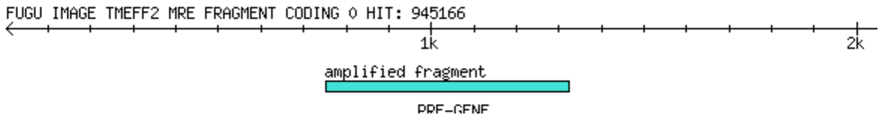
FUGU IMAGE TMEFF2 MRE FRAGMENT CODING 0 HIT: 945166
1k
2k
amplified fragment

SEQUENCES

```
>fugu_fragment_for_primer_design_26016
ATAACTATTTAGTTTTTAGTTATTTCTTCTATTTGGGGGCTTGTTTCCCATTTATGTGGG
ATGCGATCATAAACGTGGAAGGATTCCAAACTGTGTAAGGTTGAGTTCTCACGCGGCTCT
ACGATACTCTATGGTAAAGACTCCCATGTAAAGCCAATAAAAGCACAGGTGAGGTGCGTG
GGAAGAGGGCCTTCAAACATTTGCAGGCACGCTGGCATAAATTCATGAGCAATCTGCTTT
TACTGGTGCGCAGCCGTCCTCAATAAGGCTGGGAAACGTCTCCGGGCTCTTCTAGGGGGC
TGCCTCGTGGTGAGGAGCTATAAACTAACACCGACGAGACTGAATCCTGAAGAGCGCGAG
CGTTGACCTGCTCAGACGGAGGTCTTCCCTCCCTGTCACCCAGTGTGGCTCTGGAACTTG
TCTCGGGCCAATTAATCTGCTTTGAGACAGGTGTCTAAGCCTGCATGTCGAGCGGGGATC
TATTTCCTCTCTGCCTCCAGCTGGCTCCTGAAGCACATCCACCGGGTGTCACAGCGTCTG
ATTACCTGCTGTCCCGGCCAGTCAGCCTTAGCTGAACCAATTGCTTTTGCTTTGTAAAGT
ACTGGTAAACATTCTGAACCAAATGCGCGTTAGGAACGGCGCCGCTTGAAGACACGNNNN
NNNNNNNNNNNNNNNNNNNNNNNACCTATTCATGCAAAACTGCATTTAGCAAATTATTAC
TAATAAGACTGACGATCATTTTGATTTTTCCTTTGGGGAGAAACAATGTAGGCTAACACC
TGAAGATCTCATAGCTGCTTTGCCTTGAGCAGAATAAGGAGAGAGCAGGTAATCGGCCCA
AACGTCATAATCACTTCAGTATTTAGCACGTTATTCGGGCTACACTTGTAAATAAAACTC
AGTGATTCCGCTGCTTTTCAATCCNNNNNNNNAGTAGTAATTGGGTATTTATCCTGCATA
CTGATGTTAGCAGCAAACCGCGCTGTCAACACCCGGATGTGTTCCGGTTGGGAAGTTGCT
AAAATGACGATTCCCCTTAAAACTCAAGCTGCAGTTGGTCAGCACACGACCAAAGCAGGA
GAGGTGACTGGTGGCTCCGGGGTCGCTGACCCCAGCAGCGGACCAGCTACGCCTGCTCCG
ATCGGCTGCAGGCTCCCCGAGCTCGTGGAAGGACAGGATGTCTTCCTCTGATCAGCGTCT
CTCTTCTTTCCTGTCCTCCGTCCTCAGTGGTGTCCAGCCTCTGGAAAAGGCTGAATATTT
TAACGAAGTATTTTAAAGTATTCCTCCGTCTTTTTGGCATCCTGGATCATGGAGGAGGGT
TTCTCGAGTTCTTTCGCTGAAGNNNNNNNNNNNNNCAATCGCATTCTCTTTAAATGTAAA
AGCATCTCCGATCAATAATCAACGCATCTTTTTCTAAATAACTCATGAGTCTGCGAAACT
TGACGGCCCCTCTCTCACTCAGAATAAGCGATTCCTTCAGTGCTTGACAGCTGGATCTCA
AAGCAAAAGAAGGAGATTTCAGGCCCTGGGGACCGGAATCCTTTCAAAGTCCGGGGGGAG
GGACGGGTAGATCTGTGGACCGCCTGTTCTTCACTGGCAGGCTGCAGTAACAGAAGGATG
ACGGATACATCTCCTAATGTGTTCTGACCTTTGCCAGGAAGGTTCTTCTAATGGTGTCAA
AAACTACAGATGAAGACGCCTTCAGAAAGGGAAAAAAGAGCGTTTAATATTTGATGCCTG
AGTTCACTTTGCCGAGCCGAGAGGAATCTGGGAAAGAAAGCTGCCACTGAAGTCGGATGT
GTCGTCTTTCTCTTCCTGACGGTATCAGCTTGTCCCGAAAGAGGAGAGACGACAGTGAGC
GTCAGCGCTTTAATCTGAGGCTGGTAACGATGAAGCTTGGCCACGACACGCTCACGCTCC
CTTTTCTGTCCATCCTGGGCCATTTTCCTTCCTGATTAGCCCTTTTTCTTTTGGTGGCTC
TCAGTTCTCCTGTCATCATTTCTTGTGGATCCTTGTTTTCTTTTGGGTTTGTCTGGATTT
GCATCAGAAAAT
```

```
>amplified_fugu_segment
TGGGGAGAAACAATGTAGGCTAACACCTGAAGATCTCATAGCTGCTTTGCCTTGAGCAGA
ATAAGGAGAGAGCAGGTAATCGGCCCAAACGTCATAATCACTTCAGTATTTAGCACGTTA
TTCGGGCTACACTTGTAAATAAAACTCAGTGATTCCGCTGCTTTTCAATCCNNNNNNNNA
GTAGTAATTGGGTATTTATCCTGCATACTGATGTTAGCAGCAAACCGCGCTGTCAACACC
CGGATGTGTTCCGGTTGGGAAGTTGCTAAAATGACGATTCCCCTTAAAACTCAAGCTGCA
GTTGGTCAGCACACGACCAAAGCAGGAGAGGTGACTGGTGGCTCCGGGGTCGCTGACCCC
AGCAGCGGACCAGCTACGCCTGCTCCGATCGGCTGCAGGCTCCCCGAGCTCGTGGAAGGA
CAGGATGTCTTCCTCTGATCAGCGTCTCTCTTCTTTCCTGTCCTCCGTCCTCAGTGGTGT
CCAGCCTCTGGAAAAGGCTGAATATTTTAACGAAGTATTTTAAAGTATTCCTCCGTCTTT
TTGGCATCCTGGATCATGGAGGAGGGTTTCT
```

```
>rCNE_mouse_sequence_837802
GTCATTCCGGAATAGGTGTTTGATAGAAGCCATTAAACAGCCATCAAGGCCTTAAACAGC
TATACATGAGGGAATCTGCCACAAGTGCTACCATGATTTGGCTGCTGAGAGCCCTTAAGC
CTTGCAGCAGGTGTGATACTACTTAGAGTCAGTCTTGGGAGCAGGGAGTTGACAACAATG
GCCAAAGACTGGAGCAGAAAGAACAGGCAGCTTGAGTGTGAAAGACAGAAGCTGCCTTCC
CCATCTCCCACCTGTAATATTGCCAACCCATTAACAGAGCTTTGGAGTTCACGGTGTGCT
GAGTGACAGGAGCAGAACCATGAAGGTGGTAATGGCTTGCCAGGTTTAAATGTAGATGAA
TAACCTCTTGTCAGGGCAAAGAAGTCGTTTTTATCCTATAAAAATTCTGTGCATAACTAA
TGAGACACCTGGGCCATCATACCTGTTATAGTCAATTTCCAGTTTAGCTGTGTATCTA
```

```
>SCE_mouse_sequence_837950
GAGTGTGAAAGACAGAAGCTGCCTTCCCCATCTCCCACCTGTAATA
```

FUGU HITS IN THE CONTEST OF MAMMALIAN rCNE

CLUSTAL W (1.83) multiple sequence alignment

```
ENSMUSG00000026109      GTCATTCCGGAATAGGTGTTTGATAGAAGCCATTAAACAGCCATCAAGGCCTTAAACAGC
ENSRNOG00000016623      GTCATTCCAGAATAGGTATTTGATCAAAGCCATTAAACAGCCATCAAGTCCTTAAACAGC
ENSCAFG00000010115      GTCATTCAGGAATAGGTCTTAGAAAGGGGCCATTAAACAACTACCAAAGCATTAAACAGC
ENSG00000144339         GTCATTCACGAATAGGTGTTTGAGAGGAGCCATTAAACAACCATCAAAGCATTAAACAGA
SINFRUG00000120979      ------------------------------------------------------------


ENSMUSG00000026109      TATACATGAGGGAATCTGCCACAAGTGCTACCATGATTTGGCTGCTGAGAGCCCTTAAGC
ENSRNOG00000016623      TGTACATGAGGGAGTCCACCATAAGTGCTACCATGACTTGGCTGCTGAGAGCCCTTAAGT
ENSCAFG00000010115      TACACACATGGGAGCCTACCACAAGTACTGCCATGAGCTGGCTGTGGAGGATCCCTAAGC
ENSG00000144339         TACACACGGGGGAAG-TACCACAAGTACTGCCATAAGTTGCCTGGAGAGAATCGCTAAGC
SINFRUG00000120979      ------------------------------------------------------------


ENSMUSG00000026109      CTTGCAGCAGGTGTGATACTACTTAGAGTC--AGTCTTGGGAGCAGGGAGTTGACAACAA
ENSRNOG00000016623      CTGGCAGCAGGTGTGACACTACTTAGAGTC--AGTCTTGGGATCAGGCAGTTGACAACAA
ENSCAFG00000010115      CTTGCAGCAGGTGTGATACTACTTAGAGTAGGAGTCTTGGGAGCTGGGTGTTGACAACAT
ENSG00000144339         CTGGCAGCAGACGTGATACTACTTGGAGTAGGAATCTGGGGAGCTGAGAGTTGACAACAA
SINFRUG00000120979      ------------------------------------------------------------


ENSMUSG00000026109      TGGCCAAA-GACTGGAGCAGAAAGAACAGGCAGCTTGAGTGTGAAAGACAGAAGCTGCCT
ENSRNOG00000016623      TGTCCAAAAGACCGGAGCAGAAAGAGCAGACAGCTTGAGTGTGAAAGACAGAAGCTGCTT
ENSCAFG00000010115      TGTCCAAAGGACTGGAGGCAAAAGAGCATGCGGCTTGAGTGTGCGAGACAGATGCTACTT
ENSG00000144339         TGTCCAGAGGACTGGAGGTGAAAGAGCAGGCAGCTTGAGTGTGTGAGACAGATGCTGCTT
SINFRUG00000120979      ----------------------------TGCAGCTTGAGTTTTAAGGGGAATCGTCATTT
                                                      * ******** *    *  *   *     *

ENSMUSG00000026109      TCC-----------CCATCTCCCACCTGTAATATTGCCAACCCATTAACAGAGCTTTGGA
ENSRNOG00000016623      TCC-----------CCATCTCCCACATGTAATATTTCCAACACATTAACAGATCTCTGGA
ENSCAFG00000010115      CCTTGCTGCATTCACCATCTCCCACGTGTGATATT-CCAACACATTAACAGAGCATTAGA
ENSG00000144339         TCA-----------CCATCTCCCACGTGTAATATTTTCAACACATCAGTGGAGCATTAGA
SINFRUG00000120979      TAG-----------CAACTTCCCAACCGGAACA---------------------------
                                      * *  *****   *  * *

ENSMUSG00000026109      GTTCACGGTGTGCTGAGTGACAGGAGCAGAACCATGAAGGTGGTAATGGCTTGCCAGGTT
ENSRNOG00000016623      GTTCATGGTGTGCTGAGTGACAGGAGCAGAACCATGAAGGTGGTAATGGCTTGCCAGGAT
ENSCAFG00000010115      GTTCGTGGCATGCTTAGTGACAGGAATAGAACCATGAAATTGGTAATGTCTTGCCAGTTT
ENSG00000144339         GTTCGTGGCATGCCTAGTGACAGGAATAGAACCATGAAATTG-TAATGTCTTGCCAGTTT
SINFRUG00000120979      ------------------------------------------------------------


ENSMUSG00000026109      TAAA-TGTAGATGAATAACCTCTTGTCAGGGCAAAGAAGTCGTTTTTATCCTATAAAAAT
ENSRNOG00000016623      TAAA-TGTAGATGAATAACCTCTTGTCAGAGCAAAGAAGTCATTTTTAGCCTATAAAAAT
ENSCAFG00000010115      TAAAATGTAGATGAATAACTTCTTGTCAGAGCAATGAAGTCTTTCTCATCCTATAAAAAA
ENSG00000144339         TAAGATGTAGATGAATAACTTCTTGTCAGAGCAATGAAGTCTTTCTTATCCTATAAAAAA
SINFRUG00000120979      ------------------------------------------------------------


ENSMUSG00000026109      TCTGTGCATAACTAATGAGACACCTGGGCCATCATACCTGTTATAGTCAATTTCC-----
ENSRNOG00000016623      TCTGTGCATAACTAATGAGACACCTGGGCCATCATACCTGCTCTAGTCAATTTCC-----
ENSCAFG00000010115      TTTGTGTATAACTAATGAGACTCCTGAGCCATCATACCTGTTCAATTCAATTTCTTACAC
ENSG00000144339         TCCACATATAACTAATGAGACTCTTGGGCCATCATACCTGCTCTGTTCAATGTCTTACAC
SINFRUG00000120979      ------------------------------------------------------------


ENSMUSG00000026109      -AGTTTAGCTGTGTATCTA
ENSRNOG00000016623      -AGTTTAGCTGCGTATCTA
ENSCAFG00000010115      TAGTTTGGCTGTTCAACTA
ENSG00000144339         TGATTTGGCTGTTCAACTA
SINFRUG00000120979      -------------------
```

Fragment Pax6_15696

Representation of conserved regions within their genic context

Within the mouse context is shown the rCNE from which the tested SCE is derived (red diamond)

Within the fugu context all CHAOS hits obtained from the mouse rCNE are shown (green diamonds, in red the one tested)

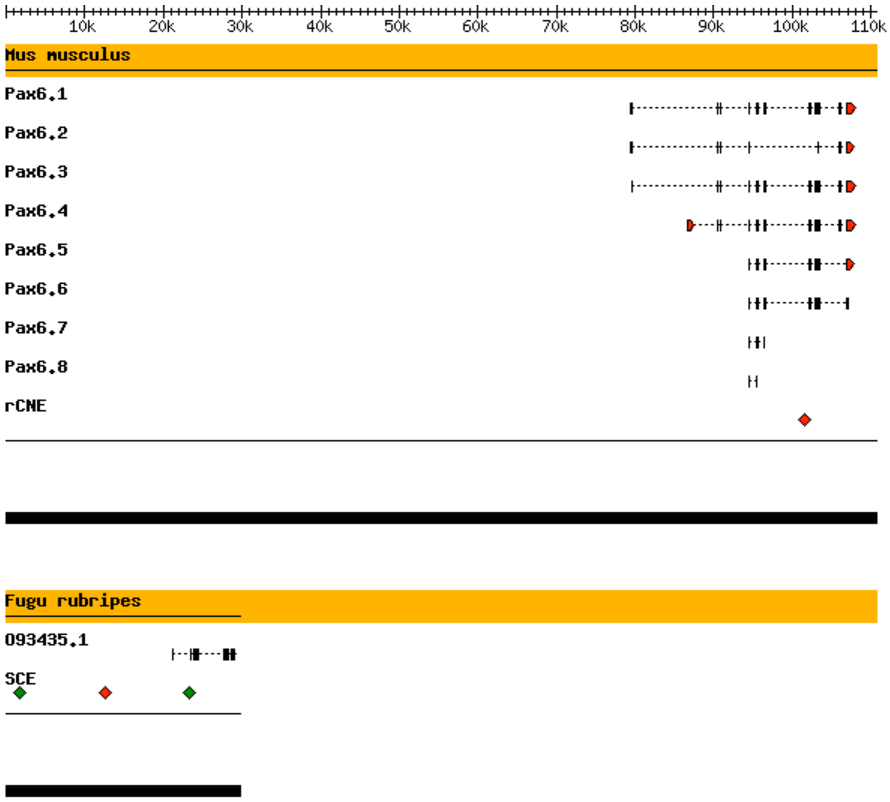

Conserved regions shown within 2000bp context

The mouse context contains:

-rCNEs green boxes, in red the one considered for this test

-SCEs yellow boxes, in red the one considered for this test

-CHAOS hits blue boxes, in red the one considered for this test

The fugu context contains:

-the fragment amplified in this test

-CHAOS hits blue boxes, in red the one considered for this test

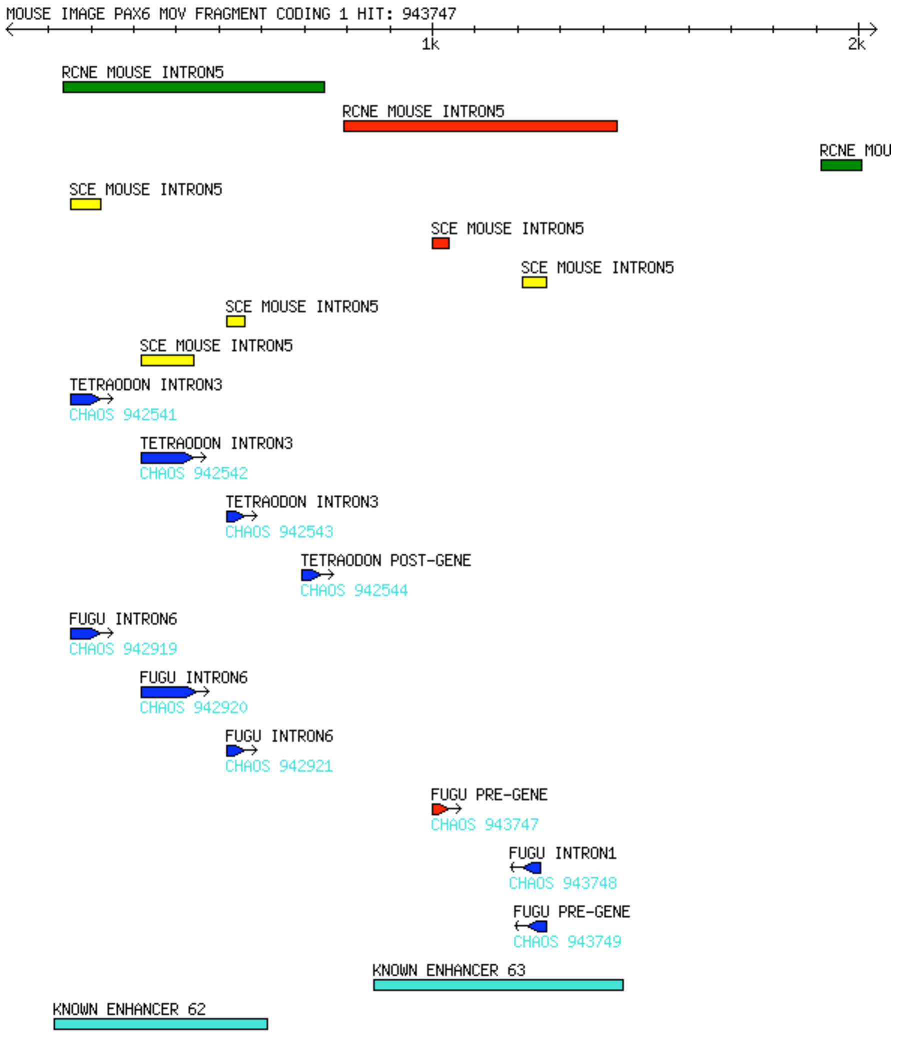

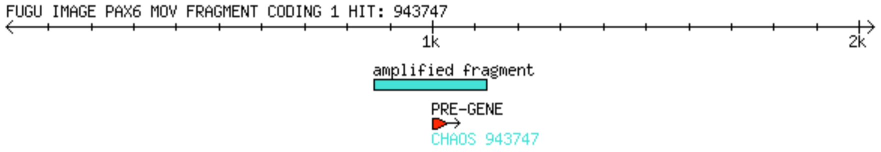

SEQUENCES

```
>fugu_fragment_for_primer_design_15696
ATCTGCTTCTGGAGAGCGCGGCTGTGTGGGAAGGGAAAGGGAGCACCTGGCTCGACCGCT
GTATACCCATCTCGTTTTATTGACAGGCTGATAAGCAGGGACCGAGCCGTGCGGTTTCCA
GCGCGCATGTCGAGCACCCTGCTGCGTTGCACAGTATCGAGACCTCTTAATGGCTGCGCC
TCCAAATAATGGCCCGAACACAGCCAGTCGTGACTGGCCCGTACCTGCGCCGTCACGGTG
TCTTCCTGAGAGACAGGCAGGCGGCGCGGTTGCCCATTTACTCACCGTCTTGTTCAAATT
GACACAAACATCAGAGGTCGTGAAACAGCATTTGAGGCTTTTAGCCGGCCTTGGTAAAAG
GGAAACTGAAGCTCCATTACGAGTCCGGATAGCAACTTTATAGGGCTGTAGACATAAAAA
```

```
GGGGATTTTAGCTAAAACAGTGGCATTATTTATAACATCATTTATGTTTATGGTGGTTCA
GCCTCGAAACACAACCACGGCGGGATGTAGCTGCACATCTGACCCCAACCCGCTGCTGGA
GGTTCAGATAAGTCACAAGGTTTACCTGCACAACACCTGTTGCAGGTTCACGGAAAACAC
GACCGCCACTTTTTATGAATAAATATCGAAAAGTCACAGTTTGTAGCAACTGCTGTCATC
AAGGTGAAGCCTGANNNNNNNNNNNNNNNNNNNNNNNNNNNNNNNNNNNNNNNNNNNNNT
TTTAACCTTATTTGTTCAAATGAAACAAAGCACCTGCACCAATGGGATACTTAAGACTTA
TTTGTAATATTTTGTAAAATACGAATTCTACTACGACTATTAAATAATTTAACAATAACA
CCTTAGAAATAAAATGATTAATGACACAACGGGAAACTACATAATAATAATTATACAAAT
AATATCAGNNNNNNNNNNNNNNNNNNNNNNNNNNNNNNNNNNNNNNNNNNNNNNNNNNNN
NNNNNNNNAACCATATTTTACTTCAATGAACTAACCTAGAAAATTACAAAAAATCTAAAA
GTAAATTCTCTCATCGGTACACGCACGTATACACGACGTGCACGCGCGCCAGCGGACAAT
AAACAGGCCGGTGAGCGCCACATGCACCTTGCACACTTCCCAGTTCGGCGCGTCCGTGTC
CTGTAAGGTGTGACGTGGCCGCGTCTCTGCAGCCAATCACTGCCAACTTGAGGCTGATGC
TGAAGGGAAAGTAACTGGTAAGAGAAGCGCATTTTTTTCCCTCACTCTCATTGACATTTC
AACTCCACGACGGATCCGACGCAGATAGATTTTTAATCGGGACCTATAATTTTGTGTCGC
TGACGCGCGGAGGGAAAGACCCGCGGAAGGAGAAAGCGGCTTTACGCGAGCAGGATACCC
AAACTGACATTCATCCGGTGAGTGTGCAGCACTTTATTGCACTTTACATTTGGTTCCCTT
CTGAACAACCACCACTTATATAGGTTAAAACAAATCTGAAGACCGATGTCCACTCATCAA
ATGTGCGCCCCACTGGCTCCGATGACGTTTCAGCCTTTACAAAGTGTGCAGACAGGTATT
AAAACAAACACTCGGTCTCTCCGCCCAACTGATGACTGATGTGATGGATCAATAATGGTA
AAATCACTTGGTAAGTCCTTGTTCACTGTAGCCGGGAGGTGAACACCCACTCTTGTTGTC
ATTTATCACGCGCAAATACAGCGTGTAATTAGCTGTTCTCGCCGTAGAATCAAGCGCGCG
TCACTCTTGAAAAACGGCTTCGTCCTGCACAGAGAACCGAATGTGTTCAATCCCGCGTTC
GATCCATCAACTTCCATCAGGTTATTGAAGCCCAGCAGCGCTCCAGCGCGCGCGGCTGAG
CTCTTGGACGCAAATGTTGGGTTCGACAAGTTTGTGGCGTCAGAACTTGGTCAGAATCTG
GAGAGACGACGCGCGTGATAAAGCTTTTAAAAAATGCTCCTCGGGTTTTGACTCCACGGG
TTTGCACGGTTGTGTGCGTGTGTTTGTGCGCGTTATTTTAAAATACATTTGTTA
```

```
>amplified_fugu_segment
TGACACAACGGGAAACTACATAATAATAATTATACAAATAATATCAGNNNNNNNNNNNNN
NNNNNNNNNNNNNNNNNNNNNNNNNNNNNNNNNNNNNNNNNNNNNNNAACCATATTTTAC
TTCAATGAACTAACCTAGAAAATTACAAAAAATCTAAAAGTAAATTCTCTCATCGGTACA
CGCACGTATACACGACGTGCACGCGCGCCAGCGGACAATAAACAGGCCGGTGAGCGCCAC
ATGCACCTTGCACACTTCCCAGTTCG
```

```
>rCNE_mouse_sequence_835990
GCACAGCAGAGCCGGTTAGCCCAAGTGCACAGCTCTTCACCAAGCGCAAAGGAAAACAAT
GAAACTTTCCCTGTTTAATGATAGAAATTACATTAAAAGTGGTGGAAATGCCCTAATTAA
AAATGTACCACTTAAATGATATGCCAAGGACTCAGCTGCAGCTTAGCATATTTAGCTAGT
TAGTGAACTCTCCGCCGTCTCTCTTTTAAAATTACACGTTAAAAATTTAAAAGAAATCTT
ACTTTTCTCTGGTGCAACACATTACAAAGAATGGACAGTCCTTTTATCTAAATATAAAAT
TCCATTTTCAGCAATTATAGCCTGTTCTTGGTGATGATATAATTACAGCTGTGCTCGTAA
TAGGTGTCAAGGCGAAACACACTCACACAATGGTTGAATAAAACTGCAGAACAATGCAAG
CACTTCTAAATTCACAACCTTTAAAATGAAATTGACTGTGCAGAATTCAAATAAAAACTA
GATAAATATTTTTTTAAAAAGATTACAAGCTTGGTTTGGTTTCTTAATAGCATTTTCTAC
AAACCTATCAACATCTAATTGATTAGATAGGAATTACTGATTATAGATCAGCTGAAATCG
TGCACATCACTCTATTTTCCCAACACAGCCATAGGTAAATA
```

```
>SCE_mouse_sequence_836071
AAAATTACACGTTAAAAATTTAAAAGAAATCTTACTTTTC
```

FUGU HITS IN THE CONTEST OF MAMMALIAN rCNE

CLUSTAL W (1.83) multiple sequence alignment

```
ENSRNOG00000004410      GCACGGC-AGAGCCAGTTAGCCCAAGTGCAGAGCGC-TTCACCCAGC-GCAAAGGAAAAC
ENSMUSG00000027168      GCACAGC-AGAGCCGGTTAGCCCAAGTGCACAGCTC-TTCACCAAGC-GCAAAGGAAAAC
ENSG00000007372         GCAAGCCGGGAGCCAGCTAGCCCAAGTGCACATCTTGCCCTCCAGGCAGCAAGGGAAAAC
ENSCAFG00000007491      GCAAGGCGAGATACAGCTAGCCCAAGTGTGCATCTTGCCCTCCAAGCAGCAAGGGAAAAC
SINFRUG00000121553      ------------------------------------------------------------

ENSRNOG00000004410      AATGAAACTTTCCCA--GTTTAATGATAGAAATTACATTAAAAGTGGTGGAAATGCCCTA
ENSMUSG00000027168      AATGAAACTTTCCCT--GTTTAATGATAGAAATTACATTAAAAGTGGTGGAAATGCCCTA
ENSG00000007372         CATAAAACTTTCCCCCTGTATAATGATAGAAGTTACATTCAAAGTGGTGGAAATGCCCTA
```

```
ENSCAFG00000007491      AATAAAACTTTCCCC-TGTTTAATGATAGAAATTACATTAAAAGTGGTGGAAATGCCCTA
SINFRUG00000121553      ------------------------------------------------------------


ENSRNOG00000004410      ATTAAAAATGTACCACTTAAATGATATGCCAAGGACTCAGCTGCAGCTTA-----TTTAG
ENSMUSG00000027168      ATTAAAAATGTACCACTTAAATGATATGCCAAGGACTCAGCTGCAGCTTAGCATATTTAG
ENSG00000007372         ATTAAAAATGTACCACTTAAATGATATGCCAAAGATTCAGCTGCAGCATAGAATATTTAG
ENSCAFG00000007491      ATTAAAAATGTACCACTTAAATGATATGCCAAGGATTCAGCTGCAGCATAGCATATTTAG
SINFRUG00000121553      ------------------------------------------------------------


ENSRNOG00000004410      CTAGTTAGTGAACTCTCCACCATCTCTTTCTTAAAATTACACGTTAAAAATTTAAAAGAA
ENSMUSG00000027168      CTAGTTAGTGAACTCTCCGCCGTCTCTCTTTTAAAATTACACGTTAAAAATTTAAAAGAA
ENSG00000007372         CTAGTTAGTGAACTCTCTACTATTTCTTTTTTAAAATTACACGTTAAAAATTTAAAAGAA
ENSCAFG00000007491      CTAGTTAGTGAACTCTCTACTATTTCTTTTTTAAAATTACACGTTAAAAATTTAAAAGAA
SINFRUG00000121553      --------------------------------AAAATTACA----AAAAATCTAAAAGTA
                                                        *********    ****** ****** *

ENSRNOG00000004410      ATCTTACTTTTCTCTGGTGCAACACATTACAAAGAATGGACAGTCCTTTTATCTAAATAT
ENSMUSG00000027168      ATCTTACTTTTCTCTGGTGCAACACATTACAAAGAATGGACAGTCCTTTTATCTAAATAT
ENSG00000007372         ATCTTACTTTTCTCTGGTGCAACACATTACAAAGAATGGACAGTCCTTTTATCTAAATAT
ENSCAFG00000007491      ATCTTACTTTTCTCTGGTGCAACACATTACAAAGAATGGACAGTCCTTTTATCTAAATAT
SINFRUG00000121553      AATTCTCTCATC------------------------------------------------
                        *  *  **  **

ENSRNOG00000004410      AAAATTCCATTTTCAGCAATTATAGCCTGTTCTTGGTGATGATATAATTACAGCTGTGCT
ENSMUSG00000027168      AAAATTCCATTTTCAGCAATTATAGCCTGTTCTTGGTGATGATATAATTACAGCTGTGCT
ENSG00000007372         AAAATTCCATTTTCAGCAATTATAGCCTGTTCTTGGTGATGATATAATTACAGCTGTGCT
ENSCAFG00000007491      AAAATTCCATTTTCAGCAATTATAGCCTGTTCTTGGTGATGATATAATTACAGCTGTGCT
SINFRUG00000121553      ------------------------------------------------------------


ENSRNOG00000004410      CGTAATAGGTGTCAAGGCGAAACACACTCACACAATGGTTGAATAAAACTGCAGAACAAT
ENSMUSG00000027168      CGTAATAGGTGTCAAGGCGAAACACACTCACACAATGGTTGAATAAAACTGCAGAACAAT
ENSG00000007372         CGTAATAGGTGTCAAGGCGAAGCGCACTCATACAATAGTTGAATAAAACTGCAGAACAAT
ENSCAFG00000007491      CGTAATAGGTGTCAAGGCAAAGCGCACTCATACAATAGTTGAATAAAACTGCAGAACAAT
SINFRUG00000121553      ------------------------------------------------------------


ENSRNOG00000004410      GCAAGCACTTCTAAATTCACAACCTTTAAAATGAAATTGACTGTGCAGAATTCAAATAAA
ENSMUSG00000027168      GCAAGCACTTCTAAATTCACAACCTTTAAAATGAAATTGACTGTGCAGAATTCAAATAAA
ENSG00000007372         GCGAGCACTTCTAAATTCACAACCTTTAAA-TGAAATTGACTGTGCAGAATTCAAATAAA
ENSCAFG00000007491      GCGAGCATTTCTAAATTCACAACCTTTAAAATGAAATTGACTGTGCAGAATTCAAATAAA
SINFRUG00000121553      ------------------------------------------------------------


ENSRNOG00000004410      AACTAGATAAATATTTTTTTAAAAAGATTACAAGCTTGGTTTGGTTTCTTAATAGCATTT
ENSMUSG00000027168      AACTAGATAAATATTTTTTTAAAAAGATTACAAGCTTGGTTTGGTTTCTTAATAGCATTT
ENSG00000007372         AACTAGATAAATATTTTTTAAAAAAGATTACAAGCTTGGTTTGGTTTCTTAATAGCATTT
ENSCAFG00000007491      AACTAGATAAATATTTTTTTAAAAAGATTACAAGCTTGGTTTGGTTTCTTAATAGCATTT
SINFRUG00000121553      ------------------------------------------------------------


ENSRNOG00000004410      TCTACAAACCTATCAACATCTAATTGATTAGATAGGAATTACTGATTATAGATCAGCTGA
ENSMUSG00000027168      TCTACAAACCTATCAACATCTAATTGATTAGATAGGAATTACTGATTATAGATCAGCTGA
ENSG00000007372         TCTACAAACCTATCAACATCTAATTGATTAGATAGGAATTACTGATTATAGATCAACTGA
ENSCAFG00000007491      TCTACAAACCTATCAACATCTAATTGATTAGATAGGAATTACTGATTATGGATCAGCTGA
SINFRUG00000121553      ------------------------------------------------------------


ENSRNOG00000004410      AATCTTGCACATCACTCT---ATTTTCCCAACACAGACATAGGTAAATA
ENSMUSG00000027168      AATCGTGCACATCACTCT---ATTTTCCCAACACAGCCATAGGTAAATA
ENSG00000007372         AATCTTGAACATCACTCTTCTATTTTCCCAACACAGCCATAGGTAAAGA
ENSCAFG00000007491      AATCTTGAACATCATTCTTCTATTTTCCCAACAGAGCTATAGGTAAATA
SINFRUG00000121553      -------------------------------------------------
```

Fragment ETS1_MOUSE_8744

Representation of conserved regions within their genic context

Within the mouse context is shown the rCNE from which the tested SCE is derived (red diamond)

Within the fugu context all CHAOS hits obtained from the mouse rCNE are shown (green diamonds, in red the one tested)

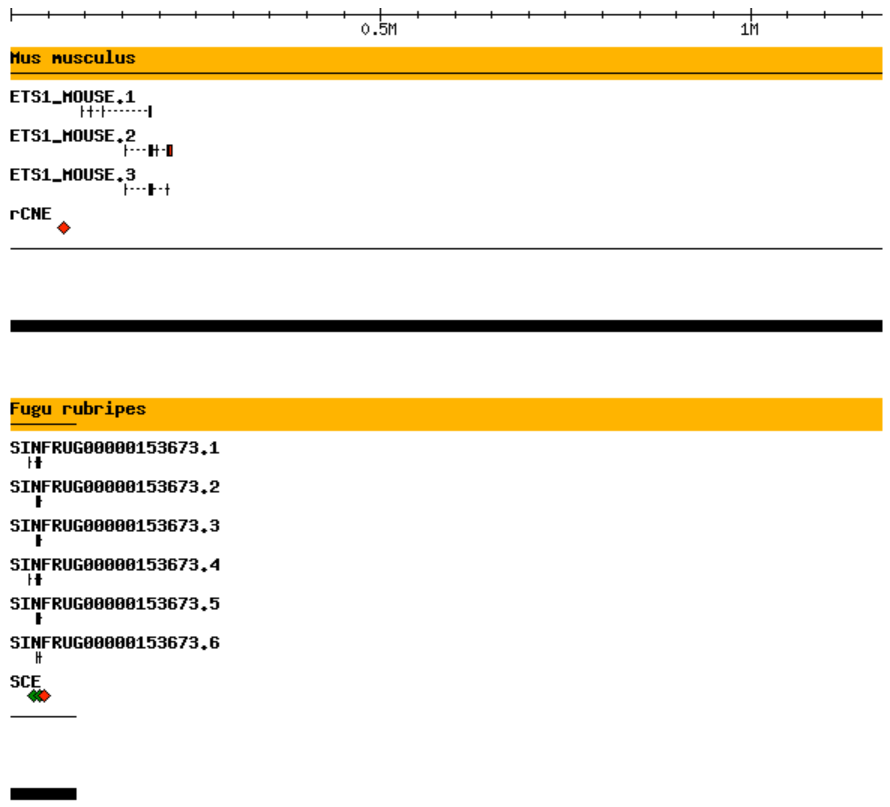

Conserved regions shown within 2000bp context

The mouse context contains:

-rCNEs green boxes, in red the one considered for this test

-SCEs yellow boxes, in red the one considered for this test

-CHAOS hits blue boxes, in red the one considered for this test

The fugu context contains:

-the fragment amplified in this test

-CHAOS hits blue boxes, in red the one considered for this test

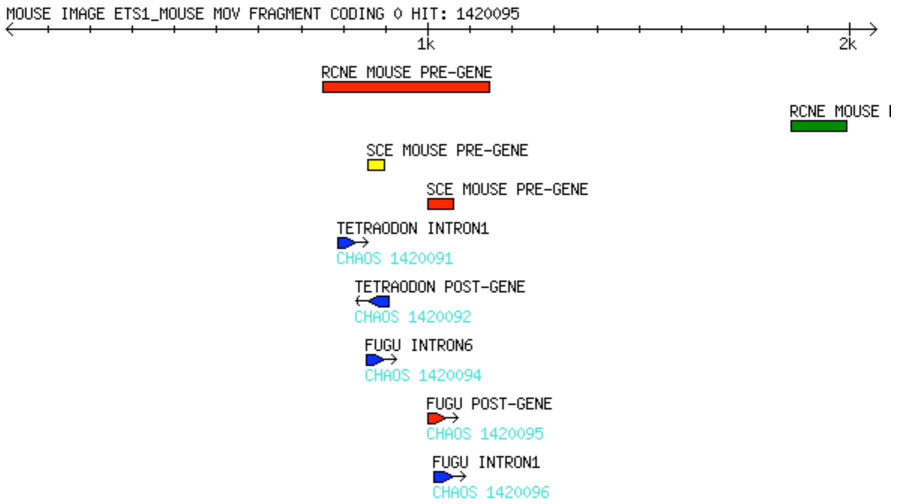

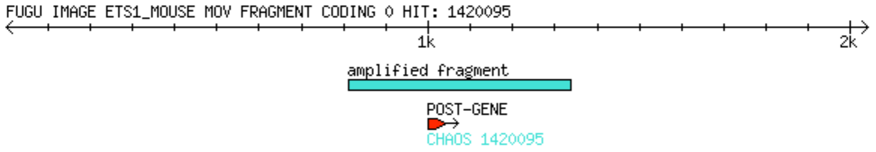

SEQUENCES

```
>fugu_fragment_for_primer_design_8744
TTTTTCCGCTCTTCTCCACTCCGCTGAGATAAAGCCTCCGCTGATTTAACGCGGTGTTTG
AAAGACAAACACAGAAAAGAGGAAGTCTTTCAGAACACCAGCCTGTCACACCTGGCAGTC
GCACAGAAACTATTTCTGACGAAAAGCATCATCCTGTCACCAGCAGCTCTCACAGCTCTC
TGACACTGGAATGTAGGCGCTTTGTCAAATTTCTAATCTAAATATTTGTCTAAAGGCTCT
CACCTAGATATATCTGTCTCCTGCATGGAGCCATAACCGAGGGAGAGGGACCGGGGTGTC
TCACCGTCCAACGTGTTCAGATAATGAGAATTTTTCTGAAGCTATTTCATGTGCACTGTA
TGGCTGTTAGTTAGAATATATTCACCATTTCTTAAGTTTCCAGACAATCTGGGAAGCCAT
TGTTGAATGGGTTGGATAATTTGTGCCCTTGAGGTGTGATTGAATAAGGTTTTTGGATTT
CACAATACGTGTTGTAAGAAATACCCACATGGGGAAGTACAGAAGAACACCAGGGCAGGT
AGGTTGCCAGAGGAAACCTCAGTGAAAGTATACAAAGTGCGCCCTAATGGCTAGAGCGCT
TGTCACTTTTTGTGTTGTGCATTTTTGGATTAACGACGTTGACTTTGGATGAAAGGAAGA
TGCTGGTCCACAAGAAATTTGCGTCTTTTTTTAGCCTGAGAAGCCATGTGACGTTTAGGG
CCAACTCAATGGTTCCATTACGCAGAGAAGCTGTGAAATATGACCAGCGTCCCAGTGGAC
CCTGAAGGCCAAGCTTAAATCACGTCGTCCCGGGGATGTGTTCTCCATGCTTTCCCCATG
TCTCATTGTCTGTCATGGTTGACTTGAGCCTCTGTGAACACAGAGATGTCCAGGGAGTTT
CATCCAGCATGTTGTTTCAAAGATGACCCTTCTCATGGACAGAGAGACTGAAGACAAGCA
TCCCTGACGCGATCAATGGACTTGGAAGAATGTACGCTAACATAAACAGAAAAAGTCCGT
CGAGGACCCTGATTTTGTGGCCTATTCACTGTGTTAATGACTGAGCAAAAAAAGGGGTCG
CAGTACTTAACTCACATAAGTGGATAAGACACAATGGAACTCTGACTCCCAGAAATCAGT
AAGCACACAGATTAACGTTGCCTAATTTCAAACTTTTACATTGATGATTGAAGAAAATTT
TGTGCTAAAAATAATAGAAAGTGGTTTGACGAACTTATAATGGCTGCCAGATAGAAGTGG
TTCAAGGTTGCTCACTTCGCCCGTCCTTCTCCTGTTACGTCTCCTGCTCCTCCGCCCTCT
CCGTCATTGTATTGTTGTGTCTGAGATGTGTTGAACCACATGTCTTGACAGGTGTGCTGA
CTGTATTATCCAGACTTATGGAGGGCAGATTCAGGCCATTGCCTGTTTTGCCTGGTAATT
TGCATTTTTATTGCAGCTGTCGCATCCTCAGTTAATACACCATTGACCCCCATAGCGGAC
CCCGGACCTAACGGCAGCACCCTGCTATCTCACCTGAAATGAGAGTGCCCACTGGTCCCT
GGATCAGCAATGGCTGAAACCCAGGCACCAGTTGACCTGGCATTCTGGGACGGAAATGGA
GTGGCCTCTCATGTTAATGTGGACTTTCCATTAGGAAAGATGATTGATTTGTGCTGTGCT
TTGGCTGTGAATAGGATATAGACTGGCACAATAAAGTGAAGAAATTGTTCTTTGTGTGCA
GGTGAAGCCGTGTGTGAACCAGACTGTGACTAAATCAGAGTGCTGATGATAGGGCAAGAG
GGAAGGTGAGAGGAGAGACGGAGCAGCAGGGGGGGTTAGGGAGGGATAAAACAAGGGCTG
```

```
AGAAGTGGAATGGCAGGTGCAATAGATGAAGGTTAGGAGACGAAACAGGACAAAGCTTTC
TTAAAGAAAGAGAGTGACCATGAGTAATGGGAGATGGAAACAATGAGGAGCCAGCTCTAT
TCATTTCAGCCCTTTTAAAAAATTAGATGAAAGGTGATGAAGCAGACAGAGCTTGTCAGT
G
```

```
>amplified_fugu_segment
GGGATGTGTTCTCCATGCTTTCCCCATGTCTCATTGTCTGTCATGGTTGACTTGAGCCTC
TGTGAACACAGAGATGTCCAGGGAGTTTCATCCAGCATGTTGTTTCAAAGATGACCCTTC
TCATGGACAGAGAGACTGAAGACAAGCATCCCTGACGCGATCAATGGACTTGGAAGAATG
TACGCTAACATAAACAGAAAAAGTCCGTCGAGGACCCTGATTTTGTGGCCTATTCACTGT
GTTAATGACTGAGCAAAAAAAGGGGTCGCAGTACTTAACTCACATAAGTGGATAAGACAC
AATGGAACTCTGACTCCCAGAAATCAGTAAGCACACAGATTAACGTTGCCTAATTTCAAA
CTTTTACATTGATGATTGAAGAAAATTTTGTGCTAAAAATAATAGAAAGTGGTTTGACGA
ACTTATAATGGCTGCCAGATAGAAGTGGTTCAAGGTTGCTCACTTCGCCCGTCCTTCTCC
TGTTACGTCTCCTGCTCCTCCGCCCTCTCCGTCATTGTATTGT
```

```
>rCNE_mouse_sequence_1261874
GCCCCAAGCTGCAGGTGGTAATTTGCATAGGGCAGTGTCTCCTCTCCCAGGAGGTTAGGC
CTCTCTGAGTTTCTTTTCTGTACAAGGGGAGTCGAACAGTGTCTTTAAAAAGAAAGAAAA
ACAAGACGGGTGACTTTGCTTGGACCTGGGTAGTAAGGTGTTCCAAAGTCTCTGTGGAAG
TAGCTATTTCTGGCCCCATTGTCGTCTGGGTTACAGAGGAAGGCCTCCTGGCAATTCTTT
GCTTGAAAGACAGAAACAGAACATTGTTTCTGACGGCTTTGATTTAATGGCCAAAAACAG
TTATAGGAAACTGTTGAGCAGATGTTCTGAGAAACTCAGAAGAGACTGGGTCTTTGAGAA
GGGCTATTTTTAGACAAATGCTTCCTACCATTGG
```

```
>SCE_mouse_sequence_1262086
ACAGAAACAGAACATTGTTTCTGACGGCTTTGATTTAATGGCCAAAAACAGTTATAGGAA
AC
```

FUGU HITS IN THE CONTEST OF MAMMALIAN rCNE

```
CLUSTAL W (1.83) multiple sequence alignment


ENSMUSG00000032035      GCCCCAA-GCTGCAGGTGGTAATTTGCATAGGGCAGTGTCTCCTCTCCCAGGAGGTTAGG
ENSRNOG00000008941      GCCCCAG-GCTGCAGGTGGTAATTTGCATAGGGCAGCATCTCCTCTCCCAAGAGGTTAGG
ENSG00000134954         GCCCCACCACTGCAGGTGGTAATTTGCATGGGGAGCAGAGCGTCTCCCAGGAGGCTAGG
ENSCAFG00000010304      ---GCGGCCCTGCAGGTGCGGATTTGCATGGGGAGGCTGGCCTCTCCCGTGAGGTTTTG
SINFRUG00000153673      ------------------------------------------------------------

ENSMUSG00000032035      CCTCTCTGAGTTTCTTTTCTGTACAAGGGGAGTCGAACAGTGTCTTTAAAAAGAAAGAAA
ENSRNOG00000008941      CCTATCTAAGTTTCTTTTCTGTACAAGGGGAGTCGAACAGTGTCTTTAAAAAGAAAGAAA
ENSG00000134954         CCTATCTAAGTTTCTCTTCTGTACTTGGG-AGACGAACAGCCACTTTAAAA-GAGAGAAA
ENSCAFG00000010304      CCTATCCAAGTTTCTTTTCTGCTCTTGGG-AGATGAACAGCCTCTTTAAAAAGAAAGAAA
SINFRUG00000153673      ------------------------------------------------------------

ENSMUSG00000032035      AACAAGAC--GGGTGACTTTG---CTTGGACCTGGG--TAGTAAGGTGTTCCAAAGTCTC
ENSRNOG00000008941      AACAAGACAGGGGTAGCTCTG---CTTGGACCTGGG--TAGTGAGGTGTTCCAAAGTCTC
ENSG00000134954         AACAAGACACTAGTAACTCTGTG-CTTGGACCCGGG--CCTTTAGGTGTTTCAAAGCCTC
ENSCAFG00000010304      AACAAGACACTAGTAACCCCGCTTCTTGGCCCCGGGGGCCTCAAGGTGTTTCAAAGCCTC
SINFRUG00000153673      ------------------------------------------------------------

ENSMUSG00000032035      TGTGGAAGTAGCTATTTCTGGCCCCATTGTCGTCTGGGTTACAGAGGAAGGCCTCCTGGC
ENSRNOG00000008941      TGTGGAACTGGCTATTTCTGGCCCCATTGTCGTCTGGGTTACAGAGGAAGGCCTCCTGGC
ENSG00000134954         CC-----GTGGCTATTTCTGGCCCCATTGTCGTCTGGGTCTGAGAGGAAGGCCTGATGGC
ENSCAFG00000010304      CC-----GTGGCTATTTCGGGCCCCATTGTCGTCGGGGGCTGAGAGGAAGGTCTGATGGC
SINFRUG00000153673      ------------------------------------------------------------

ENSMUSG00000032035      AATTCTTTGCTTGAAAGACAGAAACAGAACATTGTTTCTGACGGCTTTGATTTAATGGCC
ENSRNOG00000008941      AATTCTTTGCTTGAAAGACAGAAACAGAACATTGTTTCTGACGGCTTTGATTTAATGGCC
ENSG00000134954         GATTCTGTGCTTTAAAGGCAGAAACAGAACATTGTTTTTGCCAGCTTTGATTTAATGGCC
ENSCAFG00000010304      AATTCTTTGCTTTAAAGGCAGAAACAGAACATTGTTTCTCCGGGCTTTGATTTAATGGCC
```

```
SINFRUG00000153673      -----------------ACATAAACAGAAAAAGTCCGTCGAGGACCCTGATTTTGTGGCC
                                          ** ******** *            *  ******  *****

ENSMUSG00000032035      AAAAACAGTTATAGGAAACTGTTGAGCAGATGTTCTGAGAAACTCAGAAGAGACTGGGTC
ENSRNOG00000008941      AAAAACAGTTATAGGAAACTGTTGAGCAGATGTTCTGTGAAACTCAGAAAAGACTGGGTC
ENSG00000134954         AAAAACAGTTATAGGAAACTGTTCAGCAGATGTTTTATGAAACTCAGAAAAGATTGGGTC
ENSCAFG00000010304      AAAAACAGTTATAGGAAACTGTTCAGCAGATGTTTTATGAAACTCAGAAAAGATTGGGTC
SINFRUG00000153673      ------------------------------------------------------------


ENSMUSG00000032035      TTTGAGAAGGGCTATTTTTAGACAAATGCTTCCTACCATTGG
ENSRNOG00000008941      TTTGAGAAGGGCTATTTTTAGACAAATGCTTCCTACCATTGG
ENSG00000134954         TTTTAGAAGGGCTATTTTTAGACAAATGCTTCCTACCATTGG
ENSCAFG00000010304      TTTTAGAAGGGCTATTTTTAGACAAATGCTTCCTACCATTGG
SINFRUG00000153673      ------------------------------------------
```

Fragment ETS1_MOUSE_8745

Representation of conserved regions within their genic context

Within the mouse context is shown the rCNE from which the tested SCE is derived (red diamond)

Within the fugu context all CHAOS hits obtained from the mouse rCNE are shown (green diamonds, in red the one tested)

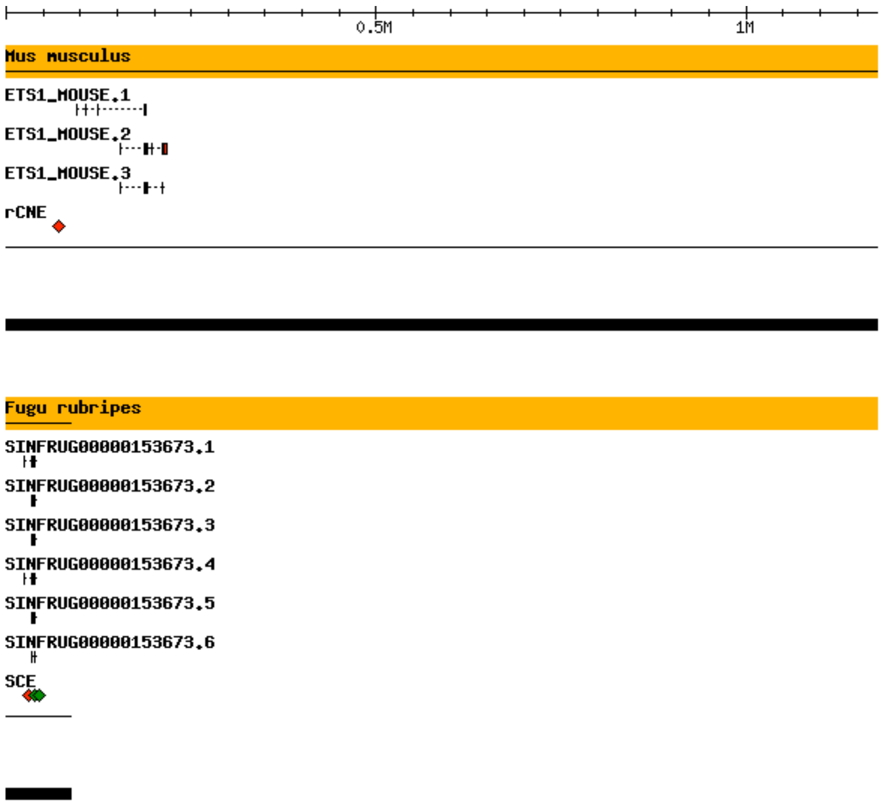

Conserved regions shown within 2000bp context

The mouse context contains:

-rCNEs green boxes, in red the one considered for this test

-SCEs yellow boxes, in red the one considered for this test

-CHAOS hits blue boxes, in red the one considered for this test

The fugu context contains:

-the fragment amplified in this test

-CHAOS hits blue boxes, in red the one considered for this test

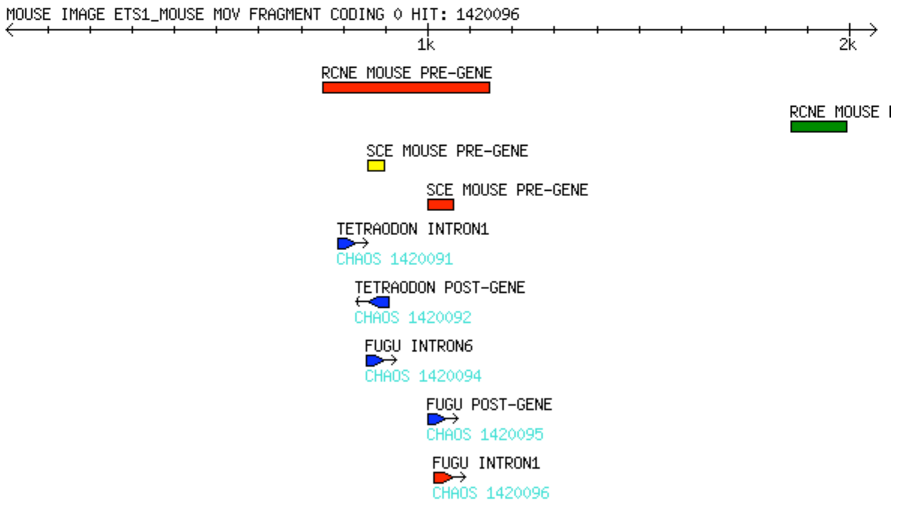

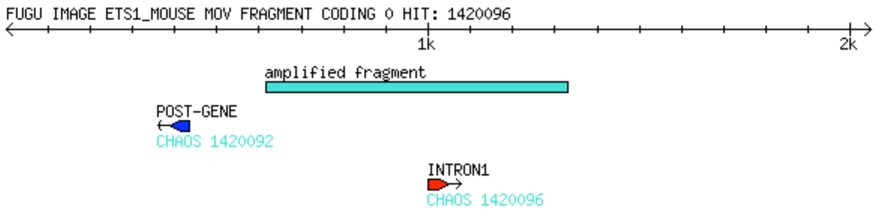

SEQUENCES

>fugu_fragment_for_primer_design_8745
GTAGACTATAAATGGACTTCAAACATGATGCACGCCCATTTGTGCATGTAATATAATTGA
AGACAGCGTTTGCATCACTAATAACCCGCGCATGCATCAAGAATGCCGGTCATTTGACAA
ATCGCTGCTACATTTTCCATGTGTGACTGTTTTCCTGTAGGGTCCCATGCTGGCATCATA
AATAGGGTTGTGGAATGTATCAGTTTGGACTAAAAAATGGGACAAGCACTGTTTGGTGGA
AAAAATAGAACACTAAGATTAAACAGTTTGTCAGATATGTTGTGTGTTTGGTTAAATGCC
TAAACAGTCCCCCCCAGAGCATCCTCTGTCTGTTTACAGCAATGCTGGCCTGCCAGCCTG
GACGCAGCCATGCTAACAACACCTGCACATCCTGAAGTTCTCATTTCTCTGCCACTGTTG
TCGTCAGAGACATAACATGTCAGTCTGATACTATTGGACATGTAGTGCTTCTATCATAGT
TTCCACCTGCAGACATGATGGGGAGTTGATCTTTTTGACTCAGTTTCAACCTAATGGAGA
ATTTGTGGGAGGTTCGGTAGANNNNNNNNNNCTATCCGAAGACAGGAAACCTAAAAGAATC
CCATCTAGTTTACACCGATTCTCCCATCAGTTCAACATGTTCAGAATAAAAGTCACAACC

```
TATGTTGTTTCTTCGAGTTTATGNNNNNNNNNNGTTACATTTACAATTTTGCATGCAGAC
ATGCTGGAAAATTTGGCTGCTGACGTTTNNNNNNNNNNNNTAAGCAGAGCAAAACTGTTC
AAATAGCTTCTCTTCAGCTTTGAGAATCGATGTTTTGCAGCTGTGCGTCCGATTGCAGAG
GTAATGCCATATCAAGTGGTGAGACCTTAGTCCTGATGCACTTTTACATCATAAGCTAAT
GTGATTTCTTTGATGGAGGAAGTGGAAAGTAAGAAAGGCAGCTATGGGGTATTAGTCTCA
CTTACAGTCAGCTTCAGTAGCCAGTATGTCTGTGCTGTGATCATCCTGGACCACTCTTGT
TTCATGGCCAAAGACAGATGTCAGCAGCAGGCCTCATCCTGCACAGCTGGNNNNNNNNNN
NNAGCCGCCTCGGGCTTGTTCTTCAGCGGTGCAGCCAGCAGCCAGTCGTGCCAACCTAAG
AATAGATCCTATGTGTCAGCATCATCCGCGATGGCAGGATTTAAGTTTAACACCCCTTTG
ACATGAGAGAAGACGCCATGTCTGTTCCTGGTTGTCACTGAAATGAAAAGTCTAAATTGC
CACGGTTGAACGAGCCGCCCTCTCACATCCCAGCCGCCACCTCACCCTGCTCCTGTTGCC
CCACTACAGCGGGGTTGTATCGCCCTGTGGTTCTCAATGGCCCCCTTCATGGAAGTCAGT
TTTATGACCCTGTGTTCTCCAACCAGGGGTCCAGATATAGATCTCCATCAAAGAGCCTCT
CACATACCGGCGTCACCTCTGCTCTGCTGCGGGCGCTCTAGCTGGCCTCAAAGAGACAAT
TATATGTCTGTAAACCCTTTTTTGCGAGTCTGCTGATATGTACCTGTTGACTTGTGGGGT
TATTTAGGAAGGGTGTAAGACCTCGGCGACAAGTCGGAAATTATTTGACCCTCAACTGAA
CCCATCACTTACTTCCCTAGGGATGGCAACTTAGCCACAGGAAATTGACTCTTTGGCTCT
CGNNNNNNNNCAACCCCCCAAAGAAACAGGTACATTCAGTAGCAAGAGGGCGCACATCGC
TGCCTGCCTTTCTTTCTCTTTTTCTTTCCAGAACTCCCAAACGTTTGTGCAGAAATGGTT
CNNNNNNNNNNNNNNNNNNNNNNNNNNNATGTCAACTTCCCGATGATTACAGATGTGGAA
TGAATGCGGTTTAAATAACCTTGTATCTTGGAGAAATGAAGTAGATGGGGTCAGAAATGC
TGGCAACAGAGACAGGAACCCATGATAGGGGTATTGTTGAGACCACCCTGGCTCTGACTA
AAACTCTAAAGGGCTATTTATAGCTGTGTTGTTTGGAAGCATACTGTCCTTTATTTTTTG
GTCTGGT
```

```
>amplified_fugu_segment
CCGATTCTCCCATCAGTTCAACATGTTCAGAATAAAAGTCACAACCTATGTTGTTTCTTC
GAGTTTATGNNNNNNNNNNGTTACATTTACAATTTTGCATGCAGACATGCTGGAAAATTT
GGCTGCTGACGTTTNNNNNNNNNNNNTAAGCAGAGCAAAACTGTTCAAATAGCTTCTCTT
CAGCTTTGAGAATCGATGTTTTGCAGCTGTGCGTCCGATTGCAGAGGTAATGCCATATCA
AGTGGTGAGACCTTAGTCCTGATGCACTTTTACATCATAAGCTAATGTGATTTCTTTGAT
GGAGGAAGTGGAAAGTAAGAAAGGCAGCTATGGGGTATTAGTCTCACTTACAGTCAGCTT
CAGTAGCCAGTATGTCTGTGCTGTGATCATCCTGGACCACTCTTGTTTCATGGCCAAAGA
CAGATGTCAGCAGCAGGCCTCATCCTGCACAGCTGGNNNNNNNNNNNNAGCCGCCTCGGG
CTTGTTCTTCAGCGGTGCAGCCAGCAGCCAGTCGTGCCAACCTAAGAATAGATCCTATGT
GTCAGCATCATCCGCGATGGCAGGATTTAAGTTTAACACCCCTTTGACATGAGAGAAGAC
GCCATGTCTGTTCCTGGTTGTCACTGAAATGAAAAGTCTAAATTGCCACGGTTGAACGAG
CCGCCCTCTCACATCCCAGCCGCCACCTCACCCTGCTCCTGTTGCCCCACTACAGC
```

```
>rCNE_mouse_sequence_1261874
GCCCCAAGCTGCAGGTGGTAATTTGCATAGGGCAGTGTCTCCTCTCCCAGGAGGTTAGGC
CTCTCTGAGTTTCTTTTCTGTACAAGGGGAGTCGAACAGTGTCTTTAAAAAGAAAGAAAA
ACAAGACGGGTGACTTTGCTTGGACCTGGGTAGTAAGGTGTTCCAAAGTCTCTGTGGAAG
TAGCTATTTCTGGCCCCATTGTCGTCTGGGTTACAGAGGAAGGCCTCCTGGCAATTCTTT
GCTTGAAAGACAGAAACAGAACATTGTTTCTGACGGCTTTGATTTAATGGCCAAAAACAG
TTATAGGAAACTGTTGAGCAGATGTTCTGAGAAACTCAGAAGAGACTGGGTCTTTGAGAA
GGGCTATTTTTAGACAAATGCTTCCTACCATTGG
```

```
>SCE_mouse_sequence_1262086
ACAGAAACAGAACATTGTTTCTGACGGCTTTGATTTAATGGCCAAAAACAGTTATAGGAA
AC
```

FUGU HITS IN THE CONTEST OF MAMMALIAN rCNE

CLUSTAL W (1.83) multiple sequence alignment

```
ENSG00000134954         GCCCCACCACTGCAGGTGGTAATTTGCATGGGGGAGCAGAGCGTCTCCCAGGAGGCTAGG
ENSCAFG00000010304      ---GCGGCCCTGCAGGTGCGGATTTGCATGGGGGAGGCTGGCCTCTCCCGTGAGGTTTTG
ENSMUSG00000032035      -GCCCCAAGCTGCAGGTGGTAATTTGCATAGGGCAGTGTCTCCTCTCCCAGGAGGTTAGG
ENSRNOG00000008941      -GCCCCAGGCTGCAGGTGGTAATTTGCATAGGGCAGCATCTCCTCTCCCAAGAGGTTAGG
SINFRUG00000153673      ------------------------------------------------------------

ENSG00000134954         CCTATCTAAGTTTCTCTTCTGTACTTGGG-AGACGAACAGCCACTTTAAAA-GAGAGAAA
ENSCAFG00000010304      CCTATCCAAGTTTCTTTTCTGCTCTTGGG-AGATGAACAGCCTCTTTAAAAAGAAAGAAA
```

```
ENSMUSG00000032035      CCTCTCTGAGTTTCTTTTCTGTACAAGGGGAGTCGAACAGTGTCTTTAAAAAGAAAGAAA
ENSRNOG00000008941      CCTATCTAAGTTTCTTTTCTGTACAAGGGGAGTCGAACAGTGTCTTTAAAAAGAAAGAAA
SINFRUG0000153673       ------------------------------------------------------------


ENSG00000134954         AACAAGACACTAGTAACTCTG-TGCTTGGACCCGGG--CCTTTAGGTGTTTCAAAGCCTC
ENSCAFG00000010304      AACAAGACACTAGTAACCCCGCTTCTTGGCCCCGGGGGCCTCAAGGTGTTTCAAAGCCTC
ENSMUSG00000032035      AACAAGAC--GGGTGACTTTG---CTTGGACCTGGG--TAGTAAGGTGTTCCAAAGTCTC
ENSRNOG00000008941      AACAAGACAGGGGTAGCTCTG---CTTGGACCTGGG--TAGTGAGGTGTTCCAAAGTCTC
SINFRUG0000153673       ------------------------------------------------------------


ENSG00000134954         CC-----GTGGCTATTTCTGGCCCCATTGTCGTCTGGGTCTGAGAGGAAGGCCTGATGGC
ENSCAFG00000010304      CC-----GTGGCTATTTCGGGCCCCATTGTCGTCGGGGGCTGAGAGGAAGGTCTGATGGC
ENSMUSG00000032035      TGTGGAAGTAGCTATTTCTGGCCCCATTGTCGTCTGGGTTACAGAGGAAGGCCTCCTGGC
ENSRNOG00000008941      TGTGGAACTGGCTATTTCTGGCCCCATTGTCGTCTGGGTTACAGAGGAAGGCCTCCTGGC
SINFRUG0000153673       ------------------------------------------------------------


ENSG00000134954         GATTCTGTGCTTTAAAGGCAGAAACAGAACATTGTTTTTGCCAGCTTTGATTTAATGGCC
ENSCAFG00000010304      AATTCTTTGCTTTAAAGGCAGAAACAGAACATTGTTTCTCCGGGCTTTGATTTAATGGCC
ENSMUSG00000032035      AATTCTTTGCTTGAAAGACAGAAACAGAACATTGTTTCTGACGGCTTTGATTTAATGGCC
ENSRNOG00000008941      AATTCTTTGCTTGAAAGACAGAAACAGAACATTGTTTCTGACGGCTTTGATTTAATGGCC
SINFRUG0000153673       ------------------------------ATCATCCTGGACCACTCTTGTTTCATGGCC
                                                      **  *         ** *  *** ******

ENSG00000134954         AAAAACAGTTATAGGAAACTGTTCAGCAGATGTTTTATGAAACTCAGAAAAGATTGGGTC
ENSCAFG00000010304      AAAAACAGTTATAGGAAACTGTTCAGCAGATGTTTTATGAAACTCAGAAAAGATTGGGTC
ENSMUSG00000032035      AAAAACAGTTATAGGAAACTGTTGAGCAGATGTTCTGAGAAACTCAGAAGAGACTGGGTC
ENSRNOG00000008941      AAAAACAGTTATAGGAAACTGTTGAGCAGATGTTCTGTGAAACTCAGAAAAGACTGGGTC
SINFRUG0000153673       AAAGACAGATGTCAGCAGC-----------------------------------------
                        *** **** * *  * * *

ENSG00000134954         TTTTAGAAGGGCTATTTTTAGACAAATGCTTCCTACCATTGG
ENSCAFG00000010304      TTTTAGAAGGGCTATTTTTAGACAAATGCTTCCTACCATTGG
ENSMUSG00000032035      TTTGAGAAGGGCTATTTTTAGACAAATGCTTCCTACCATTGG
ENSRNOG00000008941      TTTGAGAAGGGCTATTTTTAGACAAATGCTTCCTACCATTGG
SINFRUG0000153673       ------------------------------------------
```

Fragment ETS1_MOUSE_8726

Representation of conserved regions within their genic context

Within the mouse context is shown the rCNE from which the tested SCE is derived (red diamond)

Within the fugu context all CHAOS hits obtained from the mouse rCNE are shown (green diamonds, in red the one tested)

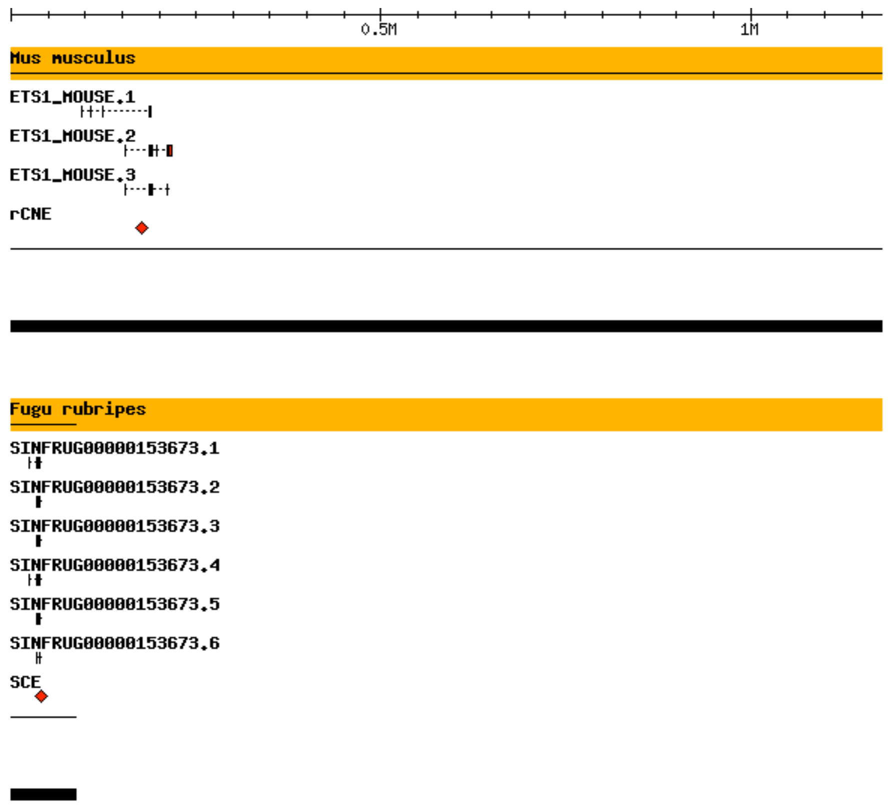

Conserved regions shown within 2000bp context

The mouse context contains:

-rCNEs green boxes, in red the one considered for this test

-SCEs yellow boxes, in red the one considered for this test

-CHAOS hits blue boxes, in red the one considered for this test

The fugu context contains:

-the fragment amplified in this test

-CHAOS hits blue boxes, in red the one considered for this test

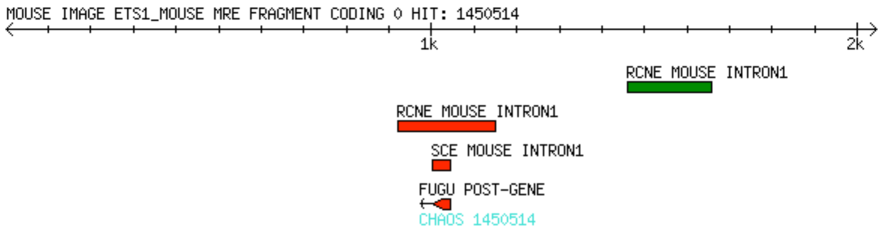

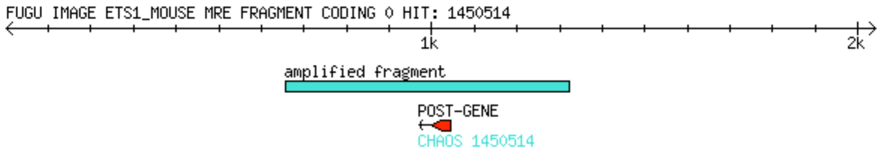

SEQUENCES

```
>fugu_fragment_for_primer_design_8726
XXXXXXXXXXXXXXXXXXXXXXXXXXXXXXXXXXXXXXXXXXXXXXXXXXXXXXXXXXXX
XXXXXXXXXXXXXXXXXXXXXXXXXXXXXXXXXAGCACAAGAACGAACGCGAACGTTCGGG
AGCCATGCGGACTTTGGCGAGGAATGTGGTCTTAATTGTAAGAAACGGCAAACTTTTTGG
GGGGGACCAAAATGTTTGGNNNNNNNNNNNNNNNNNAAATTTATGGTCAGTTTATGGTCTTT
GTCAACATCAAGCATCCATCTCTCAGACCAATGGGGTTTATTTTTGGTTGTTTTTTAAAG
TCCCAGACGCGTGCGTGCGTGCGTGCCTGATCTGTGCTGGTATTATGCAGCAGCTCAAAG
CAAATCAGGTAGAGTCCAATGTTTTGCCCAAGTAGCACGATGGGAAGACCATGAGGACCA
AGACGGAAAGACTGAGACTAATCAGAGATTCATTTCACCGTGAGTGTGAACAGATAGTGG
GCAGAATGTTCAGGCTGAGAGAAAGAGGACGGCAGAATCCGAGCAAACTCTGGGCTGTTT
CCCTTTTATTTACAGATGTGATCACTCTCCCGTTTGTCAAAAAGAGGGAGTTTTAGACGT
GGACCAAATACCTGTTTATATTTAGTTGTCTGAGGAGCCAGTGTTATATCATCCACATTC
AGAAGAGCCAGCGTAAAGCTGCCAGCCACATGTTGGAATGACTGTTTTTATGTTGTCAAT
GAAGGTTTTAAAGTGGAAGCAGACGTTAGCGGCTAGGTTCTCCAAAGAGGCATTGGCAGT
AATAAGCTGCTTCATTACCAGACAACACTATCGACAGCAGCGCAGCGCTGCGCAGCGCGC
GACAAGGTTACTCATTTCAGGAACGTCTGACGGGTATTCAACTGAAAGAAACCGTTGCTT
CAGAAACTGATGCTTTCTGCTTGACGTACATTCGCTACCCATGATGACTGAAACTTGTGC
ACCGTTCTGCATTACCCACCGGCAGTATTAACATTATTTAGATAGTTTTATGCAGATTTT
TCAAATTCTTTTTAAAGGAAGCCATTTAATTTAATTCTCCTTCTGTGCACCACTCAGGAA
GGTACAGTGTATTCAAATGCAACAGACACTTGAGGNNNNNNNNNGAATAGGAAGTTGTTA
CGGGATAAATGAAGGTGGATCTGAGAGGTGTGGAGTTGCTGGTGATGTGTGGAATTGTGC
ATTGAAGGAATTGAACAGGTTGCGTACATTGGCCTTTTGTCCAGAACATAAATCACTGTA
AAGATTTTCTTGTTCCAGTTAATTGGCAGCATTTTCTCCTCAAGACTGGGAACTATGGCT
GCATTCCGGCCTTATGGGAAATGCCAAAGCGGAATATCATCTTATTGTTTACAGTGGCTG
TATTTTAGCCAGATGGATGAAAATAGCTCATACGATGTCTATTTGTACAGATTGCATTTC
ATAACGCTTTATAGAGACCCAGATTAGACAAATAAGCAAGAGACAAAGCAGGAATAATAA
CCAAAGCTCATGTGAGGTCTTCGGCCGCTGCGCTGCATGCATCGTCCACTGATGCCAGAT
TCTCTCGCATCTCTGAAAAGAGTCGACTTTTGCTTTTCATCTTATGTGAAATGAGCTTTG
GGTCCGGGACGTGGGAAGAGGAGATGCTGTATGTGTTCCTGTTGGGTTTCACTGGGTTGA
CGCAGTTGAAACCATCGGATACCGAATCCAACAAGTTCATGAGCGAACAGCCGCACTGTC
CTTTGCATCGTTTTCTGCTTCGTGTCAAGGTTTTGAAACACTGNNNNNNNNNNNNNNNNN
NNNNNNNNNNNNNNCCCTATTGTCACTGTTTTGTGTTGCAGAGCTTTAAACAGAACTAAG
ACGACTGTCACTGGCCTCCCCCCGCTCAAAGTTAATGTGCTAAAGAAAACCTTGTTACCT
AAAATGTTGTATTGTTGAATGTCACAAATTATGATCTACTGCAGGTCTTATGTATTATGG
AATCATAGTCAGGAAATGTGTGTAAACACCAGTAGTATGTAACATACACGCATATCTCAA
AA
```

```
>amplified_fugu_segment
ACATTCAGAAGAGCCAGCGTAAAGCTGCCAGCCACATGTTGGAATGACTGTTTTTATGTT
GTCAATGAAGGTTTTAAAGTGGAAGCAGACGTTAGCGGCTAGGTTCTCCAAAGAGGCATT
```

```
GGCAGTAATAAGCTGCTTCATTACCAGACAACACTATCGACAGCAGCGCAGCGCTGCGCA
GCGCGCGACAAGGTTACTCATTTCAGGAACGTCTGACGGGTATTCAACTGAAAGAAACCG
TTGCTTCAGAAACTGATGCTTTCTGCTTGACGTACATTCGCTACCCATGATGACTGAAAC
TTGTGCACCGTTCTGCATTACCCACCGGCAGTATTAACATTATTTAGATAGTTTTATGCA
GATTTTTCAAATTCTTTTTAAAGGAAGCCATTTAATTTAATTCTCCTTCTGTGCACCACT
CAGGAAGGTACAGTGTATTCAAATGCAACAGACACTTGAGGNNNNNNNNNNGAATAGGAAG
TTGTTACGGGATAAATGAAGGTGGATCTGAGAGGTGTGGAGTTGCTGGTGATGTGTGGAA
TTGTGCATTGAAGGAATTGAACAGGTTGCGTACATTGGCCTTTTGTCCAGAACATAAATC
ACTGTAAAGATTTTCTTGTTCCAGTTAATTGGCAGCATTTTCTCCTCAAGACTGGGAACT
ATGGCTGCA
```

```
>rCNE_mouse_sequence_1262007
TGTGTCACAGTTTTCCTGCTTTGGATACAGGCAGTTGGGCTGTGTGGATGTGTGTGAATA
TGCATCTTCCTTCTCAGCCCGGATTTATTTATTAAAAGAAAAACACATCTGCATAAAAAA
GCCTCAGAACACAGGAAGAATGTCTACTTTGTTTTTCTTTCTGTAGCATCAAGACACGCG
GGGTTTTGGCTTCCTCTGAGGGATTTTGAAGACTGGCTTTCATCTCAAAC
```

```
>SCE_mouse_sequence_1262069
GGATTTATTTATTAAAAGAAAAACACATCTGCATAAAAAAGCCT
```

FUGU HITS IN THE CONTEST OF MAMMALIAN rCNE

```
CLUSTAL W (1.83) multiple sequence alignment


ENSG00000134954         TTTTTCATTGTGGTTTCCCTGGGTTGAAAACAGACAGTTGCACTGTGTGAATGTGTATGA
ENSCAFG00000010304      TTTTTCATTGTGGTTTCCCTGGGTTGAAAACAGACAGTTGTACTGTGTGAATGTGTATGA
ENSRNOG00000008941      TGTGTCACGNNNNNNNNNCTGGTTTGGATACAGACAGTTGTGCTGTGTGGATGTGTGTGA
ENSMUSG00000032035      ---TGTGTCACAGTTTTCCTGCTTTGGATACAGGCAGTTGGGCTGTGTGGATGTGTGTGA
SINFRUG00000153673      ------------------------------------------------------------


ENSG00000134954         GTATGCTTTTTCCTTCTCAGTCCAGATTTATTTATTAAAAGAAAAACACATCTGCATAAA
ENSCAFG00000010304      GTACGCGTTTTCCTTCTCTGTCCAGATTTATTTATTAAAAGAAAAACACATCTGCATAAA
ENSRNOG00000008941      ATATGCATTTTCCTTCTCTGCCCAGATTTATTTATTACANNNNNNNNNNATCTGCATAAA
ENSMUSG00000032035      ATATGCATCTTCCTTCTCAGCCCGGATTTATTTATTAAAAGAAAAACACATCTGCATAAA
SINFRUG00000153673      -----------------------GGCTTCCTTTAAAAAGAATTTGAAAAATCTGCATAAA
                                                * **  ****  *            ***********

ENSG00000134954         AAAGCCTTAGAACGCAGGAAGAATGTCCACTTTGTTTTTCTTTCTGTAGCACCGAGACAT
ENSCAFG00000010304      AAAGCCTCAGAACACAGGAAGAATGTCCACTTTGTTTTTCTTTCTGTAGCACCAAGACAT
ENSRNOG00000008941      AAAGCTTCAGAACACAGGAAGAATGTCTACTTTGTTTTTCTTTCTGTAGCACTAAGACAC
ENSMUSG00000032035      AAAGCCTCAGAACACAGGAAGAATGTCTACTTTGTTTTTCTTTCTGTAGCATCAAGACAC
SINFRUG00000153673      ACTATCT-----------------------------------------------------
                        *     *

ENSG00000134954         -GTGGGGTTTCAGCTTCCTCTAAGAAGTTTTTGAAGACTGGCTTTCATCTCAAAC
ENSCAFG00000010304      -GTGGGGTTTCAGCTTCCTCTAAGGGGTTTTTGAAGACTGGCTTTCATCTCAGAC
ENSRNOG00000008941      CGCCGGGTTTCGGCTTCCTCTGAGGGATTTT-GAAGACTGGCTTTCATCTCAAAC
ENSMUSG00000032035      -GCGGGGTTTTGGCTTCCTCTGAGGGATTTT-GAAGACTGGCTTTCATCTCAAAC
SINFRUG00000153673      -------------------------------------------------------
```

Fragment ETS1_MOUSE_8728

Representation of conserved regions within their genic context

Within the mouse context is shown the rCNE from which the tested SCE is derived (red diamond)

Within the fugu context all CHAOS hits obtained from the mouse rCNE are shown (green diamonds, in red the one tested)

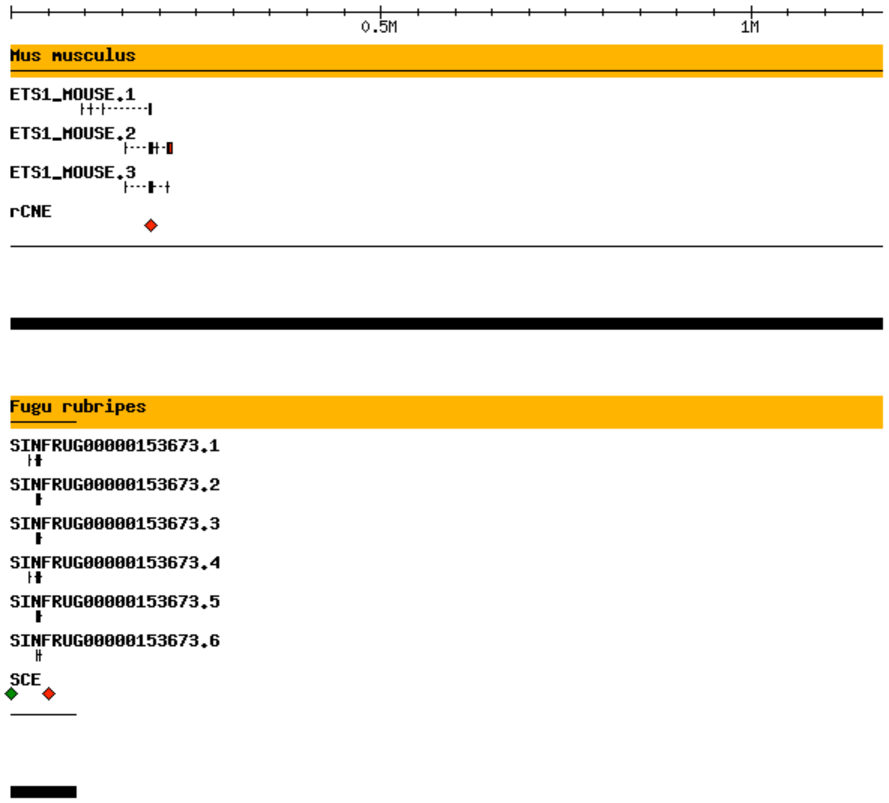

Conserved regions shown within 2000bp context

The mouse context contains:

-rCNEs green boxes, in red the one considered for this test

-SCEs yellow boxes, in red the one considered for this test

-CHAOS hits blue boxes, in red the one considered for this test

The fugu context contains:

-the fragment amplified in this test

-CHAOS hits blue boxes, in red the one considered for this test

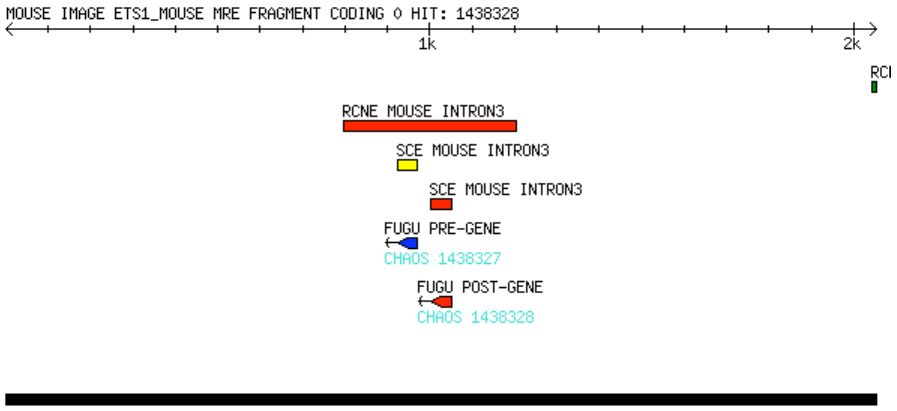

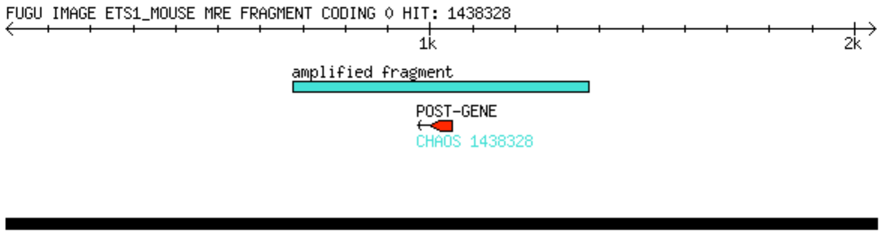

SEQUENCES

>fugu_fragment_for_primer_design_8728
ACTTCTGTTTAAACCCAAATGGAAAGACAGACTCTCCAGTGCAGACCAGCATCATTGCCA
GTGAGGTGTTTGGATGCAGGCAGAGCTGTGTAGCTCTACACAAATACACAAGTTTAAGAT
CGGCTACAGTGTTTGAACTTCCTCTCGTCTCCACGTTGATTCCATGATTGACGAACACTG
ATAATTAATAATTGATAAGGACTTTATGCCAAGAAAACCTACTACTATTAACTATTTATA
CTTCACCATGGAGCTGTATTAGACAGAACTTCAATGCTGTTGAATAAGAGATGCTTAACC
TGAGATGCTTAAACTCTCTGTGGTTGTCATGTAATCATATTTAATGTGACTTGGTTTCTA
CAATAAAACATTCTGTGCACCCTGACTGTGTCAGAGGAAGCATTCACATGATGAGTTGAG
CCAAGACCTTGTTTTTGCCATTACCACAAACCATCTTCTCATGCTTCTTCCCCACGGCGC
AAGTATCCTGGATGTCTCAAGGTCCAAACTGAAACACTGTGGACGTTCTCCTGGCTGTCT
CCGTCATACCAAGTTAAGAGTACTTGCAAACACCACATCTGCCTCAAATGAGGGTTTGCC
CTGAACTCTAACTGGCTGCGTTTATCACTGAAGAGAAAGTTATGCTGAATATTGTCTCAG
GCCACAAATAATTGCTGAGCCTAAACACACCACCTCGGGTGTAATTTGCATGGTCTGTGC
ATGAAGGCCAGGCGCAACCATAAATGCACCAAGCATAAATAACTAAACCCCATGAAAAAT
GCATTATATGATGTCTGAGAGCAGTTTCAATAGGACTGATGTTCATGTTCCAGGGATACA
GAGGAAGGACAGCTAGATGCTTAGCTAAGTTTGGTAAATAACAGAACGAAAGCAAAGAGT
TGAATTACCCCAGTGTCCCGGTACACAACAAGGACAGTGATGGGAGACTGCAGAAGAAGA
AGAAAGCCCAAGGACACATACAGGAGGTCGACTGAGCGCTATATAGAAATTTAGAATAGG
ATATGAAGGAGCACGGAAATGTCCGACTTACGTTGCCTCTCATTTCATGAGCTGAGTTTG
GAGGGTTCCAGAATGTCCATCTTTGAGTCATTTCAGATGATAACCGTGTTGGACCCGGAG
CCAGCCATACATTATCAGCCGCTATCTTAAAATGCAGTTCCTCAAAGCTCTACGGAATCC
CTCCTGCTCTCTGCAGCTGGAATGCAAGTCCTTACACTTCAAATATAAGTCTCCAGTAAC
AACATGGTTTATTATTACAATAGCAACAAATACTAAGACAAGTAGGAATTTGATAAAAAG
CAGAGACTTATAAGAAAACTATCATCTGACTTACGTCAGCCATTTTCTCACGATTTTCTT
TTATTTTCATGTGTGGAATTTAAGCCACATTTACTCAAAAATGAGTAGCACTGTTTAGTC
TATACCAAAATGTGAATACATGGCCAACAATCCAACCCGACGCCAGCTACTGTTGTGTCT
GTTTCAGTAGTCTGACTAAGGTTATAGCTTAGAAGGTTTAATGACAAGTTTAGAATGGAG
TGCCTACTCAGTGGGACACTCTCCCAGTTTTTTCCCACATTACGGGGGCCAAGGGGCCAA
GGACAAGGCGAGAGATGGGCTCCTTGCTCCACAGCAATCTTCTGTGGCTGGGAAAGGCTA
CGGCCCACTGTTCTCACCATTTCCCCTCTGTATTCACCAGCCCTGGTNNNNNNNNNNNNN
NNNNNNNNNNNNNNNNNNNNNNNNNNNNNNNNNNNNNNNNNNGGAGTGGACTCCAGGAAC
AACTATGGTCAGGGACTTAGGCCAAACTATTAAGTCAAAGGATGACTCGTGTGTGGTAAA
AAAGCATGGCACTTATTTAACTTTCATAGGAAAAGGGAGTTTAAGAAGAACTTTCTCTCT
TTTTTGTAATGGTGAGTGTCCTGTGTAGGATCTTTGGGAAAACACTCAGCCGCATGACAA
CAATCCACATTGCAGGTTACTCTTGTGACGAAGTATAAGAATTAGAAAACATTTTTCCTT
TTGTTAGTAG

```
>amplified_fugu_segment
AGCCTAAACACACCACCTCGGGTGTAATTTGCATGGTCTGTGCATGAAGGCCAGGCGCAA
CCATAAATGCACCAAGCATAAATAACTAAACCCCATGAAAAATGCATTATATGATGTCTG
AGAGCAGTTTCAATAGGACTGATGTTCATGTTCCAGGGATACAGAGGAAGGACAGCTAGA
TGCTTAGCTAAGTTTGGTAAATAACAGAACGAAAGCAAAGAGTTGAATTACCCCAGTGTC
CCGGTACACAACAAGGACAGTGATGGGAGACTGCAGAAGAAGAAGAAAGCCCAAGGACAC
ATACAGGAGGTCGACTGAGCGCTATATAGAAATTTAGAATAGGATATGAAGGAGCACGGA
AATGTCCGACTTACGTTGCCTCTCATTTCATGAGCTGAGTTTGGAGGGTTCCAGAATGTC
CATCTTTGAGTCATTTCAGATGATAACCGTGTTGGACCCGGAGCCAGCCATACATTATCA
GCCGCTATCTTAAAATGCAGTTCCTCAAAGCTCTACGGAATCCCTCCTGCTCTCTGCAGC
TGGAATGCAAGTCCTTACACTTCAAATATAAGTCTCCAGTAACAACATGGTTTATTATTA
CAATAGCAACAAATACTAAGACAAGTAGGAATTTGATAAAAAGCAGAGACTTATAAGAAA
ACTATCATCTGACTTACGTCAGCCATTTTCTCACGA
```

```
>rCNE_mouse_sequence_1261937
AGACAAGTTTCTCTGCCAGTCACTGGCTTGGGATTAAGGCAGCGTAGAATACCTTTTACA
TTTGGAGCCAAAGCTAGGGTTATATCAGGACGTGGTGTTGCAACCTTGTAATGACACAGC
CTCCTCTACTGGCCTGGCCTCTTCTAACCTTTTTGGACCAAGTGACTCATGTCCCGTAGC
TCCGGCTGAGTATAGCAACAGCAGCGGGCCGCAAACTTGGGTGTTTCTGTGCTTATTTGT
CTAAATTTCTTCTTAAAATAAATGGCAGTTACCAGCAGTCAGCAGTGCCCTACAGCTGAC
TGTAAGCCTAGAAGCCTGCTGCCCTCTGTAACCAGGCCCACCCTTTCCCTGTGGTTACCA
GCCTCAATCTCTAGGCACAGCATCTGGGGGCAGGGCACACAGCTCAG
```

```
>SCE_mouse_sequence_1262071
GCGGGCCGCAAACTTGGGTGTTTCTGTGCTTATTTGTCTAAATTTCTTCTTA
```

FUGU HITS IN THE CONTEST OF MAMMALIAN rCNE

```
CLUSTAL W (1.83) multiple sequence alignment


ENSMUSG00000032035      AGACAAGTTTCTCTGCC-AGTCACTGGCTTGGGATTAAGGCAGCGTAGAATACCTTTTAC
ENSRNOG00000008941      CGACAAATGTCTCTGCC-AGTTGCTGGTTTGGCATTATGGCGGTGTAGAACACCTTTTAC
ENSG00000134954         AGACAGATTTCTCTGCCCACTAACTGAATTGGAATTGCAGTAGTGCATCATACCTTTTAC
ENSCAFG00000010304      AGACTGGCTTCTCTGCCCCCTACCTGGGGTGGAATTGCAGTCGTGTGGGCTACCTTTTTC
SINFRUG00000153673      ------------------------------------------------------------


ENSMUSG00000032035      ATTTGGAGCCAAAGCTAGGGTTATATCAGGACGTGGTGTTGCAACCTTGTAATGACACAG
ENSRNOG00000008941      ACTCCGAGCCAAAGCTAGGGTTATATCAGGACGTGGTGTTGCAACCTTTTAATGACACAG
ENSG00000134954         ATCTGGAGCAGGAACTGGGGTTATATCAGGACGCTATGTTGCAACCTTATAATGACATGG
ENSCAFG00000010304      ATCTGGAGCAGCAGCTGGGGTTATATCAGGATACCGTGTTGCAACCTTGTAATGACATGG
SINFRUG00000153673      ------------------------------------------------------------


ENSMUSG00000032035      CCTCCTCTACTGGCCTGGCCTCTTCTAACCTTTTTGGACCAAGTGACTCATGTCCCGTAG
ENSRNOG00000008941      CCTCCTCTACTGGCC-----TCTTCTAACCTTTTTGGACCAAGTGACTCATGTCCCGTAG
ENSG00000134954         CCTCCCCTAACAGCC-----TCTTCCAACCTTTTTGGACCAAGTGACTCATGTACCACAG
ENSCAFG00000010304      CCTCCCCTACCAGCC-----TCTTCCAACCTTTTTGGACCAAGTGACTCATGTACCACAG
SINFRUG00000153673      ------------------------------------------------------------


ENSMUSG00000032035      CTCCGGCTGAGTATAGCAACAGCAGCGGGCCGCAAACTTGGGTGTTTCTGTGCTTATTTG
ENSRNOG00000008941      CACCGGCTGAGTATAGCAACAGCAGCGGGTGGCAAACTTGGGTGTTTCTGTGCTTATTTG
ENSG00000134954         TCCTGGCTGAGTATAGAAACAGCAGCAGGCGGCAAACTTGGGTGNNNNNNNNNNNNNNNN
ENSCAFG00000010304      TCCTGGCTGAGTATAGAAACAGCAGCAGGCGGCAAACTTGGGTGTTTTTGCACTTATTTT
SINFRUG00000153673      ------------------------GTAAGTCGGACATTTCCGTGCTCCTTCATATCCTAT
                                                *   *  * * * **  ***

ENSMUSG00000032035      TCTAAATTTCTTCTTAAAATAAATGGCAGTTACCAGCAGTCAGCAGTGCCCTACAGCTGA
ENSRNOG00000008941      TCTAAATTTCTTCTTAAAATAAATTGCAGTTACCAGCAGTCAGCAGTGCCCTACAGCTGA
ENSG00000134954         NNNNNNNNNNNNCTTAAAATAAATTGCAGTTACCAGCAGTCAGCAGTGCCCTTCAGCTGA
ENSCAFG00000010304      TCTGAATTTCTTCTTAAAATAAATTGCAGTTACCAGCAGTCAGCGGTGCCCTCCAGCTGA
SINFRUG00000153673      TCTAAATTTCTATATA--------------------------------------------
                                      **

ENSMUSG00000032035      CTGTAAGCCTAGAAGCCTGCTGCCCTCTGTAACC-AGGCCCACCCTTTCCCTGTGGTTAC
ENSRNOG00000008941      CTGTAAGCCTAGAAGCCTGCTGCCCTCTGTAACC-AGGCCCACCCTTTCCCTGTGGTCAC
ENSG00000134954         CTGTAAGCCTAGAAGCCCATTGCCCTCTCTAACCCAGGCCCATCCTTTCCCTGTGGTCAT
```

```
ENSCAFG00000010304      CTGTAAGCCTAGAAGCCCGTTGCCCTCCCTGACCCAGGCCCACCCTTTCCCTGTGGTCAC
SINFRUG00000153673      ------------------------------------------------------------


ENSMUSG00000032035      CAGCCTCAATCTCTAGGCACAGCATCTGGGGGCAGGGCACACAGCTCAG
ENSRNOG00000008941      CAGCCTCAAGCTCTAGGCACAGTCACTGGGGGTAGGGCACAGAGCTCAT
ENSG00000134954         CAGCTCTAA-CCCTTGGCACAGACGCTGGGGGAGGGGAGCAAAACTCAG
ENSCAFG00000010304      CCGCTCCAA-CCCTTGGCGCAGACGCTGGGGGCAGGGAGCAAAGCTCAG
SINFRUG00000153673      -------------------------------------------------
```

Fragment Mapkap1_17058

Representation of conserved regions within their genic context

Within the mouse context is shown the rCNE from which the tested SCE is derived (red diamond)

Within the fugu context all CHAOS hits obtained from the mouse rCNE are shown (green diamonds, in red the one tested)

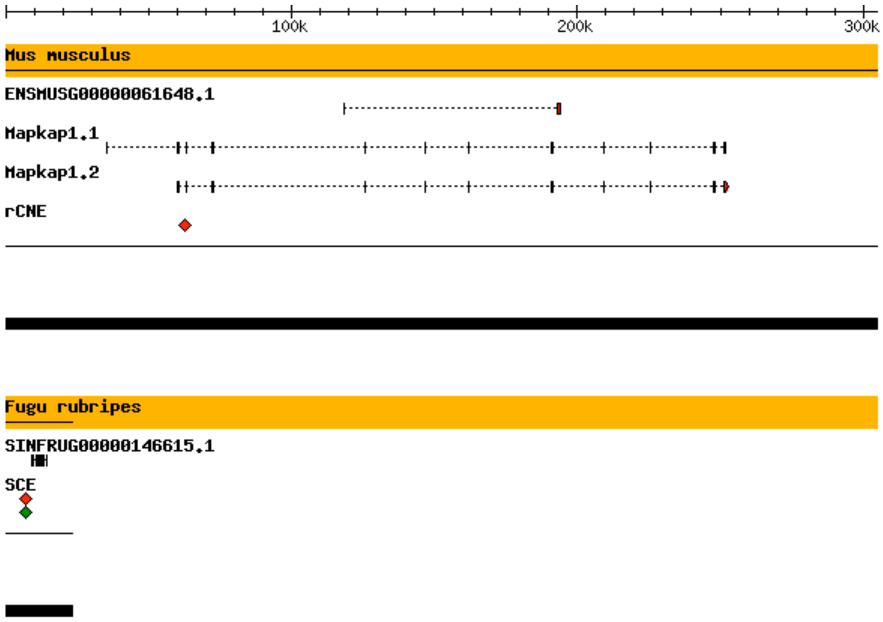

Conserved regions shown within 2000bp context

The mouse context contains:

-rCNEs green boxes, in red the one considered for this test

-SCEs yellow boxes, in red the one considered for this test

-CHAOS hits blue boxes, in red the one considered for this test

The fugu context contains:

-the fragment amplified in this test

-CHAOS hits blue boxes, in red the one considered for this test

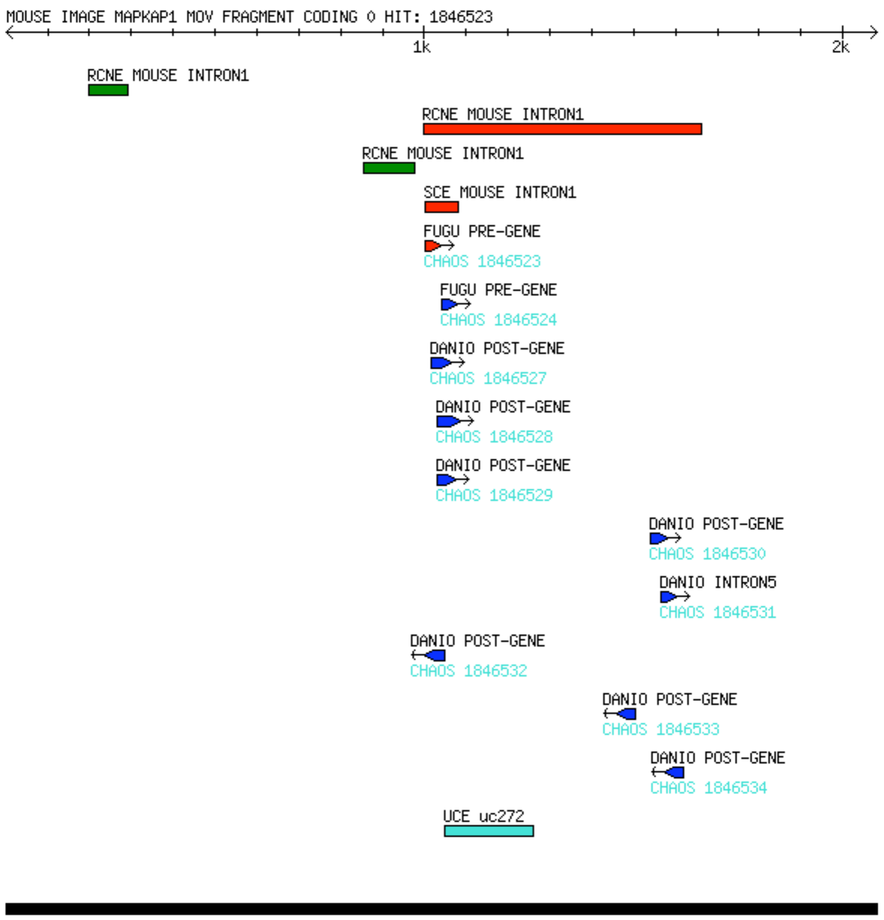

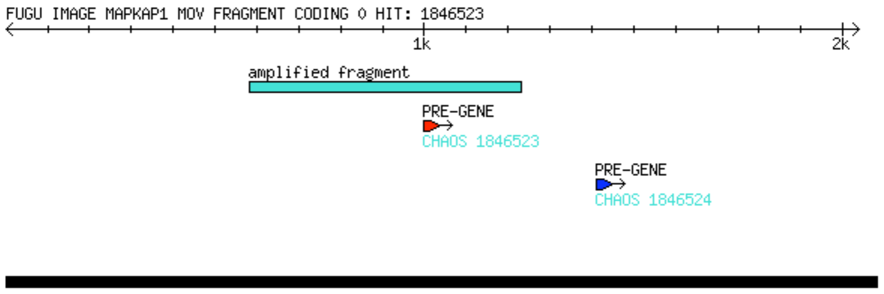

SEQUENCES

>fugu_fragment_for_primer_design_17058
TCCTGACTGCTGGAAACCCTCGTCTGCAGCCAGGACAACTTTTCAGATCTCAGTCCAGCT
CGGTCCCTGTCACTAATCGCTGTGAAGTCAAAACAATGCAGAAGCCTTTTGGGAAACCAG
TGAGAGTGATCGACACTCCAGACTTTTTTCACAGCCAGATGAAAGACTCGGAGCTGCAGA
TAGAAGAATGCAAGAAGTTCTGTCGGCCAGGGCAGTGTGTGGTGTTACTGGTGTTTCAGC
TGGGACGCTTCACCCGTGAGGACCAAGAACTCCTGGAGAAGCTGGAGGACAAGTTTGGCT
GGAGAATCAGGGAGAGCACAATCATTCTCTTGACCCACGGAGAGGACCTGAAAGGAGACT
GGGGGCCATCTATTAAGGCCGACACTGAACTCTGGAATACTATTGAAGCATGCGGCTCTC
GTTATCATGTCATCAGCAACAATTCCAAGAAAGTGAAGCAAGTCATAGACCTCCTCAAGA
AGATACCAGACTTTAAAAACATTTTCCCAGACTTCAGGAGCTCACCGATTAATTGTTTTC
AAAGTTAGTTTGACCATTTTTATGGACTGGAAATCTACGGTTAGGCACTGGGGACAGAGT

```
GAAAAGAACACTTCTCATTCACACAAAATATGACATGAGTATACCAATCAATAAAATGAA
AGATGAATCATTCATCCTGCATATATGAGGATAAAGCAACTGATATATAACAATTCCTGT
CAACTGGTACAAAGAAGAAAATCTAATGAGCTATAATTTTACCAAATCTTACCAAATCTT
TTTTTAAATTATCTTATTCACTCCCTTGGCTAAAAGATTCAATATGGAAACTTATAATGT
GAAAAATGTCTTCTCTATATTGTCTTTTCTCTGGTGTTGGGGGAGGGCAGAGATTTGTCA
AGAAGAGACTAGAATTTAAAAACCAACACAACTTTTGCTGAGTTTAATTTTCCAAAAGGA
GCAGGGTGGCCCATGTGGAGCTGACAAGTCAGGAAATTCCCAAGGAATTGACTCTTGGGT
CTTTTAAGAACTACCTAAATTGAGAGACTTGAGACAGATTCCAAATCATAACCACTTTAT
AATGAATAATGTTTAAAGTTTTTGACTGGGATCCAGGGCGAAATAGAGGAAATTACTAAA
TGTCACGTTCAGAGGTCTATGAACAGAAAAACTGAAATGAGACTGACCCCTAGGGGCAGC
AAATAAGGCAAAATGCAACCACCATCAATAAATTCCTCATCTTATAGAGCCCGATTGTGT
AGGTGTGTGAGGCAGGCTTGGTTAAGGAGGGCTTAGGTCAGAAGAAGTACTTTTGAACAT
AATGCAATTTGGAACAGATTGGAGGTTATGGTGGACGGGTTCACTGAAGGGGAAGTGAGT
GAATAGGGAGTTCTGAATACATTTAAGTTTATTTTTTTAAAACCCTATTGTACTTTTCTA
CATTTATTTAATAAAGTCATACTTTACTTTCTCCCGTAGAACTAACGTGGTGGATTTTTG
AATTGATAATTGAGTTAGAAGATGGCTCCAGGAGACTTTTTTGCCCGTCTTTTGATGTTC
CATAACCCCACCAAATTTAATTCCATGTAGCTCTAACCATATCTGTCNNNNNNNNNNNNN
NNNNNNNNNNNNNTTTTTTGTACCCCCCAAAAATTGCGTACTCTCCCGCATTTTGGGGTA
CGCAAATTTTCAACGGTTGTCGAGCGATTTCAAATGCAAATTTATGTGGGAGAAGAATAA
CAAAAACACAAATATTTGAAACAAATAGGGCCTTATGAAGATAATCTCAGGTTTTATGCA
TAAACAGACCACAACACATGGGCTAGACTTCCAAACTAAATCGGTGAAATAAATTTTACC
ACAAGGTGGCAGATTGGCCCTTTGCGCCGGTGGACACGCGAAGAAATCCACCACAGAAGA
AGAGAACTCTGCATCGAAGCTACACTAAATGAGCTATTTGCATCGTTTTACAGTTTTATC
ATGCCTTTAGTATATCGCTTTCCTAAGCTCCGAACTATTTATTCTGCTCACAGACTGG
```

```
>amplified_fugu_segment
TTAGGCACTGGGGACAGAGTGAAAAGAACACTTCTCATTCACACAAAATATGACATGAGT
ATACCAATCAATAAAATGAAAGATGAATCATTCATCCTGCATATATGAGGATAAAGCAAC
TGATATATAACAATTCCTGTCAACTGGTACAAAGAAGAAAATCTAATGAGCTATAATTTT
ACCAAATCTTACCAAATCTTTTTTTAAATTATCTTATTCACTCCCTTGGCTAAAAGATTC
AATATGGAAACTTATAATGTGAAAAATGTCTTCTCTATATTGTCTTTTCTCTGGTGTTGG
GGGAGGGCAGAGATTTGTCAAGAAGAGACTAGAATTTAAAAACCAACACAACTTTTGCTG
AGTTTAATTTTCCAAAAGGAGCAGGGTGGCCCATGTGGAGCTGACAAGTCAGGAAATTCC
CAAGGAATTGACTCTTGGGTCTTTTAAGAACTACCTAAATTGAGAGACTTGAGACAGATT
CCAAATCATAACCACTTTATAATGAATAATGTTTAAAGTTTTTGACTGGGATCCAGGGCG
AAATAGAGGAAATTACTAAATGTCACGTTCAGAGGTCTATGAACAGAAAAACTGAAATGA
GACTGACCCCTAGGGGCAGCAAATAAGGCAAAATGCAACCACCATCAAT
```

```
>rCNE_mouse_sequence_1596340
GTGATCAAGGAATTTACTTTTTAATCTTTTCAGTACATCTTAAAGTTTTTTAAAGAAAAA
TTGTTCTTTTTAAGATTTGACTAATGTTACAAAGATACAAATCATTTATTTTACTACCTA
TAAACTGTTTCAGCCCACTGAAGAAAAGCTATGCTAATTTATATAGCTTTGACATTTTAT
AATTTTCATGATTTTTAGATAAAGGATTTTATATTTAAAACTTTAATAGTCTAGTCTGAG
TTCATTTATCTTTAATTTTTGAAACGGGTAGAGTAATTCATAACAGTAATTTTATTCCTG
TAATGTAATATTCACTTCAGTTCATTTGTGCATTAAAATTCCTTTAAGACCGTTATTAAT
GATTTATATAATGAAATGTCATATTAATCACAGAAGTTTTCACATTTTATTATTCTCTTC
CATGTTAATACTATCTTTATGATCTCTATTAATGTTTTATATCCTCTAATTTCAATTATA
AATCAAGAATATTTTCATGTTTTTGTTGGTATTTGCTTTTCAGGGGTGCCACCTTTAAAA
TTTTATGACTTTTTCCCTCCATATGCAATATAAAACATCTTTATTCATATAAAGGTAGTT
AATTATGTCTTTGTTTATAGTAAACTTTCATTGACAAACTTATAGTTATAATATTTCTTT
TCAAG
```

```
>SCE_mouse_sequence_1596400
TCAAGGAATTTACTTTTTAATCTTTTCAGTACATCTTAAAGTTTTTTAAAGAAAAATTGT
TCTTTTTAAGATTTGACTAAT
```

FUGU HITS IN THE CONTEST OF MAMMALIAN rCNE

CLUSTAL W (1.83) multiple sequence alignment

```
ENSG00000119487         GTGACCAAAGAATTTACTTTTTAATCCATGCAGCATATCTTAAAATTTTGTAAAAGAAAA
ENSCAFG00000020178      GTGACCAAAGAATGTACTTTTTAATCCTTTCAGCATATCTTAAAATTTTGTATAAGAAAA
ENSRNOG00000017583      GTGATCAAGGAATTTAGTTTTTAATCTTTTCAGTACATCTTAAAAGTTTTTTAAAGAAAA
ENSMUSG00000038696      GTGATCAAGGAATTTACTTTTTAATCTTTTCAGTACATCTTAAAGTTTTTTA-AAGAAAA
SINFRUG00000146615      ----CCAAGGAATTGACTCTTGGGTCTTTTAAGAACTACCTAAA----------------
```

```
                               *** ****  * * **   **  *  ** *   * ****

ENSG00000119487           ATTGTTCTTTTTAAGATTTGACTAATGTTACAAAGATACAAATCATTTATTTTACTACCT
ENSCAFG00000020178        ATTATTCTTTTTAAGATTTGACTAATGTTACGAAGATACAAATCATTTATTTTACTACCT
ENSRNOG00000017583        ATTGTTCTTTTTAAGATTTGACTAATGTTACAAAGATACAAATCATTTATTTTACTACCT
ENSMUSG00000038696        ATTGTTCTTTTTAAGATTTGACTAATGTTACAAAGATACAAATCATTTATTTTACTACCT
SINFRUG00000146615        ------------------------------------------------------------


ENSG00000119487           ATAAACTGTTTCAGCCCACTGAAGAAAAGCTATGCTAATTTATATAGCTTTGACATTTTA
ENSCAFG00000020178        ATAAACTGTTTGAGCCCACTGAAGGAAAGCTATGCTAATTTATATAGCTTTGACATTTTA
ENSRNOG00000017583        ATAAACTGTTTCAGCCCACTGAAGAAAAGCTATGCTAATTTATATAGCTTTGACATTTTA
ENSMUSG00000038696        ATAAACTGTTTCAGCCCACTGAAGAAAAGCTATGCTAATTTATATAGCTTTGACATTTTA
SINFRUG00000146615        ------------------------------------------------------------


ENSG00000119487           TAATTTTCATGATTTTTAGATAAAGGATTTTATATTTAAAACTTTAATAGTCTAGTCTGA
ENSCAFG00000020178        TAATTTTCATGATTTTTAGATAAAGGATTTTATATTTAAAACTTTAATAGTCTAGTCTGA
ENSRNOG00000017583        TAATTTTCATGATTTTTAGATAAAGGATTTTATATTTAAAACTTTAATAGTCTAGTCTGA
ENSMUSG00000038696        TAATTTTCATGATTTTTAGATAAAGGATTTTATATTTAAAACTTTAATAGTCTAGTCTGA
SINFRUG00000146615        ------------------------------------------------------------


ENSG00000119487           GTTCATTTATCTTTAATTTTTGAAATGGGTAGAGTAATTCATAACAGTAATTTTATTCCT
ENSCAFG00000020178        GTTCATTTATCTTTAATTTTTGAAACGGGTAGAGTAATTCATAACAGTAATTTTATTCCT
ENSRNOG00000017583        GTTCATTTATCTTTAATTTTTGAAACGGGTAGAGTAATTCATAACAGTAATTTTATTCCT
ENSMUSG00000038696        GTTCATTTATCTTTAATTTTTGAAACGGGTAGAGTAATTCATAACAGTAATTTTATTCCT
SINFRUG00000146615        ------------------------------------------------------------


ENSG00000119487           GTAATGTAATATTCACTTCAGTTCATTTGTGCATTAAAATTCCTTTAAGACCGTTATTAA
ENSCAFG00000020178        GTAATGTAATATTCACTTCAGTTCATTTGTGCATTAAAATTCCTTTAAGACCGTTATTAA
ENSRNOG00000017583        GTAATGTAATATTCACTTCAGTTCATTTGTGCATTAAAATTCCTTTAAGACCGTTATTAA
ENSMUSG00000038696        GTAATGTAATATTCACTTCAGTTCATTTGTGCATTAAAATTCCTTTAAGACCGTTATTAA
SINFRUG00000146615        ------------------------------------------------------------


ENSG00000119487           TGATTTATATAATGAAATGTCATATTAATCACAGAAGTTTTCACATTTTATTATTCTCTT
ENSCAFG00000020178        TGATTTATATAATGAAATGTCATATTAATCACAGAAGTTTTCACATTTTATTATTCTCTT
ENSRNOG00000017583        TGATTTATATAATGAAATGTCATATTAATCACAGAAGTTTTCACATTTTATTATTCTCTT
ENSMUSG00000038696        TGATTTATATAATGAAATGTCATATTAATCACAGAAGTTTTCACATTTTATTATTCTCTT
SINFRUG00000146615        ------------------------------------------------------------


ENSG00000119487           CCATGTTAATACTATCTTTATGATCTCTATTAGTGTTTTATATCCTCTAATTTCAATTAT
ENSCAFG00000020178        CCATGTTAATACTATCTTTATGATCTCTATTAATGTTTTATATCCTCTAATTTCAATTAT
ENSRNOG00000017583        CCATGTTAATACTATCTTTATGATCTCTATTAATGTTTTATACCCTCTAATTTCAATTAT
ENSMUSG00000038696        CCATGTTAATACTATCTTTATGATCTCTATTAATGTTTTATATCCTCTAATTTCAATTAT
SINFRUG00000146615        ------------------------------------------------------------


ENSG00000119487           AAATCAAGAATATTTTCATGTTTTTGTTGGTATTTGCTTTTCAGGGGTGCCACCTTTAAA
ENSCAFG00000020178        AAATCAAGAATATTTTCATGTTTTTGTTGGTATTTGCTTTTCAGGGGTGCCACCTTTAAA
ENSRNOG00000017583        AAATCAAGAATATTTTCATGTTTTTGTTGGTATTTGCTTTTCAGAGGTGCCACCTTTAAA
ENSMUSG00000038696        AAATCAAGAATATTTTCATGTTTTTGTTGGTATTTGCTTTTCAGGGGTGCCACCTTTAAA
SINFRUG00000146615        ------------------------------------------------------------


ENSG00000119487           ATTTTATGACTTTTTCTCTACATATGCAATATAAAACATCTTTATTCATGTAAAGGTAGT
ENSCAFG00000020178        ATTTTATGACTTTTTCCCTACATGTGCAATATAAAACATCTTTATTCATATAAAGGTAGT
ENSRNOG00000017583        ATTTTATGACTTTCTCCCTCCATATGCAATATAAAACATCTTTATTCATATAAAGATAGT
ENSMUSG00000038696        ATTTTATGACTTTTTCCCTCCATATGCAATATAAAACATCTTTATTCATATAAAGGTAGT
SINFRUG00000146615        ------------------------------------------------------------


ENSG00000119487           TAATTATGTCTTTGTTTATGGTAAACTTTCATTGACAAACTTACAGTTATAATATTTCTT
ENSCAFG00000020178        TAATTATGTCTTTGTTTATAGTAAACTTTCATTGACAAACTTATAGTTATAATATTTCTT
ENSRNOG00000017583        TAATTATGTCTTTGTTTATAGTAAACTTTCATTGACAAACTTATAGTTATAATATTTCTT
ENSMUSG00000038696        TAATTATGTCTTTGTTTATAGTAAACTTTCATTGACAAACTTATAGTTATAATATTTCTT
SINFRUG00000146615        ------------------------------------------------------------


ENSG00000119487           TTCAAG
ENSCAFG00000020178        TTCAAG
ENSRNOG00000017583        TTCAAG
ENSMUSG00000038696        TTCAAG
SINFRUG00000146615        ------
```

Fragment Mapkap1_17059

Representation of conserved regions within their genic context

Within the mouse context is shown the rCNE from which the tested SCE is derived (red diamond)

Within the fugu context all CHAOS hits obtained from the mouse rCNE are shown (green diamonds, in red the one tested)

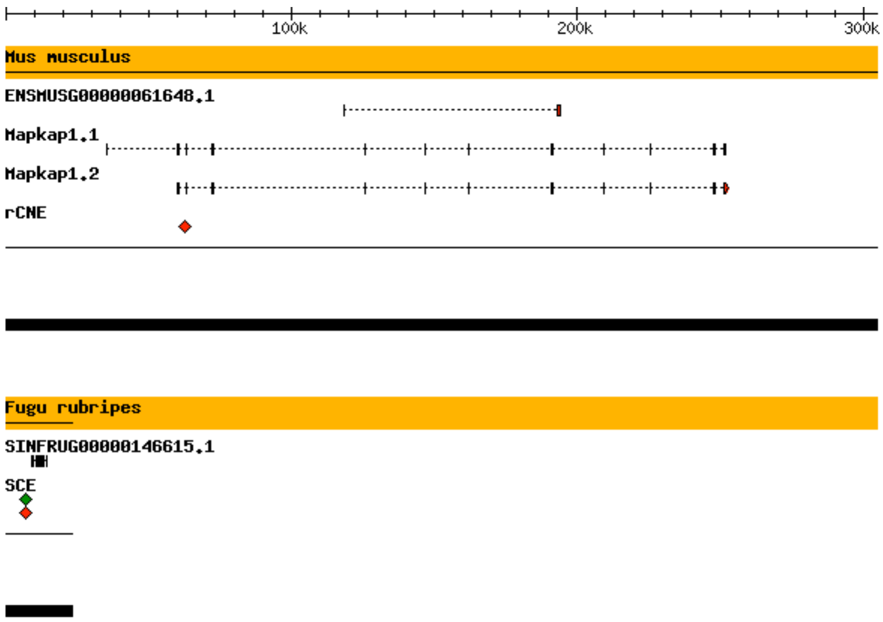

Conserved regions shown within 2000bp context

The mouse context contains:

-rCNEs green boxes, in red the one considered for this test

-SCEs yellow boxes, in red the one considered for this test

-CHAOS hits blue boxes, in red the one considered for this test

The fugu context contains:

-the fragment amplified in this test

-CHAOS hits blue boxes, in red the one considered for this test

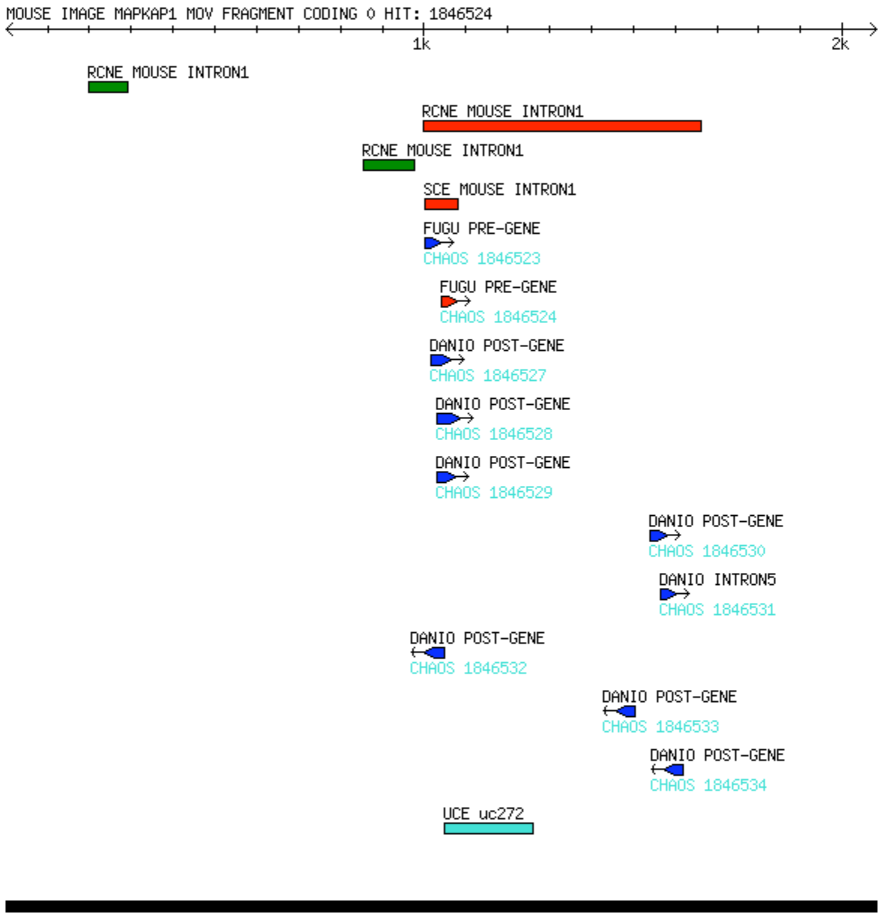

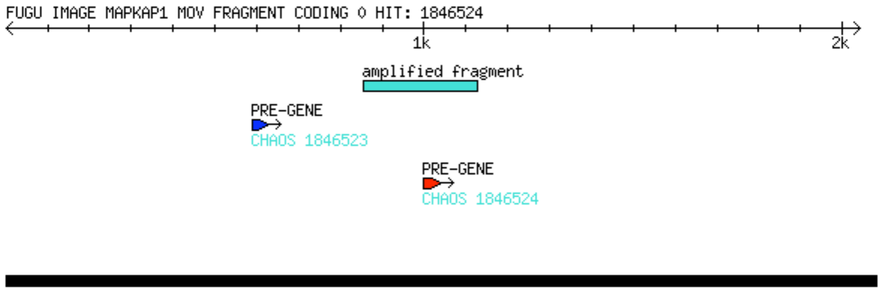

SEQUENCES

>fugu_fragment_for_primer_design_17059
GCGGCTCTCGTTATCATGTCATCAGCAACAATTCCAAGAAAGTGAAGCAAGTCATAGACC
TCCTCAAGAAGATACCAGACTTTAAAAACATTTTCCCAGACTTCAGGAGCTCACCGATTA
ATTGTTTTCAAAGTTAGTTTGACCATTTTTATGGACTGGAAATCTACGGTTAGGCACTGG
GGACAGAGTGAAAAGAACACTTCTCATTCACACAAAATATGACATGAGTATACCAATCAA
TAAAATGAAAGATGAATCATTCATCCTGCATATATGAGGATAAAGCAACTGATATATAAC
AATTCCTGTCAACTGGTACAAAGAAGAAAATCTAATGAGCTATAATTTTACCAAATCTTA
CCAAATCTTTTTTTAAATTATCTTATTCACTCCCTTGGCTAAAAGATTCAATATGGAAAC
TTATAATGTGAAAAATGTCTTCTCTATATTGTCTTTTCTCTGGTGTTGGGGGAGGGCAGA
GATTTGTCAAGAAGAGACTAGAATTTAAAAACCAACACAACTTTTGCTGAGTTTAATTTT
CCAAAAGGAGCAGGGTGGCCCATGTGGAGCTGACAAGTCAGGAAATTCCCAAGGAATTGA
CTCTTGGGTCTTTTAAGAACTACCTAAATTGAGAGACTTGAGACAGATTCCAAATCATAA
CCACTTTATAATGAATAATGTTTAAAGTTTTTGACTGGGATCCAGGGCGAAATAGAGGAA
ATTACTAAATGTCACGTTCAGAGGTCTATGAACAGAAAAACTGAAATGAGACTGACCCCT
AGGGGCAGCAAATAAGGCAAAATGCAACCACCATCAATAAATTCCTCATCTTATAGAGCC

CGATTGTGTAGGTGTGTGAGGCAGGCTTGGTTAAGGAGGGCTTAGGTCAGAAGAAGTACT
TTTGAACATAATGCAATTTGGAACAGATTGGAGGTTATGGTGGACGGGTTCACTGAAGGG
GAAGTGAGTGAATAGGGAGTTCTGAATACATTTAAGTTTATTTTTTTAAAACCCTATTGT
ACTTTTCTACATTTATTTAATAAAGTCATACTTTACTTTCTCCCGTAGAACTAACGTGGT
GGATTTTTGAATTGATAATTGAGTTAGAAGATGGCTCCAGGAGACTTTTTTGCCCGTCTT
TTGATGTTCCATAACCCCACCAAATTTAATTCCATGTAGCTCTAACCATATCTGTCNNNN
NNNNNNNNNNNNNNNNNNNNNTTTTTTGTACCCCCCAAAAATTGCGTACTCTCCCGCAT
TTTGGGGTACGCAAATTTTCAACGGTTGTCGAGCGATTTCAAATGCAAATTTATGTGGGA
GAAGAATAACAAAAACACAAATATTTGAAACAAATAGGGCCTTATGAAGATAATCTCAGG
TTTTATGCATAAACAGACCACAACACATGGGCTAGACTTCCAAACTAAATCGGTGAAATA
AATTTTACCACAAGGTGGCAGATTGGCCCTTTGCGCCGGTGGACACGCGAAGAAATCCAC
CACAGAAGAAGAGAACTCTGCATCGAAGCTACACTAAATGAGCTATTTGCATCGTTTTAC
AGTTTTATCATGCCTTTAGTATATCGCTTTCCTAAGCTCCGAACTATTTATTCTGCTCAC
AGACTGGATGGATAAAATGGGCCGATGTGGAAAGGTTTTTTTCTCACTGGATGTGACTAA
AGGTAGGTCTCCGTCATGAAGTAGCAGCATAGCAGCTAGTTTAGCTGGTCCCTTAAGGAA
GCATTACTGGGGTTAACCTGCGTTAACCTACTGAAACAACCTGGTTTTTATCACCTGAAA
GACACCTTCACACTTTCATGTCATCAAACAAGGTTATATTTAATGAAAATGTAGTCTGTC
AACAAGGGAATATAGCTAGGTCAAGCGACAGCAACGAATGCCACTGTCACACGGAAATAA
CACATCCAGTTTGATTCTAAAGTTAATCAAGATCAAATATTTCAGAAAAGACCACATCCA
TTCTTAAACAAAACAACTTTTTAATGCATTTGATCATCATCTTCAGTTGAAGGATATTAT

>amplified_fugu_segment
GTGAGGCAGGCTTGGTTAAGGAGGGCTTAGGTCAGAAGAAGTACTTTTGAACATAATGCA
ATTTGGAACAGATTGGAGGTTATGGTGGACGGGTTCACTGAAGGGGAAGTGAGTGAATAG
GGAGTTCTGAATACATTTAAGTTTATTTTTTTAAAACCCTATTGTACTTTTCTACATTTA
TTTAATAAAGTCATACTTTACTTTCTCCCGTAGAACTAACGTGGTGGATTTTTGAATTGA
TAATTGAGTTAGAAGATGGCTCCAGGAGACTTT

>rCNE_mouse_sequence_1596340
GTGATCAAGGAATTTACTTTTTAATCTTTTCAGTACATCTTAAAGTTTTTTAAAGAAAAA
TTGTTCTTTTTAAGATTTGACTAATGTTACAAAGATACAAATCATTTATTTTACTACCTA
TAAACTGTTTCAGCCCACTGAAGAAAAGCTATGCTAATTTATATAGCTTTGACATTTTAT
AATTTTCATGATTTTTAGATAAAGGATTTTATATTTAAAACTTTAATAGTCTAGTCTGAG
TTCATTTATCTTTAATTTTTGAAACGGGTAGAGTAATTCATAACAGTAATTTTATTCCTG
TAATGTAATATTCACTTCAGTTCATTTGTGCATTAAAATTCCTTTAAGACCGTTATTAAT
GATTTATATAATGAAATGTCATATTAATCACAGAAGTTTTCACATTTTATTATTCTCTTC
CATGTTAATACTATCTTTATGATCTCTATTAATGTTTTATATCCTCTAATTTCAATTATA
AATCAAGAATATTTTCATGTTTTTGTTGGTATTTGCTTTTCAGGGGTGCCACCTTTAAAA
TTTTATGACTTTTTCCCTCCATATGCAATATAAAACATCTTTATTCATATAAAGGTAGTT
AATTATGTCTTTGTTTATAGTAAACTTTCATTGACAAACTTATAGTTATAATATTTCTTT
TCAAG

>SCE_mouse_sequence_1596400
TCAAGGAATTTACTTTTTAATCTTTTCAGTACATCTTAAAGTTTTTTAAAGAAAAATTGT
TCTTTTTAAGATTTGACTAAT

FUGU HITS IN THE CONTEST OF MAMMALIAN rCNE

```
CLUSTAL W (1.83) multiple sequence alignment


ENSG00000119487         GTGACCAAAGAATTTACTTTTTAATCCATGCAGCATATCTTAAAATTTTGTAAAAGAAAA
ENSCAFG00000020178      GTGACCAAAGAATGTACTTTTTAATCCTTTCAGCATATCTTAAAATTTTGTATAAGAAAA
ENSMUSG00000038696      GTGATCAAGGAATTTACTTTTTAATCTTTTCAGTACATCTTAAAGTTTTTTA-AAGAAAA
ENSRNOG00000017583      GTGATCAAGGAATTTAGTTTTTAATCTTTTCAGTACATCTTAAAAGTTTTTTAAAGAAAA
SINFRUG00000146615      ---------------------------------------------ATTTTTTTAAAACCCT
                                                                      *** *  **

ENSG00000119487         ATTGTTCTTTTTAAGATTTGACTAATGTTACAAAGATACAAATCATTTATTTTACTACCT
ENSCAFG00000020178      ATTATTCTTTTTAAGATTTGACTAATGTTACGAAGATACAAATCATTTATTTTACTACCT
ENSMUSG00000038696      ATTGTTCTTTTTAAGATTTGACTAATGTTACAAAGATACAAATCATTTATTTTACTACCT
ENSRNOG00000017583      ATTGTTCTTTTTAAGATTTGACTAATGTTACAAAGATACAAATCATTTATTTTACTACCT
SINFRUG00000146615      ATTGTACTTTTCTACATTTATTTAAT----------------------------------
                        *** * *****  * ****   ****

ENSG00000119487         ATAAACTGTTTCAGCCCACTGAAGAAAAGCTATGCTAATTTATATAGCTTTGACATTTTA
```

```
ENSCAFG00000020178      ATAAACTGTTTGAGCCCACTGAAGGAAAGCTATGCTAATTTATATAGCTTTGACATTTTA
ENSMUSG00000038696      ATAAACTGTTTCAGCCCACTGAAGAAAAGCTATGCTAATTTATATAGCTTTGACATTTTA
ENSRNOG00000017583      ATAAACTGTTTCAGCCCACTGAAGAAAAGCTATGCTAATTTATATAGCTTTGACATTTTA
SINFRUG00000146615      ------------------------------------------------------------


ENSG00000119487         TAATTTTCATGATTTTTAGATAAAGGATTTTATATTTAAAACTTTAATAGTCTAGTCTGA
ENSCAFG00000020178      TAATTTTCATGATTTTTAGATAAAGGATTTTATATTTAAAACTTTAATAGTCTAGTCTGA
ENSMUSG00000038696      TAATTTTCATGATTTTTAGATAAAGGATTTTATATTTAAAACTTTAATAGTCTAGTCTGA
ENSRNOG00000017583      TAATTTTCATGATTTTTAGATAAAGGATTTTATATTTAAAACTTTAATAGTCTAGTCTGA
SINFRUG00000146615      ------------------------------------------------------------


ENSG00000119487         GTTCATTTATCTTTAATTTTTGAAATGGGTAGAGTAATTCATAACAGTAATTTTATTCCT
ENSCAFG00000020178      GTTCATTTATCTTTAATTTTTGAAACGGGTAGAGTAATTCATAACAGTAATTTTATTCCT
ENSMUSG00000038696      GTTCATTTATCTTTAATTTTTGAAACGGGTAGAGTAATTCATAACAGTAATTTTATTCCT
ENSRNOG00000017583      GTTCATTTATCTTTAATTTTTGAAACGGGTAGAGTAATTCATAACAGTAATTTTATTCCT
SINFRUG00000146615      ------------------------------------------------------------


ENSG00000119487         GTAATGTAATATTCACTTCAGTTCATTTGTGCATTAAAATTCCTTTAAGACCGTTATTAA
ENSCAFG00000020178      GTAATGTAATATTCACTTCAGTTCATTTGTGCATTAAAATTCCTTTAAGACCGTTATTAA
ENSMUSG00000038696      GTAATGTAATATTCACTTCAGTTCATTTGTGCATTAAAATTCCTTTAAGACCGTTATTAA
ENSRNOG00000017583      GTAATGTAATATTCACTTCAGTTCATTTGTGCATTAAAATTCCTTTAAGACCGTTATTAA
SINFRUG00000146615      ------------------------------------------------------------


ENSG00000119487         TGATTTATATAATGAAATGTCATATTAATCACAGAAGTTTTCACATTTTATTATTCTCTT
ENSCAFG00000020178      TGATTTATATAATGAAATGTCATATTAATCACAGAAGTTTTCACATTTTATTATTCTCTT
ENSMUSG00000038696      TGATTTATATAATGAAATGTCATATTAATCACAGAAGTTTTCACATTTTATTATTCTCTT
ENSRNOG00000017583      TGATTTATATAATGAAATGTCATATTAATCACAGAAGTTTTCACATTTTATTATTCTCTT
SINFRUG00000146615      ------------------------------------------------------------


ENSG00000119487         CCATGTTAATACTATCTTTATGATCTCTATTAGTGTTTTATATCCTCTAATTTCAATTAT
ENSCAFG00000020178      CCATGTTAATACTATCTTTATGATCTCTATTAATGTTTTATATCCTCTAATTTCAATTAT
ENSMUSG00000038696      CCATGTTAATACTATCTTTATGATCTCTATTAATGTTTTATATCCTCTAATTTCAATTAT
ENSRNOG00000017583      CCATGTTAATACTATCTTTATGATCTCTATTAATGTTTTATACCCTCTAATTTCAATTAT
SINFRUG00000146615      ------------------------------------------------------------


ENSG00000119487         AAATCAAGAATATTTTCATGTTTTTGTTGGTATTTGCTTTTCAGGGGTGCCACCTTTAAA
ENSCAFG00000020178      AAATCAAGAATATTTTCATGTTTTTGTTGGTATTTGCTTTTCAGGGGTGCCACCTTTAAA
ENSMUSG00000038696      AAATCAAGAATATTTTCATGTTTTTGTTGGTATTTGCTTTTCAGGGGTGCCACCTTTAAA
ENSRNOG00000017583      AAATCAAGAATATTTTCATGTTTTTGTTGGTATTTGCTTTTCAGAGGTGCCACCTTTAAA
SINFRUG00000146615      ------------------------------------------------------------


ENSG00000119487         ATTTTATGACTTTTTCTCTACATATGCAATATAAAACATCTTTATTCATGTAAAGGTAGT
ENSCAFG00000020178      ATTTTATGACTTTTTCCCTACATGTGCAATATAAAACATCTTTATTCATATAAAGGTAGT
ENSMUSG00000038696      ATTTTATGACTTTTTCCCTCCATATGCAATATAAAACATCTTTATTCATATAAAGGTAGT
ENSRNOG00000017583      ATTTTATGACTTTCTCCCTCCATATGCAATATAAAACATCTTTATTCATATAAAGATAGT
SINFRUG00000146615      ------------------------------------------------------------


ENSG00000119487         TAATTATGTCTTTGTTTATGGTAAACTTTCATTGACAAACTTACAGTTATAATATTTCTT
ENSCAFG00000020178      TAATTATGTCTTTGTTTATAGTAAACTTTCATTGACAAACTTATAGTTATAATATTTCTT
ENSMUSG00000038696      TAATTATGTCTTTGTTTATAGTAAACTTTCATTGACAAACTTATAGTTATAATATTTCTT
ENSRNOG00000017583      TAATTATGTCTTTGTTTATAGTAAACTTTCATTGACAAACTTATAGTTATAATATTTCTT
SINFRUG00000146615      ------------------------------------------------------------


ENSG00000119487         TTCAAG
ENSCAFG00000020178      TTCAAG
ENSMUSG00000038696      TTCAAG
ENSRNOG00000017583      TTCAAG
SINFRUG00000146615      ------
```

Fragment Hmx3_11669

Representation of conserved regions within their genic context

Within the mouse context is shown the rCNE from which the tested SCE is derived (red diamond)

Within the fugu context all CHAOS hits obtained from the mouse rCNE are shown (green diamonds, in red the one tested)

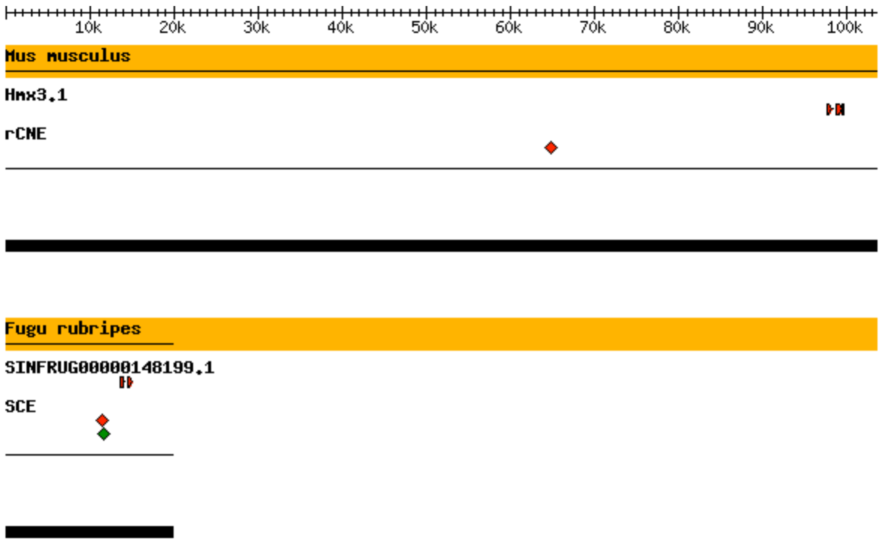

Conserved regions shown within 2000bp context

The mouse context contains:

-rCNEs green boxes, in red the one considered for this test

-SCEs yellow boxes, in red the one considered for this test

-CHAOS hits blue boxes, in red the one considered for this test

The fugu context contains:

-the fragment amplified in this test

-CHAOS hits blue boxes, in red the one considered for this test

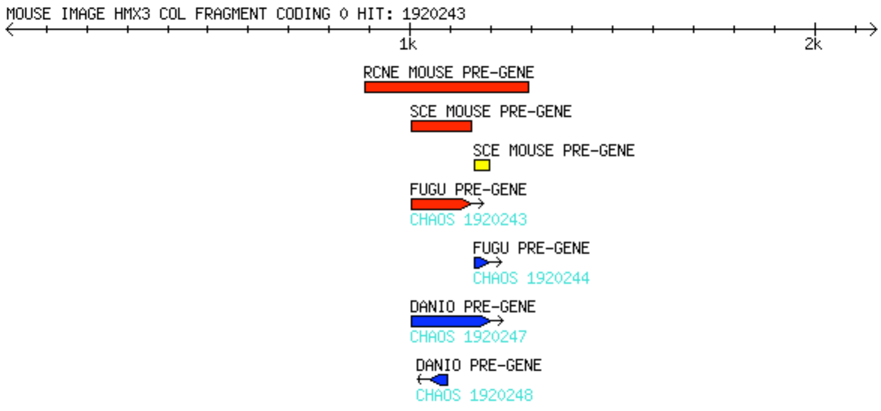

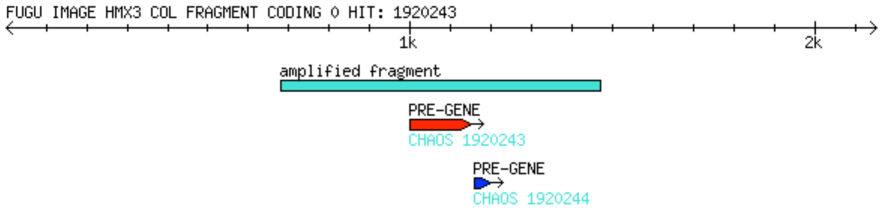

SEQUENCES

```
>fugu_fragment_for_primer_design_11669
CAAATGGAATATGAAGAGAGACAGCCAGGAATAACAAATGTCCACGCAATTAAAGAAGGA
GAATGACAAGAAGTTTGCCTTCATTCTTCACCTTAAAAATGCACTTTTTTTCCTGTTTAT
ACAGGAAGTCAAGTGGCAACTAAAGTGCACCAACCTGGATGACATTGAGCTCATTCGACT
CGCTGGCGCGAGGGCTCGCGTAAAAGCCAACTGGGTCGTAAGTCAGGATTCACCTGGACA
CCCACAATCAATACCTGCACAATCCCAATTAAAGCGTGCCGGGTCGGTGGAGGGTCACCA
ATTCCCTTTCAAGTTAAAAGCAATACTCAAATCGAAACAATCTCGGCGTGGCCTTCGCTG
GTTTAATGGCGCGAACCAACAGCGTGTCCGGTTAAAAATGGAAGAGAGGCAGGTGAGGGT
GGACGAAGTCCGATGCTGAGAGCTTCCAATTCAGCACGTAAAGTCATGAAGACAGAGTAA
ATCTAGCGGGGAAAGAAGGTTATTTTACAGGCTGGTTGTTGTTTGTTGGGTCAGTTATAA
TTATGCAAACTTTGCAAAAATGGAATATCCTTCGACTGTTTCTTGGCCTGAAATCACGCT
TTAACTGAAGCTGCAGGAACTGAAACGTCTTTACATTTCAAATTGGATGCAGCTTCTGAT
AAGATCTTCACGTTCTCATGGCTTTTGTCCATTCTGTGACTGGAATATTGCTCCCTTCAT
TAAACAGGTTCTGAATGCTTCATCACAACTCCAAGATCAGCCTCTCTCAGCTCACATGTG
CCCGCCTGTCTTTTCTTTTCTATTCTTCCCTCCAGCTTTTTCCCCCCCTCTTGTGAGCCT
TCGCCATCCCATCCAAGTCTTTTATCACAATTCTGACCCACAGAAAAGAATTCTCACAAG
ATATCAAAACAAAAATCTATATTTAATATGGGAGCAATTACAGAGCGAAGTTGGCTGCGC
GGGGCCGTTGTCACCAGAGCGCGTGTTTTTCATCAAACAGGTCATTAGACCATGATGGAT
GTAAACCTATAAAGTATCTAAACTGGTCCTTTTAACAAACCATGAGCAACACTATCATTT
CTCTAATAGAACCTGCTGACCGTGTCATTCCCCGCTGCCATTCGTCATCTTTCCTTGTGA
AATAATCAATAGCTGGCTGCTTTGAAATAAATAATATAAAAGTCATAGTAACCTACCTAA
GGAGCAACAAAAATGGATTTATGGAGCATCCAAGAGACTTGGGGCAGCATATATGGCACA
ACTATTTTTAAAATATCTACAATTTTGAAGATGCAGGATTTTTGAGGCTTATTTCATTGC
AATTAAATAAGAAGATTTCAGTGGTTCGTAAACTCATTGGGACACGTCTCACCTCGATTC
GAGGCCTGATATACTCACTTCATGTGATCATTTTCTAATAAAGCACAATTTAGGAACATA
ATTTACACAACAGAATGTTGGGTTAGGGATAGAAAATACTTTAATGTTAATTGACATACT
GTAGATCATCGTCTGCAAAGAGAGAATAGGTGGAAAGTTACTCAGTGTTTGCANNNNNNN
NNNNNNNNNNNNNNNNNNNNAGTATCAGCTGTATTAGGAAAGGCAGTAATTACCTGACAG
TAACAGGATATCCTAATTTGAGCTGCCCACTACCTGAATAACACATGTTAATAACATGTT
TAATACTGTATGTCACAACTGCAGACTGGAATATATGTGTGGAGGAGAGGGGGTGTTAAT
GTTGGAGAATTTAGTCTAGTAATCCATCTAAAGTCGTGTGTTTTTGGAATGCAGATTTTT
TCGGGGGAATTTTTTTGCTTTCATGTGTTTCTTTACGTGTTGGTCCCCTACTTGCAGTAC
CCACACTTAACCCACAGAGGGCGCTGTCCCACCGCCATTCTTGCACGCCAGAAGATTTTT
AACAAGTCAATTTATATCACTATGTGCATACATAAAACGTTTGTAAGGATGAATTATCCA
```

```
CATCGATTAGAGAGGAATTAAAGTCCACCGTGAGGAAGTGACCGCATGAAAAAAATTGAC
TAATCAAAAATATGAATTTGATCGTCATTTAGAGCTAAAAATTAAAATTGGACCTTTTTC
AGATTATTTTTGCAAATCTGANNNNNNNNNAGGCCTTTAGACGCCACATTT
```

```
>amplified_fugu_segment
TGGCTTTTGTCCATTCTGTGACTGGAATATTGCTCCCTTCATTAAACAGGTTCTGAATGC
TTCATCACAACTCCAAGATCAGCCTCTCTCAGCTCACATGTGCCCGCCTGTCTTTTCTTT
TCTATTCTTCCCTCCAGCTTTTTCCCCCCCTCTTGTGAGCCTTCGCCATCCCATCCAAGT
CTTTTATCACAATTCTGACCCACAGAAAAGAATTCTCACAAGATATCAAAACAAAAATCT
ATATTTAATATGGGAGCAATTACAGAGCGAAGTTGGCTGCGCGGGGCCGTTGTCACCAGA
GCGCGTGTTTTTCATCAAACAGGTCATTAGACCATGATGGATGTAAACCTATAAAGTATC
TAAACTGGTCCTTTTAACAAACCATGAGCAACACTATCATTTCTCTAATAGAACCTGCTG
ACCGTGTCATTCCCCGCTGCCATTCGTCATCTTTCCTTGTGAAATAATCAATAGCTGGCT
GCTTTGAAATAAATAATATAAAAGTCATAGTAACCTACCTAAGGAGCAACAAAAATGGAT
TTATGGAGCATCCAAGAGACTTGGGGCAGCATATATGGCACAACTATTTTTAAAATATCT
ACAATTTTGAAGATGCAGGATTTTTGAGGCTTATTTCATTGCAATTAAATAAGAAGATTT
CAGTGGTTCGTAAACTCATTGGGACACGTCTCACCTCGATTCGAGGCCTGATATACTCAC
TTCATGTGATCATTTTCTAATAAAGCACAATTTAGGAACATAATTTACACAACAGAATGT
TGGGTTAGGGATA
```

```
>rCNE_mouse_sequence_1645923
TCACTGCCAACCCATTCCCCCACCAAAATGGAGTGTCTTATTTTGCTAACTGCTTAAACA
TAAAACTCCTGCAGNNNNNNNNNNNTTGTCCCCACAGCTCATATTTTTCATCATTTGATCA
TTAAATAGTGATGGATATAAACCTATAAAGATCCTAAACTGGAGCTTTTAACAATCCACG
AGCAATGCTATCATTTCTTTAATAGAACCTGCTGACCGTGCCTTGCACAGACACTATTTG
TCATTTTCTCCACGCAATAATCAATAAGTTGGCTTGAAAATAAATAATATAACAGTCACA
ATATATTATCTTTCAGACTGGCCTTAGACTAACCATAAAACGGGACCAAAACAAGAAAGA
AACTTTTACATAAATGTTAAAAAGTTATGAACATCACAGTTGGGCCTA
```

```
>SCE_mouse_sequence_1645954
GATCATTAAATAGTGATGGATATAAACCTATAAAGATCCTAAACTGGAGCTTTTAACAAT
CCACGAGCAATGCTATCATTTCTTTAATAGAACCTGCTGACCGTGCCTTGCACAGACACT
ATTTGTCATTTTCTCCACGCAATAATCAATAA
```

FUGU HITS IN THE CONTEST OF MAMMALIAN rCNE

```
CLUSTAL W (1.83) multiple sequence alignment


ENSRNOG00000020637      TCACCACCACCCCATTCCCCCACCAAAATGGAGTGTCTTATTTTGCTAACTGCTTAAACA
ENSMUSG00000040148      TCACTGCCAACCCATTCCCCCACCAAAATGGAGTGTCTTATTTTGCTAACTGCTTAAACA
ENSG00000188620         TTGCCACTTCCCCATGCCCCCACCAAAATGGAGTATCTTATTTTGCTAACTGCTTAAACA
ENSCAFG00000012698      TTGCCGCCACCCCACTGCCCCACCAAAATGGAGTATCTTATTTTGCTAACTGCTTAAACA
SINFRUG00000148199      ------------------------------------------------------------


ENSRNOG00000020637      TAAAACTCCCGCAGNNNNNNNNNTT--GTCCCCACAGCTCATATTTTTCATCATTTGATCA
ENSMUSG00000040148      TAAAACTCCTGCAGNNNNNNNNNNNTTGTCCCCACAGCTCATATTTTTCATCATTTGATCA
ENSG00000188620         TAAAACTCCAGAAGAAAAAAAATT---GTCCCCACAGCTCATATTTTTCATCATTTGGTCA
ENSCAFG00000012698      TATAACTCTAACAGAAAAAAAATT---GTCCCCGTGGCTCATATTTTTCATCATTTGGTCA
SINFRUG00000148199      -------------------------------------------------------GGTCA
                                                                               * ***

ENSRNOG00000020637      TTAAATAGTGATGGATATAAACCTATAAAGATCCTAAACTGGAGCTTTTAACAATCCATG
ENSMUSG00000040148      TTAAATAGTGATGGATATAAACCTATAAAGATCCTAAACTGGAGCTTTTAACAATCCACG
ENSG00000188620         TTAAATAGTGATGGATATAAACCTATAAAGAACCTAAACTGAACCTTTTAACAATCCATG
ENSCAFG00000012698      TTAAATAGTGATGGATATAAACCTATAAAGAACCTAAACTGAACCTTTTAACAATCCATG
SINFRUG00000148199      TTAGACCATGATGGATGTAAACCTATAAAGTATCTAAACTGGTCCTTTTAACAAACCATG
                        *** *   ******** *************   ********   ********** *** *

ENSRNOG00000020637      AGCAATACTATCATTTCTTTAATAGAACCTGCTGACCGTGCCTTGCACAGACACTATTTG
ENSMUSG00000040148      AGCAATGCTATCATTTCTTTAATAGAACCTGCTGACCGTGCCTTGCACAGACACTATTTG
ENSG00000188620         AGCAATACTATCATTTCTTTAATAGAACCTGCTGACGGTGCCGCGCGCAGACACCATTTG
ENSCAFG00000012698      AGCAATACTATCATTTCTTTAATAGAACCTGCTGACCGCGCCTCGTGCAGACACCATTTG
SINFRUG00000148199      AGCAACACTATCATTTCTCTAATAGAACCTGCTGACCGTGTCATTCCCCGCTGCCATTCG
                        *****  *********** **************** * * *      * *   * *** *
```

```
ENSRNOG00000020637      TCAT-TTTCTCCACGCAATAATCAATAAGTTGGCTCGAAAATAAATAATATAACAGTCAC
ENSMUSG00000040148      TCAT-TTTCTCCACGCAATAATCAATAAGTTGGCTTGAAAATAAATAATATAACAGTCAC
ENSG00000188620         TCAT-TTTCAGCACGCAATAATCAATAAGTTGGCTTGAAAATAAATAATATAACAGTCAC
ENSCAFG00000012698      TCAT-TTTCAGCACGCAATAATCAATAAGTTGGCTTGAAAATAAATAATATAACAGTCAC
SINFRUG00000148199      TCATCTTTCCTTGTGAAATAATCAATAG--------------------------------
                        **** ****     * ***********

ENSRNOG00000020637      AATATATTATCTTTCAGACTAGCCTTAGACTAACCATAAAATGGGACTAAAACAAGAAAG
ENSMUSG00000040148      AATATATTATCTTTCAGACTGGCCTTAGACTAACCATAAAACGGGACCAAAACAAGAAAG
ENSG00000188620         AATATATTATCTTTCAGACTGGCCTTATACTAACCATAAAATGGGACTAAAACAAGACAG
ENSCAFG00000012698      AATATATTATCTTTCAGACTGGCCTTATACTAACCATAAAATTGGACCAAAATAAGAGGA
SINFRUG00000148199      ------------------------------------------------------------


ENSRNOG00000020637      AAACTTTTACGTAAATGTTAAAAAGTTATGAACGTCACAGTTGGGCCTA
ENSMUSG00000040148      AAACTTTTACATAAATGTTAAAAAGTTATGAACATCACAGTTGGGCCTA
ENSG00000188620         AAACTTTTACATGAATGTTAAAAAGTTAGAGACATCACAGTGAGGACTA
ENSCAFG00000012698      AAACTTTTACATAAATGTTAAAAAGTTAGGAACTTCACAGCGGGGCCTA
SINFRUG00000148199      -------------------------------------------------
```

Fragment Pax3_24781

Representation of conserved regions within their genic context

Within the mouse context is shown the rCNE from which the tested SCE is derived (red diamond)

Within the fugu context all CHAOS hits obtained from the mouse rCNE are shown (green diamonds, in red the one tested)

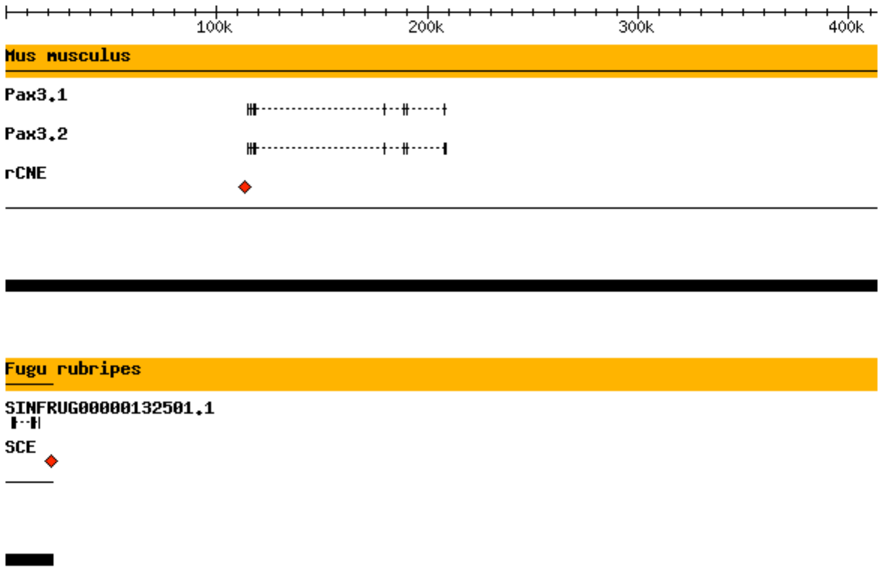

Conserved regions shown within 2000bp context

The mouse context contains:

-rCNEs green boxes, in red the one considered for this test

-SCEs yellow boxes, in red the one considered for this test

-CHAOS hits blue boxes, in red the one considered for this test

The fugu context contains:

-the fragment amplified in this test

-CHAOS hits blue boxes, in red the one considered for this test

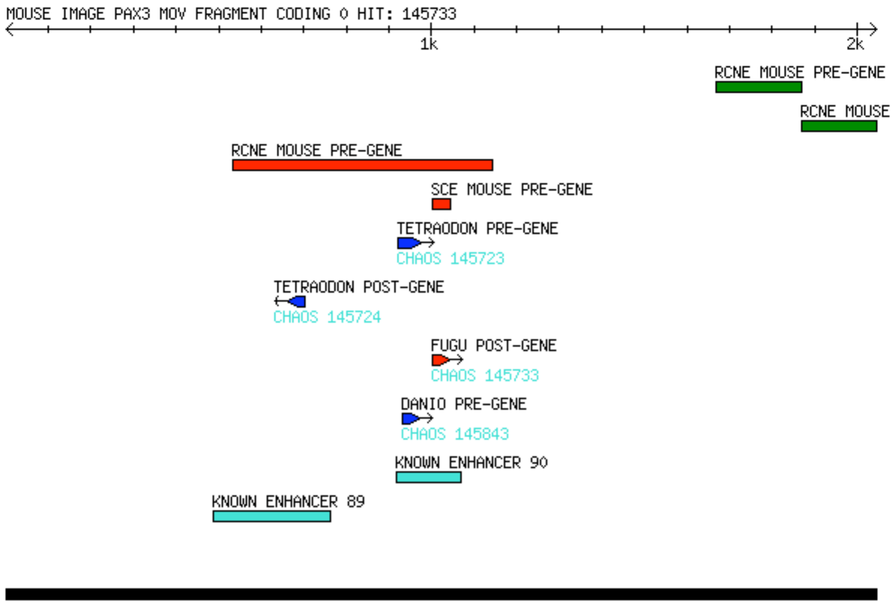

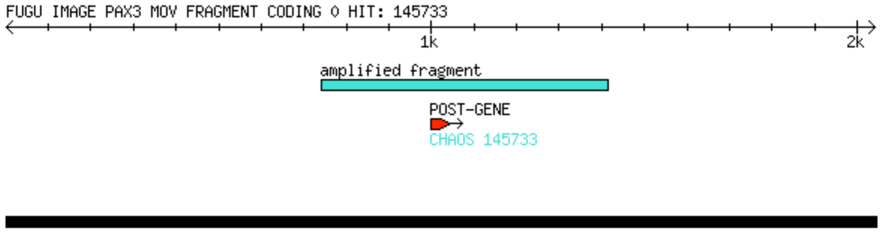

SEQUENCES

>fugu_fragment_for_primer_design_24781
TCAGGGAGGTCAGAGGTGAGTCTGGGGTGACACCGCAGGCCTAATTTGAACTATCCGCTC
CCAGGTTGTGCCGGTCCCCGAGGGCATGTGCGGAGATGTTTCCTCTCCTTCCCTCGTAAT
TAAATCTGTGTGTCATCCTCCTCCGCCCCGCAGACGCGCGGATCCCCATCGAGCCCGGCG
ACGCGCAGCAGATCAGCGCTGCGTGAATTATACGCGGGGTTTAGGTCACAATGTTTGCCT
CATCAGTATGACACGACAGCCCAAAGTTATAGAGTTAAAAACAACCCCAAAACACACGCA
CGCGTGCACGCTCAGAGTTCTACCGCAGCAAATCGAACGAACACATGACAACTTGCGCAA
CCTGAGGGGAAGACGCGGGGAAAGCTGTTATGATAAATAGCTGGTTTGTATTCCCGCAGG
GAAACACAAAACTAAATCTAAAAGTCACCTGTGCGTGACCGGTGCGGCATGTCCATCATT
TGAAATGATTCGTTTTGGAGGAAAACACGGGCTTGTTTTTAAATAGGACGAATTGGGCAA
AAAAGGGTGTTCTTATGGTCAAACTTTATGTAAATGGACCTGTCTGCTTTATTGTCATCA
ACAGAAAAAAGTATCCAAAAATACTTGCTGCTGTTCAAAATAACATAGGCTATAGAGTAT
ATACTTTNNNNNNNNNGACATTTAAATGATAAAATCTGCTTATGATCACAAATAAGTCAC
ATTAGCTTATCTGTCTTCATGCATACCCCTGGGTTAAAAGGCCTTTTTATAATTGGAAGA
ATAAATAAATCAGTTCTTCGTNTGGCNAAATGGCTAATGAAGCTGTGACTGTACCGCCCC
CTGGTGGTCACGATGGCGCTAGCACTAAAAAGACTCCCATTATTGGACGGTTGTCGCCAT
CTACAGGTCGGCCTGAGGTACTGTTTCAACAGGAATGATTGCTTTTACCAGTGCACCTCT
GATTGAGGAGTTAATGAAGCTCCCATATTATCATTAGATTTGTTTATTATATTTATAAGT
CAGTGTAGTCTGAAATTGAGAATATGATGTATAAAATTGAATTAATAATACGTTTATTTT
TCAAAAATTAAACGAGTAAACCATTAAGTTATAGTAATATATTGTGGTTATCTCATAAAG

```
ACAGGGGAAATAACAATGATAAAAATAACAAACAATCAAATTAGAAGACTTAGTTGATAA
TTGTTTTTCTTTAAAGATTTGGCAATATTTGCTTTAAGCAACCAAGTGTACTTTAGGTTT
TGGTAAATCCATATTGTTACTGTTTGGCTGTTACCGCAACATTTCTGTAATTCAGTTATC
AATTGGATTCAAATTGAATAATCATTTTCAATTATGCACAGTTCAGATTAATTAATTTAA
AAAGGCATCTTTACTTACCGCTGTTCAGCAGCGATTTTCAATTTTTCTTCGTAAAACTTT
TCAATTTATGTGCAATTTAGCAGTCGTATTATCACTTCTACTCCTCACGACTATGTAAAA
TCTAAGCATTGCTGCCCCCTGCTGTTAAAATGCCGTCACTGCACCATTATATCTGGTGCT
GCTATTTTAATGACAGGAAATACACTCAAAATGAAAAATGAACAATTTGGCTCTTTTTCT
TTCCAGGATAATCACTTTTAATAATATCACTTTTAAGTAGGACACAGCTAAATTCAAGCT
TGTATGCCATAAATCTTTAATTATTGCCATCATTAAAAGAATTTGCCATTACGAAGCACA
TTATCTGTTCATTAGAGTTCATTTTACAGAATCAGTTTTGGNNNNNNNNNNNNNNNNNNN
NNNNNNNNNNNNNTGGGTTCATTTATTTAAAATGCACATAAAGCTTCTCACCCTCAACAA
TAGCAGGTTGAGGCAATCGAGACAGATTTTAAAAGTGCACTTGGTCATCAGATTTAGAAT
GATCTCTCTATATGAGTTGAGGNNNNNNNNNNNNNNNNNNNNNNNNNNNNNNNNNNNNNN
NNNNNNNNNNNNNNNNNNNNNNNNNNNNNNNNNNNNNNNNNNNNNNNNNNNNNNNNNNNN
NNN
```

```
>amplified_fugu_segment
ATACCCCTGGGTTAAAAGGCCTTTTTATAATTGGAAGAATAAATAAATCAGTTCTTCGTN
TGGCNAAATGGCTAATGAAGCTGTGACTGTACCGCCCCCTGGTGGTCACGATGGCGCTAG
CACTAAAAAGACTCCCATTATTGGACGGTTGTCGCCATCTACAGGTCGGCCTGAGGTACT
GTTTCAACAGGAATGATTGCTTTTACCAGTGCACCTCTGATTGAGGAGTTAATGAAGCTC
CCATATTATCATTAGATTTGTTTATTATATTTATAAGTCAGTGTAGTCTGAAATTGAGAA
TATGATGTATAAAATTGAATTAATAATACGTTTATTTTTCAAAAATTAAACGAGTAAACC
ATTAAGTTATAGTAATATATTGTGGTTATCTCATAAAGACAGGGGAAATAACAATGATAA
AAATAACAAACAATCAAATTAGAAGACTTAGTTGATAATTGTTTTTCTTTAAAGATTTGG
CAATATTTGCTTTAAGCAACCAAGTGTACTTTAGGTTTTGGTAAATCCATATTGTTACTG
TTTGGCTGTTACCGCAACATTTCTGTAATTCAGTTATCAATTGGATTCAAATTGAATAAT
CATTTTCAATTATGCACAGTTCAGATTAATTAATTTAAAAAGGCATCTTTACTTACCGCT
GTTCAGCAGCG
```

```
>rCNE_mouse_sequence_149451
GTTTGCCTTAATTAGGGGGTGGGCAAGTAGGATTGGGGGCCTGAGTTCACATCAAAACTG
TCAAAGGAGATNNNNNNNNNNAGGACATGGAGAGAGGGTTGGAGGATTCAGGGGCAGGAA
AAATTCAAATATCATAAAAAGAAAAGGGAACTTGAAGCCCAGAAGTCATTGTTTCCTTTA
TTTCAAAGGGGAAAAAACCTGCCTGATTCTCATCCTTTTGATTGATTAAGGCCCTGAATA
CCTCTCGGCCCCAGAGTGACAGTCCCTGAATAGACTTGAGGGAATAAAGAGCAGCGCAGA
ACGTGGGGCTGGCACGGGAGGGGGGTGTAAGGGAGGAGTGTTCGCTGGCACTTACCAAGT
TATAAATAAAAGGCTAGGCACAATGGTACCTTCTCTAAGGACAGACAGTCTTTACAACAC
TCCTGGCGTCATATCCTGCTGGGGACACTTCAGCTCCTAGCCAAGACGTTGCTTCTTTTA
TTTTTCCAGCAGTTTAGTCTGAATGCCATAATAAATTCCTGAGAACAAACGCTGCACCCG
GGCAAAACCTCAACATATAGATGCAAGTGCATCGGGGATGAATGTGTACGTGGAGATTTA
AAGTCCCCGCTT
```

```
>SCE_mouse_sequence_149489
TTGCTTCTTTTATTTTTCCAGCAGTTTAGTCTGAATGCCATAATA
```

FUGU HITS IN THE CONTEST OF MAMMALIAN rCNE

```
CLUSTAL W (1.83) multiple sequence alignment


ENSG00000135903        GTTTGCCTTAATTAGGGAATGGGGAAGTAGGGTTGGGGGCCCGAGTTCACGTCAAAACTG
ENSCAFG00000016041     GTTTGCCTTAATTAGGGGGTGGGGAAGTGGGGTTGGGGGCC-GAGTTCACGTAGAAACTG
ENSRNOG00000013670     GTTTGCCTTAATTAGGGGGTGGGCAAGTAGGATTGGGGGTCTGAGTTCACATCAAAACTG
ENSMUSG00000004872     GTTTGCCTTAATTAGGGGGTGGGCAAGTAGGATTGGGGGCCTGAGTTCACATCAAAACTG
SINFRUG00000132501     ------------------------------------------------------------


ENSG00000135903        TCAAA-GGAGGTGGGGGA-GGGGGACAGAGAGAGAGGAGTGGAGAACTCCCTCCAGGGGC
ENSCAFG00000016041     TCAAAAGGAGTTGGGGGATGGGAAACTGAGAGAGGGGGGTGGTGGAGAAC--TCTGGCCC
ENSRNOG00000013670     TCAAAGGAGATNNNNNNNNAA-GGACATAGAAAGAGGGGTGGAGGAT-----TCAGGGGC
ENSMUSG00000004872     TCAAAGGAGATNNNNNNNNNNAGGACATGGAGAGAGGGTTGGAGGAT-----TCAGGGGC
SINFRUG00000132501     ------------------------------------------------------------
```

```
ENSG00000135903          AGGAAAAATTCAAAAATCATAAAAGGAAAAGGGGGAACTTGAAGCCCAGAAGTCATTGTT
ENSCAFG00000016041       AGGAAAAATTCAAAAATTGTAAAAGGAAAAGGGGGAACTTGAAGCCCAGAAGTCATTGTT
ENSRNOG00000013670       AGGAAAAATTCAAATATCATAAAAGGAAAAGG--GAACTTGAAGCCCAGAAGTCATTGTT
ENSMUSG00000004872       AGGAAAAATTCAAATATCATAAAAAGAAAAGG--GAACTTGAAGCCCAGAAGTCATTGTT
SINFRUG00000132501       ------------------------------------------------------------


ENSG00000135903          TCCTTTATTTCAAAGGG--AAAAACCTGCCCGATTCTCATCCTTTTGATTGATTAAGGCC
ENSCAFG00000016041       TCCTTTATTTCAAAGGG--AAAAACCTGCCCGATTCTCATCCTTTTGATTGATTAAGGCC
ENSRNOG00000013670       TCCTTTATTTCAAAGGG-AAAAAACCTGCCTGGTTCTCATCCTTTTGATTGATTAAGGCC
ENSMUSG00000004872       TCCTTTATTTCAAAGGGGAAAAAACCTGCCTGATTCTCATCCTTTTGATTGATTAAGGCC
SINFRUG00000132501       ------------------------------------------------------------


ENSG00000135903          CTGAATACCTCGCGGCCCCGGAGTGACAGTCCCTGAATAGGCTCGAGCGAATAAAGCGCA
ENSCAFG00000016041       CTGAATACCTCGCAGCCCCAGAGTGACAGTCCCTGAATAGACTCGAGCGCATAAAGCGCA
ENSRNOG00000013670       CTGAATACCTCTCGGCCCCAGAGTGGCAGTCCCTGAATAGACTTGAGGGAATAAAGAACA
ENSMUSG00000004872       CTGAATACCTCTCGGCCCCAGAGTGACAGTCCCTGAATAGACTTGAGGGAATAAAGAGCA
SINFRUG00000132501       ------------------------------------------------------------


ENSG00000135903          GTGCAGAGCGCGGGGCTGGCACTCGGGGG----TGTAAAGGAGGCGAGTTCGCTGGCACT
ENSCAFG00000016041       GTGCAGAGCGCGGGGCTGGCACTCGGGGG----TGTAAAGGAGGCGAGTTCGCTGGCACT
ENSRNOG00000013670       GCGCAGAACGCGGGGCTGGCACGGGAGGGGGGGTGTGAAGGAGGAGAGTTCGCTGGCACT
ENSMUSG00000004872       GCGCAGAACGTGGGGCTGGCACGGGAGGGGGG-TGTAAGGGAGGAGTGTTCGCTGGCACT
SINFRUG00000132501       ------------------------------------------------------------


ENSG00000135903          TACCAAGTTATAAATAAAAGGCTATGCACAATGGTACCTTCTCTAAGGACAGACAGTCTT
ENSCAFG00000016041       TACCAAGTTATAAATAAAAGGCTATGCACAATGGTACCTTCTCTAAGGACAGACAGTCTT
ENSRNOG00000013670       TACCAAGTTATAAATAAAAGGCTAGGCACAATGGTACCTTCTCTAAGGACAGACAATCTT
ENSMUSG00000004872       TACCAAGTTATAAATAAAAGGCTAGGCACAATGGTACCTTCTCTAAGGACAGACAGTCTT
SINFRUG00000132501       ------------------------------------------------------------


ENSG00000135903          TACAACACTCCTGGCGTCATATCCTGCTGGGGACACTTCAGCTCCTAGCCAAGACTTCGT
ENSCAFG00000016041       TACAACACTCCTGGCGTCATATCCTGCTGGGGACACTTCAGCTCCTAGCCAAGACTTCGC
ENSRNOG00000013670       TACAACACTCCTGGCGTCATATCCTGCTGGGGACACTTCAGCTCCTAGCCAAGACTTTGC
ENSMUSG00000004872       TACAACACTCCTGGCGTCATATCCTGCTGGGGACACTTCAGCTCCTAGCCAAGACGTTGC
SINFRUG00000132501       --------------------------------------------------------TTGT
                                                                                 * *

ENSG00000135903          TCCTTTTATTTTTCCAGCAGTTTAGTCTGAATGCCATAATAAATTCCTGAGAACAAACGC
ENSCAFG00000016041       TCCTTTTATTTTTCCAGCAGTTTAGTCTGAATACCATAATAAATTCCTGAGAACAAACGC
ENSRNOG00000013670       TTCTTTTATTTTTCCAGCAGTTTAGTCTGAATGCCATAATAAATTCCTGAGAACAAACGC
ENSMUSG00000004872       TTCTTTTATTTTTCCAGCAGTTTAGTCTGAATGCCATAATAAATTCCTGAGAACAAACGC
SINFRUG00000132501       TTATTATATTTATAAGTCAGTGTAGTCTGAAATTGAGAATA-------------------
                         *  ** ***** *    **** *********    * ****

ENSG00000135903          TGAACCCGGGCAAAACTTTAACATACAGACACATCTCTGTCGACGCATCGGGGATC----
ENSCAFG00000016041       TGCAGCCGGGCAAAACTTTAACATATAGACACA-------CGCCACATTGGGGATC----
ENSRNOG00000013670       TGCACCGGGGCAAAACCTCAACGTATAGTTGCA--------AGTGCATCGGGGATCAAAC
ENSMUSG00000004872       TGCACCCGGGCAAAACCTCAACATATAGATGCA--------AGTGCATCGGGGATGAATG
SINFRUG00000132501       ------------------------------------------------------------


ENSG00000135903          TATATGTAGAGATTTAGAACCGCAGCTT
ENSCAFG00000016041       TATACCTCGAGGTTTAGAGTTGGGGCTT
ENSRNOG00000013670       TGTATGTGGAGATTTAAAGTCCCCGCTT
ENSMUSG00000004872       TGTACGTGGAGATTTAAAGTCCCCGCTT
SINFRUG00000132501       ----------------------------
```

Fragment Gata3_15402

Representation of conserved regions within their genic context

Within the mouse context is shown the rCNE from which the tested SCE is derived (red diamond)

Within the fugu context all CHAOS hits obtained from the mouse rCNE are shown (green diamonds, in red the one tested)

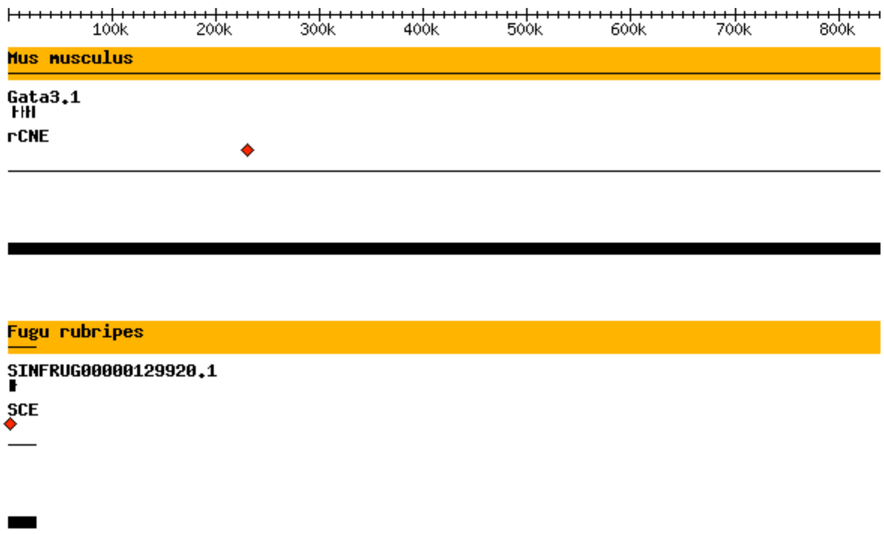

Conserved regions shown within 2000bp context

The mouse context contains:

-rCNEs green boxes, in red the one considered for this test

-SCEs yellow boxes, in red the one considered for this test

-CHAOS hits blue boxes, in red the one considered for this test

The fugu context contains:

-the fragment amplified in this test

-CHAOS hits blue boxes, in red the one considered for this test

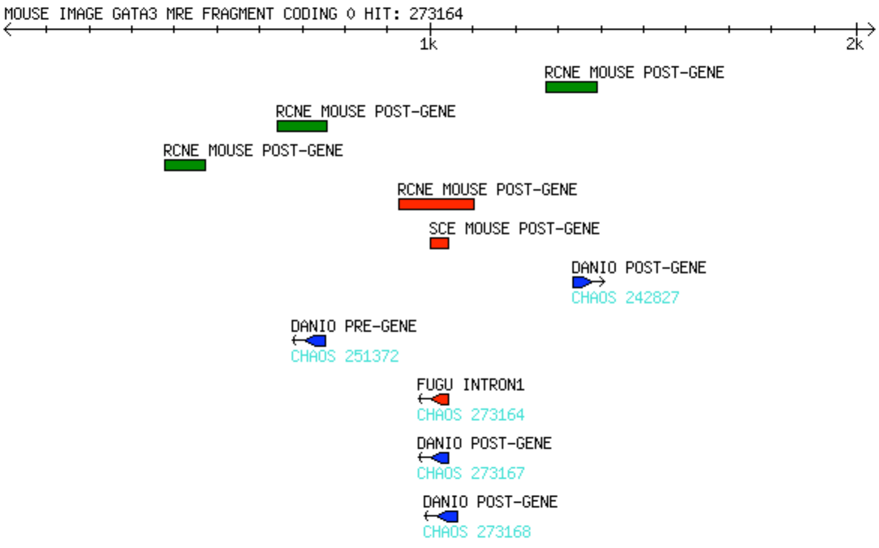

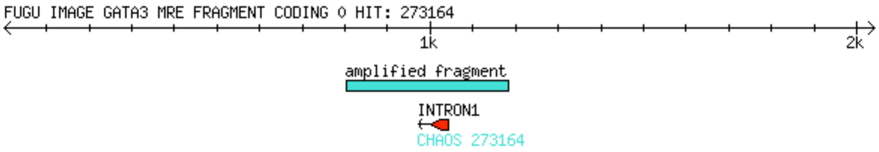

SEQUENCES

```
>fugu_fragment_for_primer_design_15402
TGACTCGCTGCTGCCGGTCAGTGCGCACCGAAACCAAGAGCCAAACGCGCAGCGAAGCTC
CGCTCCGGCCTGAAAACTCGATTCTTTTNNNNNNNNNNNNNNNNNNNNGAAATTCCTCGT
TTTCAAAGAAGAAGAAAGTTGAGCAACTGCGCGGGACTGGATCCCAGTTTGGACGTATTT
ATATATCAACAGCGTTGTTGCATTTTGTCATGTGGGGTATGTTTCCTGTAAATATCTGCT
TTTGCTGCATCTGCGCGTAAACTCAGAGAAAGNNNNNNNNNNNNNNNNNNNNNNNNNNNN
NNNNNNNNNNNNNNNNNNNNNNNNNNNNNNAAATAAAAAGAATTAAGACAGTGAATTTGT
GCCTCTGTTTGTGCAGGTGAGCAGAACCAACGGCGGAACCTGTGGATTTAACTTTGAACA
CTTCGGTCGGATTTGGGACTCTCGGCTTCTTTGCCGCGCTGCCGCGCTGCTTTGAGTCGC
GGTGCACGAACGTTTGCCGCGTGCAGAAGTGAACATTTGAACTATTAAAGAGAGAAATGX
XXXXXXXXXXXXXXXXXXXXXXXXXXXXXXXXXXXXXXXXXXXXXXXXXXXXXXXXXXXX
XXXXXXXXXXXXXXXXXXXXXXXXXXXXXXXXXXXXXXXXXXXXXXXXXXXXXXXXXXXX
XXXXXXXXXXXXXXXXXXXXXXXXXXXXXXXXXXXXXXXXXXXXXXXXXXXXXXXXXXXX
XXXXXXXXXXXXXXXXXXXXXXXXXXXXXXXXXXXXXXXXXXXXXXXXXXXXTAAGTCCG
CGGCCTGTTCGGGCCTTTTCCCCGGTATCTGTGCGCCTTTTCTCGGTCGGAGCCACATGA
AGCTCCGCTGTGCCTTGAACGGTTTCTGTCTAAACGCCACGAACGTGAAATGCCAGTTAG
CTCCTATCAAAGCTGAAAACACACTCTCTCTCGCCCGCACACGCGTGCGCGCAATTCCTC
CGTATCAGTGTTAGTTTCGGACGCACCTGCTTTACTTTTTATTCACTTGGCTTTAATTTA
AACGCTAATTTTTTATCTCAAAGCGGACACGTTTTTGCATATTTTCCCTACATGCAGTAN
NNNNNNNNCGTTTTACTTCCTCAACAGTCGATCACGCCTCCGTTTCTGCAGAGGCAGATT
TAAATATTTGTTCAGATCGATTCTCGCTGCAAATTCAAATCTGTGCGTAAAAGAAAATCT
CCGCTTTCTGCTCTCAATTAAATTCTGACGGCGATGTTTNNNNNNNNNNNAGGXXXXXXX
XXXXXXXXXXXXXXXXXXXXXXXXXXXXXXXXXXXXXXXXXXXXXXXXXXXXXXXXXXXX
XXXXXXXXXXXXXXXXXXXXXXXXXXXXXXXXXXXXXXXXXXXXXXXXXXXXXXXXXXXX
XXXXXXXXXXXXXXXXXXXXXXXXXXXXXXXXXXXXXXXXXXXXXXXXXXXXXXXXXXXX
XXXXXXXXXXXXXXXXXXXXXXXXXXXXXXXXXXXXXXXXXXXXXXXXXXXXXXXXXXXX
XXXXXXXXXXXXXXXXXXXXXXXXXXXXXXXXXXXXXXXXXXXXXXXXXXXXXXXXXXXX
XXXXXXXXXXXXXXXXXXXXXXXXXXXXXXXXXXXXXXXXXXXXXXXXXXXXXXXXXXXX
XXXXXXXXXXXXXXXXXXXXXXXXXXXXXXXXXXXXXXXXXXXXXXXXXXXXXXXXXXXX
XXXXXXXXXXXXXXXXXXXXXXXXXXXXXXXXXXXXXXXXXXXXXXXXXXXXXXXXXXXX
XXXXXXXXXXXXXXXXXXXXXXXXXXXXXXXXXXXXXXXXXXXXXXXXTAGGCGTGCGC
```

TTTATTTCATTCCCTTTAATCTCACTCTGCATTCCCGAGATCACCCGGTTCCATTAATAC
GAGTCTAAAATTAGCGAATTNNNNNNNNNCAACCGATAGTTTCAGCGGGTTTATTTATTT
ATTTTTTATATTCAGATGATTTAATTCCCCTGCTACGAATTGAAATCAGCAGGTTTGGTG
CACAGTTGAACGCCTGCATTATTTCAGCCTTTTTGNNNNNNNNNNNNNNNNNNNNNNNNN
N

>amplified_fugu_segment
GGTATCTGTGCGCCTTTTCTCGGTCGGAGCCACATGAAGCTCCGCTGTGCCTTGAACGGT
TTCTGTCTAAACGCCACGAACGTGAAATGCCAGTTAGCTCCTATCAAAGCTGAAAACACA
CTCTCTCTCGCCCGCACACGCGTGCGCGCAATTCCTCCGTATCAGTGTTAGTTTCGGACG
CACCTGCTTTACTTTTTATTCACTTGGCTTTAATTTAAACGCTAATTTTTTATCTCAAAG
CGGACACGTTTTTGCATATTTTCCCTACATGCAGTANNNNNNNNNCGTTTTACTTCCTCA
ACAGTCGATCACGCCTCCGTTTCTGCAGAGGCAGATTTAAATATTTGTTCAGATCGATTC
TCGCTGCAAATTCAAATCTGT

>rCNE_mouse_sequence_293208
ACCTTGTTCTTATCCATCTTTAGACTGGAGTTATAATGGCATGGGACTCATGAGGTGCTG
TATTTGAAAAGTTCTTTGAAGATAAGAAAATCCTTTTAATTAAAACAAAGATAATATCAT
CTTCAATTATGCTTCTTTGATCTTTGTGGACTAGTGCATGCTGGGACTTGTAGTTTCTG

>SCE_mouse_sequence_293320
TTGAAGATAAGAAAATCCTTTTAATTAAAACAAAGATAATA

FUGU HITS IN THE CONTEST OF MAMMALIAN rCNE

CLUSTAL W (1.83) multiple sequence alignment

```
ENSG00000107485          ----AGCCTCTTCTTATCCATCTTTAGATAAAAGTTATAATAGTATGGGACTTAGGAAGT
ENSCAFG00000005019       CCCCTCCCCCTTCTTATCCATCTTTAAATTGGATTTATAATGGTATGGGCCTTATGATGT
ENSRNOG00000019336       ----ACCTTCTTCTTATTCATCTTTAGATTGGAGTTGTAATAGCATGGGACTCATGAGGT
ENSMUSG00000015619       ----ACCTTGTTCTTATCCATCTTTAGACTGGAGTTATAATGGCATGGGACTCATGAGGT
SINFRUG00000129920       ------------------------------------------------------------

ENSG00000107485          GCTGTGAGTGTTTGAAAAGCTCTTTGAAGATAAGCGAAT--TTTTTAAATTAAAATGAAG
ENSCAFG00000005019       GCTGGGAGTATTTGAAAAGCTCTTTGAAGATAAGAGAAT--TCTTTTAATTAAAACGAAG
ENSRNOG00000019336       ACTG----TGTTTGAAACGGTCTTTGAAGATAAGAAAAT--TCTTTTAATTAAAATGAAG
ENSMUSG00000015619       GCTG----TATTTGAAAAGTTCTTTGAAGATAAGAAAAT--CCTTTTAATTAAAACAAAG
SINFRUG00000129920       -----------------------TTTGAGATAAAAAATTAGCGTTTAAATTAAAGCCAAG
                                                **  ******   * *    *** *******   ***

ENSG00000107485          ATACTATCATCTTCAATTATGTTTCTTTGATCTCTGTGGACCATTGCATGCCGGAACTTG
ENSCAFG00000005019       ATATCATCATCTCCAATTATGTCTCCTTGATCTGTGTGGACCAGTGCATGCTAGGACTTG
ENSRNOG00000019336       ATAATATCATCTTCAATTATGCTTCTTTGATCTTTGTGGACTAGTGCATGCTGGGACTTG
ENSMUSG00000015619       ATAATATCATCTTCAATTATGCTTCTTTGATCTTTGTGGACTAGTGCATGCTGGGACTTG
SINFRUG00000129920       TGAATA------------------------------------------------------
                           *  *

ENSG00000107485          TAGTTTTGG
ENSCAFG00000005019       TAGTTTCTG
ENSRNOG00000019336       TAGTTTCTG
ENSMUSG00000015619       TAGTTTCTG
SINFRUG00000129920       ---------
```

Fragment Otx2_13988

Representation of conserved regions within their genic context

Within the mouse context is shown the rCNE from which the tested SCE is derived (red diamond)

Within the fugu context all CHAOS hits obtained from the mouse rCNE are shown (green diamonds, in red the one tested)

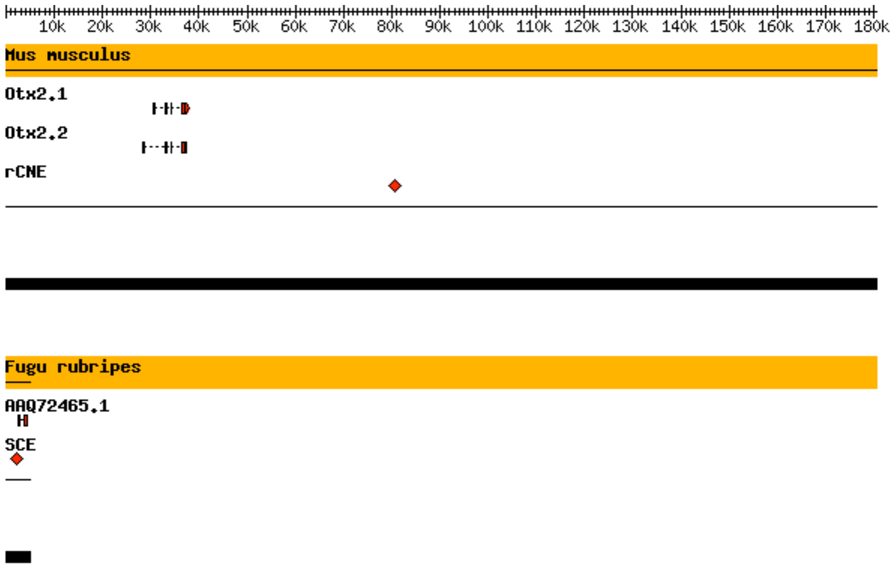

Conserved regions shown within 2000bp context

The mouse context contains:

-rCNEs green boxes, in red the one considered for this test

-SCEs yellow boxes, in red the one considered for this test

-CHAOS hits blue boxes, in red the one considered for this test

The fugu context contains:

-the fragment amplified in this test

-CHAOS hits blue boxes, in red the one considered for this test

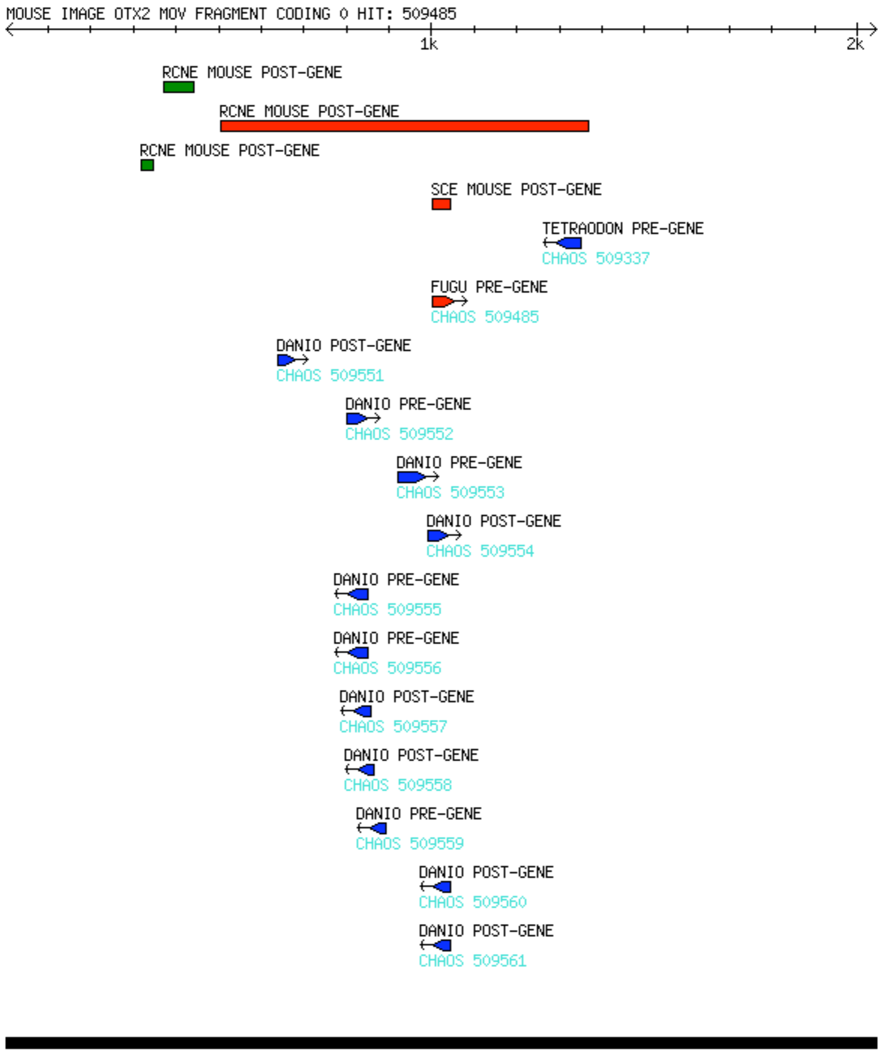

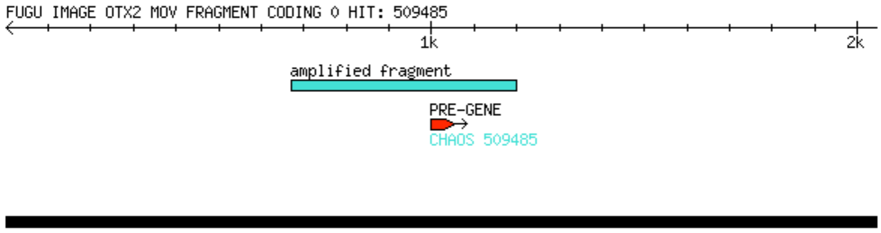

SEQUENCES

>fugu_fragment_for_primer_design_13988
GCTGGACCCTCAGTTCAGTCAAATGATTGTGAGCAGGGTAAAGAGGCTGCGTGTTTGGGC
ACCTAATTATTCTGCAGAAATCCATCCTAACGACACAGATTATGGAAATACACAGGACGC
AAATTGCGGGTCTCATCAGGATAATTGATCGAAACAGATGATGGAAGACGAAGCAGAGCT
CCGGTACATATGGTAATATAATTCACGAAAGTTGTAGGATACTTTATGTATTTCCACACG
TAGTAGTTGAGGCCACAGGACACATTGTCCTTATTGCCCGAATTTCTACTGGAAACGATG
CATTTTTGCGCTCTCACACAGATGAAAATGGAAGGGNNNNNNNNCGTCGTCAATGGCTGT
TCTAAAACAAATATATTCACGTAGGCCTGCTCTGGTCGAGGCCTTCTCCCGATGTATAAA
ACCAGCACATTATTTTTCATATATTTTAACGGCGGTTTTGTAATTCCACTGACAGATTAT
CCATTTACGCAACTATACTGCAGCCTTTGATAAACGGCCCTCACACACGTCAAATATCTC
TCCGACCTTTAGCACTCTAAAATCCCAATGAACAAAAAAGAAACTCTTAAAAAACAACTT

```
TATTTCCAGCAACAATTATTACAGAAAGACCCGAGGCGGAACCACTGCATCGATTTAACG
TTGAGTTTATACAAATGACAGACGCACCTAAGTCGTTAGACAGACANNNNNNNNNNNNNN
NNNNNNNNNNNNNNAACTATGAAGGAAGGAGGTTGTTATTGAGCGTTTTCCCCAAAACGG
GTTTGTGTAAATGTGACAGATTGGGTTGAGGGGGGTTAAAACCGTTGGTTATATTGAGGA
GAAAAAAAGAAGTGCAACTAATCAAGACGCACAGTGGGGATGATTGGTGAGGGTCAGTGA
AGTTGAAGTGACAGCAATCACGCAGGCACCACCGCATTTCTGCTTAATACCACGCCATTT
TTTAACTTAAAAATCGAATTTTCTGCGTCAGAAGTCGAATCATCTCGATCTTAAATTTAC
TGATTTTTCTGTTGGAGTGAAGGTACTGAATTGATNNNNNNNNNNNNNNNNNNNNACTAC
TACACTTTTACTACATTGATATGAGATATTTTATTGTATTATCCAGTGGATCTAAATAAT
ATATCTCTCTTTTTTTCTGGAACAGGAGCCCGGTTTGTAGCAGGGACCACAAAGAGAACT
GAAGACACCAAGTACATCGACGGAGATCCACCAGTTTTCAAAATTTTCTCCAACTTCAGC
CCTCAGACAAACATTAGCATGXXXXXXXXXXXXXXXXXXXXXXXXXXXXXXXXXXXXXXX
XXXXXXXXXXXXXXXXXXXXXXXXXXXXXXXXXXXXXXXXXXXXXXXXXXXXXXXXTGGG
TTTAAAGTCGAAATCGACAAAGATTAAATAAACAAACACTAATTGAAAATTATTGAATGT
GGTTAGAAATAAAACGGAATTGTTCGTTAAACCGTTTATGAATTATTTAACAATTAAAGC
AAATGGTAGAACAAACAACTTGTTATTACCGGCATGCTTCATTAACTTGTTACACACTCT
TGCGCTATCTCTCCAAAGCATGAATCGAAATGGTTTAAGACGTGCATCTGATTTACTGTG
TACCTTGTTGTTTGTTTTGTTTATTAAATTACGTTCCATTATTTCGCACTTGATCAAATT
GATACAATTGCCTGAAATTGCGCGTAATGATGGAATTGTTCTTTCTTATTTTATTTGCTA
AAATTACTTTGGTATTAAATTTNNNNNNNNCCCTAATAACTATTTTAAAGGTAGGCGTCT
AAAACTACTACAGTTTTAATGCGTTTCATTTTGTGCAGCAGCXXXXXXXXXXXXXXXXXX
XXXXXXXXXXXXXXXXXXXXXXXXXXXXXXXXXXXXXXXXXXXXXXXXXXXXXXXXXXXX
XXXXXXXXXXXXXXXXXXXXXXXXXXXXXXXXXXXXXXXXXXXXXXXXXXXXXXXXXXXX
XXXXXXXXXXXXXXTGAGTCCAAAACTCCGGATCTGTGTGAGCAAATGGCGATTCAAGCG
CCCATTAGCGAC
```

```
>amplified_fugu_segment
CAAATGACAGACGCACCTAAGTCGTTAGACAGACANNNNNNNNNNNNNNNNNNNNNNNNN
NNNAACTATGAAGGAAGGAGGTTGTTATTGAGCGTTTTCCCCAAAACGGGTTTGTGTAAA
TGTGACAGATTGGGTTGAGGGGGGTTAAAACCGTTGGTTATATTGAGGAGAAAAAAAGAA
GTGCAACTAATCAAGACGCACAGTGGGGATGATTGGTGAGGGTCAGTGAAGTTGAAGTGA
CAGCAATCACGCAGGCACCACCGCATTTCTGCTTAATACCACGCCATTTTTTAACTTAAA
AATCGAATTTTCTGCGTCAGAAGTCGAATCATCTCGATCTTAAATTTACTGATTTTTCTG
TTGGAGTGAAGGTACTGAATTGATNNNNNNNNNNNNNNNNNNNNACTACTACACTTTTAC
TACATTGATATGAGATATTTTATTGTATTATCCAGTGGATCTAAATAATATATCTCTCTT
TTTTTCTGGAACAGGAGCCCGGTTTGTAGCAGGGACCACAAAGAGAAC
```

```
>rCNE_mouse_sequence_478034
GCCCACTGACCTACTTGCAATAGGGGAGGTAAAATATTTTCATTACCCTCTCCCCTAGAA
TGTAGAGATCTTCAAAGGGAACGCAGGAGGGGGGTCAGCCTTTTAACCCTAATTTCCCTA
CCACTCCCTAAGCTTGGCCGTGCATCACAAATTTAGTCTTTAGTCTCCAAACATAGCGGC
ACCAGGATCGTTGTCGGGTTGGCTTTTTAAAACATCATTTCATATGGCAAACCCAGCCAT
TATTCCATGCTGCTTAAACTATATTTGTAGTCTTTGATCATGCATAAGTAGACTCAAGTT
AGCCGCTGGTAGAACGATGATATTGTTCTCTGCTTTGCCTTTTAAGGGCTCTCTAAGAGC
TGGCTTCCAGAAATTTCCATTACTACGTTATAGTGTGCCTTTGTCTTTCAGTTGGTTCAG
GACAACCTATTTGCATGACAAGTGAAATAATTCTGTTAGAAAAATCTGTTATGTATATGG
TTTGCCTAATCTATATTTATTGCATTTTAAAAATTACTGATTATTTCAAAAATTGAAAGA
CGCTGAATTGGCAAAGAATGTCTCGGGGAAGGAAAGGGCATAGTCGCTGCTGATTATTGT
TATTTCCACCCAGGCTGGCTTCTCCTCTCCTTCCTCCCCTTCTATTTCGTTTCAGAAGTT
GCTTTATATTGGCTTCCTCTTCCCACACCTCCACTCCCAGCAGGAAGATACTGTACTGCC
ACAAGTGGGGGCCGAAAATGGGGGAGAAATCGGTTCTGAATAGCGGCACCAAATAGATTG
AAGTGCTTTTCTAAATATATTTCAAGATTGAGTAATTAAAGATTGCAAAGCCCTTGGAAA
TAAATAAAGCACCTTGTGAGTGCT
```

```
>SCE_mouse_sequence_478106
TTATTGCATTTTAAAAATTACTGATTATTTCAAAAATTGAAAGA
```

FUGU HITS IN THE CONTEST OF MAMMALIAN rCNE

CLUSTAL W (1.83) multiple sequence alignment

```
ENSRNOG00000013406      GCCCACTGACCTACTTGCAATAGGGGTGGTAAAATATTTTCATTACCCTCTCCCCTAGAA
ENSMUSG00000021848      GCCCACTGACCTACTTGCAATAGGGGAGGTAAAATATTTTCATTACCCTCTCCCCTAGAA
ENSG00000165588         GCCCACTGACCTAATTGCAATAGAGGTGGTAAAATATTTTCATTACCGTCTCCCCCAGAA
ENSCAFG00000015216      GCCCACTGACCTAATTGCAATAGGGGAGGTAAAATATTTTCATTACCCTCTCCCCCAGAA
```

```
SINFRUG00000151879        ------------------------------------------------------------


ENSRNOG00000013406        TACAGAGATCTTCAAAGGGAACGCAGGAGGGGGGTCAGCCTTTTAACCCTAATTTCCCTA
ENSMUSG00000021848        TGTAGAGATCTTCAAAGGGAACGCAGGAGGGGGGTCAGCCTTTTAACCCTAATTTCCCTA
ENSG00000165588           TATAGAAATCTTCAAAGGGAACCCAGGAGGGAGGTTAGCCTCTTAACCCTAATTTCCCTA
ENSCAFG00000015216        TATAGAAATCTTCAAAGGGAACCCGGGAGGCAGGTTAGCCTCTTAACCCTAATTTCCCTA
SINFRUG00000151879        ------------------------------------------------------------


ENSRNOG00000013406        CCACTCCCTACGCTTGGCCGTGCATCACAAATTTAGTCTTTAGTCTCCAAACGTAGCGGC
ENSMUSG00000021848        CCACTCCCTAAGCTTGGCCGTGCATCACAAATTTAGTCTTTAGTCTCCAAACATAGCGGC
ENSG00000165588           CCACTACCTCAGCTTGGCTGTGCATCACAAAATTAGTCTTTAGTCTCCGAACATAGTGGC
ENSCAFG00000015216        CCACTACCTCGGCTTGGCTGTGCATCACAAAATTAGTCTTTAGTCGCCGAACATAGTGGC
SINFRUG00000151879        ------------------------------------------------------------


ENSRNOG00000013406        ACCAGGATCGTTGTCGGGTTTGCCTTTTAAAACATCATTTCATATTGCAAACCCGGCCAT
ENSMUSG00000021848        ACCAGGATCGTTGTCGGGTTGGCTTTTTAAAACATCATTTCATATGGCAAACCCAGCCAT
ENSG00000165588           ACCAGGATTGTTGTCGGTTTTGCTTTT--AAAAATCATTTCATATTGTAAACCCGGCGAT
ENSCAFG00000015216        ACCGGGATTGTTGTCGGTCTTGCTTTT--AAAAATCATTTCATATTGTAAACCCAGCGAT
SINFRUG00000151879        ------------------------------------------------------------


ENSRNOG00000013406        TATTCCATGCTGCTTAAACTATATTTGTAGTCTTTGATCATGCATAAGTAGACTCAAGTT
ENSMUSG00000021848        TATTCCATGCTGCTTAAACTATATTTGTAGTCTTTGATCATGCATAAGTAGACTCAAGTT
ENSG00000165588           TATTTCATGCTGTTTAAACTATATTTGTAGTCTTTGATCATGCATAAGTAGACTCAGGTT
ENSCAFG00000015216        TATTTCATGCTGTTTAAACTATATTTGCAGTCTTTGATCATGCATAAGTAGACTCAAGTT
SINFRUG00000151879        ------------------------------------------------------------


ENSRNOG00000013406        AGCCGCTGGTAGAACGATGATATTGTTCTC--TGCTTCGCCTTTTAAGGGCTCTCTAAGA
ENSMUSG00000021848        AGCCGCTGGTAGAACGATGATATTGTTCTC--TGCTTTGCCTTTTAAGGGCTCTCTAAGA
ENSG00000165588           AACTGCTGATAGAATGATGATATTGTTCTCCATGNNNNNNNNNNNNNNNNNNNNNNNNNN
ENSCAFG00000015216        AACTGCTGATAGAACGATGATATTGTTCTCTGCGTTTCTTTTTCTTTTTCTTCCCTGTCT
SINFRUG00000151879        ------------------------------------------------------------


ENSRNOG00000013406        GCTGG------------------------CTTCCAGAAATTTCCATTACTACATTATAGC
ENSMUSG00000021848        GCTGG------------------------CTTCCAGAAATTTCCATTACTACGTTATAGT
ENSG00000165588           NNNNNAAGGACCCTGTACCCAAGAGCTGGCCTCAAGAAATTTCCATTACTACATTACAAC
ENSCAFG00000015216        TTAAG---GACCCTGTGCCTAGGAGCTGGCATCCAGAAACTTCCATTACTCCATTACAAC
SINFRUG00000151879        ------------------------------------------------------------


ENSRNOG00000013406        GTGCCTTTGTCTTCCAGTTGGTTCAGGACAACCTATTTGCATGACAAGTGAAATAATTCC
ENSMUSG00000021848        GTGCCTTTGTCTTTCAGTTGGTTCAGGACAACCTATTTGCATGACAAGTGAAATAATTCT
ENSG00000165588           ATGCCTTTGTTTTTTAATTGGTTCAGGACTACCTATTTGCATGACAAGCAACATAATTCA
ENSCAFG00000015216        GTGCCTTTGTTTTTTAATTGGTTCAGGATTACCTATTTGCATGACAAGCAACATAATTCA
SINFRUG00000151879        ------------------------------------------------------------


ENSRNOG00000013406        GTTGGNNNNNNNNNTCTGTTATGTGTATGGTTTGCCTAATCTGTATTTATTGCATTTTTA
ENSMUSG00000021848        GTT----AGAAAAATCTGTTATGTATATGGTTTGCCTAATCTATATTTATTGCATTTTAA
ENSG00000165588           GTT----AGCAAAATCTGTTGTGTGTATGGTATGCAGAATCTATATTTATTGCATTTTAT
ENSCAFG00000015216        GTC----AGAAAAATCTGTTGTGCGTATGCTATGCATAATATATATTTATTGCATTTTAT
SINFRUG00000151879        ----------------------------------------------TCATCTCGATCTTA
                                                                        * **  *  * *

ENSRNOG00000013406        AAATTACTGATTATTTCAAAAATTGAAAGACGCTGAATTGGCAAAGAATGCCATGGGGAA
ENSMUSG00000021848        AAATTACTGATTATTTCAAAAATTGAAAGACGCTGAATTGGCAAAGAATGTCTCGGGGAA
ENSG00000165588           AAACTACTGATTATTTAAAAATTTGAAAGACGCTGGATTGGCAATGAATGCCAAAGAGAA
ENSCAFG00000015216        AAATTACTGATTATTTACAAATTTGAAAGGCGCTGGATTGGCATAGAATGCCGAAGAGAA
SINFRUG00000151879        AATTTACTGATTTTTCTGTTGGAGTGAAGGTACTGAATTG--------------------
                          **  ******** **           ***   *** ****

ENSRNOG00000013406        GGAAAGGGCA-TAGTCACTGCTGATTATTGTTATTTCCGCTCAGGCTGGCTTCTCCTCTC
ENSMUSG00000021848        GGAAAGGGCA-TAGTCGCTGCTGATTATTGTTATTTCCACCCAGGCTGGCTTCTCCTCTC
ENSG00000165588           GGAGAGAGTAGTAGTCATTGCTGATTATTGTTATTTGAGCACAGGCTGGTTTCTCCTCTC
ENSCAFG00000015216        GGGGAGAGTAGTAGTCGCGGCTGATTATTGTTATTGTACCGCAGGCTGGTTTCTCCTCAC
SINFRUG00000151879        ------------------------------------------------------------


ENSRNOG00000013406        CTTCCTCCCCTTCTATTTCGTTTCAGGAGTCCCTTTATATCGGCTTCCCCCTCCCACGCC
ENSMUSG00000021848        CTTCCTCCCCTTCTATTTCGTTTCAGAAGTTGCTTTATATTGGCTTCCTCTTCCCACACC
ENSG00000165588           CTTCCTCCCCCTCCATTTTTCTTCAGGAGTTCCTTTCTATCAGCCACCCCTTTCCACACC
ENSCAFG00000015216        CTTCCTCCCCCTGCATTTTTCTTCAGGAG---CTTTCTGGGGGCCACTCCTTCCTGCACC
SINFRUG00000151879        ------------------------------------------------------------


ENSRNOG00000013406        TCCATTCCCAGCAGGAAGATACCGTACTGCCACGAGTGGGGGCCGAAAAATGGGGAGAAA
ENSMUSG00000021848        TCCACTCCCAGCAGGAAGATACTGTACTGCCACAAGTGGGGGCCGAAAATGGGGGAGAAA
ENSG00000165588           TCGATTCCCAGCAGGAAGATACTGTACTGCCACGAGTGGGGGCTGAAAA-TGGGGCAAAA
ENSCAFG00000015216        TCGCTCCCCAGCGGGAAGACACAGAACCTCCAGGAGTGGGGGCTGCAAA-TGGGGCAAAA
SINFRUG00000151879        ------------------------------------------------------------


ENSRNOG00000013406        TCGGTTCTGAGTAGCGGCACCAAATAGATTGAAGTGCCTGTCTAAATGTATTTCAAGATT
```

```
ENSMUSG00000021848      TCGGTTCTGAATAGCGGCACCAAATAGATTGAAGTGCTTTTCTAAATATATTTCAAGATT
ENSG00000165588         TAGGTTTTGAATAGCAGCGTCAAATAAATTCAAGTGCCTTTCTAAATATATTTCAAGATT
ENSCAFG00000015216      TAGGTTTTGAATAGCAGCGTCGAATAAATTCAAGTGCCTTTCTAAATATATTGCAAGATT
SINFRUG00000151879      ------------------------------------------------------------

ENSRNOG00000013406      GAGTAATTAAAGATTGCAAGGCCCTTGGGAATAAATACAGCACCTTGTGAGTGCT
ENSMUSG00000021848      GAGTAATTAAAGATTGCAAAGCCCTTGGAAATAAATAAAGCACCTTGTGAGTGCT
ENSG00000165588         AAGTAATTAAGGAGTGTAAAGCACTTGGAAAAAAATAAAGTGCCATGTAAGTGCT
ENSCAFG00000015216      AAGTAATTAAGGATTGTAAAGCACTTGGAAA-AAATAAAGTACCACGTAAGTGCT
SINFRUG00000151879      -------------------------------------------------------
```

Fragment Jag1_16407

Representation of conserved regions within their genic context

Within the mouse context is shown the rCNE from which the tested SCE is derived (red diamond)

Within the fugu context all CHAOS hits obtained from the mouse rCNE are shown (green diamonds, in red the one tested)

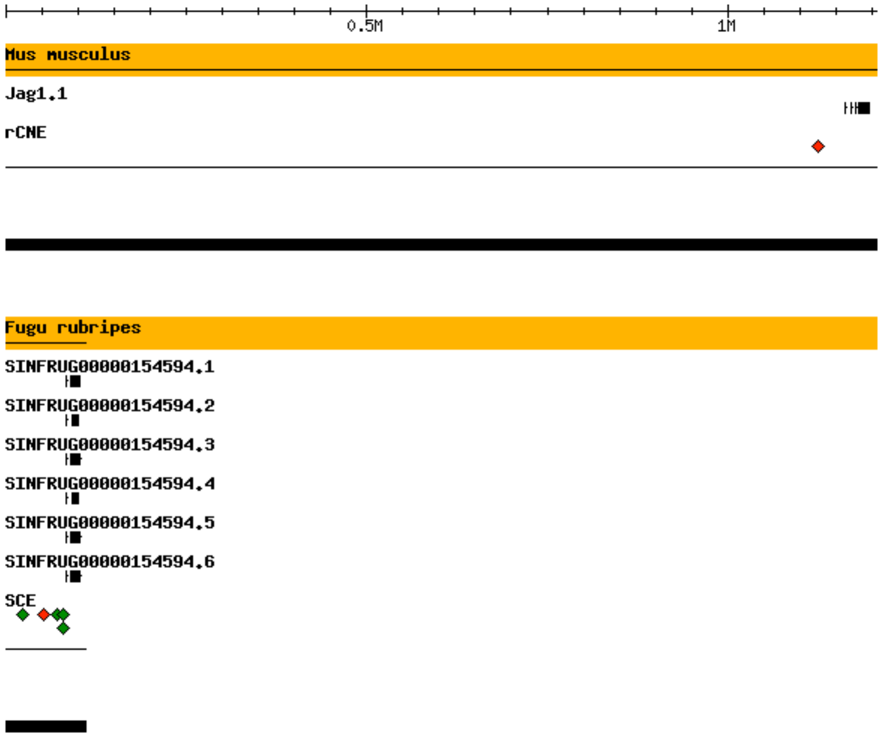

Conserved regions shown within 2000bp context

The mouse context contains:

-rCNEs green boxes, in red the one considered for this test

-SCEs yellow boxes, in red the one considered for this test

-CHAOS hits blue boxes, in red the one considered for this test

The fugu context contains:

-the fragment amplified in this test

-CHAOS hits blue boxes, in red the one considered for this test

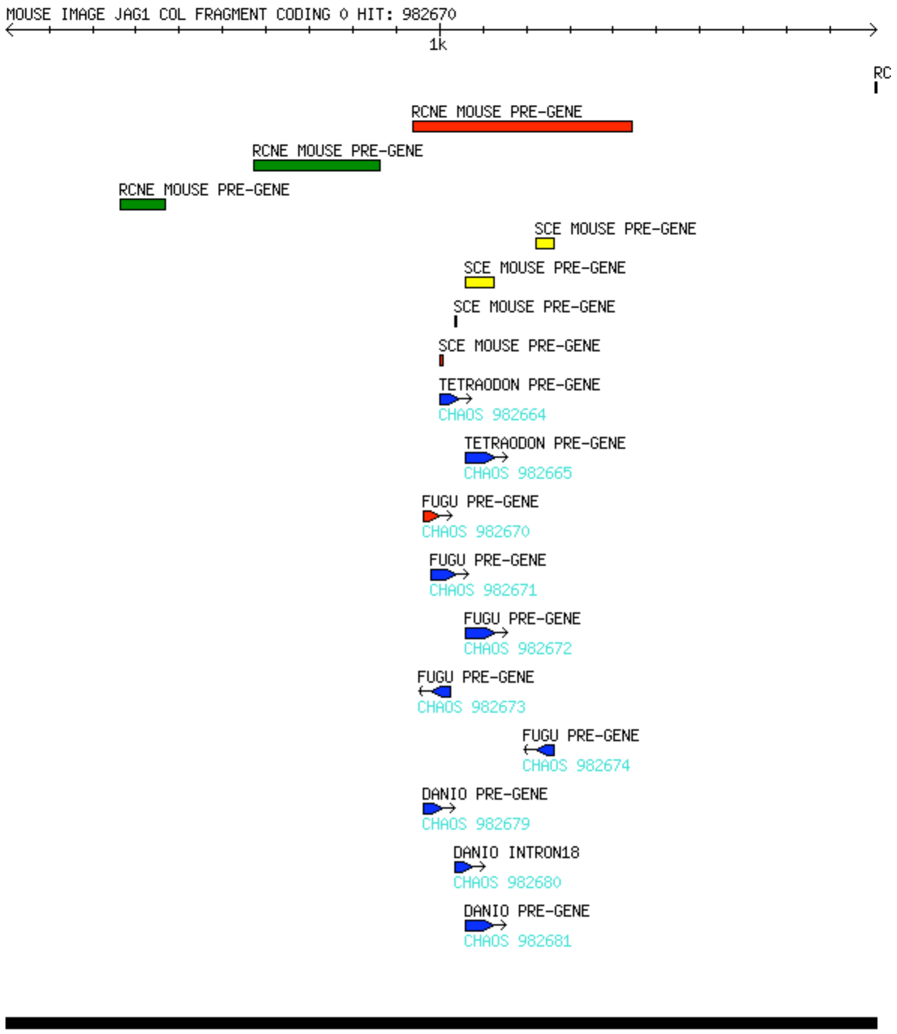

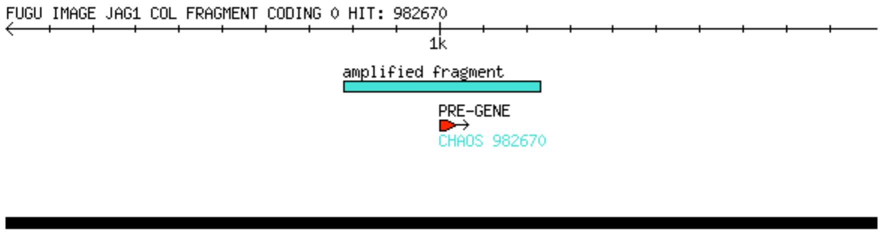

SEQUENCES

>fugu_fragment_for_primer_design_16407

CACATTGGTTCCATGCTGAAGCCATATTGAGCCCAAATTGAAGCCAGATTGAGACGCTTG
TTCAGTAGTCTCTCTTTAAAGATGCCAATTGCAAAACATGTATTTTTGGGAGGCCAAATA
TCCAGATAGATCATTCACGTCAGGTGACATCAGGTGACATGATGCATAAATGATGAAAAG
GACAGACAATCACCAAACCCAGAATCCATGAAATCAGAAACTCATTTCAGCTGCTCTTGC
TTTGCCATCATTTTACAAACTTCCAGGCCTTTTTGACTCACATGGAACCAATTGTGTTTA
AGCCGAATGTCGCGACATGATGCAAGAGACCACCAGTCCTGCCGTGTACACTGATCCATC
CAGTGTAGCCCGAAATTGACATGGCACCAACATTCAGGACCTCCGTCTGGTGTTGTTAGC
TGTCTCTGCCAGTNNNNNNNNNACTCCCTTCAAACGTGCCAATACAGACAAATCTCTCGC
ACACTCACCTTACACTATTTCCTTGCCAGCAACATGCTCAAATACCAACCACACTGAGTT
AAATGGAGGTTAAATAGAGGCTATTAATGTCACACCTGAGTGAAACTTCCATAATGTAAG
CAACCCTTACCCACTCCAGAAAGACTATGTGGTTTAGTAATTTACTGGATTTAATAACAT
TTATTGAAACCCCTGGACCTGCACTGACCTCCCCTTAATTGCAGCATGGGCCCCAAGATG
TTGGTGACTTAAAAGGTGCTGGCCTGTAAAACAAGAACAAGAAAAACTCCCTGCAATGAA
GACTCTTTTGGTGGCCTCTTTCTTCACAGAGGCTTGGCAAAGGTGAAGTGTGACTAAAGA
GGATATAGCTTGGGCATAATTTGGGAGCTGCTATGGGTACGGAGCTGCTACATTTGCTAA
GCACATCTCACTGTTTTCCATTTATTCCCGCGTGGTTAAATGCCATGGCAACCCAAGCAT
GGACTTTAATGAAGCTGAGATGGACCATGTTGCACTACTCCCTTCGGTAAAGTTTTACAG
TGTCCAACCACATTCTCCCTTAAAAGCCAGGGGAAAGTGACGCCATACGCTAACAAAATA
CTCATACTGCTGTTGGAAGAAAAGATGGATTTTGGTGTTATTTTTCAGATAATATTTGAA
GATTACAATGTTGTGCATCAACAGAAAAAATTATTATTGCTCCACATTTGATAGCAATTA
GCTCAACACACAGAGACCATCACCCTCTTGAGTTGTGCTAATCAGCCCACAAGGCATCTG
TATCTGCCAGGCTTCAGCGCCGCCGCAGCTAATGGATCCGTACTTTATCACATTGATTCT
CCCTGTAAAGCCAGCCCCCCTGTCAAAACCACCCACCTCCCCCCTTCACTCATATGGCCT
TGATGAGATCCAGACTCTGTGTGGAAGCATATTAAAGTGGCCCAACAGTGTCTGCAGGGG
AGCTAAATGGAAGGTAGTGAAATCTTTTGAACCTCTGTTACGTTACAGCAGTTCTAAACA
CATCCAGCTGAGTCTAAAGTTCAGGGAAGACATAAAAGCACCATCGCACGTTACCTAAAG
GATGATAATAGGCGAGCGCCACTAAAACTACTAAGACAGTCTACAATCAAAGGGCCGAGA
GGAAGGACTCCCTGCCCTTCTTACTGGCTGCTAATGGCCTGTGTGTGTAAGAGACAGCCT
ATCGTTATATCTTCTATAACACACACTCCGTGTGTTGGGTGTTGGTGATTAAACTCCATT
AACTCTGGCTTAATTAGAATACAAAAGACTTAGAAGAGCAGTAATTACCTTGTGCCGGCC
AGGTAGGTCACCTTTTAAGCAAATTACAGTGTACTGATTTGATGGGGTCGTGTTGTGAGT
TCATGGCTGCCAGATGTTATTTTTTTCCTACAAGATGGCTGACATTGACAAAAAGCTTGT
TTTTTAGCCTTGTTACAGAGGAGATTATCTGGACAGTTGTACTGAACGCTCGGTAAGACA
GGCNNNNNNNNNNNNNNNNNNNNNNNNNNNNNNNNNNNNNNNNNNNNNNNNNNNNN

>amplified_fugu_segment
AAGACTCTTTTGGTGGCCTCTTTCTTCACAGAGGCTTGGCAAAGGTGAAGTGTGACTAAA
GAGGATATAGCTTGGGCATAATTTGGGAGCTGCTATGGGTACGGAGCTGCTACATTTGCT
AAGCACATCTCACTGTTTTCCATTTATTCCCGCGTGGTTAAATGCCATGGCAACCCAAGC
ATGGACTTTAATGAAGCTGAGATGGACCATGTTGCACTACTCCCTTCGGTAAAGTTTTAC
AGTGTCCAACCACATTCTCCCTTAAAAGCCAGGGGAAAGTGACGCCATACGCTAACAAAA
TACTCATACTGCTGTTGGAAGAAAAGATGGATTTTGGTGTTATTTTTCAGATAATATTTG
AAGATTACAATGTTGTGCATCAACAGAAAAAATTATTATTGCTCCACATTTGATAGCAAT
TAGCTCAACACACAGAGACCATCACCCTCTTGAG

>rCNE_mouse_sequence_907951
AAAAGATCTGGCCTTCAGAAAGCTTCTCATTAAAACACTAATCTGTTCCACCACATTCTT
CCAAGACACAGCGATCAGCATTGGAGCTGGCTTGAATGGCCAGGCCTAGTAAGACTGATA
CAAACAGCTTAAGTAATTGGATTGCTATATGGGATTGAATGTCTGACAACAAAAGATAGC
CATCTCTATTCGACCACCAAAGCTTTGTTCCAGCAACGAATTCATTCAGCAATGCCCTTA
GAGGCTCCTGGACCCAGGGAGAAATGACAGACCTGAGACACAAAAACTGAGCCAGCTGAT
GAGCCCAGAGACAGAGGCTATGCAATTCCAAAAAGTTAATTACAGCGAACAAAGGCAGCA
GACGATGACTTGTTGAATCATTACCATAATCACATTACTGCACTAGTCTCCAGTTCCCAG
CCAAGGGTGGCGGTGCGGCTGCTTCTCATTCTGACCATGTACTCCCTGCTCGACTTCCAC
GTGCAAGCTACTGTACACGTCTCCT

>SCE_mouse_sequence_908243
CAAGAC

FUGU HITS IN THE CONTEST OF MAMMALIAN rCNE

CLUSTAL W (1.83) multiple sequence alignment

```
ENSMUSG00000027276      AAAAGATCTGGCCTTCAGAAAGCTTCTCATTAAAACACTAATCTGTTCCACCACATTCTT
ENSRNOG00000007443      AAAAGATCTGGCCTTCAGAAAGCTTCTCATTAAAGCACTAATCTGTTCCACCACATTCTT
```

```
ENSCAFG00000005627         AAAAGATCTGGCCTTCAGAAAGTCTCTCATTAAAGCGCTAATCTGTTCCACCACATTCTT
ENSG00000101384            AAAAGATCTGGCCTTCAGAAAGCCTCTCATTAAAGCGCTAATCTGTTCCACCACATTCTT
SINFRUG00000154594         ----------------------CCCTTCGGTAAAGTTTTACAGTGTCCAACCACATTCTC
                                                     **  ****    **   *** * **********

ENSMUSG00000027276         CCAAGACACAGCGATCAGCATTGGAGCTGGCTTGAATGGCCAGGCCTAGTAAGACTGATA
ENSRNOG00000007443         CCAAGACACAACAATCAGTGTTGGAGCTGGCTTGAATGGCCGGGCCTAATAAGACTGATA
ENSCAFG00000005627         CTGAGACACAGCGATCAGCATTCTAGCCGGCCTCAATGGCTCGTCCTAATAAGACTGATA
ENSG00000101384            CCAAGACACAGCGACCAGCATTCTAGCCAGCCTCAATGGCCAGCCCTAATAAGACTGATA
SINFRUG00000154594         CC----------------------------------------------------------
                           *

ENSMUSG00000027276         CAAACAGCTTAAGTAATTGGATTGCTATATGGGATTGAATGTCTGACAACAAAAGATAGC
ENSRNOG00000007443         CAAACAGCTTAAGTAATTGGATTGCTATATGGGATTGAATGTCTGACAACAAAAGATAGT
ENSCAFG00000005627         GAAATAGCTTAAGTAATTGGATTGCTATATGGGATTGAATGTCTGACAACAAAAGATAGC
ENSG00000101384            GAAACAGCTTAAGTAATTGGATTGGTATATGGGATTGAATGTCTGACAACAAAAGATAGC
SINFRUG00000154594         ------------------------------------------------------------


ENSMUSG00000027276         CATCTCTATTCGACCACCAAAGCTTTGTTCCAGCAACGAATTCATTCAGCAATGCCCTTA
ENSRNOG00000007443         CATCTCAATTCGACCACCAAAGCTTTGTTCTAGCAATGAATTCATTCAGCAATGCACTTA
ENSCAFG00000005627         CATCTCTATTCGACCACCATATCTTTGTTCCAGCAATGAATTCATTTAGCAATGCACTTA
ENSG00000101384            CATCTCTATTCGACCACCAAATCTTTGTTCCAGCAATGAATTCATTTAGCAATGTGCTTA
SINFRUG00000154594         ------------------------------------------------------------


ENSMUSG00000027276         GAGGCTCCTGGACCCAGGGAGAAATGACAGACCTGAGACACAAAAACTGAGCCAGCTGAT
ENSRNOG00000007443         GAGGCTCCTGGGCCCAGGGAGAAATGACAGACCTGGGACACAAAAACTGGGCCAGCTGAT
ENSCAFG00000005627         CAGGCTCCTGGGCCCAGGGAGAAATGACAGACTAGGGACACAAAAGCTAGGCCAGCTGAT
ENSG00000101384            GAGGCTCCTGAGCCTGGGCAGAAATAACAGACTGGGGACACAAAAGCTAGGCCAGCTGAT
SINFRUG00000154594         ------------------------------------------------------------


ENSMUSG00000027276         GAGCCCAGAGACAGAGGCTATGCAATTCCAAAAAGTTAATTACAGCGAACAAAGGCAGCA
ENSRNOG00000007443         GAGCCCAGAGACAGAGGCTATGCAATTCCAAAAAGTTAATTACAGCGGACAAAGGCAGCA
ENSCAFG00000005627         GAGCCCAGAGACAGAGGCTGCACAATTCCAAAAAGTTAATTATGGTGAACACCAGCAGCA
ENSG00000101384            GAGCCCAGAGACAGAGGCTGCGCAGTTCCAAAAAGTTAATTACAGTGGACAAAGGCAGCA
SINFRUG00000154594         ------------------------------------------------------------


ENSMUSG00000027276         GACGATGACTTGTTGAATCATTACCATAATCACATTACTGCACTAGTCTCCAGTTCCCAG
ENSRNOG00000007443         GACGATGACTTGTTGAGTCATTACCGTAATCACATTACTGCACTAGTCTCCGGTTCCCAG
ENSCAFG00000005627         GACAATGACTTGTTGAATCATTACCATAATCACATTACTGCACTGGTCTCCAGTTCCCAG
ENSG00000101384            GACGATGACTTGTTGAATCATTACTATAATCACATTACTGCACTGCTCTGAAGTTCCCAG
SINFRUG00000154594         ------------------------------------------------------------


ENSMUSG00000027276         CCAAGGGTGGCGGTGCGGCTGCTTCTCATTCTGACCATGTACTCCCTGCTCGACTTCCAC
ENSRNOG00000007443         CCAAGGGTGGCGGTGTGGCTGCTTCTCATTCTGACCATGTACTCCCTACTCATCTTCCAC
ENSCAFG00000005627         CCAAGGGCGGTGTTG--GCTACTTCTCATTCTGACTCTGTCCTTCATCCTCACTTGCCAC
ENSG00000101384            CCAAGG-TGGCATTG---CTGCTTCTCATTCTGCCCATGTACTTCATACTCATTTGCCAC
SINFRUG00000154594         ------------------------------------------------------------


ENSMUSG00000027276         GTGCAAGCTACTGTACACGTCTCCT-
ENSRNOG00000007443         CTACAAACACATG---GTGTCT----
ENSCAFG00000005627         CTGCAATCTTCTGTACATGACTTCTT
ENSG00000101384            CTGCAATTTACTGTGTGTGACTTCT-
SINFRUG00000154594         --------------------------
```

Fragment Jag1_16408

Representation of conserved regions within their genic context

Within the mouse context is shown the rCNE from which the tested SCE is derived (red diamond)

Within the fugu context all CHAOS hits obtained from the mouse rCNE are shown (green diamonds, in red the one tested)

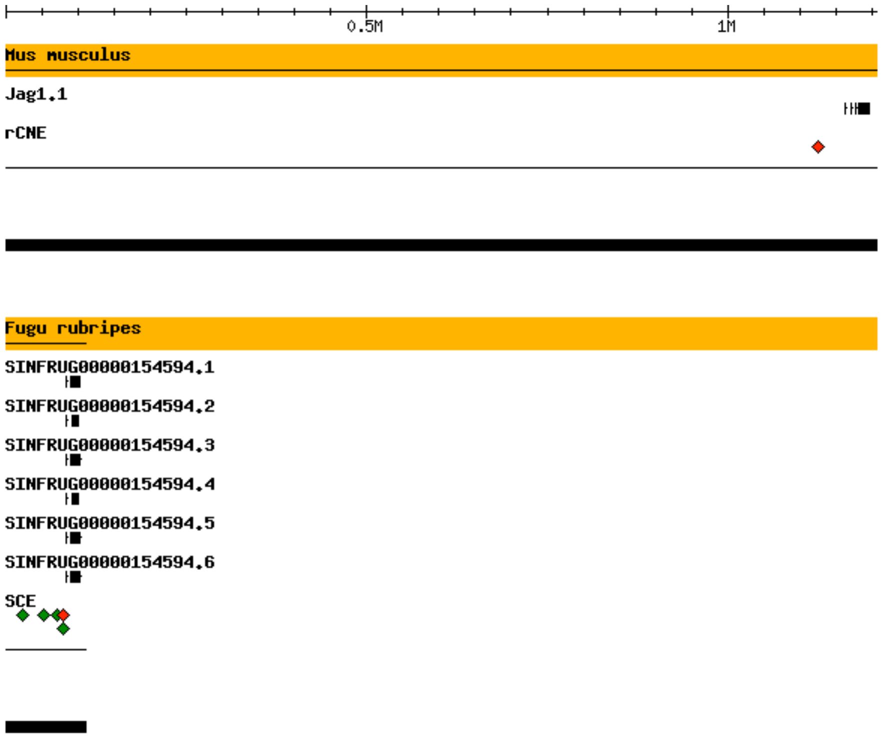

Conserved regions shown within 2000bp context

The mouse context contains:

-rCNEs green boxes, in red the one considered for this test

-SCEs yellow boxes, in red the one considered for this test

-CHAOS hits blue boxes, in red the one considered for this test

The fugu context contains:

-the fragment amplified in this test

-CHAOS hits blue boxes, in red the one considered for this test

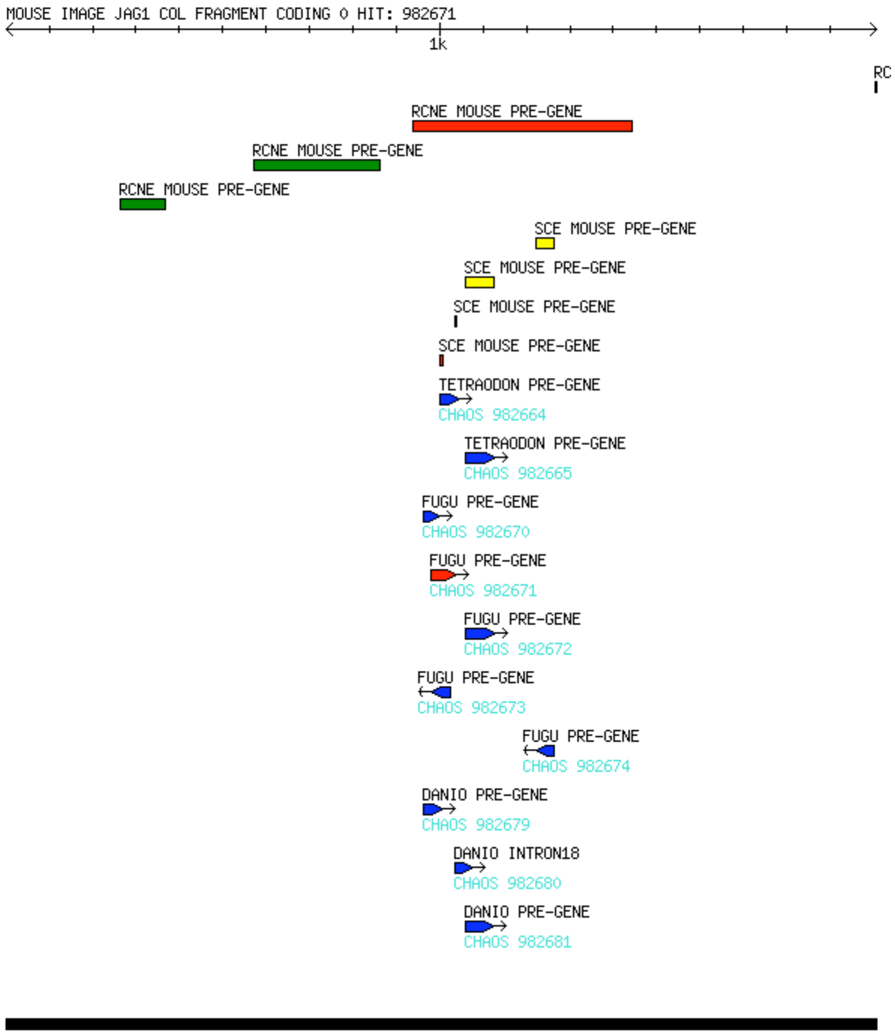

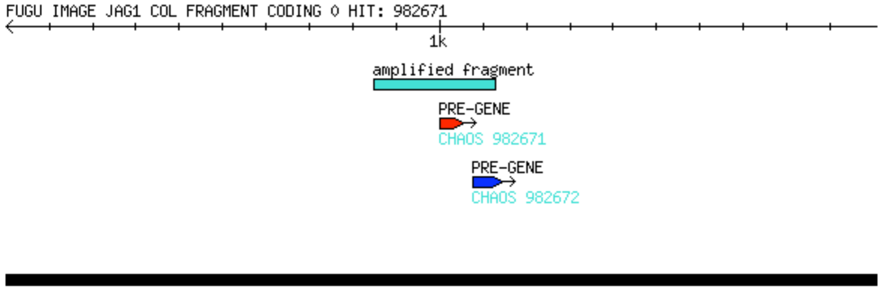

SEQUENCES

>fugu_fragment_for_primer_design_16408
GGACTCTAATATCCCTGAACCAGAGTGAGGTACCCACAGCAAGACATAAAAACACCATCA
CCTACCAGAATAGGCTCTCAAACAGATGGTGGGGATAACAAAGCCGCCACCTTTTACAGT
GGAAAATAATATTTATCTAAGTGCCGACGGCTGATACCACCTGTAACACTTTTTGTAAAG
GCAGTTCCTGTAAACATGGCGTGTAAACTCTGAAGCTTTTAAATGACTCAAACATTGAAC
CGCAGACTCGCACCTCCTCACCTTACACTGCGTTCTCTTCGGATATCCTCAGACATTCTT
GACCTCCGCTCAGAAACAAGGACTTTATGGAAAAACTCAGACTTTCTCACAGGAAGATGT
TGCCTTGTGCCAACTTGACTTCTCCTCTCAGTATTTCTGTATTTAGCCTGATCTATTCTT
TCTACCGTTCAACCAGGGATTTACAGTTCTGAACCTGACCTGCAGCAGATACTGAAGGTT

GAGCCATCGTGGTCCGCTGATGCCAGCTGAGGCCAAAGTTTGCACAGTAGCAAAGAAAAG
ACAACACACAGTCCCACCTCAAGATACTACCTCACAACTGCACACAAAGCCTTGTTAAGG
TCAGAGGTCATCATTATTCAAGCACCTCTGTAACTGCTGTTTTCAAAGATAAATCCTCTC
CTTTTAGGAAAAAAGGGCTATCAGCATTCCATGGAGAATCTGCAGTCACACAGGTGGAAG
CAGGAAGCAGCAATATGCTCGCACTCTCCTGGATTGAGGACCATCCTTGCCTACATAAAC
AAGCATCATTCTGCACAAACATCCTCGTGCAGTTTTGACTGGACGGGCTTTAAAACGTGT
CTTACAAATATCCAATTTGGCTGATCCTGTCCAGACAGTAATGAAATTAAAATAAACAGG
ATCAAGTTGGAAATAGTAGGGTGGTTGTTCTAAACCTTTTTAGCGGAGAGTGCAACACAC
ATTTTTGTAAGGGTGGGGGAGGAAAAACAGTCACTGGGCACCTAATCCACCGCATTCTTG
TCAGGGGTCCGAGTCTGAATTCAGGCTGGCGTCAATGCCGTGCACCAGGAGGGGAGCAAA
AGGCTGAGGTAATTGGATTGGAATACGGCATTGAGCGTCAGACAACAAAAGATATCCATC
TCTCTCATGGCCTTTCAGCTCTTTTGTGGCTGGCTGGAATTTATTTGGTTATTCGCTCCC
GCTCTTCCGAGGACGAGAGAGTGGGGGCAGGCCACCGGGACTGGCTCAGGCCGGTGCTCT
GGTAGACAGAGTCACGGCGCCTGTGCTGCTGCGGTGTGGGGATGTGTGAACCACAGATCC
CCCCAAAGCCCAGGGGTCATTGTGTGATCATTACCATAATCACATTAAGGCTGCAGGAGA
TTAACACCAGAGCCTCAGAGGAGCGACCCCTTCTGCGACTGTAGGCCGCGCTTACAGGGA
ACTGGGAGAGTGCTGCTGGTCTACCTCAGGTGGGAGATGGACACCATCGACGCTGGGATA
GCCCTCTTATCCCCATCAAATGCACATTTTTGTACATCCTAGCAGACCTAACCATGCATG
TGAAGCTAGTTTCAAGACTCCATCACAGAAGAATTCTCTCTGGGGTTAAAAACCTCTCAA
ATTAACCCTAATTACCCTGAAAGAGCATTTCAATGAGGCATGTAACCTGAGATGTCTATC
TGTTGAGGCAGGGAAGGGGGGTGCTGGGTCGTACCTTCAGCTTGAAGTTCACCCGCAGGA
ACTGCAGCATGGTGATTTTGACAGCTGTGATTAAAATGCTGGGGAGAGCTTGTGAAGGCG
TTACACCGATGCACCCGTGCTTCACCCTCGCAGCGAGGATGCAGAAATACCGATGAGGAA
ACGGAAAGATGAATTCAAGGAATCTGATGCTTCTTAGCCGTGCTATAATGCCTGATATCA
TCTGCTCAACAACAACAAACTCCTAGATGAAGTATTGCTAACTTACCTCCTAGCCTTTAG
AACAAGAGAGAAAATGGAACCCAAGCGCTCAGTAGAGGTTTGAGGATGTGTCGAGGGACG
TTAAGATGATGTCCAA

>amplified_fugu_segment
ATCCAATTTGGCTGATCCTGTCCAGACAGTAATGAAATTAAAATAAACAGGATCAAGTTG
GAAATAGTAGGGTGGTTGTTCTAAACCTTTTTAGCGGAGAGTGCAACACACATTTTTGTA
AGGGTGGGGGAGGAAAAACAGTCACTGGGCACCTAATCCACCGCATTCTTGTCAGGGGTC
CGAGTCTGAATTCAGGCTGGCGTCAATGCCGTGCACCAGGAGGGGAGCAAAAGGCTGAGG
TAATTGGATTGGAATACGGCATTGAGCGTCAGACAACAA

>rCNE_mouse_sequence_907951
AAAAGATCTGGCCTTCAGAAAGCTTCTCATTAAAACACTAATCTGTTCCACCACATTCTT
CCAAGACACAGCGATCAGCATTGGAGCTGGCTTGAATGGCCAGGCCTAGTAAGACTGATA
CAAACAGCTTAAGTAATTGGATTGCTATATGGGATTGAATGTCTGACAACAAAAGATAGC
CATCTCTATTCGACCACCAAAGCTTTGTTCCAGCAACGAATTCATTCAGCAATGCCCTTA
GAGGCTCCTGGACCCAGGGAGAAATGACAGACCTGAGACACAAAAACTGAGCCAGCTGAT
GAGCCCAGAGACAGAGGCTATGCAATTCCAAAAAGTTAATTACAGCGAACAAAGGCAGCA
GACGATGACTTGTTGAATCATTACCATAATCACATTACTGCACTAGTCTCCAGTTCCCAG
CCAAGGGTGGCGGTGCGGCTGCTTCTCATTCTGACCATGTACTCCCTGCTCGACTTCCAC
GTGCAAGCTACTGTACACGTCTCCT

>SCE_mouse_sequence_908243
CAAGAC

FUGU HITS IN THE CONTEST OF MAMMALIAN rCNE

CLUSTAL W (1.83) multiple sequence alignment

```
ENSMUSG00000027276      AAAAGATCTGGCCTTCAGAAAGCTTCTCATTAAAACACTAATCTGTTCCACCACATTCTT
ENSRNOG00000007443      AAAAGATCTGGCCTTCAGAAAGCTTCTCATTAAAGCACTAATCTGTTCCACCACATTCTT
ENSG00000101384         AAAAGATCTGGCCTTCAGAAAGCCTCTCATTAAAGCGCTAATCTGTTCCACCACATTCTT
ENSCAFG00000005627      AAAAGATCTGGCCTTCAGAAAGTCTCTCATTAAAGCGCTAATCTGTTCCACCACATTCTT
SINFRUG00000154594      ----------------------------------------ACCTAATCCACCGCATTCTT
                                                                * **  ****** *******

ENSMUSG00000027276      CCAAGACACAGCGATCAGCATTGGAGCTGGCTTGAATGGCCAGGCCTAGTAAGACTGATA
ENSRNOG00000007443      CCAAGACACAACAATCAGTGTTGGAGCTGGCTTGAATGGCCGGGCCTAATAAGACTGATA
ENSG00000101384         CCAAGACACAGCGACCAGCATTCTAGCCAGCCTCAATGGCCAGCCCTAATAAGACTGATA
ENSCAFG00000005627      CTGAGACACAGCGATCAGCATTCTAGCCGGCCTCAATGGCTCGTCCTAATAAGACTGATA
SINFRUG00000154594      GTCAGGGGTCCGAGTCTGAATTCAGGCTGGCGTCAATG----------------------
```

```
                           **          * *  **   **  ** * ****

ENSMUSG00000027276      CAAACAGCTTAAGTAATTGGATTGCTATATGGGATTGAATGTCTGACAACAAAAGATAGC
ENSRNOG00000007443      CAAACAGCTTAAGTAATTGGATTGCTATATGGGATTGAATGTCTGACAACAAAAGATAGT
ENSG00000101384         GAAACAGCTTAAGTAATTGGATTGGTATATGGGATTGAATGTCTGACAACAAAAGATAGC
ENSCAFG00000005627      GAAATAGCTTAAGTAATTGGATTGCTATATGGGATTGAATGTCTGACAACAAAAGATAGC
SINFRUG00000154594      ------------------------------------------------------------


ENSMUSG00000027276      CATCTCTATTCGACCACCAAAGCTTTGTTCCAGCAACGAATTCATTCAGCAATGCCCTTA
ENSRNOG00000007443      CATCTCAATTCGACCACCAAAGCTTTGTTCTAGCAATGAATTCATTCAGCAATGCACTTA
ENSG00000101384         CATCTCTATTCGACCACCAAATCTTTGTTCCAGCAATGAATTCATTTAGCAATGTGCTTA
ENSCAFG00000005627      CATCTCTATTCGACCACCATATCTTTGTTCCAGCAATGAATTCATTTAGCAATGCACTTA
SINFRUG00000154594      ------------------------------------------------------------


ENSMUSG00000027276      GAGGCTCCTGGACCCAGGGAGAAATGACAGACCTGAGACACAAAAACTGAGCCAGCTGAT
ENSRNOG00000007443      GAGGCTCCTGGGCCCAGGGAGAAATGACAGACCTGGGACACAAAAACTGGGCCAGCTGAT
ENSG00000101384         GAGGCTCCTGAGCCTGGGCAGAAATAACAGACTGGGGACACAAAAGCTAGGCCAGCTGAT
ENSCAFG00000005627      CAGGCTCCTGGGCCCAGGGAGAAATGACAGACTAGGGACACAAAAGCTAGGCCAGCTGAT
SINFRUG00000154594      ------------------------------------------------------------


ENSMUSG00000027276      GAGCCCAGAGACAGAGGCTATGCAATTCCAAAAAGTTAATTACAGCGAACAAAGGCAGCA
ENSRNOG00000007443      GAGCCCAGAGACAGAGGCTATGCAATTCCAAAAAGTTAATTACAGCGGACAAAGGCAGCA
ENSG00000101384         GAGCCCAGAGACAGAGGCTGCGCAGTTCCAAAAAGTTAATTACAGTGGACAAAGGCAGCA
ENSCAFG00000005627      GAGCCCAGAGACAGAGGCTGCACAATTCCAAAAAGTTAATTATGGTGAACACCAGCAGCA
SINFRUG00000154594      ------------------------------------------------------------


ENSMUSG00000027276      GACGATGACTTGTTGAATCATTACCATAATCACATTACTGCACTAGTCTCCAGTTCCCAG
ENSRNOG00000007443      GACGATGACTTGTTGAGTCATTACCGTAATCACATTACTGCACTAGTCTCCGGTTCCCAG
ENSG00000101384         GACGATGACTTGTTGAATCATTACTATAATCACATTACTGCACTGCTCTGAAGTTCCCAG
ENSCAFG00000005627      GACAATGACTTGTTGAATCATTACCATAATCACATTACTGCACTGGTCTCCAGTTCCCAG
SINFRUG00000154594      ------------------------------------------------------------


ENSMUSG00000027276      CCAAGGGTGGCGGTGCGGCTGCTTCTCATTCTGACCATGTACTCCCTGCTCGACTTCCAC
ENSRNOG00000007443      CCAAGGGTGGCGGTGTGGCTGCTTCTCATTCTGACCATGTACTCCCTACTCATCTTCCAC
ENSG00000101384         CCAAGG-TGGCATTG---CTGCTTCTCATTCTGCCCATGTACTTCATACTCATTTGCCAC
ENSCAFG00000005627      CCAAGGGCGGTGTTG--GCTACTTCTCATTCTGACTCTGTCCTTCATCCTCACTTGCCAC
SINFRUG00000154594      ------------------------------------------------------------


ENSMUSG00000027276      GTGCAAGCTACTGTACACGTCTCCT-
ENSRNOG00000007443      CTACAAACACATG---GTGTCT----
ENSG00000101384         CTGCAATTTACTGTGTGTGACTTCT-
ENSCAFG00000005627      CTGCAATCTTCTGTACATGACTTCTT
SINFRUG00000154594      --------------------------
```

Fragment Jag1_16409

Representation of conserved regions within their genic context

Within the mouse context is shown the rCNE from which the tested SCE is derived (red diamond)

Within the fugu context all CHAOS hits obtained from the mouse rCNE are shown (green diamonds, in red the one tested)

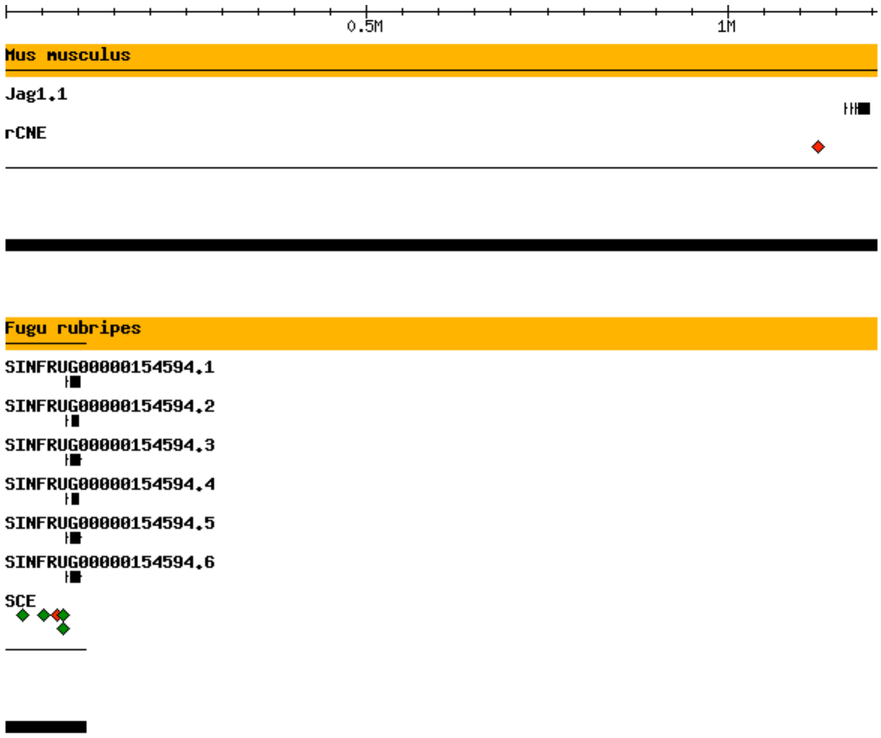

Conserved regions shown within 2000bp context

The mouse context contains:

-rCNEs green boxes, in red the one considered for this test

-SCEs yellow boxes, in red the one considered for this test

-CHAOS hits blue boxes, in red the one considered for this test

The fugu context contains:

-the fragment amplified in this test

-CHAOS hits blue boxes, in red the one considered for this test

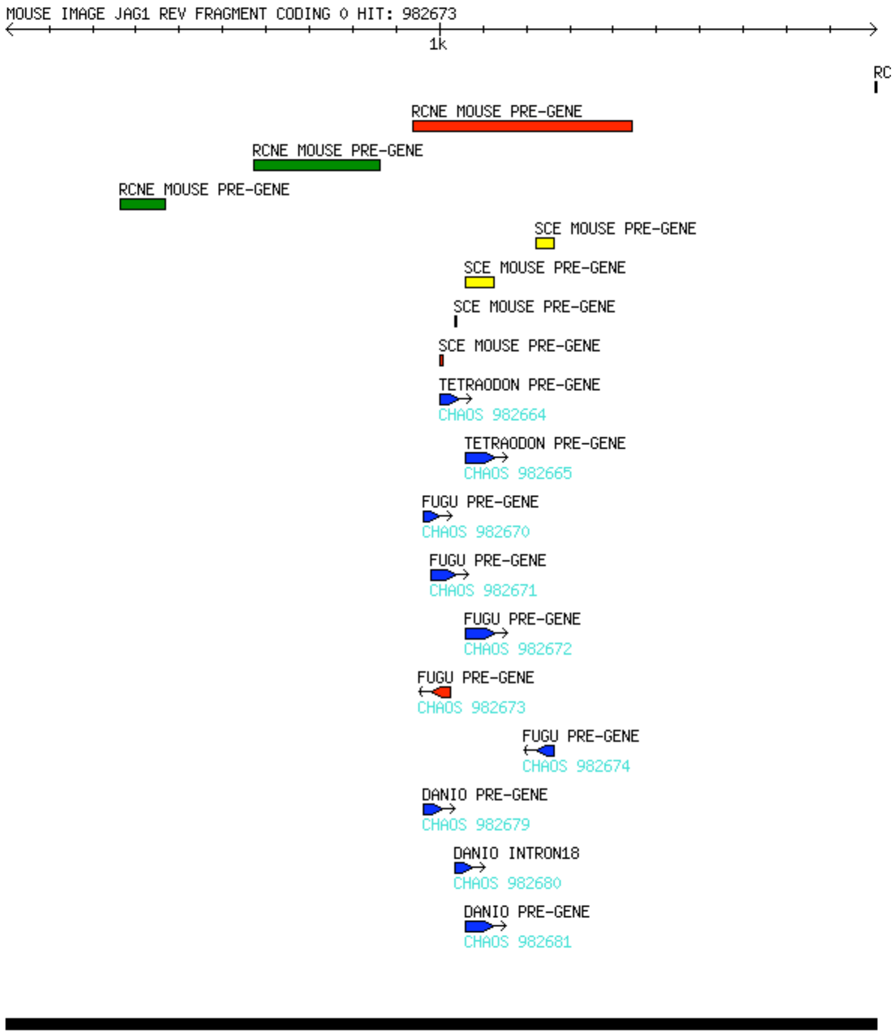

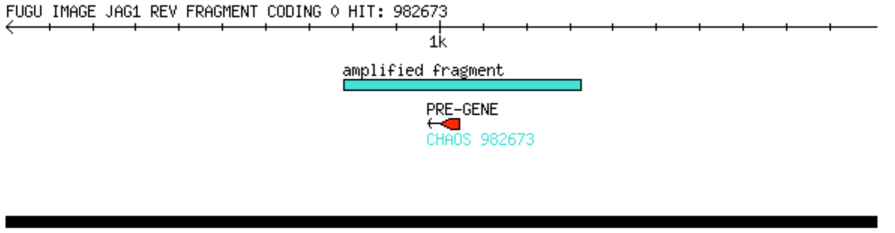

SEQUENCES

```
>fugu_fragment_for_primer_design_16409
AACCAGTAAAAGCTGCAGGAAATAGCTCTTTTTATGTGAAACTGCTACAATAATTAATGT
AAAGAATCACACACTCTGTTTTCAAAGGGATTCTGTATGTAGGGAAGCAACTGTAAGCCC
TCGAAATGTCAAAGATCTTTATCTTTATGATTAATCCTGTCATTCCCAAACACGCACGTC
TCACAATGAGCTGTGAAGCTTAAGATTAAAATGACGCATGGTATTAAGAGATCAGGCACC
TTAACCTTTCTCTCTTTGTGCTGCNNNNNNNNGTTGTTGTGTGATTTTATTATATGTGTT
CCAAAAATGTGCCTACAACCCTTAACATCCACAAAAGACAAATGTGCCTTAGGTGACTCC
GCTGCTCAACAAACCCTGTATCAACCCGAGTCATTTGTCCTCCCACACCTGGGAAATAAC
AACTCTTGTGCGGTTCAGCGTTCAACACCGTCATTTCCACAAAGTTAATCATCAAGCCCA
ATGACCGGGACATCAGTGCTCCAGTGTGCAACTAGTTATTGGACTTTTTGACCAACAGAC
CCCAACATGTTCAGCTAGACCACCAGCGCTCATCCAGCCTCGCCCTTAACACCGGTGTAC
CACAAGGCTGTGTGATGGGTCTGTTCCTTTCCTCCCTTTTCACCCAGGAATTCAGACCTG
```

TCAGGACTCTAAATCTATACACGATCTCCGTCTGGCACGGGCCTCACTGGCAGGCCGCTG
TCCTGGTTTGAATCTGGCCTATCTGGGTGTTCATTGAGTGTGTTGTGGCACGGTCAGGCG
TGCGCTCATTACCAGCTCTCCACTGGGGATTGCTGCTGGGCCCGTTCCTAAGCGAACGTG
CCACCAACCTGCTGGTAGAAGCTACTGTGATCTACCAATTGACTCCGGCAATGCCCCCTT
CTATTCTAACATGAAACTGATGCTTGTGACGTGTGGCTTCAGATGGTCCATACTGTGCAT
CGCTTCTGGTCTTCCACTAATCCAAGTGGGCCCACATCACCCCAGTGCTGATCGAGTTCT
ACTGGCTGCCTGTGGCCGCCCACATTCAGACCAATTCACTTATACTCAGCGGTTCTGCTC
TTTCTTACTTGAGCAATCTTGTTGGAGCCTGGACTACCCCTTGTCCTCATCTGGCACAGC
TACCTGTATAGACAAGGGAACTGAAAGGCCTCATACCAATGCTGGAGCGACCACCCGAGC
TCTGCCAGACCTGTGGCAGCGCTCTCCTCTGTCAGAGCTTTTGAAGACACAGATCTTACA
CCGGTATCCCCATTCACTCCCTCTCCATGGGTCTGCCATGATGGCAGTTGCACTTGTACT
CCATATTTTAGGATTGTAAGGTAGTCTTTGATCGGGGCTCTCGCTGTAAGTCGCTTGTGA
GCCACTCAGCCTCAGAACACATTCTGGGTGTGTCTGTGCACTTCTTCACACTTGCAAATG
CACCTTTTATCCTCTGTTTAACTAGAATGTCTTGTGACCCATCTGAAATGCTCTATAAAC
AGGTGGAACTTCTTGGTTTTATAGCTGCTGTCATACAAACCAATCGCATTTATATCCGTC
CTGGTTTCCAACTTTCTTTGGTTGGAATCGATTAATTTGCATCTTTGAGTAACAGTTAAA
GATAAATGTCCAACATGGTGATAAATGAAAAGAATTGCTGGTTTTTTAAACCAAATCCTT
TCATAAATTTAGACACAGTCGATCGTAGATCTCAGACTCGTGAATAGCTGGACACGGTGG
AAGGTTGCTGGCCAGCTGTGGTTCTGATCCAGCCGCTCGCCTGTGTGGGTCCAGTATGAT
CTTTGTGAGGGAGGGTTGCTGCTATGCGGCTGCTCCACCTAATCCCAAGGCTGCATTACA
CAAGTGCTCCAGTCAAACAGCGCACAGACGTGGGGACTTCCCCTTCCCTCCAAAGACGTA
GAGGAATACTGTATTGCCTCATGCGCCGCTGAGGTCATATCATTACTTCAGCCGAAAGCT
CCAAGGTTAAACCTGAATGTTGCTTTAAGTGTCCAAACATTGGTGGGTGACAGTCCTGAA
TGATC

>amplified_fugu_segment
GTGCGCTCATTACCAGCTCTCCACTGGGGATTGCTGCTGGGCCCGTTCCTAAGCGAACGT
GCCACCAACCTGCTGGTAGAAGCTACTGTGATCTACCAATTGACTCCGGCAATGCCCCCT
TCTATTCTAACATGAAACTGATGCTTGTGACGTGTGGCTTCAGATGGTCCATACTGTGCA
TCGCTTCTGGTCTTCCACTAATCCAAGTGGGCCCACATCACCCCAGTGCTGATCGAGTTC
TACTGGCTGCCTGTGGCCGCCCACATTCAGACCAATTCACTTATACTCAGCGGTTCTGCT
CTTTCTTACTTGAGCAATCTTGTTGGAGCCTGGACTACCCCTTGTCCTCATCTGGCACAG
CTACCTGTATAGACAAGGGAACTGAAAGGCCTCATACCAATGCTGGAGCGACCACCCGAG
CTCTGCCAGACCTGTGGCAGCGCTCTCCTCTGTCAGAGCTTTTGAAGACACAGATCTTAC
ACCGGTATCCCCATTCACTCCCTCTCCATGGGTCTGCCATGATGGCAGTTGCACTTGTAC
TCCA

>rCNE_mouse_sequence_907951
AAAAGATCTGGCCTTCAGAAAGCTTCTCATTAAAACACTAATCTGTTCCACCACATTCTT
CCAAGACACAGCGATCAGCATTGGAGCTGGCTTGAATGGCCAGGCCTAGTAAGACTGATA
CAAACAGCTTAAGTAATTGGATTGCTATATGGGATTGAATGTCTGACAACAAAAGATAGC
CATCTCTATTCGACCACCAAAGCTTTGTTCCAGCAACGAATTCATTCAGCAATGCCCTTA
GAGGCTCCTGGACCCAGGGAGAAATGACAGACCTGAGACACAAAAACTGAGCCAGCTGAT
GAGCCCAGAGACAGAGGCTATGCAATTCCAAAAAGTTAATTACAGCGAACAAAGGCAGCA
GACGATGACTTGTTGAATCATTACCATAATCACATTACTGCACTAGTCTCCAGTTCCCAG
CCAAGGGTGGCGGTGCGGCTGCTTCTCATTCTGACCATGTACTCCCTGCTCGACTTCCAC
GTGCAAGCTACTGTACACGTCTCCT

>SCE_mouse_sequence_908243
CAAGAC

FUGU HITS IN THE CONTEST OF MAMMALIAN rCNE

CLUSTAL W (1.83) multiple sequence alignment

```
ENSRNOG00000007443      AAAAGATCTGGCCTTCAGAAAGCTTCTCATTAAAGCACTAATCTGTTCCACCACATTCTT
ENSCAFG00000005627      AAAAGATCTGGCCTTCAGAAAGTCTCTCATTAAAGCGCTAATCTGTTCCACCACATTCTT
ENSG00000101384         AAAAGATCTGGCCTTCAGAAAGCCTCTCATTAAAGCGCTAATCTGTTCCACCACATTCTT
ENSMUSG00000027276      AAAAGATCTGGCCTTCAGAAAGCTTCTCATTAAAACACTAATCTGTTCCACCACATTCTT
SINFRUG00000154594      ---------------------------------------AATGTGGGCGGCCACAGGCAG
                                                               *** **  *  *****  *

ENSRNOG00000007443      CCAAGACACAACAATCAGTGTTGGAGCTGGCTTGAATGGCCGGGCCTAATAAGACTGATA
ENSCAFG00000005627      CTGAGACACAGCGATCAGCATTCTAGCCGGCCTCAATGGCTCGTCCTAATAAGACTGATA
ENSG00000101384         CCAAGACACAGCGACCAGCATTCTAGCCAGCCTCAATGGCCAGCCCTAATAAGACTGATA
```

```
ENSMUSG00000027276      CCAAGACACAGCGATCAGCATTGGAGCTGGCTTGAATGGCCAGGCCTAGTAAGACTGATA
SINFRUG00000154594      CCAGTAGAACTCGATCAGCACTGGGG----------------------------------
                        *    * *   * * ***   *   *

ENSRNOG00000007443      CAAACAGCTTAAGTAATTGGATTGCTATATGGGATTGAATGTCTGACAACAAAAGATAGT
ENSCAFG00000005627      GAAATAGCTTAAGTAATTGGATTGCTATATGGGATTGAATGTCTGACAACAAAAGATAGC
ENSG00000101384         GAAACAGCTTAAGTAATTGGATTGGTATATGGGATTGAATGTCTGACAACAAAAGATAGC
ENSMUSG00000027276      CAAACAGCTTAAGTAATTGGATTGCTATATGGGATTGAATGTCTGACAACAAAAGATAGC
SINFRUG00000154594      ------------------------------------------------------------


ENSRNOG00000007443      CATCTCAATTCGACCACCAAAGCTTTGTTCTAGCAATGAATTCATTCAGCAATGCACTTA
ENSCAFG00000005627      CATCTCTATTCGACCACCATATCTTTGTTCCAGCAATGAATTCATTTAGCAATGCACTTA
ENSG00000101384         CATCTCTATTCGACCACCAAATCTTTGTTCCAGCAATGAATTCATTTAGCAATGTGCTTA
ENSMUSG00000027276      CATCTCTATTCGACCACCAAAGCTTTGTTCCAGCAACGAATTCATTCAGCAATGCCCTTA
SINFRUG00000154594      ------------------------------------------------------------


ENSRNOG00000007443      GAGGCTCCTGGGCCCAGGGAGAAATGACAGACCTGGGACACAAAAACTGGGCCAGCTGAT
ENSCAFG00000005627      CAGGCTCCTGGGCCCAGGGAGAAATGACAGACTAGGGACACAAAAGCTAGGCCAGCTGAT
ENSG00000101384         GAGGCTCCTGAGCCTGGGCAGAAATAACAGACTGGGGACACAAAAGCTAGGCCAGCTGAT
ENSMUSG00000027276      GAGGCTCCTGGACCCAGGGAGAAATGACAGACCTGAGACACAAAAACTGAGCCAGCTGAT
SINFRUG00000154594      ------------------------------------------------------------


ENSRNOG00000007443      GAGCCCAGAGACAGAGGCTATGCAATTCCAAAAAGTTAATTACAGCGGACAAAGGCAGCA
ENSCAFG00000005627      GAGCCCAGAGACAGAGGCTGCACAATTCCAAAAAGTTAATTATGGTGAACACCAGCAGCA
ENSG00000101384         GAGCCCAGAGACAGAGGCTGCGCAGTTCCAAAAAGTTAATTACAGTGGACAAAGGCAGCA
ENSMUSG00000027276      GAGCCCAGAGACAGAGGCTATGCAATTCCAAAAAGTTAATTACAGCGAACAAAGGCAGCA
SINFRUG00000154594      ------------------------------------------------------------


ENSRNOG00000007443      GACGATGACTTGTTGAGTCATTACCGTAATCACATTACTGCACTAGTCTCCGGTTCCCAG
ENSCAFG00000005627      GACAATGACTTGTTGAATCATTACCATAATCACATTACTGCACTGGTCTCCAGTTCCCAG
ENSG00000101384         GACGATGACTTGTTGAATCATTACTATAATCACATTACTGCACTGCTCTGAAGTTCCCAG
ENSMUSG00000027276      GACGATGACTTGTTGAATCATTACCATAATCACATTACTGCACTAGTCTCCAGTTCCCAG
SINFRUG00000154594      ------------------------------------------------------------


ENSRNOG00000007443      CCAAGGGTGGCGGTGTGGCTGCTTCTCATTCTGACCATGTACTCCCTACTCATCTTCCAC
ENSCAFG00000005627      CCAAGGGCGGTGTT--GGCTACTTCTCATTCTGACTCTGTCCTTCATCCTCACTTGCCAC
ENSG00000101384         CCAAGG-TGGCATT---GCTGCTTCTCATTCTGCCCATGTACTTCATACTCATTTGCCAC
ENSMUSG00000027276      CCAAGGGTGGCGGTGCGGCTGCTTCTCATTCTGACCATGTACTCCCTGCTCGACTTCCAC
SINFRUG00000154594      ------------------------------------------------------------


ENSRNOG00000007443      CTACAAAC------ACATGGTGTCT-
ENSCAFG00000005627      CTGCAATCTTCTGTACATGACTTCTT
ENSG00000101384         CTGCAATTTACTGTGTGTGACTTCT-
ENSMUSG00000027276      GTGCAAGCTACTGTACACGTCTCCT-
SINFRUG00000154594      --------------------------
```

Fragment Lmx1b_17027

Representation of conserved regions within their genic context

Within the mouse context is shown the rCNE from which the tested SCE is derived (red diamond)

Within the fugu context all CHAOS hits obtained from the mouse rCNE are shown (green diamonds, in red the one tested)

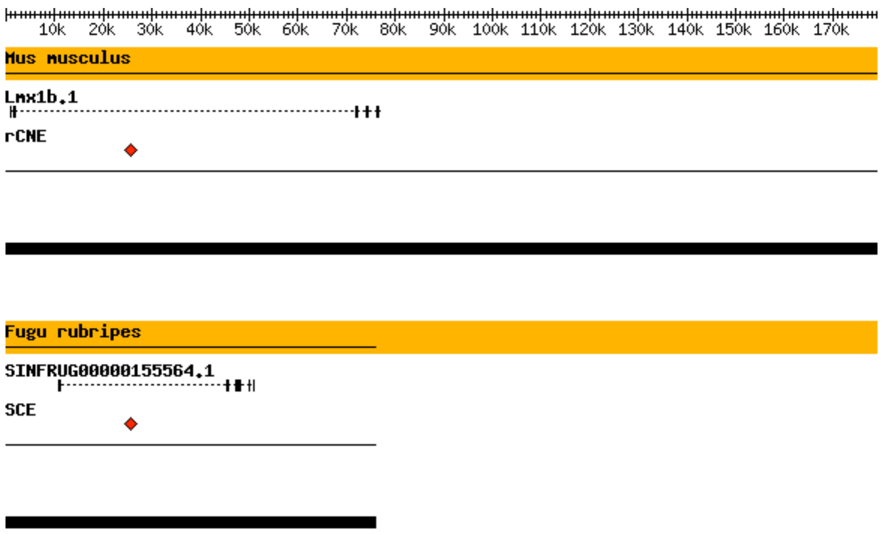

Conserved regions shown within 2000bp context

The mouse context contains:

-rCNEs green boxes, in red the one considered for this test

-SCEs yellow boxes, in red the one considered for this test

-CHAOS hits blue boxes, in red the one considered for this test

The fugu context contains:

-the fragment amplified in this test

-CHAOS hits blue boxes, in red the one considered for this test

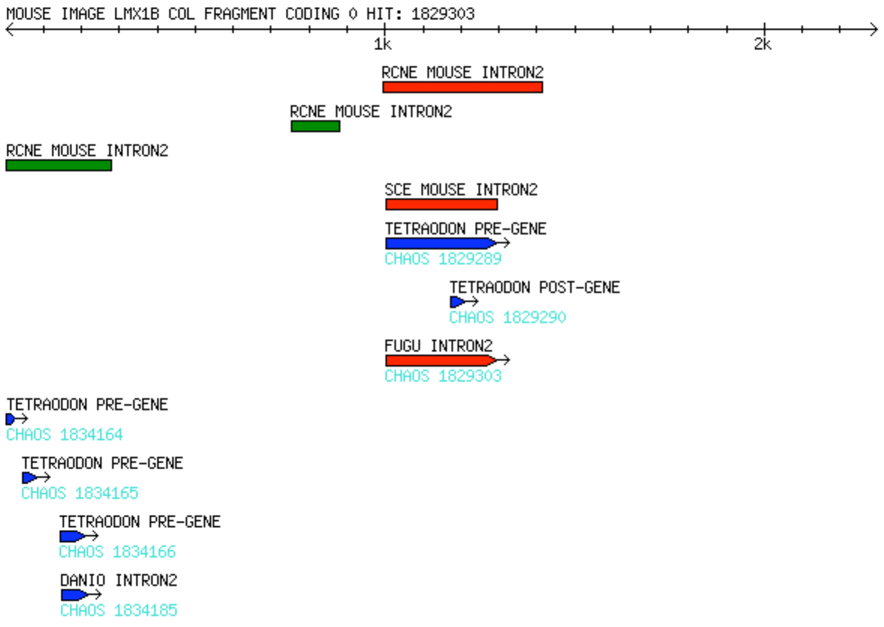

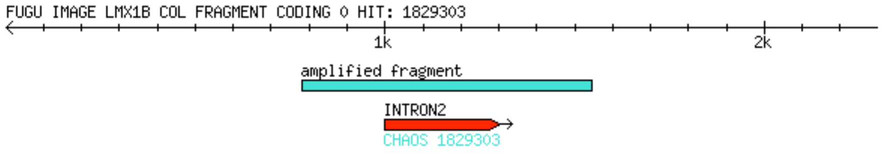

SEQUENCES

```
>fugu_fragment_for_primer_design_17027
GATGGGTAGACGTGCACCTGACCTGAATATAAATGGCACCAGTCCTTCGATAGGTTGGAG
GACGCCACTTCTTTACCCATATCAAATATGCACGCCGTCTCCGTGCGTCCGGTGGGTTAA
ACAAACTAGGATTAATCACCGGGTCGGTGTGGTTTAATCAGGATCGGCCACATTCATCTG
GTTGCAGGAATGTAAACTTAACACTTAAATGTGCGCTTTATATTGTATTTATTTGGTTTG
AAAAGAGATGATTTCTTTATTCCGTTACACTGTTACTACGCAGCTCTCCTTAATGTAATT
GAAGTTACTATTTTTATTCCAGGTGTGTAGGGGAACATTCCTCCCTCCTGGTATTTTAAT
TAATAGCCGCGAGGCATGTAGCAGCCACGTCTCTTTGATGTTTCCTCCTTTACATTCAGG
TGAACCTGGGCTCACAGCTCTTTATTTATGAGGCAATCCCTCACATGTAAAGATGATAAA
GTTGACATTTTACAGGCAGACGACGCTTTCGCATCTCAAGGAGCCGGCTTTGTAAAACAC
CCCAACAAATAGAAGCTTTTAGATTTTTATGGCCAAGTGCCAGCAGGATCTTTTGGAGAT
AAAGCGGCTGCTTCTTTGGTTTGTAAATCCTGTCAGTAATTTTCATAAAGCTAGCTATAT
ACAGTATAACATCTACCTGGGTCANNNNNNNNNNNNNNNNNNNNNNNNNNNNNNNNNNNN
NNNAACGCCGAGCTTTCAGCTAGGAATCATTGCCAGGCAGCCAGCTAATATCCAAAAAGG
TCACCCGCTGTTATGATTGAAGCGTGCACTGGTGCTTTGACCCAACGCACAAAGTGGAGT
TATAGGTGGCCGGTCAGCTTGTCAGTCAGGGTGTGTGGGTTTTTTAATCATTTCATCTTT
GCTGGGGGGCTCTCTTGAGACCCNNNNNNNNNNNNNNNNNNNNNNNNNNNNNNNNNNNNN
NNNNNNNNNNNNNGAGAGAAAGCAGTTGAGGCGATGGGGAGAGGCAGGCAAGCTAGACTG
TCAAATCTCAACCGTTGAATATTTAATGGAGCCTGGAGTGTTTATTTAGTTAGCCTGTCG
ATTTTTACCGCTCTGTCAATAGCGGCTCCCGTGTAGCCTGCTGATGATCTTTAGTGTCTC
TTCCCCCAGGAGAGGCTAGGCACTAAATGCAATTATGGATTTTCCTGCACCGCAATGTGT
TTGATATTTTTACTGATCTAATTTGCAATCTGGTAGTTTATTGTTTGGCATTTTAGTGGC
GCTCAAGTGGCGAGGCAAATTTGATTTCCCTTCATAATTCTTTATAACTTCATTAATGGT
GTTAGCGTATTGGCACGGATCTTAAATAAAGCTTGAGAATCACTGATGGATGAATGCATT
CCACCTTTTATTATGCCTGTGCCCCATTCTTTTCACTGATTCTGGTTCTAATTAAAAAAT
CATCCTGCTGGTGTACTGTCTTTGTGAAATATGAATTCATAAACGGACGCGAATGGCTCC
CTTCACATCGGCATGCGTACGCAGATCAGTCGTGGGCAGAACCAGATTGTAACTATTTTC
```

```
AGAGACAAATAAGCGTATTGGCAACAGAGCAAGAGAGGCAAGCGTGGCACAATAATGCAA
GTGGTTGTTTGGAANNNNNNNNNNNNNNNNNNNNNNNNNNNNNNNNNNNAGTTCCACCAGT
TTTGTGCAGAGCCTTAGGATTTCTCACCCAGTGACGGATGTGTTACTTAGAGGGGTAGCA
TTAGCCGTAATGCTGTTAGCATGTAGCCATGTGAGTGATGTGCTAGTTAGATGCCCAGCA
GTAGAGCTAATGGTGTTAGTGTGTTGAGCCTTCAGAGGGATGCAATCGGTTAGGCTGTGA
TGGATGAGTGTCACGCTGCGGAGGCCTGCAAGCCTCCTCATTCGCCGCGCTGTCAGTGGC
TTAAGTGGACGCGCACAATTAGCACCGAAATCAGAGAGCGCTTAATCTGATGGTAGGCTC
AATGTTCTGTAAGGAAAAAATGGCAGTGGTGAGTGAATGATGACTTAAATGAAAAAAATC
AGTATTGGAGAGAAAACCACTGATAAACAACTCTTTCAGTTTCAGAAGGTGTACAATAAG
TCGTGACGTGATGCTGAGATTCATTTTTGTTCGAGAGGGAAGCTTTATTTCAGCCTTAGA
CCCCACAGTTGCTGCTCAGGCACAAAATGACCACGCACGTGTCGCCAAGAGCCTAATCTC
ACGTCCATGCCAATAGTAAACCAACTTGCTAGACAGGGAGGCATAGTTAGACTCATTATT
TTGGAATCTTTCTAGATTCTT
```

```
>amplified_fugu_segment
GTCACCCGCTGTTATGATTGAAGCGTGCACTGGTGCTTTGACCCAACGCACAAAGTGGAG
TTATAGGTGGCCGGTCAGCTTGTCAGTCAGGGTGTGTGGGTTTTTTAATCATTTCATCTT
TGCTGGGGGGCTCTCTTGAGACCCNNNNNNNNNNNNNNNNNNNNNNNNNNNNNNNNNNNN
NNNNNNNNNNNNNNGAGAGAAAGCAGTTGAGGCGATGGGGAGAGGCAGGCAAGCTAGACT
GTCAAATCTCAACCGTTGAATATTTAATGGAGCCTGGAGTGTTTATTTAGTTAGCCTGTC
GATTTTTACCGCTCTGTCAATAGCGGCTCCCGTGTAGCCTGCTGATGATCTTTAGTGTCT
CTTCCCCCAGGAGAGGCTAGGCACTAAATGCAATTATGGATTTTCCTGCACCGCAATGTG
TTTGATATTTTTACTGATCTAATTTGCAATCTGGTAGTTTATTGTTTGGCATTTTAGTGG
CGCTCAAGTGGCGAGGCAAATTTGATTTCCCTTCATAATTCTTTATAACTTCATTAATGG
TGTTAGCGTATTGGCACGGATCTTAAATAAAGCTTGAGAATCACTGATGGATGAATGCAT
TCCACCTTTTATTATGCCTGTGCCCCATTCTTTTCACTGATTCTGGTTCTAATTAAAAAA
TCATCCTGCTGGTGTACTGTCTTTGTGAAATATGAATTCATAAACGGACGCGAATGGCTC
CCTTCACATCGGCATGCGTACGCAGATCAGTCGTGGGCAGAACC
```

```
>rCNE_mouse_sequence_1586000
TGGGGCCAAGGGGAGGAAGCCTGGATAGTCAAATTTCCATAGCTGAATATTTAATGAGGC
CTGCTGGTTTATTTGGTTGGCCTGTCGATTTTTATTCTTCTGTCAATAGCTCCTCATATG
TGGGGCTGTGGTGATCTTTAGCAGCGCCTCTCCAGGGACGGCTGCCACTAAATGCAATTA
TGGATTTTATACCTCAATGTGTTTGATATTTCCACTGATCTAATTTGCAGCCTGGTAGTT
TATTGTTTGGCATTTTAATGGCGTTCAAATGCTATGGGAAATTTGATTTCTTTTTATAAT
TCCTCCTTAACACAGTTAATGTGAGTGGCGGGATGGCCCAGATTTTAGAGTCCAATGTAT
AGAAAGAAGGTGGGAGGGGGGGCACATGCTTTGATATACTTAAAGATGTGTCCATTGGAG
```

```
>SCE_mouse_sequence_1586058
AAGGGGAGGAAGCCTGGATAGTCAAATTTCCATAGCTGAATATTTAATGAGGCCTGCTGG
TTTATTTGGTTGGCCTGTCGATTTTTATTCTTCTGTCAATAGCTCCTCATATGTGGGGCT
GTGGTGATCTTTAGCAGCGCCTCTCCAGGGACGGCTGCCACTAAATGCAATTATGGATTT
TATACCTCAATGTGTTTGATATTTCCACTGATCTAATTTGCAGCCTGGTAGTTTATTGTT
TGGCATTTTAATGGCGTTCAAATGCTATGGGAAATTTGATTTCTTTTTATAATTCCT
```

FUGU HITS IN THE CONTEST OF MAMMALIAN rCNE

```
CLUSTAL W (1.83) multiple sequence alignment


ENSCAFG00000020170      TGGGACCAAGGGGAGAGGCCTGAGATAGTCAAATTTCCGTTGCTGAATATTTAATGAAGC
ENSG00000136944         TGGGACCAAGGGGAGAGGCCCAGGATAGTCAAATTTCCATTGCTGAATATTTAATGAGGC
ENSMUSG00000038765      TGGGGCCAAGGGGAGGAAGCCTGGATAGTCAAATTTCCATAGCTGAATATTTAATGAGGC
ENSRNOG00000017019      TGGAGCCAAGGGGAGGAAGCCTGGATAGTCAAATTTCCATAGCTGAATATTTAATGAGGC
SINFRUG00000155564      -------AGAGGCAGGCAAGCTAGACTGTCAAATCTCAACCGTTGAATATTTAATGGAGC
                               *  ** **        **  ******* **    * *************  **

ENSCAFG00000020170      CTGCACGGTTTATTTGGTTGGCCTGTCGATTTTTATTCCTCTGTCAATAGCTTCTCATAT
ENSG00000136944         CCATGCGGTTTATTTGGTTGGCCTGTCGATTTTTATTCTTCTGTCAATAGCTTCTCATAT
ENSMUSG00000038765      CTGC-TGGTTTATTTGGTTGGCCTGTCGATTTTTATTCTTCTGTCAATAGCTCCTCATAT
ENSRNOG00000017019      CTGC-CGGTTTATTTGGTTGGCCTGTCGATTTTTATTCTTCTGTCAATAGCTCCTCATAT
SINFRUG00000155564      CTGGAGTGTTTATTTAGTTAGCCTGTCGATTTTTACCGCTCTGTCAATAGCGGCTCCCGT
                        *      ******** *** ***************    ************  ***   *

ENSCAFG00000020170      GTGGGGCTGTGATGATCTTTAGCAGGGCCTCTCC-AGGGACGGCT-GCCACTAAATGCAA
ENSG00000136944         GTGGGGCTGTGATGATCTTTAGCAGGGCCTCTCC-AGGGACGGCT-GCCACTAAATGCAA
```

```
ENSMUSG00000038765      GTGGGGCTGTGGTGATCTTTAGCAGCGCCTCTCC-AGGGACGGCT-GCCACTAAATGCAA
ENSRNOG00000017019      GTGGGGCCGTGGTGATCTTTAGCAGCGCCTCTCC-AGGGACGGCT-GCCACTAAATGCAA
SINFRUG00000155564      GTAGCCTGCTGATGATCTTTAGTGTCTCTTCCCCCAGGAGAGGCTAGGCACTAAATGCAA
                        ** *     ** **********     * ** ** ***   **** * ************

ENSCAFG00000020170      TTATGGATTTT---ATACCTCAATGTGTTTGATATTTCCACTGATCTAATTTGCAGCCTG
ENSG00000136944         TTATGGATTTT---ATACCTCAATGTGTTTGATATTTCCACTGATCTAATTTGCAGCCTG
ENSMUSG00000038765      TTATGGATTTT---ATACCTCAATGTGTTTGATATTTCCACTGATCTAATTTGCAGCCTG
ENSRNOG00000017019      TTATGGATTTT---ATACCTCAATGTGTTTGATATTTCCACTGATCTAATTTGCAGCCTG
SINFRUG00000155564      TTATGGATTTTCCTGCACCGCAATGTGTTTGATATTTTTACTGATCTAATTTGCAATCTG
                        ***********     *** *****************  ****************  ***

ENSCAFG00000020170      GTAGTTTATTGTTTGGCATTTTAATGGCGTTCAAATGCTATGGGAAATTTGATTTCTTTT
ENSG00000136944         GTAGTTTATTGTTTGGCATTTTAATGGCGCTCAAATGCTATGGGAAATTTGATTTCTTTT
ENSMUSG00000038765      GTAGTTTATTGTTTGGCATTTTAATGGCGTTCAAATGCTATGGGAAATTTGATTTCTTTT
ENSRNOG00000017019      GTAGTTTATTGTTTGGCATTTTAATGGCGTTCAAATGCTATGGGAAATTTGATTTCTCTT
SINFRUG00000155564      GTAGTTTATTGTTTGGCATTTTAGTGGCGCTCAAGTGGCGAGGCAAATTTGATTTCCCTT
                        *********************** ***** **** **    ** ************  **

ENSCAFG00000020170      TATAATTCCTCCTTAACGCAGTTAATGTGGGTGGCGGGATGGCCCAGATTTTAGAGCCCA
ENSG00000136944         TATAATTCCTCCTTAACGCAGTTAATGCGGGTGGCGGGATGGCCCAGATTTTAGAGCCCA
ENSMUSG00000038765      TATAATTCCTCCTTAACACAGTTAATGTGAGTGGCGGGATGGCCCAGATTTTAGAGTCCA
ENSRNOG00000017019      TATAATTCCTCCTTAACACAGTTAATGTGAGTGGCGGGATGGCCCAGATTTTAGAGTCCA
SINFRUG00000155564      CATAATTCTT--------------------------------------------------
                         ******* *

ENSCAFG00000020170      ATGTATAGAAAGGAAAAGTNNNNNNNNNNNNNA----ACATACCCTTGTATATATTTAAG
ENSG00000136944         ATGTATAGAAAGAAGAAGGGAGGAGGAAAAAAAGAGAACACCCCGTTGTATATATTTAAA
ENSMUSG00000038765      ATGTATAGAAAGAAGGTGGGAGGGGGGG----------CACATGCTTTGATATACTTAAA
ENSRNOG00000017019      ATGTATAGAAAGAAG-TNNNNNNNNNAG----------CACACACTTTGATATACTTAAA
SINFRUG00000155564      ------------------------------------------------------------


ENSCAFG00000020170      GATGTGTCCATTGAAG
ENSG00000136944         GATGTGTCCGTTGAAG
ENSMUSG00000038765      GATGTGTCCATTGGAG
ENSRNOG00000017019      GATGTGTCCATTGGAG
SINFRUG00000155564      ----------------
```

Fragment Mab21l2_23001

Representation of conserved regions within their genic context

Within the mouse context is shown the rCNE from which the tested SCE is derived (red diamond)

Within the fugu context all CHAOS hits obtained from the mouse rCNE are shown (green diamonds, in red the one tested)

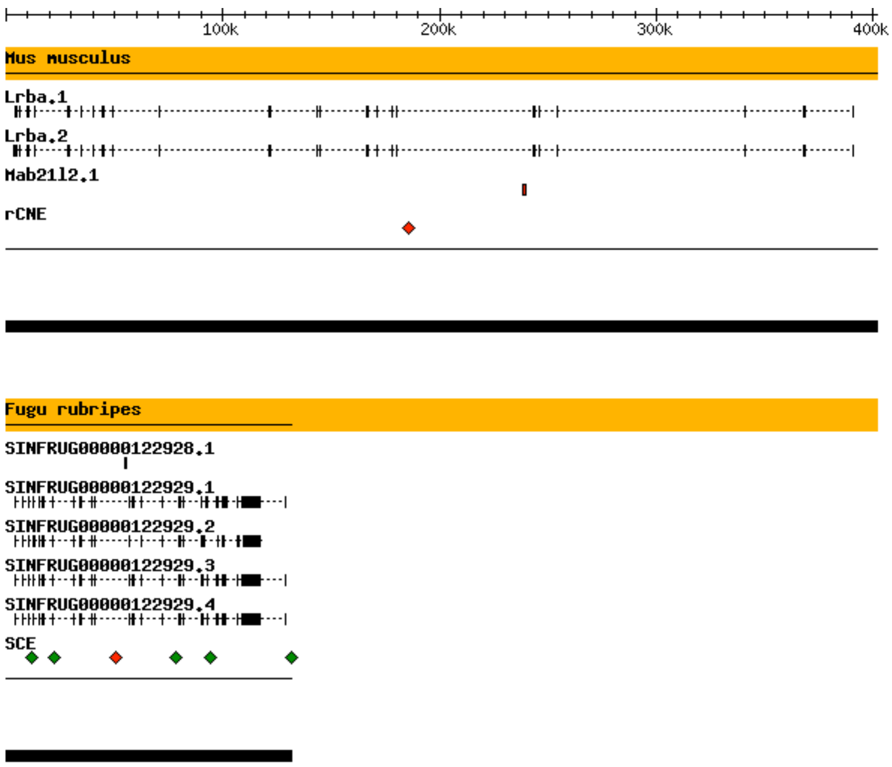

Conserved regions shown within 2000bp context

The mouse context contains:

-rCNEs green boxes, in red the one considered for this test

-SCEs yellow boxes, in red the one considered for this test

-CHAOS hits blue boxes, in red the one considered for this test

The fugu context contains:

-the fragment amplified in this test

-CHAOS hits blue boxes, in red the one considered for this test

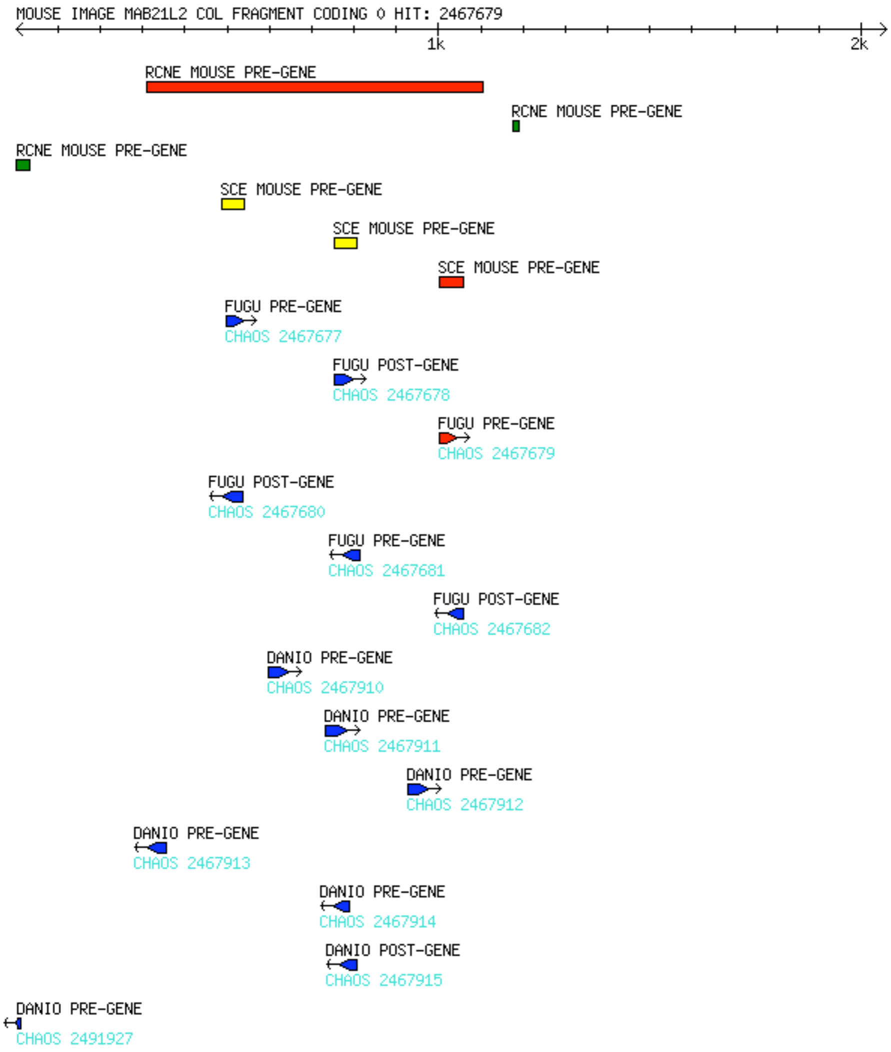

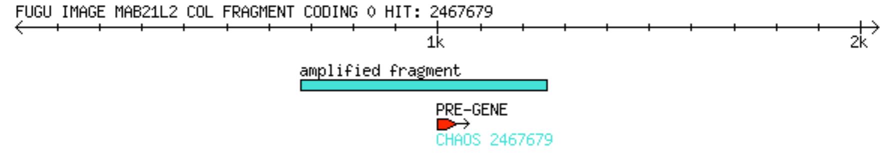

SEQUENCES

>fugu_fragment_for_primer_design_23001
TCAGGCGTCATTAATGCCTGGTTGTAATATACAGTGATGTAACGTCAACGGCAGCTACTG
GCCATGACTGGAACCAGATCCTGCAGGCAAACAAGTAATTTGTTNNNNNNNNNNGTTGTTA
GAAAAATTAAAGGAACAAGTCTTCCCCGTCTAATAAAGGAATGGCCTTACTGGAAAACAG
GATGAGGGGAAAGAGCGAGGGAACGGAGAGAGAATGAAAAAGAAGGTCATCCCAACATCA
CTCCACGAGTTGTTGTTGCGGCGCAGTTGAGCGATTTATCGACCAGCTCAGGAACCGCTC
TGTCAGAGGGGACGAGGAGGTGCGACAGCATGAGGGAGGCAGAGGAAGAGACTGACACAG
GCCAATATGAATGACCGAGAGTGAGGGGATCCGTTTCCTTGGCCTCATTGATAATGACAT

ACCTGCTTGAGCTGTCCGTTAGTCCTATAGCACACACTACTTAGGCTCTAACTGGAAAAT
CAATAGGCTCCTCTCTCGGAGACCCCGCGCTGCCATGGCTCCCTGTGATTGGCAGCTGCA
GTCGAAGGTCGACCCCGACGAGCATGAGAGTTGGGGAATGAGCGCGGCGGTNNNNNNNNN
NNNNNCCCGGTCCCTGTAGTTTGACACTTTAGGGAGAGCTTAACGATATGGGACTGGCCG
CTACATAACGCCAGGGTGGTTTTCCCTGACTGAGTGACCATCCATTCAACCGCTCACGCT
AACCCCGAGGAGCCGTCACCACCCCGCTAATTAGAGCTACCAATGCCTCCTGAATGGGAG
TTTTTATGATTGTGTAGTCTGAGGTTCAGCACTGACCTTTTGGAAAAATTTAGTGTAGTA
TTCCTACCAAAAATATATACTGCTAGAGTTTTTGGAAACGCCACAAAAATTAAAATTGTA
CGTGTTTAACAAAACTAAATACAGTTAATTTAGGTTTTTAATACTTTGGAAATAAAGGGA
AGGTTAAAATAACATGAACGAAAAAGAATGTACTATAACATTTGGTTTTAAGCAGGTAAA
AGTCTTAATTATACTTTACATGCTTTGACAGTCCAATATAAAAATGTTAAGAATAAGCAA
CACAGGAGAAACTTCTCTTGCTAAATTGCAGTTATATTATTTTTATCAAATGTATTATTC
CTAACTATTCATGCACAAACTGTATGAAATAAAAAACATAACTTTCACTTTTTTCCCAAG
TTCAAAATATTTACAGTGTAGTTGGTATTAATCTATGGTGGCTCTAGACTGCCAAAATCT
ACATTAATATTATTAGTACAATTTTAAAAGTAGTTAAACTTACCAAATAAAACTAAGATT
ACTTTCTTCATTTAAGCATTAATTATTATAGCTGGACCAGATGCTGGAATAAAAGTAGAA
ACCATTAGTAATTTCCATTCAATACCACATTGAAATTGCAATCAGGATTTGAGAGACAGA
CCCTCCAATCAGATTTCTGCTGTGCTTCCGTTGACCAATGAGGTTGCAGTTCAGTGAATT
TCACTTCCTCATTCATGCAATATGCAACGGTCTCCGGGAGCGGCTCTGCAACAATAACTG
TACATTCAATATTGATGCTTCCATCTAAAGACACGGTATTAACCCGCACACACACGCACG
TGCACGCAACAAACAGCTGCTCCGTGACTTCATCAGCTCTGCTCTCTTATGAACAGATGC
AACAGCTCTGCCAGCCAATTAGCTGCCTGTGATAACTTTCTTTGAGCCCCCTGTGAGGAG
AGCAGCTGGGTCCATCCGGGTTTGGAGGGAGGGAAAGAACGTGTGAACAAGAGGGTAAAA
ACAGCGCTCGCTGTGTTCCTGGTTGGGAGAAGGGCACTTTCACGAGCACTAGTGGATGCA
GCAAAGCTTGACCTCGGACTTAATCATTCAGAATTCAAGCAGTTTGTGACAACAGCTTGT
GTAGGTGGGGGTTCCCATCCTGATCAGGTAGAGGGGAATAAAACCGAAGCACAGCTGGGA
CAAATGGAAGACGAGAAGAAAGGGGAAGCAGAGGAGATGTGCACAGGCATGGACACTGTT
TTT

>amplified_fugu_segment
GGTGGTTTTCCCTGACTGAGTGACCATCCATTCAACCGCTCACGCTAACCCCGAGGAGCC
GTCACCACCCCGCTAATTAGAGCTACCAATGCCTCCTGAATGGGAGTTTTTATGATTGTG
TAGTCTGAGGTTCAGCACTGACCTTTTGGAAAAATTTAGTGTAGTATTCCTACCAAAAAT
ATATACTGCTAGAGTTTTTGGAAACGCCACAAAAATTAAAATTGTACGTGTTTAACAAAA
CTAAATACAGTTAATTTAGGTTTTTAATACTTTGGAAATAAAGGGAAGGTTAAAATAACA
TGAACGAAAAAGAATGTACTATAACATTTGGTTTTAAGCAGGTAAAAGTCTTAATTATAC
TTTACATGCTTTGACAGTCCAATATAAAAATGTTAAGAATAAGCAACACAGGAGAAACTT
CTCTTGCTAAATTGCAGTTATATTATTTTTATCAAATGTATTATTCCTAACTATTCATGC
ACAAACTGTATGAAATAAAAAACATAACTTTCACTTTTTTCCCAAGTTCAAAATATTTAC
AGTGTAGTTGGTATTAATCTATGGTGGCTCTAGACTGCCAA

>rCNE_mouse_sequence_2026043
TCCTTAACATTTTCATGGGTGACAAATTCAATTAAATATATTCATTACTCTAATGTTCGA
CTTGGCAGCTTTAGTCAGCATGNNNNNNNNNNNNNNNNNNNNNNNNNGTTGTATGACCTT
TGTATTGATTGGGCTAAAATGGCTTTAGCCTGGGTGTGGAAGATGGGGCAGTTGGGAGGG
AAGACAGAAAAGCACACAAATACACACACTCTTATGTTTTCTCTTCCAATTACTGAGGGA
GATGATAAAACCACAGCAAATGATGTATCAGCTCATTTTATCTTATCGCTTTGGGGCTAG
GTGACAATGAAGTTCATATTTCACTGGCTCCCATTGAAGATTGTGAAAATTACTTGCCCT
CATGACCTGAAATTTCACTAGAAGACAGAAATTTCTCTTCTGCAAAGTAATGCAATTTTT
CAGATGTCAACCTTTAGCCCAGCTTTAAAAATTAATTTCATTTATATTAACCAATGATAT
TGATATTTTCACAGAACGCTCTTTGATACATGAAGCACTGACTGGCAAATTGATGTTGCT
CTAGATGGGCAAAACTATTTTACTTTAAGCTGCGATGGCAGGCAGATTTATGGCAAACTG
AAATTCCCATTTCATAACTTAATATTTGTGCTGGAGCAATGCACCTTTAAGCCTTTGGCC
TGTAAATAATTCGGACTATTTACTTGGTTCTATTTTATTTTCAACATTCCTCAGCTTAAA
AAATAATTTACATTCTACAGGATATATTATAAAATCCAGNNNNNNNNNAACTAGTAGTTT
TTATATAGCGTAATGAG

>SCE_mouse_sequence_2026320
ATTTTATTTTCAACATTCCTCAGCTTAAAAAATAATTTACATTCTACAGGATATATTAT

FUGU HITS IN THE CONTEST OF MAMMALIAN rCNE

CLUSTAL W (1.83) multiple sequence alignment

ENSMUSG00000057777   TCCTTAACATTTTCATGGGTGACAAATTCAATTAAATATATTCATTACTCTAATGTTCGA

```
ENSCAFG00000007960      TCCTTAGCATTTTCATGGGTGACAAATTCAATTAAACATATTCATTACTCTAATGTTCTA
ENSRNOG00000031398      TCCTTAACATTTTCATGGATGACAAATTCAATTAAATATATTCATTACTCTAATGTTCTA
ENSG00000181541         ---ATAGCATTT-CATGGGTGACAAATTCAATTAAACATATTCATTACTCTAATGTTCTA
SINFRUG00000122928      -----------------------------------------------------------A
                                                                                   *

ENSMUSG00000057777      CTTGGCAGCTTTAGTCAGCATGNNNNNNNNNNNNNNNNNNNNNNNNNG-TTGTATGACCT
ENSCAFG00000007960      CTTGGCAG-TGTAGTCAGTATGNNNNNNNNNNNNNNNNNNNNNNNNNNGCTACGTGACCT
ENSRNOG00000031398      CTTGGCAGCTTTAGTCAGCATGNNNNNNNNNNNNNNNNNNNNNNNNGTGCTGTATGACCT
ENSG00000181541         CTTGGCAGTTTTAGTCAGTATGNNNNNNNNNNNNNNNNNNNNNNNNNNGCTATGTGACCT
SINFRUG00000122928      TTTGGT---TTTAAGCAG-GTAAAAGTCTTAATTATACTTTACATGCT------------
                         ****    * **  ***  *

ENSMUSG00000057777      TTGTATTGATTGGGCTAAAATGGCTTTAGCCTGGGTGTGGAAGATGGGGCAGTTGGGAGG
ENSCAFG00000007960      CTGTATTGATTGGGCTAAAATGGTTTTAGCCTGGGTGTGGATGATGGGGCAGCTGGGAGG
ENSRNOG00000031398      TTGTATTGATTGGGCTAAAATGGCTTTAGCCTGGGTGTGGATGATGGGGCAGTTGGGAGG
ENSG00000181541         TTGTATTGATTGGACTAAAATGGCTTTAGCCTGGGTGTGGACGATGGGGCAGTTGGGAGG
SINFRUG00000122928      ------------------------------------------------------------

ENSMUSG00000057777      GAAGACAGAAAAGCACACAAATACACACACTCTTATGTTTTCTCTTCCAATTACTGAGGG
ENSCAFG00000007960      GGCAAGAGGGGAGCACACAAACACATACACTCTTATGTTTTCTCTTCCAATTACTGAGGG
ENSRNOG00000031398      GGAGAGAGAAAAGCATACAAACATACACACTCTTATGTTTTCTCTTCCAATTACTGAGGG
ENSG00000181541         GGCAAGAGGGGAGCCTACAAACACACACACTCTTATGTTTTCTCTTCCAATTACTGAGGG
SINFRUG00000122928      ------------------------------------------------------------

ENSMUSG00000057777      AGATGATAAAACCACAGCAAATGATGTATCAGCTCATTTTATCTTATCGCTTTGGGGCTA
ENSCAFG00000007960      AGATGATAAAACCACAGCAAATGATGTATCAGCTCATTTTATCTTATCGCTTTGGGGCTA
ENSRNOG00000031398      AGATGATAAAACCCCAGCAAATGATGTATCAGCTCATTTTATCTTATCGCTTTGGGGCTA
ENSG00000181541         AGATGATAAAACCACAGCAAATGATGTATCAGCTCATTTTATCTTATCGCTTTGGGGCTA
SINFRUG00000122928      ------------------------------------------------------------

ENSMUSG00000057777      GGTGACAATGAAGTTCATATTTCACTGGCTCCCATTGAAGATTGTGAAAATTACTTGCCC
ENSCAFG00000007960      GGTGACAATGAAGTTCATATTTCACTGGCTCCCATTGAAGATTGTGAAAATTACTTGCCC
ENSRNOG00000031398      GGTGACAATGAAGTTCATATTTCACTGGCTCCCATTGAAGATTGTGAAAATTACTTGCCC
ENSG00000181541         GGTGACAATGAAGTTCATATTTCACGGGCTCCCATTGAAGACTGTGAAAATTACTTGCCC
SINFRUG00000122928      ------------------------------------------------------------

ENSMUSG00000057777      TCATGACCTGAAATTTCACTAGAAGACAGAAATTTCTCTTCTGCAAAGTAATGCAATTTT
ENSCAFG00000007960      TCATGACCTGAAATTTCACTAGAAGACAGAAATTTCTCTTCTGCAAAGTAATGCAATTTT
ENSRNOG00000031398      TCATGACCTGAAATTTCACTAGAAGACAGAAATTTCTCTTCTGCAAAGTAATGCAATTTT
ENSG00000181541         TTATGACCTGAAATTTCACTAGAAGACAGAAATTTCTCTTCTGCAAAGTAATGCAATTTT
SINFRUG00000122928      ------------------------------------------------------------

ENSMUSG00000057777      TCAGATGTCAACCTTTAGCCCAGCTTTAAAAATTAATTTCATTTATATTAACCAATGATA
ENSCAFG00000007960      TCAGATGTCAACCTTTAGCCCAGCTTTAAAAATTAATTTCATTTATATTAACCAATGATA
ENSRNOG00000031398      TCAGATGTCAACCTTTAGCCCAGCTTTAAAAATTAATTTCATTTATATTAACCAATGATA
ENSG00000181541         TCAGATGTCAACCTTTAGCCCAGCTTTAAAAATTAATTTCATTTATATTAACCAATGATA
SINFRUG00000122928      ------------------------------------------------------------

ENSMUSG00000057777      TTGATATTTTCACAGAACGCTCTTTGATACATGAAGCACTGACTGGCAAATTGATGTTGC
ENSCAFG00000007960      TTGATATTTTCACAGCACGCCCTTTGATACATGAAGCACTGACTGGCAAATTGATGTTGC
ENSRNOG00000031398      TTGATATTTTCACAGAACACTCTTTGATACATGAAGCACTGACTGGCAAATTGATGTTGC
ENSG00000181541         TTGATATTTTCACAGAACACTCTTTGATACATGAAGCACTGACTGGCAAATTGATGTTGC
SINFRUG00000122928      ------------------------------------------------------------

ENSMUSG00000057777      TCTAGATGGGCAAAACTATTTTACTTTAAGCTGCGATGGCAGGCAGATTTATGGCAAACT
ENSCAFG00000007960      TTTAGATGGGTAAAACTATTTTATTTTAAGCTGCTATGGCAGACAGATTTATAGCAAACT
ENSRNOG00000031398      TTTAGATGGGCAAAACTACTTTACTTTAAGCTGCGATGGCAGGCAGATTTATGGCAAACT
ENSG00000181541         TTTAGATGGGTAAAACTATTTTATTTTAAGCTGCTGTGGCACACAGATTTATGGCAAACT
SINFRUG00000122928      ------------------------------------------------------------

ENSMUSG00000057777      GAAATTCCCATTTCATAACTTAATATTTGTGCTGGAGC-AATGCACCTTTAAGCCTTTGG
ENSCAFG00000007960      GAAATTCCCATTTCATAATTTAATATTTGTGCTAGAGT-GATGCACTTTTAAGCCCCTGG
ENSRNOG00000031398      GAAATTCCCATTTCATAACTTAATATTTGTGCTGGAGA-AATGCACCTTTAAGCCTTTGG
ENSG00000181541         GAAATTCCCATTTCATAATTTAATATTTGTGCTGGAGTTGATGCACCTTTAAGCCCTTGG
SINFRUG00000122928      ------------------------------------------------------------

ENSMUSG00000057777      CCTGTAAATAATTCGGACTATTTACTTGGTTCTATTTTATTTTCAACATTCCTCAGCTTA
ENSCAFG00000007960      CCTGTAAATAATTCAGACTCTTTAGTTGGTTCTGTTTTATTTTCAATATAATTCAGCTTA
ENSRNOG00000031398      CCTGTAAATAATTCAGACTCTTTACTTGGTTCTATTTTATTTTCAACATTCCTCAGCTTA
ENSG00000181541         CCTGTAAATAATTCAGACTCTTTACTTGGT--TGTTTTATTTTCAATATTCCTCAGCTTA
SINFRUG00000122928      ------------------------------------------------------------

ENSMUSG00000057777      AAAAATAATTTACATTCTACAGGATATATTATAAAATCCAGNNNNNNNNNNAACTAGTAGT
ENSCAFG00000007960      AAAAATAATTTGCATTCTAAATGATGTATCATAAAAGCCATCTTTTATTGAAC----ATT
ENSRNOG00000031398      AAAA-TAATTTATATTCTATATGATACATTATAAAATC---CTGATTTTTAACTGGAAGT
ENSG00000181541         AAAA-TAATTTGCATTCTAAATGATGCATCTTAAAAGCCATCTCTTATTCAACTAGCATT
SINFRUG00000122928      ------------------------------------------------------------
```

```
ENSMUSG00000057777      TTTTATATAGCGTAATGAG
ENSCAFG00000007960      TAATATGTATCTTAATGAG
ENSRNOG00000031398      TACTATATAGCTTAATGAG
ENSG00000181541         AAATATCAATCTTAATGAG
SINFRUG00000122928      -------------------
```

Fragment Mab21l2_23002

Representation of conserved regions within their genic context

Within the mouse context is shown the rCNE from which the tested SCE is derived (red diamond)

Within the fugu context all CHAOS hits obtained from the mouse rCNE are shown (green diamonds, in red the one tested)

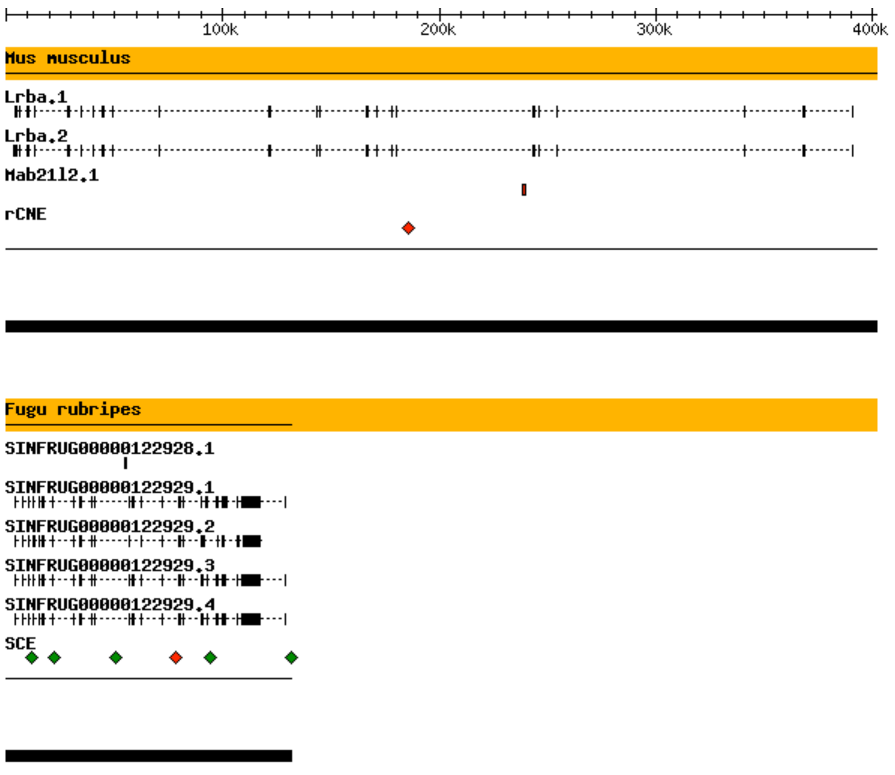

Conserved regions shown within 2000bp context

The mouse context contains:

-rCNEs green boxes, in red the one considered for this test

-SCEs yellow boxes, in red the one considered for this test

-CHAOS hits blue boxes, in red the one considered for this test

The fugu context contains:

-the fragment amplified in this test

-CHAOS hits blue boxes, in red the one considered for this test

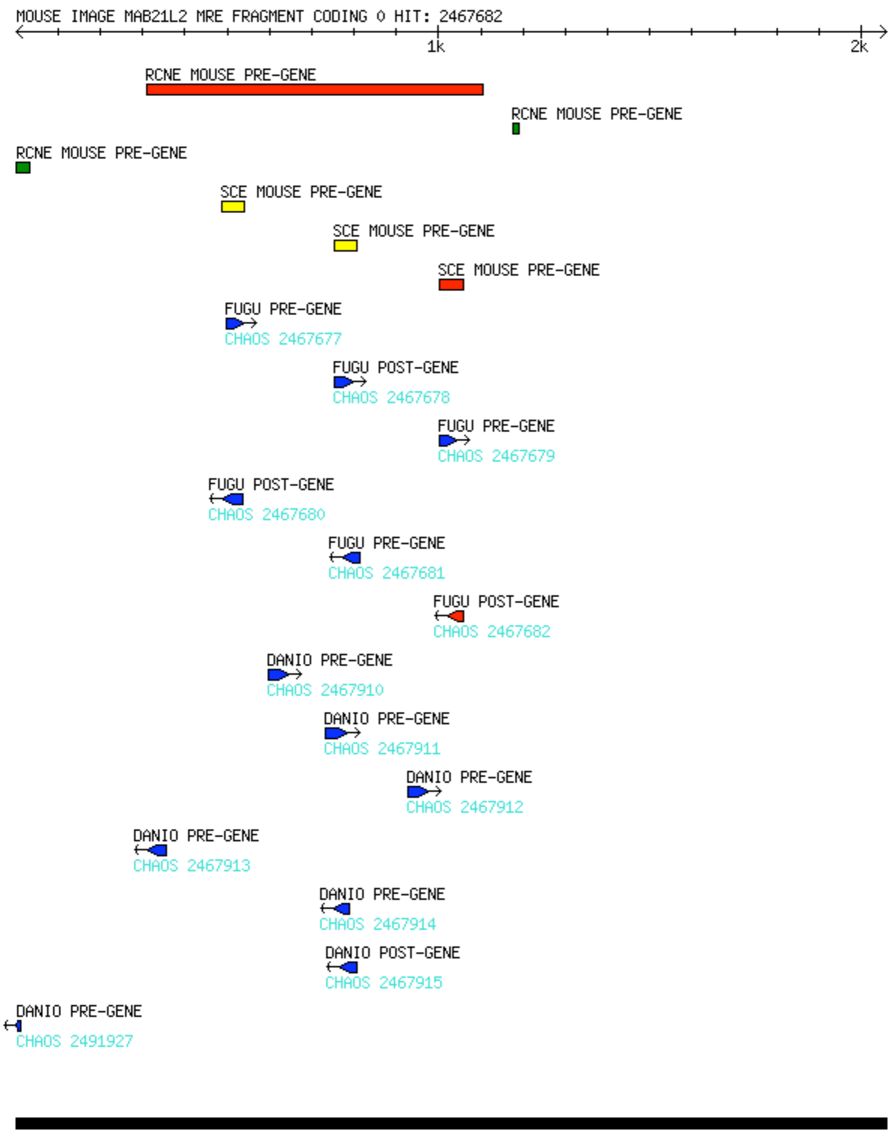

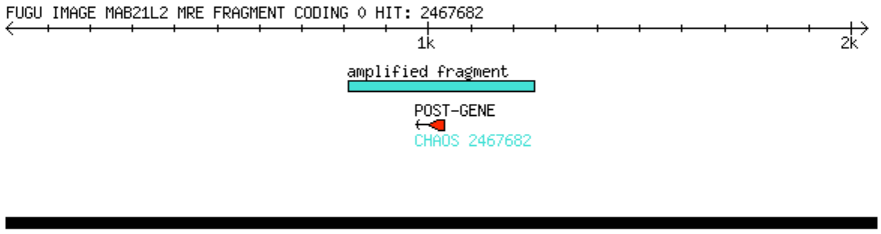

SEQUENCES

```
>fugu_fragment_for_primer_design_23002
GGCTTAAGGTGGAGGATCTATTACATCAGTACTGGTCTGTAAGCACCTGAAAAATATTAC
TGTGAAATCAAATGATTGTTGGAAANNNNNNNNNNNNNNNNNNNNNNNNNCTACACTGCACA
AGCTTGGAGGTAATATATTCCGGGGGCAATCGGGATTCGACTCACTGGTTTTCAGCTCCT
TCAATTCACACAGAGCCATGGAGGAGCCTTCTAAAGTGGTGCCCTGATTAAATGTTAACT
TGCTGTTTGTGTAAAGTCTGAGCTGGACTTCTATTAAAGTATGAAAACACATCGCCACCT
GGGTAATTTCTGATATTATTAAAACCACTAGAGGAAAAATAAGGATACTAAAAGCATTAA
ACACAGGTGACCAGGTTGGTCATAATTTTGCTTTTTTGTGTTGTGGCTGTTGTCAAGTAT
CTGCTATTTTTATTTCCGATCATTTTAACTATATGGATACATTTTAATTAATTTACAGCT
TTGTAAAACATTTCAGTGAAGGAGGTTTAAGCTACTGGTGCTGCACAATAAAGCAAACCT
CATTTTGAGTCCATCGTGGCTGGAATTAGTCATTCTAGACACATCATTTCATGGGTTTCC
ACACAACAAAAACCTATAAAGTCCCATCAACCCAACACAATAGAACAATGCCATAAAAAA
CTGGAACAGAGCTGAAAATTATTTCAAAATCCGCCACCCACGCCCTCAGGGGGATGTTTC
ATTGGCCTGTTATTTTTATTGTGGCAGAGCAAAATGAAACCCACGATTCCAGCGGGCTCA
GGCAGAGCTTGTATAGAGGCAAAAATCAATAGGGCAATATCAGAGCGAGACTTATCAAGG
GTTTTTCTTCTTCTCAGGGGTCACTGAGGAGCAACTGCATTTAAGTGGGCAATTCAATTG
TGAGACATAGTTGGGGACGAGCTGCAGTGAGTCTGATTAAAGTCGGTCCAGAGACCACAA
AGATAAGCTTATAATGACAGCAAGAGAATGAACCTAATAACAAGGTATACTTTTGAATTT
GTCGTGTTTTTTAACCTGACCCTGCCATGCTTATTTTAAAGCTTATTTTCATTGTTAAAC
ATTAATACTCTACTCATTCTAATAGTTTTATAGCAATTTATTTTAGTAACATCTATAGTG
AAGATCCAGAAATATGGTAGAGCAGAGAGCAAATGGACCAAAACACACATAAGACTAATT
CAGGAATGAATAAATCTAGAGACTGAGGTCGAACAGGAACATTTGAGGGCTCCCTTGTGC
AGATCAGCCGCCAAGTCATTCTGTCTGTCGGTCAGGCTCCATATGAAGAGGAAATTTCCC
ATGAGCTCCCCTGATGAGGACAGCCCTCACTATGGACGCACTATTTCCTTGCTTCTAAAA
AGGCATGTACACTTTTAGATCAGGCATTCACGAGGCAGCCTCATTAGAGACGGGACACGG
AAGGCATCGAGCAATAGGCTTGGCTGGACCCTGGAAAAGACACCTGTCCATTAGAGGACA
TACACCCCATACAAATCCATCCAAGCATGGAAGGAATACTAAGAGTCAACATAGAACCAA
TAAACGTAACCTAGAACCTTTTTTATATGAGGCTTATCTGCTAACCATTGCGCCACTGTC
CCATCCAGGCTCCTACCAGATTCTGTTGTTCACCAGTAAAGAAAGGAGGGCAGAAATTTA
GACATCAAAGGCAAACTTTGATTTGGAACTGTCCTGCCTCCTTCCTGAAGGGCTTTTAAC
ACCCCTTTGAGAGTTGGCTTTACACAAATGTAAAAAAGAACCTTCTTTGACCCTAAAAAG
CATCACATTTTACTGCGGAGTGGGGGCTGAGAGTAGGAGGTGTGGGGTTTACACACAGCC
AGGCCTTAAGGTCTGCACCTCTCAGGTCTGTCTGCAGCTTTTAGTGCTGGCAGGTTCCTG
AGAAATGAGGCTGGGAGAGAGGGAGGAGAGGGGAAAATGCCATATTCCTGTCACTGGCGA
AATACATTCCCGGTGGGGGATAATGACAGCATCTCAGCAGGACAGGAAATGATTTGAT
```

```
>amplified_fugu_segment
GGGCAATATCAGAGCGAGACTTATCAAGGGTTTTTCTTCTTCTCAGGGGTCACTGAGGAG
CAACTGCATTTAAGTGGGCAATTCAATTGTGAGACATAGTTGGGGACGAGCTGCAGTGAG
TCTGATTAAAGTCGGTCCAGAGACCACAAAGATAAGCTTATAATGACAGCAAGAGAATGA
ACCTAATAACAAGGTATACTTTTGAATTTGTCGTGTTTTTTAACCTGACCCTGCCATGCT
TATTTTAAAGCTTATTTTCATTGTTAAACATTAATACTCTACTCATTCTAATAGTTTTAT
AGCAATTTATTTTAGTAACATCTATAGTGAAGATCCAGAAATATGGTAGAGCAGAGAGCA
AATGGACCAAAACACACATAAGACTAATTCAGGAATGAATAAATCTAGAGACTGAGGTCG
AACAGGAACATTTGAGGGC
```

```
>rCNE_mouse_sequence_2026043
TCCTTAACATTTTCATGGGTGACAAATTCAATTAAATATATTCATTACTCTAATGTTCGA
CTTGGCAGCTTTAGTCAGCATGNNNNNNNNNNNNNNNNNNNNNNNNNNGTTGTATGACCTT
TGTATTGATTGGGCTAAAATGGCTTTAGCCTGGGTGTGGAAGATGGGGCAGTTGGGAGGG
AAGACAGAAAAGCACACAAATACACACACTCTTATGTTTTCTCTTCCAATTACTGAGGGA
GATGATAAAACCACAGCAAATGATGTATCAGCTCATTTTATCTTATCGCTTTGGGGCTAG
GTGACAATGAAGTTCATATTTCACTGGCTCCCATTGAAGATTGTGAAAATTACTTGCCCT
CATGACCTGAAATTTCACTAGAAGACAGAAATTTCTCTTCTGCAAAGTAATGCAATTTTT
CAGATGTCAACCTTTAGCCCAGCTTTAAAAATTAATTTCATTTATATTAACCAATGATAT
TGATATTTTCACAGAACGCTCTTTGATACATGAAGCACTGACTGGCAAATTGATGTTGCT
CTAGATGGGCAAAACTATTTTACTTTAAGCTGCGATGGCAGGCAGATTTATGGCAAACTG
AAATTCCCATTTCATAACTTAATATTTGTGCTGGAGCAATGCACCTTTAAGCCTTTGGCC
TGTAAATAATTCGGACTATTTACTTGGTTCTATTTTATTTTCAACATTCCTCAGCTTAAA
AAATAATTTACATTCTACAGGATATATTATAAAATCCAGNNNNNNNNNNAACTAGTAGTTT
TTATATAGCGTAATGAG
```

```
>SCE_mouse_sequence_2026320
ATTTTATTTTCAACATTCCTCAGCTTAAAAAATAATTTACATTCTACAGGATATATTAT
```

FUGU HITS IN THE CONTEST OF MAMMALIAN rCNE

CLUSTAL W (1.83) multiple sequence alignment

```
ENSMUSG00000057777      TCCTTAACATTTTCATGGGTGACAAATTCAATTAAATATATTCATTACTCTAATGTTCGA
ENSRNOG00000031398      TCCTTAACATTTTCATGGATGACAAATTCAATTAAATATATTCATTACTCTAATGTTCTA
ENSCAFG00000007960      TCCTTAGCATTTTCATGGGTGACAAATTCAATTAAACATATTCATTACTCTAATGTTCTA
ENSG00000181541         ---ATAGCATTT-CATGGGTGACAAATTCAATTAAACATATTCATTACTCTAATGTTCTA
SINFRUG00000122928      ------------------------------------------------------------

ENSMUSG00000057777      CTTGGCAGCTTTAGTCAGCATGNNNNNNNNNNNNNNNNNNNNNNNNN-GTTGTATGACCT
ENSRNOG00000031398      CTTGGCAGCTTTAGTCAGCATGNNNNNNNNNNNNNNNNNNNNNNNNNGTGCTGTATGACCT
ENSCAFG00000007960      CTTGGCAG-TGTAGTCAGTATGNNNNNNNNNNNNNNNNNNNNNNNNNNNNGCTACGTGACCT
ENSG00000181541         CTTGGCAGTTTAGTCAGTATGNNNNNNNNNNNNNNNNNNNNNNNNNNNNGCTATGTGACCT
SINFRUG00000122928      --------------TCAG-GTTAAAAAACACGACAAATTCAAAAGTATACCTTGT-----
                                      ****  *                                 *

ENSMUSG00000057777      TTGTATTGATTGGGCTAAAATGGCTTTAGCCTGGGTGTGGAAGATGGGGCAGTTGGGAGG
ENSRNOG00000031398      TTGTATTGATTGGGCTAAAATGGCTTTAGCCTGGGTGTGGATGATGGGGCAGTTGGGAGG
ENSCAFG00000007960      CTGTATTGATTGGGCTAAAATGGTTTTAGCCTGGGTGTGGATGATGGGGCAGCTGGGAGG
ENSG00000181541         TTGTATTGATTGGACTAAAATGGCTTTAGCCTGGGTGTGGACGATGGGGCAGTTGGGAGG
SINFRUG00000122928      ------------------------------------------------------------

ENSMUSG00000057777      GAAGACAGAAAAGCACACAAATACACACACTCTTATGTTTTCTCTTCCAATTACTGAGGG
ENSRNOG00000031398      GGAGAGAGAAAAGCATACAAACATACACACTCTTATGTTTTCTCTTCCAATTACTGAGGG
ENSCAFG00000007960      GGCAAGAGGGGAGCACACAAACACATACACTCTTATGTTTTCTCTTCCAATTACTGAGGG
ENSG00000181541         GGCAAGAGGGGAGCCTACAAACACACACACTCTTATGTTTTCTCTTCCAATTACTGAGGG
SINFRUG00000122928      ------------------------------------------------------------

ENSMUSG00000057777      AGATGATAAAACCACAGCAAATGATGTATCAGCTCATTTTATCTTATCGCTTTGGGGCTA
ENSRNOG00000031398      AGATGATAAAACCCCAGCAAATGATGTATCAGCTCATTTTATCTTATCGCTTTGGGGCTA
ENSCAFG00000007960      AGATGATAAAACCACAGCAAATGATGTATCAGCTCATTTTATCTTATCGCTTTGGGGCTA
ENSG00000181541         AGATGATAAAACCACAGCAAATGATGTATCAGCTCATTTTATCTTATCGCTTTGGGGCTA
SINFRUG00000122928      ------------------------------------------------------------

ENSMUSG00000057777      GGTGACAATGAAGTTCATATTTCACTGGCTCCCATTGAAGATTGTGAAAATTACTTGCCC
ENSRNOG00000031398      GGTGACAATGAAGTTCATATTTCACTGGCTCCCATTGAAGATTGTGAAAATTACTTGCCC
ENSCAFG00000007960      GGTGACAATGAAGTTCATATTTCACTGGCTCCCATTGAAGATTGTGAAAATTACTTGCCC
ENSG00000181541         GGTGACAATGAAGTTCATATTTCACGGGCTCCCATTGAAGACTGTGAAAATTACTTGCCC
SINFRUG00000122928      ------------------------------------------------------------

ENSMUSG00000057777      TCATGACCTGAAATTTCACTAGAAGACAGAAATTTCTCTTCTGCAAAGTAATGCAATTTT
ENSRNOG00000031398      TCATGACCTGAAATTTCACTAGAAGACAGAAATTTCTCTTCTGCAAAGTAATGCAATTTT
ENSCAFG00000007960      TCATGACCTGAAATTTCACTAGAAGACAGAAATTTCTCTTCTGCAAAGTAATGCAATTTT
ENSG00000181541         TTATGACCTGAAATTTCACTAGAAGACAGAAATTTCTCTTCTGCAAAGTAATGCAATTTT
SINFRUG00000122928      ------------------------------------------------------------

ENSMUSG00000057777      TCAGATGTCAACCTTTAGCCCAGCTTTAAAAATTAATTTCATTTATATTAACCAATGATA
ENSRNOG00000031398      TCAGATGTCAACCTTTAGCCCAGCTTTAAAAATTAATTTCATTTATATTAACCAATGATA
ENSCAFG00000007960      TCAGATGTCAACCTTTAGCCCAGCTTTAAAAATTAATTTCATTTATATTAACCAATGATA
ENSG00000181541         TCAGATGTCAACCTTTAGCCCAGCTTTAAAAATTAATTTCATTTATATTAACCAATGATA
SINFRUG00000122928      ------------------------------------------------------------

ENSMUSG00000057777      TTGATATTTTCACAGAACGCTCTTTGATACATGAAGCACTGACTGGCAAATTGATGTTGC
ENSRNOG00000031398      TTGATATTTTCACAGAACACTCTTTGATACATGAAGCACTGACTGGCAAATTGATGTTGC
ENSCAFG00000007960      TTGATATTTTCACAGCACGCCCTTTGATACATGAAGCACTGACTGGCAAATTGATGTTGC
ENSG00000181541         TTGATATTTTCACAGAACACTCTTTGATACATGAAGCACTGACTGGCAAATTGATGTTGC
SINFRUG00000122928      ------------------------------------------------------------

ENSMUSG00000057777      TCTAGATGGGCAAAACTATTTTACTTTAAGCTGCGATGGCAGGCAGATTTATGGCAAACT
ENSRNOG00000031398      TTTAGATGGGCAAAACTACTTTACTTTAAGCTGCGATGGCAGGCAGATTTATGGCAAACT
ENSCAFG00000007960      TTTAGATGGGTAAAACTATTTTATTTTAAGCTGCTATGGCAGACAGATTTATAGCAAACT
ENSG00000181541         TTTAGATGGGTAAAACTATTTTATTTTAAGCTGCTGTGGCACACAGATTTATGGCAAACT
SINFRUG00000122928      ------------------------------------------------------------

ENSMUSG00000057777      GAAATTCCCATTTCATAACTTAATATTTGTGCTGGAGC-AATGCACCTTTAAGCCTTTGG
ENSRNOG00000031398      GAAATTCCCATTTCATAACTTAATATTTGTGCTGGAGA-AATGCACCTTTAAGCCTTTGG
ENSCAFG00000007960      GAAATTCCCATTTCATAATTTAATATTTGTGCTAGAGT-GATGCACTTTTAAGCCCCTGG
ENSG00000181541         GAAATTCCCATTTCATAATTTAATATTTGTGCTGGAGTTGATGCACCTTTAAGCCCTTGG
SINFRUG00000122928      ------------------------------------------------------------

ENSMUSG00000057777      CCTGTAAATAATTCGGACTATTTACTTGGTTCTATTTTATTTTCAACATTCCTCAGCTTA
ENSRNOG00000031398      CCTGTAAATAATTCAGACTCTTTACTTGGTTCTATTTTATTTTCAACATTCCTCAGCTTA
ENSCAFG00000007960      CCTGTAAATAATTCAGACTCTTTAGTTGGTTCTGTTTTATTTTCAATATAATTCAGCTTA
ENSG00000181541         CCTGTAAATAATTCAGACTCTTTACTTGGT--TGTTTTATTTTCAATATTCCTCAGCTTA
SINFRUG00000122928      ------------------------------------------------------------
```

```
ENSMUSG00000057777      AAAAATAATTTACATTCTACAGGATATATTATAAAATCCAGNNNNNNNNNAACTAGTAGT
ENSRNOG00000031398      AAAA-TAATTTATATTCTATATGATACATTATAAAATC---CTGATTTTTAACTGGAAGT
ENSCAFG00000007960      AAAAATAATTTGCATTCTAAATGATGTATCATAAAAGC----CATCTTTTATTGAACATT
ENSG00000181541         AAAA-TAATTTGCATTCTAAATGATGCATCTTAAAAGCCATCTCTTATTCAACTAGCATT
SINFRUG00000122928      ------------------------------------------------------------

ENSMUSG00000057777      TTTTATATAGCGTAATGAG
ENSRNOG00000031398      TACTATATAGCTTAATGAG
ENSCAFG00000007960      TAATATGTATCTTAATGAG
ENSG00000181541         AAATATCAATCTTAATGAG
SINFRUG00000122928      -------------------
```

Fragment Shh_12058

Representation of conserved regions within their genic context

Within the mouse context is shown the rCNE from which the tested SCE is derived (red diamond)

Within the fugu context all CHAOS hits obtained from the mouse rCNE are shown (green diamonds, in red the one tested)

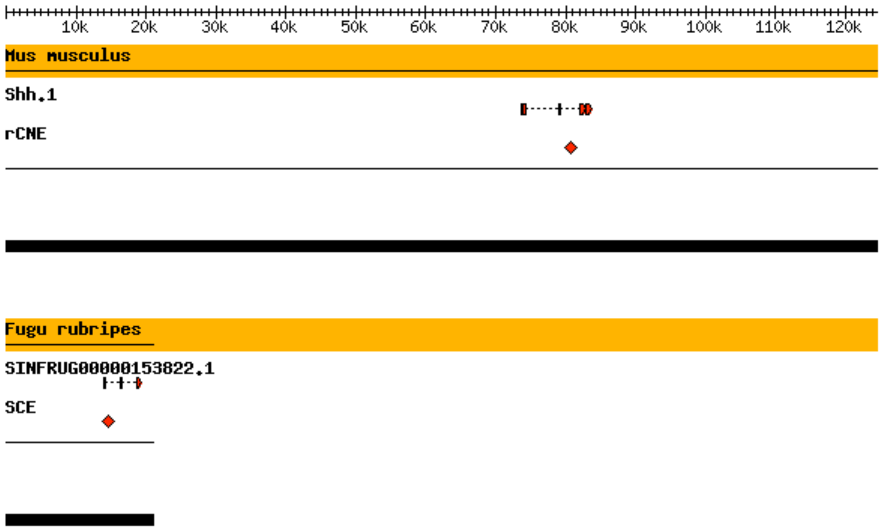

Conserved regions shown within 2000bp context

The mouse context contains:

-rCNEs green boxes, in red the one considered for this test

-SCEs yellow boxes, in red the one considered for this test

-CHAOS hits blue boxes, in red the one considered for this test

The fugu context contains:

-the fragment amplified in this test

-CHAOS hits blue boxes, in red the one considered for this test

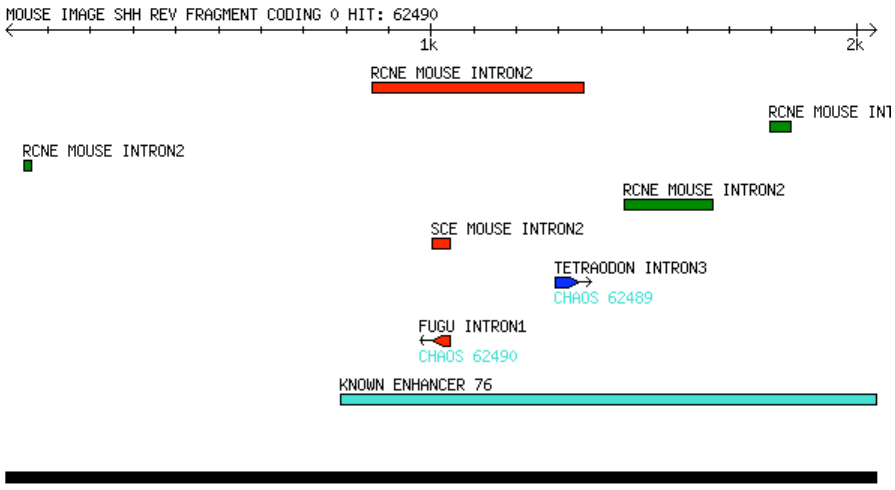

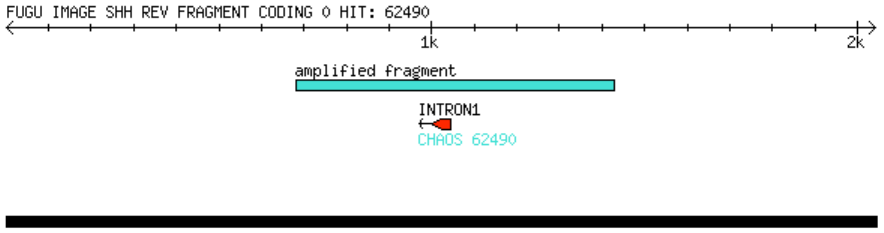

SEQUENCES

```
>fugu_fragment_for_primer_design_12058
TGTCTGCCAAGATAAACTCACCTCGCCTATTTATGCCCCAATCTCCGCCAACACTCGGGA
CAGGAGCGCGTCGGCGGCTCAGGGGACAGGTTCACAGGAGCGGCGCACTGGCCACTTTTA
CGCGCCGGGAATGGCCGCTTTTTGGGACTCCGGACTGAGTCCTGCTCCGTCAGCCAGACT
TTAAAATTCCCCAGGAGACACCCACCCACGTCAAAAGAAATCCAATTAGGGGGTTACAAG
CGGTCCCGGGACGGAATGXXXXXXXXXXXXXXXXXXXXXXXXXXXXXXXXXXXXXXXXXX
XXXXXXXXXXXXXXXXXXXXXXXXXXXXXXXXXXXXXXXXXXXXXXXXXXXXXXXXXXXX
XXXXXXXXXXXXXXXXXXXXXXXXXXXXXXXXXXXXXXXXXXXXXXXXXXXXXXXXXXXX
XXXXXXXXXXXXXXXXXXXXXXXXXXXXXXXXXXXXXXXXXXXXXXXXXXXXXXXXXXXX
XXXXXXXXXXXXXXXXXXXXXXXXXXXXXXXXXXXXXXXXXXXXXXXXXXXXXXXXXXXX
XXXXXXXXXXXXXXXXXTAACCCGCTCGGCTACCGCTGTGCCTTTCAGCCTGGCTTTTCC
CCGCAATCAAACCTGCGCGTTATTGGCGCACTTTGGCCAGACCCGAAACACGTGGACGCG
GTTATCTTCTCTGCCTTTCGTGTCGACTCGAGGTGAAGAAATTACCAAACAACTCGGGCA
TTTTCCGGTGATTTCTGAGAACGCTGCTTCAGCAGCTCGCGCTCCCAGCGCCCCGCTGAC
CTCTGCGGGCTTCCAAGCTTTTCTCGAGAAGTNNNNNNNNNNNTTCTACTTCGGAGAAGT
GGAGGAATTCGCAGCTGATAACTGCTGTTTTGCACAATCCGCTCCTCAGTCCGGGCTCGG
AGCCTCGCGGCGTGTTGACACAGCGCGGTGAGGTGACCGGCGTGAATCAGAGCTCCGTGT
GAACCCTCAGAGGTCCACTGTGCGACCCCCGTCTCAAAGGGAAGGCTTTTAATTAAAACA
CTATGTCCTCTGTCTCGCGGCGTGTTTTTGGTGGAAAAAACAGCCATTTGGTCAGAGCCC
GGGTCACGCGTGTGCGCATGTTTACCGTTGTCGTTTTTAGCCCGACTGGCCAGACTTAAC
AGTCGCCTTTTGGACTGTCAAAAATTTTTACGACACCGTCATTGATGTGGCCGCGGGTGG
AATTTATTTGACTCCTCGTTTCCCCAGGAATAAAGTATTTATATCTGCCTCTTGTGACCG
CGGGCTTCCTCTCCGAGCCGCGGCGCTTGCGTCCAGAGACCCACGTGGGGGGAGCGAAAG
CGCGCCCTCGCCGTCTGGAGCTGTCACCTCGGTGCGCGCACTGCAAAAAGTCAACCCGGT
TTACCTGCGCAGAGACGCCAAAATGGGAGCTGAGACCTCGATTCAAGCGCCACAACATCC
GAAGCCACCTTCGTTTGTTGATGTGGCGCACGGAAACGCACGACGGCGCGAGGGAAATGA
GCAATTGAAAGCAAGGAGACACAGAAACACGTGTTTTCCAACACTTTTTACAGCAAGCCG
CGCACAATTTACGAGCTCCGGCCCGCAGGAGACAGCCCTCCGTTTGTCTTTTACTGTTCC
GTGTGATTTTTGCGTTTAAAATAGCGGCTCCGCTCGCGCCGAGGCTTTAATTACAGACAC
CGCATCTTTTCACCTCTTTTCCTCACTTTTTTCCTCAACTTTTGGACGTTTCGAGGCGCT
GCGCGCCGCACACGGGCGCGCCTGTTTCCACACAGTGTTAAAATTCATTCTCTCGAGCTC
GAGCTTCAACATTTATTTCCAGTTTTAACTCATTTTATTTGTCATTTTGGGTCGATCAAG
ACTATTAAAGAGTCCGGACCATTTCTTCTGCAGCGCAGAGGTGCGCACAGACGCACGGAG
TCAAATGTTTACTCAGCCAGGGGTTTGTATAGTTCAACCCANNNNNNNNNNNAGAAACCC
```

```
CCGTCGATCCGCCCTGCACCTTGAAAGGAAAACCAACAGAATCTATCGTCTGATTTATGG
CGT
```

```
>amplified_fugu_segment
GTGTCGACTCGAGGTGAAGAAATTACCAAACAACTCGGGCATTTTCCGGTGATTTCTGAG
AACGCTGCTTCAGCAGCTCGCGCTCCCAGCGCCCCGCTGACCTCTGCGGGCTTCCAAGCT
TTTCTCGAGAAGTNNNNNNNNNNNNTTCTACTTCGGAGAAGTGGAGGAATTCGCAGCTGAT
AACTGCTGTTTTGCACAATCCGCTCCTCAGTCCGGGCTCGGAGCCTCGCGGCGTGTTGAC
ACAGCGCGGTGAGGTGACCGGCGTGAATCAGAGCTCCGTGTGAACCCTCAGAGGTCCACT
GTGCGACCCCCGTCTCAAAGGGAAGGCTTTTAATTAAAACACTATGTCCTCTGTCTCGCG
GCGTGTTTTTGGTGGAAAAAACAGCCATTTGGTCAGAGCCCGGGTCACGCGTGTGCGCAT
GTTTACCGTTGTCGTTTTTAGCCCGACTGGCCAGACTTAACAGTCGCCTTTTGGACTGTC
AAAAATTTTTACGACACCGTCATTGATGTGGCCGCGGGTGGAATTTATTTGACTCCTCGT
TTCCCCAGGAATAAAGTATTTATATCTGCCTCTTGTGACCGCGGGCTTCCTCTCCGAGCC
GCGGCGCTTGCGTCCAGAGACCCACGTGGGGGGAGCGAAAGCGCGCCCTCGCCGTCTGGA
GCTGTCACCTCGGTGCGCGCACTGCAAAAAGTCAACCCGGTTTACCTGCGCAGAGACGCC
AAAATGGGAGCTGAGACCTCGATTCAAGCG
```

```
>rCNE_mouse_sequence_60350
GCTCATTGGCATTCTACACACGCGGGCTGGGGCGCCGCGCCTTGTGTGTCAAGCGGGGTC
GTGCGACTCGACGACTCCGGCTCGCCAGAGCCCCGGGTCGCATTCCGGGGGGCTACGGAG
TGCTTCCAACGGCCAGCCCCACACTTCATGCCGGGGAAAACGATGAGAAGATTAAAAGCC
TTCTGTAATTCCAGCAGAAAGATTCTTTCTGGCAATCTCTATTTGCCAAAAGCATGATCC
TGGAGATTGGAATGCAAAGAAGACTAGGACCTCCCCACCCCTCCTCCCTGGCCCCCTGCG
CTCCGCCCCATCCTCAATTATTGTCCTCCCGGACAGTGAGCCTTGGAGAGCTACGGAGGG
CTGAGTAAAGCGGGTAGTCAAGGGGCCTTGAGAACCCGCGCTTCCAGCGCTCAGAGCAGG
CCTGGCCATTTAAAATTCAAATACACATCTCGAGTGCTTGGAAAAGAGGCCGGGCTGTGC
AAATAGTGCCTTGTGA
```

```
>SCE_mouse_sequence_60386
CACACTTCATGCCGGGGAAAACGATGAGAAGATTAAAAGCCTTCT
```

FUGU HITS IN THE CONTEST OF MAMMALIAN rCNE

CLUSTAL W (1.83) multiple sequence alignment

```
ENSCAFG00000005102      GCTCATTGGCATTCGGCGCACACCGGCCGGGGCGCGGTGCGCGGCGTGTCAAGCGGGGTC
ENSG00000164690         GCTCATTGGCATTCTGAGCACACGGGCGGGGGCGCGGGGCGCAGCGTGTCAAGCCGGGCC
ENSMUSG00000002633      GCTCATTGGCATTCTACACACGCGGGCTGGGGCGCCGCGCCTTGTGTGTCAAGCGGGGTC
ENSRNOG00000006120      GCTCATTGGCATTCTACACACGCGGGCTGGGGCGCCGCGCCTTGTGTGTCAAGCGGGGTC
SINFRUG00000153822      ------------------------------------------------------------


ENSCAFG00000005102      GTGCGACAGGACGACT---AGGGCTCGCCTGCGCCCCGGGCCGCATTCCGGGGGGCGACG
ENSG00000164690         GTGCGACTCGACGACT---CGGGCTCGCCAGCGCCCGGGGTCGCATTCCGGGGGGCTACG
ENSMUSG00000002633      GTGCGACTCGACGACT---CCGGCTCGCCAGAGCCCCGGGTCGCATTCCGGGGGGCTACG
ENSRNOG00000006120      GTGCGACTCGACGACTACTCCGGCTCGCTAGAGCCCCGGGTCGCATTCCGGGGAGCTACC
SINFRUG00000153822      ------------------------------------------------------------


ENSCAFG00000005102      GCGGGCCTCCAACGGCCAAGCCCGCACTTCATGCCGCAGAAACCGATGAGAAGATTAAAA
ENSG00000164690         GAGGGCCTCCAACGGCCAGCCCCGCACTTCATGCCAGAGAAACCGATGAGAAGATTAAAA
ENSMUSG00000002633      GAGTGCTTCCAACGGCCAGCCCCACACTTCATGCCGGGGAAAACGATGAGAAGATTAAAA
ENSRNOG00000006120      GAGTGCTTCCAATGGCCAGCCCCGCACTTCATGCCAGGGAAAACGATGAGAAGATTAAAA
SINFRUG00000153822      ----------------------CACGCCGCGAGACAGAGGACATAGTGTTTTAATTAAAA
                                              * * *  *  * *   * *     **     *******

ENSCAFG00000005102      ACCCTCTGTAATTCCAGCAGGAAGATTCTTTCTCGCAATCTCTATTTGCGAAAAGCATGA
ENSG00000164690         GCCCCCTGTAATTCCAGCAGGAAGATTCTTTCTGGCAATCTCTATTTGCAAAAAGCATGA
ENSMUSG00000002633      GCCTTCTGTAATTCCAGCAGAAAGATTCTTTCTGGCAATCTCTATTTGCCAAAAGCATGA
ENSRNOG00000006120      GCCTTCTGTAATTCCAGCAGGAAGATTCTTTCTGGCAATCTCTATTTGCCAAAAGCATGA
SINFRUG00000153822      GCCTTCC-----------------------------------------------------
                         **  *

ENSCAFG00000005102      TCCCGGAGATTGGAATGCAAAGAAGACCGGGCTGTCCCCGCCCCTCCTCCGCGGCCCCCT
```

```
ENSG00000164690         TCCCGGAGATTGGAATGCAAAGAAGAC--GGCCCTCCCCGCCC-TCCTCCCCGGCCCCCT
ENSMUSG00000002633      TCCTGGAGATTGGAATGCAAAGAAGACTAGGACCTCCCCACCCCTCCTCCCTGGCCCCCT
ENSRNOG00000006120      TCCTGGAGATTGGAATGCAAAGA-GACTGGGACCTCCCC---CCGCCTCCCTGGCCCCCC
SINFRUG00000153822      ------------------------------------------------------------

ENSCAFG00000005102      GCTCGCCACCCCGGCTCCGATCATTGTCCTC-GGGACAGTGAGCCTCAGAGAGCGACAGA
ENSG00000164690         GCGCTCCGCCCCAACTTCAATTATTGTCCTG-GGGACAGTGAGCCTCAGAGAGCGACAGA
ENSMUSG00000002633      GCGCTCCGCCCCATCCTCAATTATTGTCCTCCCGGACAGTGAGCCTTGGAGAGCTACGGA
ENSRNOG00000006120      GCGCTCCGCCCTATCCTTAATTATTGTCCTCTGGGACAGTGAACCTCCGAGAGCTACGGA
SINFRUG00000153822      ------------------------------------------------------------

ENSCAFG00000005102      GGGCTCGGGAAAGCGGGTAGTCAAGGGGCCTTGAGAACGCAGCAGCTTCCAGCGCCTGGA
ENSG00000164690         GGGCTCGAGAAAGCGGGTAGTCAAGGGGCCTTGAGA-CCCGGC-GCTTCCAGCGCTCCGA
ENSMUSG00000002633      GGGCTGAGTAAAGCGGGTAGTCAAGGGGCCTTGAGAACCC-GC-GCTTCCAGCGCTCAGA
ENSRNOG00000006120      GGGCTGGGTAAAGCGGGTAGTCAAGGGGCCTTGAGAACCC-GC-GCTTCCAGCGCTCCTA
SINFRUG00000153822      ------------------------------------------------------------

ENSCAFG00000005102      GCAGGCCCCGCCATTTAAAATTCAAATACACATCTTGAGTGCTTGGAAGAG--GCCTGGC
ENSG00000164690         ACAGGCCCCGCCATTTAAAATTCAAATACACATCTTGAGTGCTTGGAAGAGAGGCCTGGC
ENSMUSG00000002633      GCAGGCCTGGCCATTTAAAATTCAAATACACATCTCGAGTGCTTGGAAAAGAGGCCGGGC
ENSRNOG00000006120      GCAGGCCTGGCCATTTAAAATTCAAATACACATCTCGAGTGCTTGGAAAAGAGGCCGGGC
SINFRUG00000153822      ------------------------------------------------------------

ENSCAFG00000005102      TGTGCAAATAGTGC-TTGTGA
ENSG00000164690         TGTGCAAATAGTGC-T-----
ENSMUSG00000002633      TGTGCAAATAGTGCCTTGTGA
ENSRNOG00000006120      TGTGCAAATAGTGCCTTGTGA
SINFRUG00000153822      ---------------------
```

Fragment 3110004L20Rik_5803

Representation of conserved regions within their genic context

Within the mouse context is shown the rCNE from which the tested SCE is derived (red diamond)

Within the fugu context all CHAOS hits obtained from the mouse rCNE are shown (green diamonds, in red the one tested)

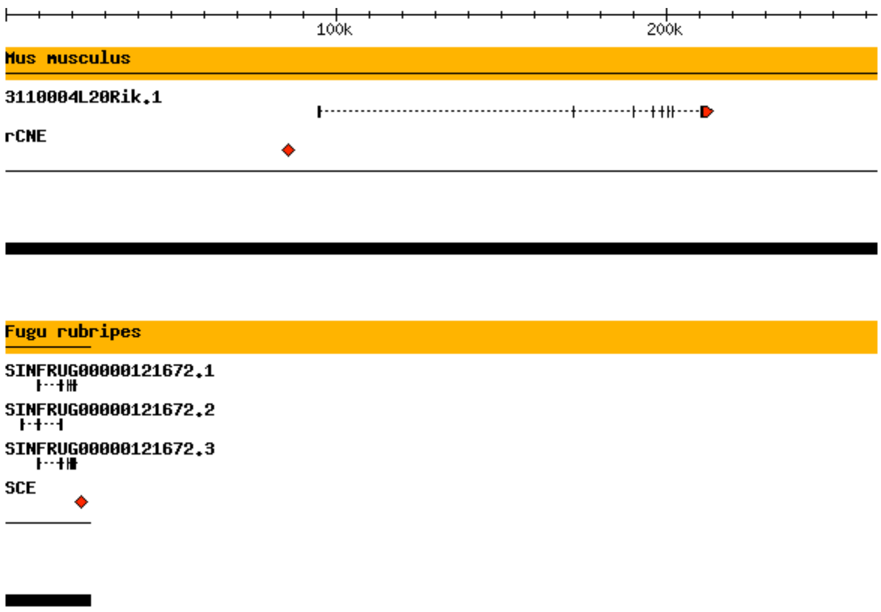

Conserved regions shown within 2000bp context

The mouse context contains:

-rCNEs green boxes, in red the one considered for this test

-SCEs yellow boxes, in red the one considered for this test

-CHAOS hits blue boxes, in red the one considered for this test

The fugu context contains:

-the fragment amplified in this test

-CHAOS hits blue boxes, in red the one considered for this test

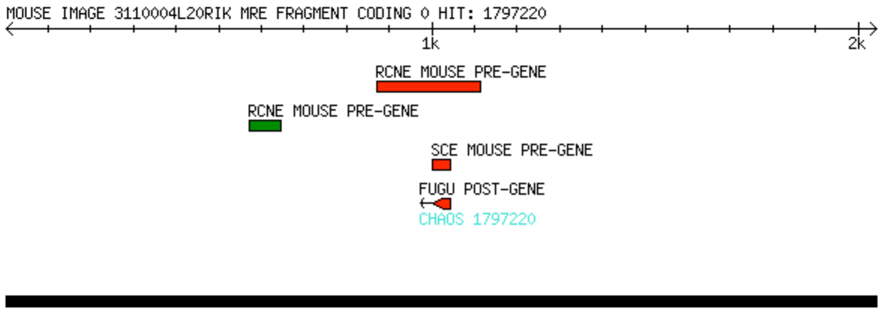

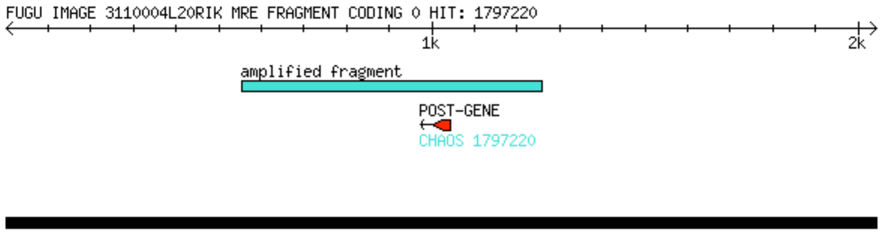

SEQUENCES

```
>fugu_fragment_for_primer_design_5803
CCACCCCAGCAGCCCTCCTGATCTCCACCACCCCCACCATCGAACCCCGGCCCAGCTCCG
TGTTAGAATCCCCCGACCCAGTAGAAACTCGCAGCGTCTCCCCAGAATCTCCCCAGTCTT
TGCAGGAGGACCTTCAAGCCAGTTTAGCAGAACCTGGTGCTCTATCTATGCAGGACAGAC
CTNNNNNNNNACCCACTCCTCCTCCAGTACCAGGTACTGACCATTCCATAGAGCCATCAG
CCAACTCTTGCCCCCCCACCGAGTCTAACGTGGACCAGTCTCCACCTGGTCCCACTACAC
ACTCCCCCTTACTCCCTGTCACAGACTCCCCCATCACCCCAAAATTAGACTCAGCCCCTC
CCTGTATTAACAGCAGANNNNNNNNNNNNNNNNNNNNNNGGCCAAAAGTCCATTCTTGACG
CTTTGGGTCCTGTTGTCATAGACACTGACGGTACTGCAGCACAAAGTCCCACAATCCTCC
CAGTGACACAAATCGTGCGCCCCGCCACTCCAGAACCCCCCACAGCCGTTGCCCCGCCAG
CACAGGGTCCGGTTGCCATTGATTCCCTCCAGACTCTTATAGACTGCACTGTCTCCTCGC
CCATAGACAGGGACACCCTCTCCTTCACGGACGGTGCCAGCCCTGACAACAACACCCCCA
ACGGTGCCGCGTCGTCGTGAATCAGTGTTGGCAGCCGGCGCTCGTGGTCACCGGGGGGGT
CGCTCGCGCCCGATGGAACCGTGAGAGGTGGGGGTGGGCGGAGGGGGTCCTGCTTTGACC
CGAGGCGCCGTCCCCTGGTCTTCAGGAGCTGCTTACGTCTCGGTGTGCCCTCTTCCTGCA
TTTAGCCAATGAATACGATCAAGTGAGCGAGTGCCAGAGGCTGGTCATGTGACAGTGTCT
CCCGCTCGCCGTGAATGTCACTTGAAATCACCAGCCTATAAACTACTTTGGTGTAAATGA
ATGGGAATTTGTCCTTGTGAAGAGGCAGCGCTGCCCCCGCAGGAGCGTCGGGGGAAAACA
TGCAATCGAGGGGGGAAAAAAATAATAATAATCCACGGTACTTTTTGCCGAAAATGAAGT
GTCTGTCTCGTGTGTTTGCTAGGNNNNNNNNNNNNNTGCCTTACCCGTACGAACAAAACAC
CTGAATGTTACAGCGTATATTTCATTTCACGATTACAGGAAACGGGCCGAGGTCAACGCA
CCAAAACCACATTTCTGAGTAAAGTCAAAGAGAAAAAGTGCCAAAATTCCCTTCCAAAAT
CAAACAAAAGAATCATGGGTGAAAAGCCAATATTCACGAATTCAATTTACAGAGCGCGGA
AGCATTTCTTTGTAAATGTGATTTGTAAGTAAAAGCTTAACTTGTTGTGGAGCTCGANNN
NNNNNNNNNNNNNNNNNNNNNNNNNNNNNNNAAGGATCCAGCGGCCCCACCGTCCTGTAAAA
ATCGTAACCTTCAGTGTTGGCGGTCGCTCCACGTGGTCTTGGGTGGGCGACGGAGCATGA
AAATCAGGGTCTGAGGGTGGCAAAGCAAACGTGTCTCATGCCGCCACGTGCACGTACGTT
TTACAGCAGCTCCATAATTTGTCTATAGGAACTCATGCGCGATTAGCATCACCTACCTGT
TTACAATAATAATTATCTGTTACTGTAAAACTCTAGAGCCAACTGAATGATCCTCATTTC
ACATGTGACGGGGTCACATGACATTTGTTCAGGAAGTGTTTCCGGTCTACCGCGGCGTCG
CCGTGACTCATCATTTTCCTCTTGTATCCTGGTCGTGTGTGTCCCGACACACACGACCTG
CCTGTGAATGTTGTTGNNNNNNNNNNNNNNNNNNNNNNNNNNNNNNNNNNNNNNNNNNNNN
NNNNNNNNNNNNNACAGCACGACGGGGCGGTTTTGGTGGATTTTTCCCTGTGGAGAGTTG
AAATCTCGCACACGCCGTGCTGCTAAGCCTCAAAGCACAATGCCCTGCAATCATGTGACG
TCATATCTGGTGGAGTTCAGAATGGTGACAGTNNNNNNNNNNNNNGGACAGTGGGAAAGA
ATC
```

```
>amplified_fugu_segment
GCCATTGATTCCCTCCAGACTCTTATAGACTGCACTGTCTCCTCGCCCATAGACAGGGAC
ACCCTCTCCTTCACGGACGGTGCCAGCCCTGACAACAACACCCCCAACGGTGCCGCGTCG
TCGTGAATCAGTGTTGGCAGCCGGCGCTCGTGGTCACCGGGGGGGTCGCTCGCGCCCGAT
GGAACCGTGAGAGGTGGGGGTGGGCGGAGGGGGTCCTGCTTTGACCCGAGGCGCCGTCCC
CTGGTCTTCAGGAGCTGCTTACGTCTCGGTGTGCCCTCTTCCTGCATTTAGCCAATGAAT
ACGATCAAGTGAGCGAGTGCCAGAGGCTGGTCATGTGACAGTGTCTCCCGCTCGCCGTGA
ATGTCACTTGAAATCACCAGCCTATAAACTACTTTGGTGTAAATGAATGGGAATTTGTCC
TTGTGAAGAGGCAGCGCTGCCCCCGCAGGAGCGTCGGGGGAAAACATGCAATCGAGGGGG
GAAAAAAATAATAATAATCCACGGTACTTTTTGCCGAAAATGAAGTGTCTGTCTCGTGTG
TTTGCTAGGNNNNNNNNNNNNNTGCCTTACCCGTACGAACAAAACACCTGAATGTTACAGC
GTATATTTCATTTCACGATTACAGGAAACGGGCCGAGGTCAACGCACCAAAACCACATTT
CTGAGTAAAGTCAAAGAGAAAAAGTGCCAAAATTCCCTTCCA
```

```
>rCNE_mouse_sequence_1573131
CATTGTTAATTGATAGGGGTCACTTTGAGGTGACTACTGAACAACTTATTAGGTGGCAAC
TAAATTGGGTCTGGCTGCCCTGGGCTGGGTGTAGTAATTGCTCAGGTGACTGAGTTGGCA
AAATTGCAGGCTATTTTTCTCCACTTCCCTGGATGTTTTTATTAAAGTCTCCTGTCTCCC
GGCCATCTAATATAAGTGTAATGTGCTCTTTTGCATCATTTAACCTTGAGTAAACTCGTC
AATGGG
```

```
>SCE_mouse_sequence_1573258
TATTTTTCTCCACTTCCCTGGATGTTTTTATTAAAGTCTCCTG
```

FUGU HITS IN THE CONTEST OF MAMMALIAN rCNE

CLUSTAL W (1.83) multiple sequence alignment

```
ENSG00000137266           -------------CACTGTTAATGGATAGGGCTGATCACTGAATGTCTTATTAGCTAGCA
ENSCAFG00000009344        CATTGTTAATTGATAGAAGTTGTGCATTGAGGTGACCGCAGGACGTCTTATTAGGTAGCA
ENSMUSG00000038267        CATTGTTAATTGATAGGGGTCAC--TTTGAGGTGACTACTGAACAACTTATTAGGTGGCA
ENSRNOG00000017210        CATTGTTAATTGATAGGGGTCAC--TTTGAGGTGACTCCCAAACAACTTATTAGGTGGTA
SINFRUG00000121672        ------------------------------------------------------------


ENSG00000137266           CCTTAATGGGGTCATGGCTGCCCAAGGCCAGGTCTGGTCATTG-CTCAGGTGGCTGAACT
ENSCAFG00000009344        ACTAAATTGAGTCGTGGCTGCCCAAGGTCAGGTATGGTTATCG-CTCACGTGGCTGAGTT
ENSMUSG00000038267        ACTAAATTGGGTC-TGGCTGCCCTGGGCTGGGTGTAGTAATTG-CTCAGGTGACTGAGTT
ENSRNOG00000017210        ACTAAATTGGGTC-TGGCTGCCCTGGGCTGGGTGTAGTAATTGGCTCAGGTGACTGAGTT
SINFRUG00000121672        ------------------------------------------------------------


ENSG00000137266           CACAACAGTATTGGCAATTTC-CTCCAGTCCCCTAAATGCTTT-ATTAAAATTTCTTCTG
ENSCAFG00000009344        CACAGCAGCATCAGCA-TTTC-CCCCGTCTCCCTGAATGCTTTTATTAAAATCTCTTTTG
ENSMUSG00000038267        GGCAAAATTGCAGGCTATTTTTCTCCACTTCCCTGGATGTTTTTATTAAAGTCTC--CTG
ENSRNOG00000017210        GGCCAAATTGCAGGCTATTTT-CTTCACTTCCCTGGATGTTTTTATTAAAGTCTC--CCG
SINFRUG00000121672        -------------TATTTTTTTCCCCCCTCGATTGCATGTTTTCCCCCGACGCTC--CTG
                                           ***  *  *       *  *** ***      *   **    *

ENSG00000137266           CCTCCTGGCCATCTCATAGAAGCATAATATACTCATTTGCATCCTTTAACCTCAAGTAAA
ENSCAFG00000009344        TCTCCTGGCCATCGAATCTCAGCATAATGTGCTCTTTTGCATCTCTTAACCTCAAATAAA
ENSMUSG00000038267        TCTCCCGGCCATCTAATATAAGTGTAATGTGCTCTTTTGCATCATTTAACCTTGAGTAAA
ENSRNOG00000017210        TCACCCGGCCATTTAATATAAGTGTAATGCACTCTTTTGCATCATTTAACCTTGAGTAAA
SINFRUG00000121672        ------------------------------------------------------------


ENSG00000137266           CTCATCAATGGG
ENSCAFG00000009344        CTCATCAATGGG
ENSMUSG00000038267        CTCGTCAATGGG
ENSRNOG00000017210        CTCATCAATGAG
SINFRUG00000121672        ------------
```

Fragment 3110004L20Rik_5802

Representation of conserved regions within their genic context

Within the mouse context is shown the rCNE from which the tested SCE is derived (red diamond)

Within the fugu context all CHAOS hits obtained from the mouse rCNE are shown (green diamonds, in red the one tested)

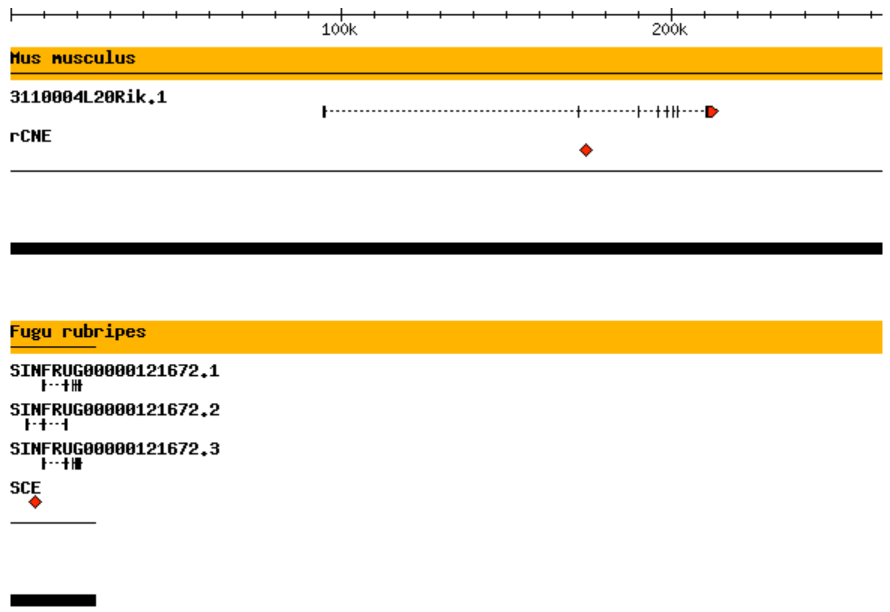

Conserved regions shown within 2000bp context

The mouse context contains:

-rCNEs green boxes, in red the one considered for this test

-SCEs yellow boxes, in red the one considered for this test

-CHAOS hits blue boxes, in red the one considered for this test

The fugu context contains:

-the fragment amplified in this test

-CHAOS hits blue boxes, in red the one considered for this test

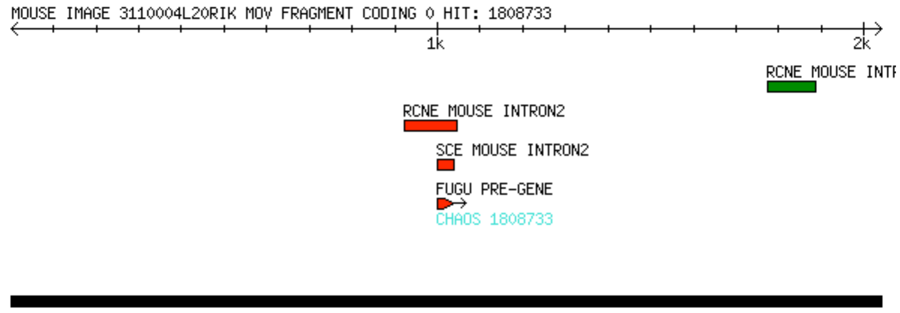

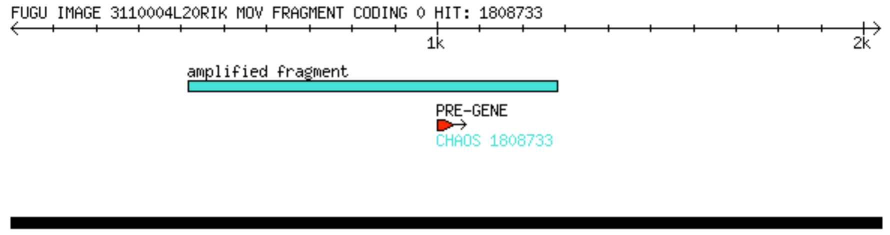

SEQUENCES

```
>fugu_fragment_for_primer_design_5802
TCCCATCTCCCAAGGTCCCTGACCAGAGATGGGTGGGACGTGAGGGATGACTCGGTGTTG
AGGACACTTCATAGCGTCCAGCGCTCTTCCGTCCCCTTTGTGACACATTATGTGACCATA
GTAACGCATTTAGTTGCTTCACCACCACCGAATCGGAGAAGACACGGGGACAGATCGCCG
GTGGGAGGTAGTCCAGGAGGTGAACCTAGAGTCCCAGGAGTCCTAGAAATGATTGTATAT
GATTACACTCAGCCCAGCTAGCTTCTCTTGTAAATGTGGCCGCAGATATTTGGGCACAAA
GTGTCTTTAAATGAGACTTTTTATGTCCCACCTTGGCCTCCATCTGCAGTGTTGAGGACT
CGGCCCTTTACCCCGTCCCCCACTGACGGGACAGGGTACAGATGTTGGACAGACTGTTTA
GCTCCAGCCCTGATGTCATACTTGAGAGTATTGTGTGAACCTGATAATGAGGAGCTCTTG
TATAATCGTGTTAGTGGGTGCAGGCAGGGCCCGTTATCACTGTTTTGGAGGTGGGACGCC
CATAGATCAGAAAGATGACACCGCCGCCGAGGCCTCGCTGCGCCGCGTCCCTTCATATTT
ACTCAGCTGGCTGGCCCCCGCCGCAGACCCCAGATCAGCGTGGGACGCTCTGAAATGATC
ACAGCTGTCTGAAGGTGACGGCCCCGCTGCAGATACCGAATCTACCCACAATGGGAGCCT
GCGCCGAAAAGGCGTGTGACCCAAAGATAAAGCGCCGCCGCGGCTCTACTGTGCGTCTGA
TAGGGGGGCTGTCTGGTCGCGGGGAGGGGGACGCAGTGCGGGGTCTCTAACCCTCGCCAC
GGGTTAAGGTTTGTTTGTTGGAGCTTGAGTTAAGCAGGAATGTGGCTGGGACAGGGTTGG
GGCGAGGGTCACAGCAGCCCCCTGCAGCGGCAGCTGTGCAGCCTCGGGCCGCAACGGTAA
ATGCAAAGAACCAGCTAATTATTTCTTTTGGAGCTGCTCAAATATTTGTCGTTGCCATTC
AGTGTTGCAGCAAGCTGCAGCGTGTGGACGCCAGTGTTTCAGAGGAAAGCCCTCGATGTT
TACGCAGCAGTAAATACGTTATTTCCACACCGAGCATCCCCACGCGTGTCACTATGTGAT
GCTAACCCTTTATCTGTGGCTGGGCTCAGCAAACTGGCGCTCTCTGCTGCGGCTGTCGTT
CTACACTGTTCCTCTCCAGTTGTTGGGTTTTTACAAATGTTGCTGGTGTTTCCCATGAAG
CCTGTCGAGGTTAGTGCTCTGTGGAGGGGGGGCGTTTGTCCTTTTGGGCCACCAAAGCTC
ATGTGGACTAATGGAAACGCGCTGGATTCCTCTCGTCATTTCCCCACCTCATTATTCTCC
ATCATCATCACCTGTAAACGACCACCGTGTAATGGCTTTAGCCAGGCAGCGGCGCCCACA
GGGTGGCGGAGGGAGAGTGTTGCTGAGCAGTAGCTCTGCCGCAGAGTCCTCGTGTGCACG
TTGNNNNNNNNNNNNNNACACACGTTTGGCTCATTTTGAAGCCGCAGACTAAATTCTAGA
TTCACTCTTCAATGTGGTGTTGTTCAGCCAGCCGCCGCTAGAGCCCCGCCTGCTCGGGAT
CGAGGAAGCACCGTTGAGCAACAAAATGTAACCACGGTTACTCGTGGATTCGTCTTTGCT
AATCTTCCTCCTCTCCGTCCTCCCCTCTGTGCTGCCTCTCTATCGNNNNNNNNNNNNNAC
CATCCCGGGCCGTGCAGTTCACCGAGTGCAGAAATGTGCAGCTGCAGTTGAGGGCGTCCT
CATCCGAGCTCGCCGCCGCGGCTGTGGGCCGGCCACCGGCGGCCATCGGGCTTGGGCTTG
GCGTGCACGCTGGGGATATGGACTGATTTGCTGCTTGGCTCTGAATTCTCCCAGCTAATG
AATCTGCAGCGCAAGCCGACTCTGCACCTGCTCCAAATGAGCGAGACAATGGGGGAGAAG
CTGCCGGGGCTAATGAGGCCGCGGCGGCACGGCCCCACAGATTTGACACGTTCGAGG
```

```
>amplified_fugu_segment
GTTTAGCTCCAGCCCTGATGTCATACTTGAGAGTATTGTGTGAACCTGATAATGAGGAGC
TCTTGTATAATCGTGTTAGTGGGTGCAGGCAGGGCCCGTTATCACTGTTTTGGAGGTGGG
ACGCCCATAGATCAGAAAGATGACACCGCCGCCGAGGCCTCGCTGCGCCGCGTCCCTTCA
TATTTACTCAGCTGGCTGGCCCCCGCCGCAGACCCCAGATCAGCGTGGGACGCTCTGAAA
TGATCACAGCTGTCTGAAGGTGACGGCCCCGCTGCAGATACCGAATCTACCCACAATGGG
AGCCTGCGCCGAAAAGGCGTGTGACCCAAAGATAAAGCGCCGCCGCGGCTCTACTGTGCG
TCTGATAGGGGGGCTGTCTGGTCGCGGGGAGGGGGACGCAGTGCGGGGTCTCTAACCCTC
GCCACGGGTTAAGGTTTGTTTGTTGGAGCTTGAGTTAAGCAGGAATGTGGCTGGGACAGG
GTTGGGGCGAGGGTCACAGCAGCCCCCTGCAGCGGCAGCTGTGCAGCCTCGGGCCGCAAC
GGTAAATGCAAAGAACCAGCTAATTATTTCTTTTGGAGCTGCTCAAATATTTGTCGTTGC
CATTCAGTGTTGCAGCAAGCTGCAGCGTGTGGACGCCAGTGTTTCAGAGGAAAGCCCTCG
ATGTTTACGCAGCAGTAAATACGTTATTTCCACACCGAGCATCCCCACGCGTGTCACTAT
GTGATGCTAACCCTTTATCTGTGGCTGGGCTCAGCAAACTGGCGCTCTCTGCTGCGGCTG
TCGTTCTACACTGTTCCTCTCCAGTTGTTGGGTTTTTACAAATGTTGCTGGTGTTTCCCA
TGAAGCCTGTCGAGGTTAGTGCTCT
```

```
>rCNE_mouse_sequence_1573175
GGTGAATGGTAGCACCGGCCAGCACAACCAGCTGTTGGTGGCAAAGTACAGAGAGTGACA
CATTTTCCTTAACAAGGAAGTATATACTGCCTGCATTCAGGGTGCCCAGCAAGATGCTTG
GT
```

```
>SCE_mouse_sequence_1573257
AAGTATATACTGCCTGCATTCAGGGTGCCCAGCAAGATGC
```

FUGU HITS IN THE CONTEST OF MAMMALIAN rCNE

CLUSTAL W (1.83) multiple sequence alignment

```
ENSRNOG00000017210      GGTGAATGAGAGCATCGGCCAGCACGACCAGCTGTTGGTGGCAAAGTACAGAGAGTGAC-
ENSG00000137266         GGTGAGCTGTAGCACCAGCCAGCATGACCAGCTGTTGGTAGCAACATATTGAAAGTTGCT
ENSMUSG00000038267      GGTGAATGGTAGCACCGGCCAGCACAACCAGCTGTTGGTGGCAAAGTACAGAGAGTGACA
SINFRUG00000121672      ------------------------------------------------------------

ENSRNOG00000017210      -ACATTTCTTTAACAAGAAGGTATTTACTGCCTGCACTTGGGGTGCCCAGCAGCATGCTT
ENSG00000137266         TGCTTTCCTTTAATGAGCAAGTATTTATTGCATGCACC-AGGGTGCCCAGCAGCGTACTT
ENSMUSG00000038267      CATTTTCCTT-AACAAGGAAGTATATACTGCCTGCATTCAGGGTGCCCAGCAAGATGCTT
SINFRUG00000121672      ------------------AAATATTTGTCGTTGCCATTCAGTGT-TGCAGCAAGCTGC--
                                          *  *** *   *    **    * **   *****   * *

ENSRNOG00000017210      GAT
ENSG00000137266         GGT
ENSMUSG00000038267      GGT
SINFRUG00000121672      ---
```

Fragment Elmo1_6026

Representation of conserved regions within their genic context

Within the mouse context is shown the rCNE from which the tested SCE is derived (red diamond)

Within the fugu context all CHAOS hits obtained from the mouse rCNE are shown (green diamonds, in red the one tested)

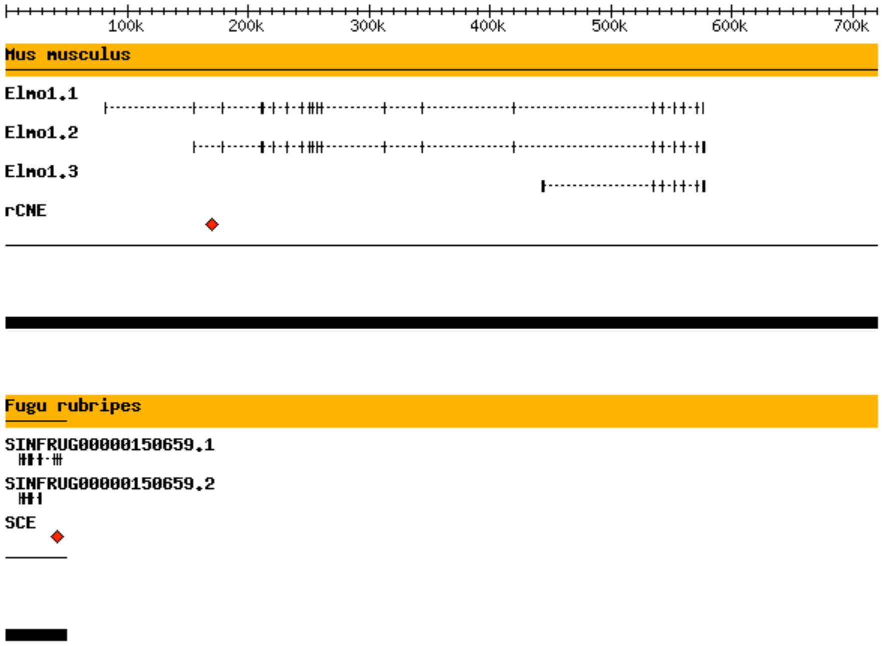

Conserved regions shown within 2000bp context

The mouse context contains:

-rCNEs green boxes, in red the one considered for this test

-SCEs yellow boxes, in red the one considered for this test

-CHAOS hits blue boxes, in red the one considered for this test

The fugu context contains:

-the fragment amplified in this test

-CHAOS hits blue boxes, in red the one considered for this test

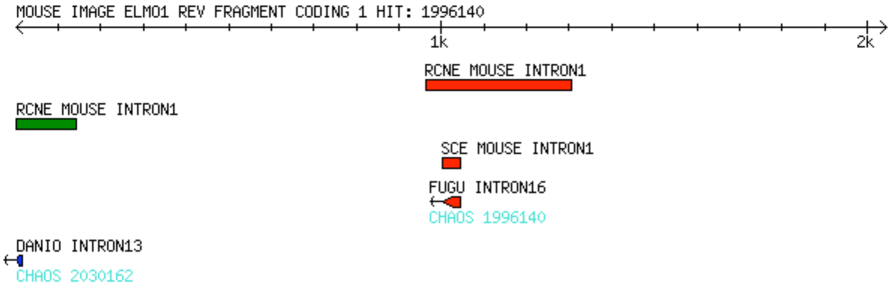

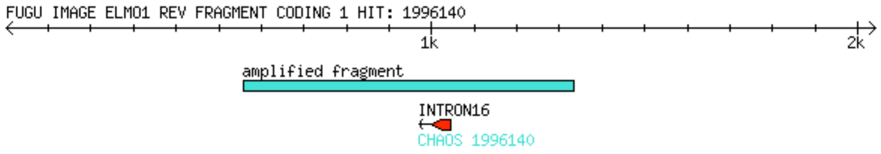

SEQUENCES

>fugu_fragment_for_primer_design_6026
ATGTATTATTCACTCTGACCTCACACATCCAACAGCAACACGAATGTATGCAGATGCTTG
AGACTCATCGGGGAACGTCGACAACATAGTCGTGTCTGGCAGCACATCATTGCAACAGTG
ATGCTGTTGGAACTCCGCCTATAAGGGGGAAAAAACCACCTACATGGTGCATCAGATGTG
ACTGACATAATTCTCTGGTGTGTTGTCTTGTTTTTCTGAAGAXXXXXXXXXXXXXXXXXX
XXXXXXXXXXXXXXXXXXXXXXXXXXXXXXXXXXXXXXXXXXXXXXXXXXXXXXXXXXXX
XXXXXXXXXXXXXXXXXXXXXXXXXXXXXXXXXXXTGAGGCCCGCGGTCACGGCATGATC
CAGCCTCCCCCGCACGACCCAAACGTCTTTCTGCACGTTTTGGTCTTTGGCTGAGGTTTA
ATGGCAGGAAGATGGTAGAGAGAAACCACGACGTCTCACTCTGAGCGTCCGTTCACTCCT
GCTTTCACGGCATTAAAACCCCACTGAAGATGAGGCCTTTGGACAAGCTGCAGAGCTGCC
GTCATGGCGGCTCCCGCCAATATTTCCCATCAGCCTTTGAGGCCAGGATGTCCTGCAGCA
CAGCACCTCGCTCAACACAACTCGCATCTTTTTAAAATCTACAATCTGGCACGATAAAGC
AACTCATTGTATCCAGGGGAAATGATTAGGGTCATTAAATTTGTCTGATTTATCACATGT
GCTGATAAAGATCACACCAGAACATGCTTTGTTTTTTAAGCGAATCAGCAAATATTAATT
TTCCCCATCCTCTTGCTGGATGTCTGATTGTATCCATGACAACGATCCGGTAGCAGCAAG
CGGCATATTTTAATACGGTCATTGCGGTAAAATGCTGGCAAGAGTAAGGTGACATTAAAA
GCGTGCAGGAGGAGCAGCTGTGGGTAGCAGAAGAGAACGCGGTCGGGGTCCGATCCCAAC
AACAGGTGCAAAGGATCAGCAGTCAAAGACGGGCAGATTTGGGAGCAGCTCAGCCAGATG
TTTCACATCCATGATAAGAACAGGAGCTGAGGTCCGTCTGTCCGTCTGTCCCAACAACAC
ACAAAATCAAACGGCCACTGTTGGTTTCCCTGAAGCTTCAGTCAGACCGAAGAGAACCGG
ACGTCGCTCTACTCCTCTTTACCCCCCCAGGTTCATCGATAGATTTTAAATGTCTGACAA
GGATTCGTCTTGACAGCTGTCGCCCAGATGTTGCCAGAGCTGCCTGATGGAAATAGAGAG

AGCCTAAGGAGAGCGAAGGGGCGGACGGGCTGATTTATGCTGGGCTGGCTTTTAAGACAT
TGTGACGCGTGCGCGCTCCCGTGCACAGAGTGAAGGCCTGCAGGGTTACACCAGTTTCCA
CTTTTCCTTCTACACTCATGGAGATGCTCTCATCTCCTCCGCTGGGGGTCTTTGCTCCTC
ATCTTCTCCTCCCTCCTTCCCTGAAAGGCTCTTTGTCGGGCAGAAACTGCAAGAATAAAA
GGGCGTTAAATTCCCTCCTGGTTTAAGAGGCCGTGGCAAACGTTCTCAAACGTTTAACTG
CCCCCCTCCGTTGTCTTGTTACCCCCCTCCTCCACCCTCCCATTTTAGTACCTGCATCAA
AGATTCATTTCGCCAACTTCAGCCCGGATGACTGGGCTCTTCCCCCCGCCTTCCCCTCAC
ATCGGACGTGTTCCGAAGAAAAAGCAGGAGCCCAAATTTGTCAAATTTGGCCCCATCCTC
TTGGATCTCACTCCACTGCCCACGTCCTGGCTACAGATTCTTATTTAGATAATTATAGCT
GAAGAGTCTGATCCTAGAGGGAGATGCACGCATCTATTAAATGCCAGCTGAAGAACATAT
GCGCCCGCACACGTCCACGTGAATCAGCCTCGCTCAGTTGAGCCGTGAGGCTCGGAAAAG
TCTCCTGTCACAGCCTCCCCCGTGGACCTGACTGCTTCCTGCACGCCAGGTACGACCAGA
TGTCTATTTTCAGCAATTAGGATTTGGACGGCGCCGCACCGGCCTCCCCGGTCTGAGCGC
GCT

>amplified_fugu_segment
CCAATATTTCCCATCAGCCTTTGAGGCCAGGATGTCCTGCAGCACAGCACCTCGCTCAAC
ACAACTCGCATCTTTTTAAAATCTACAATCTGGCACGATAAAGCAACTCATTGTATCCAG
GGGAAATGATTAGGGTCATTAAATTTGTCTGATTTATCACATGTGCTGATAAAGATCACA
CCAGAACATGCTTTGTTTTTTAAGCGAATCAGCAAATATTAATTTTCCCCATCCTCTTGC
TGGATGTCTGATTGTATCCATGACAACGATCCGGTAGCAGCAAGCGGCATATTTTAATAC
GGTCATTGCGGTAAAATGCTGGCAAGAGTAAGGTGACATTAAAAGCGTGCAGGAGGAGCA
GCTGTGGGTAGCAGAAGAGAACGCGGTCGGGGTCCGATCCCAACAACAGGTGCAAAGGAT
CAGCAGTCAAAGACGGGCAGATTTGGGAGCAGCTCAGCCAGATGTTTCACATCCATGATA
AGAACAGGAGCTGAGGTCCGTCTGTCCGTCTGTCCCAACAACACACAAAATCAAACGGCC
ACTGTTGGTTTCCCTGAAGCTTCAGTCAGACCGAAGAGAACCGGACGTCGCTCTACTCCT
CTTTACCCCCCCAGGTTCATCGATAGATTTTAAATGTCTGACAAGGATTCGTCTTGACAG
CTGTCGCCCAGATGTTGCCAGAGCTGCCTGATGGAAATAGAGAGAGCCTAAGGAGAGCGA
AGGGGCGGACGGGCTGATTTATGCTGGGCTGGCTTTTAAGACATTGTGACGCGTGCG

>rCNE_mouse_sequence_1764573
GTGGCTGGCAGATTTAGAGAACTGCGGCCCATAGTACCCTGTCACTGACAGGTGTGTGGG
TACATCAGCTGGGCTGCTGCCAGCTGCAGCAGGCAGGTGGCAAGGCAGAAACGTGGACAG
CAACAGATTGGAAAGCTCGAAGGTGAACAAGGGGGCTGGGTAAAGTCCCCAAGCCAGGAG
TGTTGGTTGGCAGTCAGGAACAGGTGGCTGTGTAGCCAGGAGCAGCCAGGATCATCCTGT
GCTGGACTGGGAGGATCTCTGATGAGGGATAGATAACAGCTGAGAGGTCTCCTGGGAAAG
TGCCTCCTGGCAGGCTAGCCTGGCTTCCTCCTAAACAAACAGCC

>SCE_mouse_sequence_1764967
CCTGTCACTGACAGGTGTGTGGGTACATCAGCTGGGCTGCTGCCA

FUGU HITS IN THE CONTEST OF MAMMALIAN rCNE

CLUSTAL W (1.83) multiple sequence alignment

```
ENSG00000155849         GTGGATGTCAGAGCT-GAGAACGGCAGCCCATGCTCCATAGTGGCTAAAACAGGTGTGTG
ENSRNOG00000018726      GTGGCTGGCAGATTTAGAGAACTGCAGCCCATAGTGCCCTGTCACTGG--CAGGTGTGTG
ENSMUSG00000041112      GTGGCTGGCAGATTTAGAGAACTGCGGCCCATAGTACCCTGTCACTGA--CAGGTGTGTG
SINFRUG00000150659      ------------------------------------CCTGTTCTTAT--CATGGATGTG
                                                                **   *    ** *  ****

ENSG00000155849         GGCACATTGGCTGGGGGCGCTGCCAGTCTCAGCAGGCCAGTGTCAAGGCAGAAATGTGGG
ENSRNOG00000018726      GGTACATCAGCTGGGT-TACTGCCAGCTGCAGCAGGCAGGTGGCAAGGCAGAAACGTGGA
ENSMUSG00000041112      GGTACATCAGCTGGGC-TGCTGCCAGCTGCAGCAGGCAGGTGGCAAGGCAGAAACGTGGA
SINFRUG00000150659      AAACATCTGGCTGAGC-TGCTCCCA-----------------------------------
                                 **** *    ** ***

ENSG00000155849         CAGCAACAGACTTGAGCTCTCCAAGGTGAGCATTTGGGCTTAGTAAAGTCCCCAAGCCAG
ENSRNOG00000018726      CAGCAGCAGATTGGAATGCTCAAAGGTGAACAG--GGTCTGCCTAAAGTCCCGGAGCCAG
ENSMUSG00000041112      CAGCAACAGATTGGAAAGCTCGAAGGTGAACAAGGGGGCTGGGTAAAGTCCCCAAGCCAG
SINFRUG00000150659      ------------------------------------------------------------

ENSG00000155849         GTGCCTTGGCCAACAGACAGGAACAGGTGGCTGCGCAGCCAGGGCCTGCCAGTGCTCATC
ENSRNOG00000018726      GAGTGTTGGATGGCAGTCAGGAACAGGTGGCCGAGTAGCCAGGATCATCCTGTGCT----
```

```
ENSMUSG00000041112      GAGTGTTGGTTGGCAGTCAGGAACAGGTGGCTGTGTAGCCAGGAGCAGCCAG-GATCATC
SINFRUG00000150659      ------------------------------------------------------------


ENSG00000155849         AGAGGCGGGGCAGGGAGGGGCTCTGATGAGGGCTG----ACAGCTGGGGGGTCTCCTGGG
ENSRNOG00000018726      -------GGACCGGGAGGATCTCTGATGAGGGATA----ACAGCTGAGAGGTCTCCTGGG
ENSMUSG00000041112      CTGTGCTGGACTGGGAGGATCTCTGATGAGGGATAGATAACAGCTGAGAGGTCTCCTGGG
SINFRUG00000150659      ------------------------------------------------------------


ENSG00000155849         AAGGTGCTTCCAGGAAGGCTGGACTGGCCATCTCCTAAATAAACAGCC
ENSRNOG00000018726      GAAGAGCCTCCTGGCAGACTGGCTTGGCTTCCTCTTAAATAAATAGCC
ENSMUSG00000041112      AAAGTGCCTCCTGGCAGGCTAGCCTGGCTTCCTCCTAAACAAACAGCC
SINFRUG00000150659      ------------------------------------------------
```
