# Supplementary figures and images for: Shuffling of cis-regulatory elements is a pervasive feature of the vertebrate lineage
Source: Genome Biol. 2006 Jul 19;7(7):R56. doi: 10.1186/gb-2006-7-7-r56 (PMC1779573; doi:10.1186/gb-2006-7-7-r56)

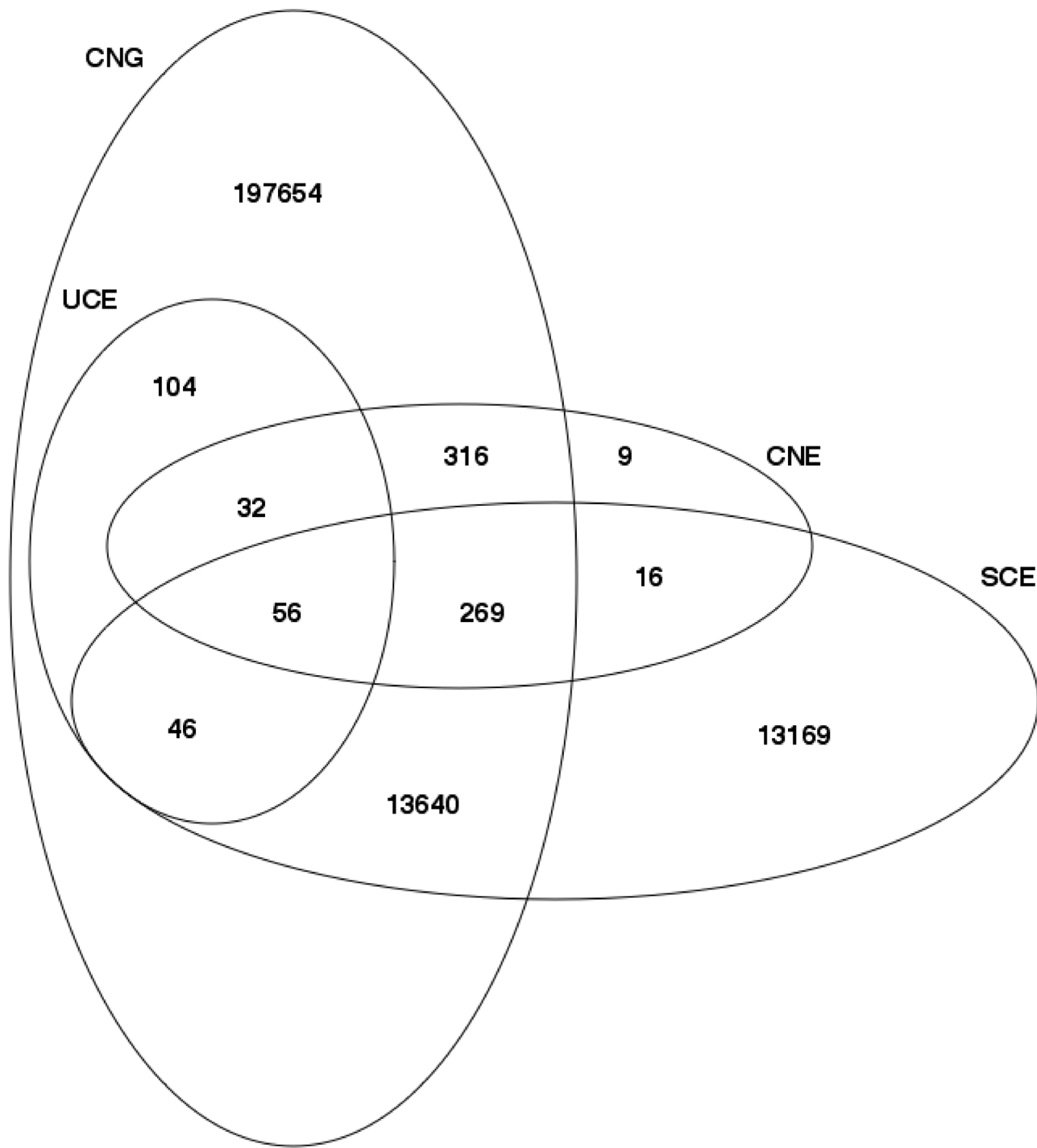

Supplement: Additional data file 5 — A figure showing a Venn diagram that illustrates the overlap analysis of four datasets (CNGs, UCEs, CNEs and SCEs) [file gb-2006-7-7-r56-S5.pdf]

**A****CHAOS**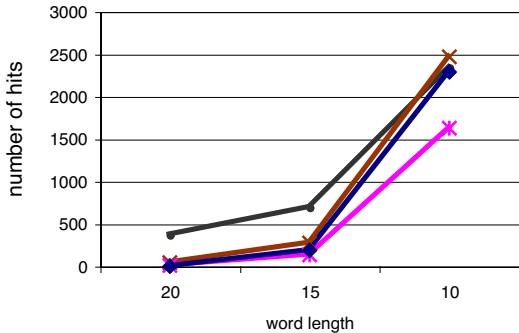**B****BLAST**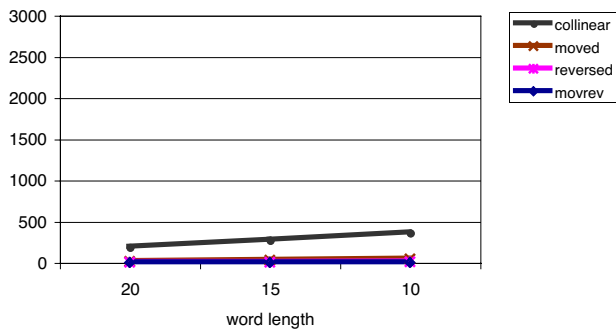

Supplement: Additional data file 6 — A figure showing the number and type of conserved elements identified by CHAOS and BLAST2 in our dataset as a function of the word size used [file gb-2006-7-7-r56-S6.pdf]
